# Supplementary material for: Seeking culturally safe care: a qualitative systematic review of the healthcare experiences of women and girls who have undergone female genital mutilation/cutting
Source: BMJ Open. 2019 May 29;9(5):e027452. doi: 10.1136/bmjopen-2018-027452 (PMC6549627; doi:10.1136/bmjopen-2018-027452)
Supplement: Supplementary data [file bmjopen-2018-027452supp001.pdf]

Supplementary File 1: Medline Search Strategy

Ovid Medline In-Process & Other Non-indexed Citations and Ovid Medline 1946 to present – run on 04/08/2017

Search statement	Search terms	Hits	Notes on strategy
1	exp Circumcision, Female/ or (exp Genitalia, Female/ and exp Medicine, Traditional/)	1367	
2	("female genital mutilation" or "female circumcision" or "female genital cutting" or "traditional female genital surgery" or infibulat* or reinfibulat* or defibulat* or clitoridect*).mp.	1509	
3	((((ritual* or traditional* or ceremon* or sociali#ation*) adj3 (practic* or cut or cutting or surg*)) or circumcis* or excis*) adj3 (female* or wom#n or girl* or child* or adolescen*)).mp.	2924	
4	(FGM or FGC or TFGS or FGMC or "FGM/C").mp.	1133	
5	(thara or tahar or khitan or khifad or khafad or megrez or absum or mekhnishab or kutairi or ibi or ugwu or sunna or bondo or sonde or gudiniin or halalays or qodiin or tahoor or bagne or gadja or ganza or "fanadu di mindjer" or niaka or kuyango or "musolula karoola").mp. not (thara or tahar or khitan or khifad or khafad or megrez or absum or mekhnishab or kutairi or ibi or ugwu or sunna or bondo or sonde or gudiniin or halalays or qodiin or tahoor or bagne or gadja or ganza or "fanadu di mindjer" or niaka or kuyango or "musolula karoola").au,in.	1650	Expanded terms to include country of origin terminology as described in Department of Health guidance.[121]
6	4 or 5	2779	
7	limit 6 to female	1200	In order to exclude irrelevant records from terminology from other disciplines, or male circumcision related hits
8	((FGM or FGC or TFGS or FGMC or "FGM/C" or thara or tahar or khitan or khifad or khafad or megrez or absum or mekhnishab or kutairi or ibi or ugwu or sunna or bondo or	507	Adjacency to "female" terms to complement

Search statement	Search terms	Hits	Notes on strategy
	sonde or gudiniin or halalays or qodiin or tahoor or bagne or gadja or ganza or "fanadu di mindjer" or niaka or kuyango or "musolula karoola") adj3 (female* or wom#n or girl* or child* or adolescen*)).mp.		"limit to female" hit set
9	(egypt* or sudan* or ethiopi* or somali* or djibouti* or eritrea* or burkin* or guinea* or sierra leone or leonean* or mauritan* or mali or malian*).ti,ab.	151913	Country of origin used as proxy for FGM/C terms – as FGM/C is so common, inference is that women from these countries will fit an FGM/C profile even without the term being explicitly mentioned
10	limit 9 to female	46657	
11	((egypt* or sudan* or ethiopi* or somali* or djibouti* or eritrea* or burkin* or guinea* or sierra leone or leonean* or mauritan* or mali or malian*) adj3 (female* or wom#n or girl* or child* or adolescen*)).mp.	5441	
12	1 or 2 or 3 or 7 or 8 or 10 or 11	51901	All women with FGM/C
13	"Emigrants and Immigrants"/ or Refugees/ or "Transients and Migrants"/ or "Emigration and Immigration"/	45756	
14	(immigrant* or immigrat* or emigrant* or emigrat* or emigre* or expat* or (ex adj pat*) or transients or newcomer* or (new adj comer*) or alien* or incomer* or (in adj comer*)).mp.	79630	
15	((immigrant* or immigrat* or emigrant* or emigrat* or emigre* or expat* or (ex adj pat*) or transients or newcomer* or (new adj comer*) or alien* or incomer* or (in adj comer*)) adj3 (female* or wom#n or girl* or child* or adolescen*)).mp.	4382	
16	(refugee* or (asylum adj seek*) or asylee* or (refused adj3 (asylum* or refugee*)) or (displaced adj person*) or exile* or (new adj	17539	

Search statement	Search terms	Hits	Notes on strategy
	arrival) or (country adj2 (birth or origin)) or transnational*).mp.		
17	((refugee* or (asylum adj seek*) or asylee* or (refused adj3 (asylum* or refugee*)) or (displaced adj person*) or exile* or (new adj arrival) or (country adj2 (birth or origin)) or transnational*) adj3 (female* or wom#n or girl* or child* or adolescen*)).mp.	1462	
18	(foreigner* or (foreign adj (born or citizen* or national* or origin*)) or (non adj (citizen* or native*)) or ((adoptive or naturali#ed) adj (citizen* or resident*)) or overstay* or trafficked or "spousal migrant*").mp	11442	
19	((foreigner* or (foreign adj (born or citizen* or national* or origin*)) or (non adj (citizen* or native*)) or ((adoptive or naturali#ed) adj (citizen* or resident*)) or overstay* or trafficked or "spousal migrant*") adj3 (female* or wom#n or girl* or child* or adolescen*)).mp.	753	
20	(((established or long-term or "first generation*" or new* or recent* or current*) adj3 (migrant* or migrat* or immigrant* or immigrat* or emigrant* or emigrat* or emigre* or expat* or (ex adj pat*) or transient* or alien*)) or newcomer* or (new adj comer*) or incomer* or (in adj comer*) or ((international or overseas or foreign) adj2 (student* or employee* or worker*))).mp.	18239	
21	((((established or long-term or "first generation*" or new* or recent* or current*) adj3 (migrant* or migrat* or immigrant* or immigrat* or emigrant* or emigrat* or emigre* or expat* or (ex adj pat*) or transient* or alien*)) or newcomer* or (new adj comer*) or incomer* or (in adj comer*) or ((international or overseas or foreign) adj2 (student* or employee* or worker*))) adj3 (female* or wom#n or girl* or child* or adolescen*)).mp.	559	
22	("non-UK-born" or "born outside the UK" or "length of residence in the UK" or (("not lawful*" or "not legal*" or unlawful* or illegal* or unauthori#ed* or "not authori#ed" or uncertain or insecure or legal* or legitimate* or permit* or visa* or irregular* or refused or undocumented) adj3 (residen* or student* or worker* or employee* or	2887	

Search statement	Search terms	Hits	Notes on strategy
	unemployed or immigrant* or imigrat* or migrant* or migrat*))).mp.		
23	(("non-UK-born" or "born outside the UK" or "length of residence in the UK" or (("not lawful*" or "not legal*" or unlawful* or illegal* or unauthori#ed* or "not authori#ed" or uncertain or insecure or legal* or legitimate* or permit* or visa* or irregular* or refused or undocumented) adj3 (residen* or student* or worker* or employee* or unemployed or immigrant* or imigrat* or migrant* or migrat*))) adj3 (female* or wom#n or girl* or child* or adolescen*)).mp.	136	
24	exp Vulnerable Populations/	8275	
25	((vulnerab* or disadvantag* or minorit*) adj3 (individ* or person* or people* or population* or communit* or group*)).ti,ab.	33112	
26	((vulnerab* or disadvantag* or minorit*) adj3 (individ* or person* or people* or population* or communit* or group*) adj3 (female* or wom#n or girl* or child* or adolescen*)).mp.	1756	
27	exp Ethnic Groups/	139042	
28	(ethnic* or ethno* or race or racial*).mp.	248038	
29	("Black and Minority Ethnic" or "Black & Minority ethnic" or BME or BAME or black african*).mp.	3756	
30	((ethnic* or ethno* or race or racial* or "Black and Minority Ethnic" or "Black & Minority ethnic" or BME or BAME or black african*) adj3 (female* or wom#n or girl* or child* or adolescen*)).mp.	9504	
31	exp african continental ancestry group/ or exp asian continental ancestry group/	136483	
32	(africa* or "middle east*" or asia* or iran* or iraq* or israel* or oman* or "united arab emirat*" or UAE or "saudi arabia*" or palestin* or india* or indonesia* or malaysia* or pakistan*).ti,ab.	520081	
33	(egypt* or yemen* or sudan* or ethiopi* or somali* or djibouti* or eritrea* or kenya* or uganda* or tanzania* or camero?n* or chad* or niger* or benin* or togo* or ghana* or burkin* or ivory coast* or cote d'ivoire or liberia* or guinea* or sierra leone or leonean*	259558	

Search statement	Search terms	Hits	Notes on strategy
	or gambia* or senegal* or mauritan* or mali or malian* or "sara subgroup" or ngama).ti,ab.		
34	((africa* or "middle east*" or asia* or iran* or iraq* or israel* or oman* or "united arab emirat*" or UAE or "saudi arabia*" or palestin* or india* or indonesia* or malaysia* or pakistan* or egypt* or yemen* or sudan* or ethiopi* or somali* or djibouti* or eritrea* or kenya* or uganda* or tanzania* or camero?n* or chad* or niger* or benin* or togo* or ghana* or burkin* or ivory coast* or cote d'ivoire or liberia* or guinea* or sierra leone or leonean* or gambia* or senegal* or mauritan* or mali or malian* or "sara subgroup" or ngama) adj3 (female* or wom#n or girl* or child* or adolescen*)).mp.	58342	
35	13 or 14 or 16 or 18 or 20 or 22 or 24 or 25 or 27 or 28 or 29 or 31 or 32 or 33	1117829	
36	limit 35 to female	491179	Keywords for immigrant populations limited to female (Medline standard limit feature)
37	(13 or 24 or 27 or 31) and (female* or wom#n or girl* or child* or adolescen*).mp.	177101	MeSH Headings for immigrant populations limited by “female” keywords
38	15 or 17 or 19 or 21 or 23 or 26 or 30 or 34 or 36 or 37	526145	Terms for immigrant populations, including specific terms for countries of origin where FGM known to be practised, limited to female
39	exp Community Health Workers/ or exp Volunteers/	23517	All health and allied workers (who may come into contact with women with FGM)
40	exp Occupational Groups/	531564	
41	exp Health Personnel/	454091	
42	exp Midwifery/	17848	

Search statement	Search terms	Hits	Notes on strategy
43	exp General Practitioners/ or exp Physicians/	117502	
44	exp Nurses/ or exp Nurses, Community Health/	81947	
45	exp Nurses' Aides/ or exp Nursing Staff, Hospital/ or exp Nursing Staff/	66494	
46	exp Social Workers/ or exp Police/	4605	
47	39 or 40 or 41 or 42 or 43 or 44 or 45 or 46	562187	
48	exp Pregnancy/ or (pregnan* or birth* or childbirth* or matern* or gyn#e* or obstetric* or menstru* or labo#r* or vulv* or vagina* or uter*).ti,ab.	1391519	Physical complications or disorders as identified as health impacts of FGM in Department of Health guidance,[121] already "female" by context of terms – so proxy for FGM.
49	exp Pregnancy Complications/ or exp Pregnancy High Risk/	401801	
50	((pregnan* or birth* or childbirth* or matern* or gyn#e* or obstetric* or menstru* or labo#r* or vulv* or vagina* or uter*) adj3 (poor* or adverse or complicat* or difficult* or disorder* or dysfunction* or disease* or pain* or risk* or danger* or problem* or issue* or concern* or infect* or inflamm*)).ti,ab.	137220	
51	exp Obstetric Labor Complications/	63618	
52	exp Female Urogenital Diseases/	1161123	
53	exp Menstruation Disturbances/	27153	
54	exp Genital Diseases, Female/ or exp Vaginal Diseases/ or exp Vulvar Diseases/	432325	
55	exp Vaginismus/ or exp Vulvodynia/ or exp Dyspareunia/ or exp Vaginitis/ or exp Vulvovaginitis/	14171	
56	exp Pelvic Inflammatory Disease/	10694	
57	exp Vaginal Fistula/ or exp Rectovaginal Fistula/	4659	
58	exp Genital Diseases, Female/	432325	
59	exp Infertility, Female/	27170	
60	((pelvic* or back* or urolog* or urogenit* or urinat* or genit* or abdomin*) adj3 (poor* or adverse or complicat* or difficult* or	29027	

Search statement	Search terms	Hits	Notes on strategy
	disorder* or dysfunction* or disease* or pain* or risk* or danger* or problem* or issue* or concern* or infect* or inflamm*) adj3 (female* or wom#n or girl* or child* or adolescen*)).mp.		
61	exp Urologic Diseases/	698515	Physical complications or disorders as identified as health impacts of FGM in Department of Health guidance,[121] limited to "female" (female genital diseases, etc.) – so proxy for FGM
62	exp Pelvic Pain/	8163	
63	exp Sexual Dysfunctions, Psychological/	24271	
64	exp Pelvic Infection/	5409	
65	exp Kidney Diseases/	474329	
66	exp Urinary Fistula/ or exp Rectal Fistula/ or exp Urinary Bladder Fistula/	11750	
67	61 or 62 or 63 or 64 or 65 or 66	737520	
68	limit 67 to female	352622	
69	67 and (female* or wom#n or girl* or child* or adolescen*).mp.	397916	
70	48 or 49 or 50 or 51 or 52 or 53 or 54 or 55 or 56 or 57 or 58 or 59 or 60 or 68 or 69	2386342	
71	exp Mental Disorders/	1122844	
72	exp Depressive Disorder, Major/ or exp Suicide/ or exp Depression/ or exp Suicidal Ideation/ or exp Depressive Disorder/ or exp Bipolar Disorder/ or exp Suicide, Attempted/	259964	
73	exp Self-Injurious Behavior/ or exp Substance-Related Disorders/ or exp Social Isolation/	329005	
74	exp Sexually Transmitted Diseases, Viral/ or exp Sexually Transmitted Diseases/	319070	
75	(anxiet* or anxious* or depress* or self-harm* or self-injur* or suicid*).mp.	650968	
76	((anxiet* or anxious* or depress* or self-harm* or self-injur* or suicid*) adj3 (female* or wom#n or girl* or child* or adolescen*)).mp.	30927	
77	(((sex* or viral* or virus* or bacteria*) adj3 diseas*) or HIV or STD* or AIDS).mp.	516543	
78	((((sex* or viral* or virus* or bacteria*) adj3 diseas*) or HIV or STD* or AIDS) adj3	29597	

Search statement	Search terms	Hits	Notes on strategy
	(female* or wom#n or girl* or child* or adolescen*)).mp.		
79	71 or 72 or 73 or 74 or 75 or 77	2107493	
80	limit 79 to female	932164	
81	79 and (female* or wom#n or girl* or child* or adolescen*).mp.	1098666	
82	76 or 78 or 80 or 81	1098666	Psychological or STD related impacts of FGM, as above
83	exp United Kingdom/ or ("united kingdom" or "great britain" or britain or england or scotland or wales or "northern ireland" or british or english or scottish or welsh or "northern irish" or hebrid* or "isle of man").ti,ab,gc,ia,pl,in.	1167455	
84	(AUSTRALIA* or AUSTRIA* or BELGIUM* or belgian* or CANAD* or CHILE* or CZECH* or DENMARK* or danish* or ESTONIA* or FINLAND or finnish or FRANCE or french).mp.	641227	
85	(GERMAN* or GREECE or greek* or HUNGAR* or ICELAND* or IRELAND or irish or ISRAEL* or ITALY or italian* or JAPAN* or KOREA* or LATVIA* or LUXEMB* or MEXIC*).mp.	791022	
86	(NETHERLAND* or holland* or dutch or "low countries" or europe* or "NEW ZEALAND*" or NORWAY or norwegian* or POLAND or polish or PORTUGAL or portuguese).mp.	597994	
87	(SLOVAK* or SLOVENIA* or SPAIN or spanish or SWED* or SWITZERLAND or swiss or TURK* or "UNITED KINGDOM" or "UK" or britain or british or england or english or scot* or wales or welsh or UNITED STATES or "USA" or america*).mp.	3770430	
88	83 or 84 or 85 or 86 or 87	5147200	UK or other OECD countries
89	randomized controlled trial.pt.	470445	
90	controlled clinical trial.pt.	94472	
91	randomized.ab.	403685	

Search statement	Search terms	Hits	Notes on strategy
92	placebo.ab.	189129	
93	clinical trials as topic.sh.	187621	
94	randomly.ab.	280498	
95	trial.ti.	181309	
96	(animals not (humans and animals)).sh.	4412254	
97	89 or 90 or 91 or 92 or 93 or 94 or 95	1138847	
98	97 not 96	1049209	Standard search hedge for randomised clinical trials in Medline – adapted from SIGN.[315]
99	38 and 47 and (70 or 82)	7952	Female immigrants AND Health Workers AND (physical or mental disorders)
100	47 and 12	1144	
101	12 or 99 or 100	59304	Women with FGM or (Female immigrants AND Health Workers AND (physical or mental disorders)) or (Health workers AND FGM)
102	101 and 88	12154	…AND OECD countries
103	102 not 98	11547	EXCLUDE RCTs
104	103 not 96	9261	EXCLUDE animal studies
