# Supplementary material for: Seeking culturally safe care: a qualitative systematic review of the healthcare experiences of women and girls who have undergone female genital mutilation/cutting
Source: BMJ Open. 2019 May 29;9(5):e027452. doi: 10.1136/bmjopen-2018-027452 (PMC6549627; doi:10.1136/bmjopen-2018-027452)
Supplement: Supplementary data [file bmjopen-2018-027452supp002.pdf]

Supplementary File 2: Excluded Studies (with Reasons)

No.	Study	Reason for Exclusion
1.	Abdel Halim AM. Honorable daughters: The lived experience of circumcised Sudanese women in the United States. PhD Thesis, Ann Arbor: Ohio University, 2003	Data presented on FGM/C, but no specific focus on FGM/C related healthcare
2.	Abdel Magied A and Shareef S. Knowledge, perception and attitudes of a sector of female health providers towards FGM - case study: female doctors. *Ahfad Journal*. 2003; 20: 4-17.	Not OECD
3.	Abdulcadir O, Catania L and Caselli A. Female genital mutilation (FGM): a comparison between the male and the female view. *Int J Gynaecol Obstet*. 2012; 119: S262-S3.	Conference abstract
4.	Alizadeh V, Hylander I, Kocturk T and Tornkvist L. Counselling young immigrant women worried about problems related to the protection of "family honour"-From the perspective of midwives and counsellors at youth health clinics. *Scand J Caring Sci*. 2010; 24: 32-40	Data presented on FGM/C, but no specific focus on FGM/C related healthcare
5.	Alizadeh V, Tornkvist L and Hylander I. Counselling teenage girls on problems related to the 'protection of family honour' from the perspective of school nurses and counsellors. *Health Soc Care Community*. 2011; 19: 476-84.	Data on FGM/C not presented
6.	Allag F, Abboud P, Mansour G, Zanardi M and Quereux C. Female genital mutilation. Women's point of view. *Gynecologie Obstetrique Fertilite*. 2001; 29: 824-8.	Data presented on FGM/C, but no specific focus on FGM/C related healthcare
7.	Aquino M, Edge D and Smith DM. Pregnancy as an ideal time for intervention to address the complex needs of black and minority ethnic women: Views of British midwives. *Midwifery*. 2015; 31: 373-9.	Data on FGM/C not presented
8.	Ariyo D, Ssali R, King-Webb L and Ikpaahindi S. Voices of the community: exploring female genital mutilation in the African community across Greater Manchester. London: AFRUCA, 2015.	Data presented on FGM/C, but no specific focus on FGM/C related healthcare
9.	Beck-Karrer C, Levin T and Levin T. Lion women. Conversations with Somalian women and men about female circumcision. *European Journal of Women's Studies*. 1998; 5: 533-6.	Not empirical research
10.	Bergqvist, H. & Svensson, J. (2016) [Midwives Experiences of Encounters wth Young Women who come from Areas Where the Practice of Genital Mutilation is Common] Barnmorskors Erfarenheter Av Möten Med Unga Kvinnor Som Kommer Från Områden Där Kvinnlig Könsstympning är Aanligt Förekommande. University of Skövde, MSc Thesis, Sweden.	Data on healthcare-specific experiences is only from health professionals, not women/girls

No.	Study	Reason for Exclusion
11.	Bibi, N. & Rahimian, N. (2013) [Nurses' Experience and Knowledge about Female Genital Mutilation] Sjuksköterskans Erfarenheter Och Kunskaper Om Kvinnlig Könssympning. Sophiahemmet University, MSc Thesis, Sweden.	Data on healthcare-specific experiences is only from health professionals, not women/girls
12.	Brodin, E. & Mårtensson, N. (2016) [District Nurses' Knowledge and Experience of Female Genital Mutilation] Distriktssköterskors Kunskap Och Erfarenhet Av Kvinnlig Könsstympning. University of Örebro, MSc Thesis, Sweden.	Data on healthcare-specific experiences is only from health professionals, not women/girls
13.	Brown E, Carroll J, Fogarty C and Holt C. "They get a C-section . . . they gonna die": Somali women's fears of obstetrical interventions in the United States. *J Transcult Nurs*. 2010; 21: 220-7.	Data on FGM/C not presented
14.	Brown E and Porter C. The tackling FGM initiative: evaluation of the second phase (2013-2016). Options UK, Report. 2016.	Not empirical research
15.	Buckland RL. The everyday experience of Somali women in Canada: Implications for health. MSc Thesis, University of Ottawa (Canada), 1997	Data presented on FGM/C, but no specific focus on FGM/C related healthcare
16.	Bulman, K. & McCourt, C. (1997) Report on Somali Womens' Experiences of Maternity Services. Centre for Midwifery Practice, Wolfson Institute of Health Sciences, Thames Valley University and Hammersmith Hospitals National Health Service Trust, London, UK.	Data on healthcare-specific experiences is only from health professionals, not women/girls
17.	Burchill J. Safeguarding vulnerable families: work with refugees and asylum seekers. *Community Practitioner*. 2011; 84: 23-6.	Data presented on FGM/C, but no specific focus on FGM/C related healthcare
18.	Burchill, J. & Pevalin, D.J. (2014) Demonstrating cultural competence within health-visiting practice: working with refugee and asylum-seeking families. *Divers Equal Health Care,* 11(2), 151-159.	Data on healthcare-specific experiences is only from health professionals, not women/girls
19.	Byrskog, U., Olsson, P., Essen, B. & Allvin, M.K. (2015) Being a bridge: Swedish antenatal care midwives' encounters with Somali-born women and questions of violence - a qualitative study. *BMC Pregnancy Childbirth,* 15(1).	Data on healthcare-specific experiences is only from health professionals, not women/girls
20.	Carolan M. Antenatal care perceptions of pregnant African women attending maternity services in Melbourne, Australia. *Midwifery*. 2010; 26: 189-201.	Data on FGM/C not presented
21.	Chalmers B and Hashi KO. 432 Somali women's birth experiences in Canada after earlier female genital mutilation. *Birth*. 2000; 27: 227-34.	Not qualitative research
22.	Christiansen CD. The lived experience of circumcision in immigrant Somali women: a Heideggerian hermeneutic analysis. MA Thesis, University of Illinois, 1995,	Data presented on FGM/C, but no specific focus on FGM/C related healthcare

No.	Study	Reason for Exclusion
23.	Clarke ALF. The process of changed meanings: A study of the Canadian experience of Somali women in the Kitchener-Waterloo area in regard to female circumcision. PhD Thesis, Ann Arbor: Wilfrid Laurier University (Canada), 1995,	Not empirical research
24.	Clayton-Hathway K. A Pilot Evaluation of Health Services for Communities Affected by FGM in Oxfordshire: Final Report. Oxford, UK: Healthwatch Oxfordshire, 2016.	Not qualitative research
25.	Connor JJ, Hunt S, Finsaas M, Ciesinski A, Ahmed A and Robinson BBE. Sexual Health Care, Sexual Behaviors and Functioning, and Female Genital Cutting: Perspectives From Somali Women Living in the United States. *J Sex Res*. 2016; 53: 346-59.	Data presented on FGM/C, but no specific focus on FGM/C related healthcare
26.	Dahlen UM. Female Genital Cutting: Phenomenological Interviews on the Ethiopian Immigrant Mothers' Experience. Masters Thesis, Regent University, 2011,	Data presented on FGM/C, but no specific focus on FGM/C related healthcare
27.	Dawson, A.J., Turkmani, S., Varol, N., Nanayakkara, S., Sullivan, E. & Homer, C.S. (2015) Midwives' experiences of caring for women with female genital mutilation: insights and ways forward for practice in Australia. *Women Birth,* 28(3), 207-14.	Data on healthcare-specific experiences is only from health professionals, not women/girls
28.	Degni F, Suominen S, Essen B, El Ansari W and Vehvilainen-Julkunen K. Communication and cultural issues in providing reproductive health care to immigrant women: health care providers' experiences in meeting the needs of Somali women living in Finland. *Journal of Immigrant and Minority Health*. 2012; 14: 330-43.	Data on FGM/C not presented
29.	Essen B, Binder P and Johnsdotter S. An anthropological analysis of the perspectives of Somali women in the West and their obstetric care providers on caesarean birth. *Journal of Psychosomatic Obstetrics and Gynecology*. 2011; 32: 10-8.	Data on FGM/C not presented
30.	Essen B, Johnsdotter S and Binder P. Not too far to walk but too far for reciprocity: Maternal mortality in a migration context using the 'three delays' framework. *International Journal of Gynecology and Obstetrics*. 2012; 119: S343.	Conference abstract
31.	Feldman R. When maternity doesn't matter: Dispersing pregnant women seeking asylum. *British Journal of Midwifery*. 2014; 22: 23-8.	Data on FGM/C not presented
32.	Finnstrom B and Soderhamn O. Conceptions of pain among Somali women. *J Adv Nurs*. 2006; 54: 418-25.	Data presented on FGM/C, but no specific focus on FGM/C related healthcare
33.	García Aguado S and Sánchez López MI. Knowledge of healthcare professionals about female genital mutilation. *Metas de Enfermería*. 2013; 16: 18-22.	Not qualitative research
34.	Gele AA, Kumar B, Hjelde KH and Sundby J. Attitudes toward female circumcision among Somali immigrants	Data presented on FGM/C, but no specific

No.	Study	Reason for Exclusion
	in Oslo: a qualitative study. *International Journal of Women's Health*. 2012; 4: 7-17.	focus on FGM/C related healthcare
35.	Gele AA, Sagbakken M and Kumar B. Is female circumcision evolving or dissolving in Norway? A qualitative study on attitudes toward the practice among young Somalis in the Oslo area. *International Journal of Women's Health*. 2015; 7: 933-43.	Data presented on FGM/C, but no specific focus on FGM/C related healthcare
36.	Gerrish K, Naisby A and Ismail M. Experiences of the diagnosis and management of tuberculosis: A focused ethnography of Somali patients and healthcare professionals in the UK. *J Adv Nurs*. 2013; 69: 2285-94.	Data on FGM/C not presented
37.	Gertsson, M. & Serpan, H. (2009) [Meeting with the Unthinkable: Value Conflicts in Meetings with Women Vulnerable to Female Genital Mutilation] Mötet Med Det Otänkbara: Värdekonflikter I Mötet Med Kvinnor Utsatta för Kvinnlig Könsstympning. University of Kalmar, MSc Thesis, Sweden.	Data on healthcare-specific experiences is only from health professionals, not women/girls
38.	Glavin K and Sæteren B. Cultural Diversity in Perinatal Care: Somali New Mothers' Experiences with Health Care in Norway. *Health Science Journal*. 2016; 10: 1-9.	Data on FGM/C not presented
39.	Glazer E. Gendering chronic pain: Socio-cultural embodiment and circumcision in Somali women in Toronto. *Gend Med*. 2010; 7: 528.	Conference abstract
40.	Goldblatt H. Caring for abused women: Impact on nurses' professional and personal life experiences. *J Adv Nurs*. 2009; 65: 1645-54.	Data on FGM/C not presented
41.	Hai DNT and Ghebre R. Comparison of barriers to cervical cancer screening among Somali and Vietnamese women. *J Womens Health*. 2013; 22: 36.	Conference abstract
42.	Henderson A. Factors influencing nurses' responses to abused women: What they say they do and why they say they do it. *Journal of Interpersonal Violence*. 2001; 16: 1284-306.	Data on FGM/C not presented
43.	Herrel N, Olevitch L, DuBois DK, et al. Somali refugee women speak out about their needs for care during pregnancy and delivery. *Journal of Midwifery & Women's Health*. 2004; 49: 345-9.	Data on FGM/C not presented
44.	Holm, L. & Kammensjö, H. (2012) [School Nurses' Experiences of Female Genital Mutilation among Girls] Skolsköterskors Upplevelser Kring Kvinnlig Könsstympning Bland Flickor. University of Skövde, MSc Thesis, Sweden.	Data on healthcare-specific experiences is only from health professionals, not women/girls
45.	Houston AR. (In)visible embodiment: Somali perspectives of diabetes and mental health in diaspora. MSc Thesis, Boston University, 2016,	Data presented on FGM/C, but no specific focus on FGM/C related healthcare
46.	Hoyt MA. In her own words: An exploration of immigrant women's experiences of reproductive health	Data on FGM/C not presented

No.	Study	Reason for Exclusion
	care through ethnographic narrative. MA Thesis, University of New Brunswick (Canada), 2010,	
47.	Isman E, Ekeus C and Berggren V. Perceptions and experiences of female genital mutilation after immigration to Sweden: an explorative study. *Sexual and Reproductive Healthcare*. 2013; 4: 93-8.	Data presented on FGM/C, but no specific focus on FGM/C related healthcare
48.	Jacoby SD, Lucarelli M, Musse F, Krishnamurthy A and Salyers V. A Mixed-Methods Study of Immigrant Somali Women's Health Literacy and Perinatal Experiences in Maine. *Journal of Midwifery and Women's Health*. 2015; 60: 593-603.	Data presented on FGM/C, but no specific focus on FGM/C related healthcare
49.	Jatau, M. (2011) Living Between Two Cultures: A Reproductive Health Journey of African Refugee Women. Arizona State University, PhD Thesis, USA.	Data on healthcare-specific experiences is only from health professionals, not women/girls
50.	Johansen REB. Experiencing sex in exile: can genitals change their gender? On conceptions and experiences related to female genital cutting (FGC) among Somalis in Norway. In: *Transcultural Bodies: Female Genital Cutting In Global Context Ed Ylva Hernlund & Bettina Shell-Duncan*. Rutgers University Press, 2007, p. 248-77.	Data presented on FGM/C, but no specific focus on FGM/C related healthcare
51.	Johansen, R.E. (2006) Care for infibulated women giving birth in Norway: an anthropological analysis of health workers' management of a medically and culturally unfamiliar issue. *Med. Anthropol. Q.,* 20(4), 516-44.	Data on healthcare-specific experiences is only from health professionals, not women/girls
52.	Johansen, R.E. (2017) Virility, pleasure and female genital mutilation/cutting: a qualitative study of perceptions and experiences of medicalized defibulation among Somali and Sudanese migrants in Norway. *Reprod Health,* 14(1), 25.	Data on healthcare-specific experiences is only from health professionals, not women/girls
53.	Johnsdotter S, Ingvarsdotter K, Ostman M and Carlbom A. Koran reading and negotiation with jinn: strategies to deal with mental ill health among Swedish Somalis. *Mental Health, Religion & Culture*. 2011; 14: 741-55.	Data on FGM/C not presented
54.	Johnsdotter S and Essén B. It is only a tradition: making sense of Swedish Somalis' narratives of female circumcision and avoiding submission to hegemonic political discourse. *Crit Soc Policy*. 2005; 25: 577-89.	Not empirical research
55.	Johnsdotter S and Essen B. Cultural change after migration: Circumcision of girls in Western migrant communities. *Best Practice and Research: Clinical Obstetrics and Gynaecology*. 2016; 32: 15-25.	Not empirical research
56.	Johnsdotter S, Moussa K, Carlbom A, Aregai R and Essen B. "Never my daughters": A qualitative study	Data presented on FGM/C, but no specific

No.	Study	Reason for Exclusion
	regarding attitude change toward female genital cutting among Ethiopian and Eritrean families in Sweden. *Health Care Women Int*. 2009; 30: 114-33.	focus on FGM/C related healthcare
57.	Johnson-Agbakwu CE, Helm T, Killawi A and Padela AI. Perceptions of obstetrical interventions and female genital cutting: insights of men in a Somali refugee community. *Ethn Health*. 2014; 19: 440-5	Focus on men, not women
58.	Kallon I and Dundes L. The cultural context of the Sierra Leonean Mende woman as patient. *J Transcult Nurs*. 2010; 21: 228-36.	Not empirical research
59.	Kaplan-Marcusan A, Del Rio NF, Moreno-Navarro J, et al. Female genital mutilation: perceptions of healthcare professionals and the perspective of the migrant families. *BMC Public Health*. 2010; 10: 193.	Not qualitative research
60.	Kay M, Wijayanayaka S, Cook H and Hollingworth S. Understanding quality use of medicines in refugee communities in Australian primary care: A qualitative study. *Br J Gen Pract*. 2016; 66: e397-e409.	Data on FGM/C not presented
61.	Koukoui S, Hassan G and Guzder J. The mothering experience of women with FGM/C raising 'uncut' daughters, in Ivory Coast and in Canada. *Reproductive Health*. 2017; 14: 1-11.	Data presented on FGM/C, but no specific focus on FGM/C related healthcare
62.	Lazar J, Shipp M and Johnson C. Provider perceptions of sexual desire and dyspareunia among Somali women with female genital cutting. *J Sex Med*. 2010; 7: 148.	Conference abstract
63.	Lazar, J.N., Johnson-Agbakwu, C.E., Davis, O.I. & Shipp, M.P.L. (2013) Providers' perceptions of challenges in obstetrical care for Somali women. *Obstet. Gynecol. Int.,* 2013, 149640.	Data on healthcare-specific experiences is only from health professionals, not women/girls
64.	Lane J and Cole G. Pregnancy, prenatal care and delivery perceptions and beliefs of resettled African refugee women in the western United States. *Contraception*. 2013; 88: 313.	Conference abstract
65.	Leishman J. Perspectives of cultural competence in health care. *Nurs Stand*. 2004; 19: 33	Data on FGM/C not presented
66.	León-Larios, F. & Casado-Mejía, R. (2012) [Influence of gender on knowledge, perception and approach to harmful traditional practices: female genital mutilation]. *Evidentia,* 9(40), 1-7.	Data on healthcare-specific experiences is only from health professionals, not women/girls
67.	Lephard E and Haith-Cooper M. Pregnant and seeking asylum: Exploring women's experiences 'from booking to baby'. *British Journal of Midwifery*. 2016; 24: 130-6.	Data on FGM/C not presented
68.	Leval, A., Widmark, C., Tishelman, C. & Maina Ahlberg, B. (2004) The encounters that rupture the myth: contradictions in midwives' descriptions and explanations of circumcised women immigrants' sexuality. *Health Care Women Int.,* 25(8), 743-60.	Data on healthcare-specific experiences is only from health professionals, not women/girls

No.	Study	Reason for Exclusion
69.	Lewig K, Arney F and Salveron M. Challenges to parenting in a new culture: Implications for child and family welfare. *Eval Program Plann*. 2010; 33: 324-32.	Data on FGM/C not presented
70.	Leye E. Midwifery training needs identified when caring for women with female genital mutilation. *Evid Based Nurs*. 2016; 19: 7.	Not empirical research
71.	Liao LM, Elliott C, Ahmed F and Creighton SM. Adult recall of childhood female genital cutting and perceptions of its effects: a pilot study for service improvement and research feasibility. *J Obstet Gynaecol*. 2013; 33: 292-5.	Not qualitative research
72.	Lien IL and Schultz JH. Internalizing knowledge and changing attitudes to female genital cutting/mutilation. *Obstet Gynecol Int*. 2013; 2013: 467028.	Data presented on FGM/C, but no specific focus on FGM/C related healthcare
73.	McCarthy R and Haith-Cooper M. Evaluating the impact of befriending for pregnant asylum-seeking and refugee women. *British Journal of Midwifery*. 2013; 21: 404-9.	Data on FGM/C not presented
74.	McLeish J. Maternity experiences of asylum seekers in England. *British Journal of Midwifery* 2005; 13: 782-5.	Data on FGM/C not presented
75.	Mitello L and Proietti A. Feminine genitals modification: survey of a new phenomenon for nurse today. Second Part. *Prof Inferm*. 2006; 59: 242-5.	Not qualitative research
76.	Mitello L and Proietti A. Female genital modification: survey of new phenomenon for nurses today. First Part. *Prof Inferm*. 2006; 59: 171-81.	Not empirical research
77.	Moore, K. (2012) Female Genital Mutilation and Cultural Competency: Moving Towards Improved Management of Obstetric Care. Queen Margaret University, MSc Thesis, Edinburgh.	Data on healthcare-specific experiences is only from health professionals, not women/girls
78.	Morris MD, Popper ST, Rodwell TC, Brodine SK and Brouwer KC. Healthcare barriers of refugees post-resettlement. *J Community Health*. 2009; 34: 529-38.	Data on FGM/C not presented
79.	Morison L and others. How experiences and attitudes relating to female circumcision vary according to age on arrival in Britain: a study among young Somalis in London. *Ethnicity & Health*. 2004; 9: 75-100.	Data presented on FGM/C, but no specific focus on FGM/C related healthcare
80.	Mwangi-Powell F. Female genital mutilation: a case study in Birmingham. FORWARD, Report, 2000.	Data presented on FGM/C, but no specific focus on FGM/C related healthcare
81.	Nash E and Ranka P. Female genital mutilation: Knowledge, confidence, and approach to care in clinical practice of midwives and nurses in the UK. *International Journal of Gynecology and Obstetrics*. 2015; 131: E110.	Not qualitative research
82.	Nur Z. Cultural Competence in Health Care: Exploring the Experiences of Muslim Women within the Ontario	Data on FGM/C not presented

No.	Study	Reason for Exclusion
	Healthcare System. MSc Thesis, University of Ontario Institute of Technology (Canada), 2014,	
83.	Ogbagzy RA. The construction of Eritrean women's identity in the Canadian context. MA Thesis, University of Toronto (Canada), 1999, p. 236.	Data presented on FGM/C, but no specific focus on FGM/C related healthcare
84.	Ogunsiji O. Understanding the dilemma of de-infibulation for women living with female genital mutilation. *Australian Nursing & Midwifery Journal*. 2015; 22: 51	Not empirical research
85.	Ogunsiji O and Wilkes L. Cultural explanations and procedures about female genital mutilation. *Australian Nursing & Midwifery Journal*. 2015; 22: 51	Data presented on FGM/C, but no specific focus on FGM/C related healthcare
86.	Ogunsiji O, Wilkes L, Peters K and Jackson D. Knowledge, attitudes and usage of cancer screening among West African migrant women. *J Clin Nurs*. 2013; 22: 1026-33.	Data on FGM/C not presented
87.	Ogunsiji, O. (2015) Female genital mutilation (FGM): Australian midwives' knowledge and attitudes. *Health Care Women Int.,* 36(11), 1179-1193.	Data on healthcare-specific experiences is only from health professionals, not women/girls
88.	Ogunsiji, O. (2016) Australian midwives' perspectives on managing obstetric care of women living with female genital circumcision/mutilation. *Health Care Women Int.,* 37(10), 1156-69.	Data on healthcare-specific experiences is only from health professionals, not women/girls
89.	Olsson E, Lau M, Lifvergren S and Chakhunashvili A. Community collaboration to increase foreign-born women's participation in a cervical cancer screening program in Sweden: A quality improvement project. *International Journal for Equity in Health*. 2014; 13	Data on FGM/C not presented
90.	Pavlish, C.L., Noor, S. & Brandt, J., Somali immigrant women and the American health care system: discordant beliefs, divergent expectations, and silent worries, *Soc Sci Med.* 2010; 71:2, 353-361	Data on FGM/C not presented
91.	Peters K. Reasons why women choose a medical practice or a women's health centre for routine health screening: worker and client perspectives. *J Clin Nurs*. 2010; 19: 2557-64.	Data on FGM/C not presented
92.	Phillimore J. Migrant maternity in an era of superdiversity: new migrants' access to, and experience of, antenatal care in the West Midlands, UK. *Soc Sci Med*. 2016; 148: 152-9.	Data on FGM/C not presented
93.	Quilliam S. Female genital mutilation: three questions for us to consider. *J Fam Plann Reprod Health Care*. 2015; 41: 235-7.	Not empirical research
94.	Radford K. 'Unkind cuts': health policy and practice versus the health and emotional well-being of asylum-	Data on FGM/C not presented

No.	Study	Reason for Exclusion
	seekers and refugees in Ireland. *Journal of Ethnic and Migration Studies*. 2010; 36: 899-915.	
95.	Razzak M. Sexual medicine: Pain and pleasure-reconstruction after female genital mutilation. *Nature Reviews Urology*. 2012; 9.	Not empirical research
96.	Reis E. Culture and cutting. *The Hastings Center Report*. 2012; 42: 3.	Not empirical research
97.	Rimmer SN. [Theme: circumcision. Somali women talk about taboo]. *Sygeplejersken*. 1994; 94: 12-3.	Not empirical research
98.	Roberts TL and Poblete X. Female genital mutilation: A survey of awareness, training and current practice. *Archives of Disease in Childhood: Annual Conference of the Royal College of Paediatrics and Child Health, RCPCH 2015*. Birmingham, UK: BMJ Publishing Group, 2015, p. G55.	Conference abstract
99.	Rogers C and Earnest J. A Cross-Generational Study of Contraception and Reproductive Health Among Sudanese and Eritrean Women in Brisbane, Australia. *Health Care Women Int*. 2014; 35: 334-56.	Data on FGM/C not presented
100.	Rosenberg LB, Gibson K and Shulman JF. When cultures collide: Female genital cutting and U.S. obstetric practice. *Obstet Gynecol*. 2009; 113: 931-4.	Not empirical research
101.	Rosenberg E, Kirmayer LJ, Xenocostas S, Dao MD and Loignon C. GPs' strategies in intercultural clinical encounters. *Fam Pract*. 2007; 24: 145-51.	Data on FGM/C not presented
102.	Roth K. Giving refuge: reflections on working with asylum seekers. *J Pain Symptom Manage*. 2010; 40: 149-54.	Not empirical research
103.	Rubin, E.A. (2000) When Cultures Collide: An Exploration of Cultural Competence and Cross-Cultural Communication between American Medical Providers and Immigrant Women who have been Circumcised. University of Massachusetts, PhD Thesis, USA.	Data on healthcare-specific experiences is only from health professionals, not women/girls
104.	Sala R and Manara D. Nurses and requests for female genital mutilation: cultural rights versus human rights. *Nurs Ethics*. 2001; 8: 247-58.	Not empirical research
105.	Schuster S. 'Gosh': a cross-cultural encounter with a Somali woman, a male interpreter and a gynecologist on female genital cutting/mutilation. *Patient Educ Couns*. 2015; 98: 127-8.	Not empirical research
106.	Shelp A. Women helping women: the Somali doula initiative. *International Journal of Childbirth Education*. 2004; 19: 4-7.	Not qualitative research
107.	Shipp MPL, Francis SA, Fluegge KR and Asfaw SA. Perceived Health Issues: A perspective from East-African immigrants. *Health, Culture and Society*. 2014; 6: 13-32.	Data on FGM/C not presented
108.	Sigurjonsson H, Jordal M and Lundgren TK. Reconstructive surgery after female genital mutilation. *International Journal of Gynecology and Obstetrics*. 2015; 131: E318-E9.	Conference abstract

No.	Study	Reason for Exclusion
109.	Simpson J, Robinson K, Creighton SM and Hodes D. Female genital mutilation: the role of health professionals in prevention, assessment, and management. *Br Med J*. 2012; 344.	Not empirical research
110.	Sokolik L. Somali immigrant and refugee women's experience of pregnancy and childbirth in the American healthcare system. *Commun Nurs Res*. 2007; 40: 538	Conference abstract
111.	Sudbury H and Robinson A. Barriers to sexual and reproductive health care for refugee and asylum-seeking women. *British Journal of Midwifery*. 2016; 24: 275-81.	Not empirical research
112.	Sundby J. Genital mutilation of women--is it a concern for Gynecologists? *Acta Obstet Gynecol Scand*. 1996; 75: 513-5.	Not empirical research
113.	Talle A. From "complete" to "impaired" body: Female circumcision in Somalia and London. *Disability in Local and Global worlds*. Berkeley, CA: University of California Press; US, 2007, p. 56-77.	Not empirical research
114.	Taillens F. ["I will not circumcise my daughter"]. *Krankenpflege - Soins Infirmiers*. 2012; 105: 55.	Not empirical research
115.	Tait P. Refugee Women in Australia and Woman-Centered Midwifery Care. *Nuritinga*. 2013: 31-9.	Not empirical research
116.	Tantet C, Aupiais C, Sorge F, Levy D, Lafon-Desmurs B and Faye A. MIG-01 - Female genital mutilations: Evaluation of the knowledge of general practitioners and physicians in travel counseling. *Med Mal Infect*. 2016; 46: 77.	Not qualitative research
117.	Tatah EF. Female Circumcision: A Phenomenological Study of Somalian Immigrants to the United States. PhD Thesis, Walden University, 2016,	Data presented on FGM/C, but no specific focus on FGM/C related healthcare
118.	Trueland J. School nurses take lead on FGM. *Nurs Stand*. 2014; 28: 22-3.	Not empirical research
119.	Vercoutere A, Temmerman M and Leye E. Female genital mutilation, a story far away from home? Ethical, legal and clinical aspects of mutilated women in western countries. *Tijdschrift voor Geneeskunde*. 2011; 67: 237-41.	Not empirical research
120.	Villani M. From the “maturity” of a woman to surgery: Conditions for clitoris repair. *Sexologies*. 2009; 18: 259-61.	Not qualitative research
121.	Villani M. The way of speaking about "mutilation" and performing "repairing". *J Sex Med*. 2011; 8: 242.	Not qualitative research
122.	Villani M. Experiences with clitoris repair with counselling or counselling only in France, for effect on sexual satisfaction for women with FGM. *Int J Gynecol Obstet*. 2012; 119: S255.	Conference abstract
123.	Villani M, Griffin J and Bodenmann P. In their own words: the health and sexuality of immigrant women with infibulation living in Switzerland. *Soc Sci*. 2016; 5: 1.	Data presented on FGM/C, but no specific focus on FGM/C related healthcare

No.	Study	Reason for Exclusion
124.	Vissandjee B, Denetto S, Migliardi P and Proctor J. Female genital cutting (FGC) and the ethics of care: community engagement and cultural sensitivity at the interface of migration experiences. *BMC Int Health Hum Rights*. 2014; 14: 1-18.	Not empirical research
125.	Vloeberghs E, Knipscheer J, van der Kwaak A, Naleie Z and van den Muijsenbergh M. Veiled Pain: A Study In The Netherlands On The Psychological, Social And Relational Consequences Of Female Genital Mutilation. Pharos–Dutch National Knowledge and Advisory Centre on Refugees and Migrants' Health, 2010.	Duplicate report of included paper
126.	Wade L. The politics of acculturation: Female genital cutting and the challenge of building multicultural democracies. *Soc Probl*. 2011; 58: 518-37.	Data presented on FGM/C, but no specific focus on FGM/C related healthcare
127.	Widmark, C., Leval, A., Tishelman, C. & Ahlberg, B.M. (2010) Obstetric care at the intersection of science and culture: Swedish doctors' perspectives on obstetric care of women who have undergone female genital cutting. *J. Obstet. Gynaecol.,* **30**(6), 553-8.	Study focus is on health professionals, not women/girls
128.	Widmark, C., Tishelman, C. & Ahlberg, B.M. (2002) A study of Swedish midwives' encounters with infibulated African women in Sweden. *Midwifery,* 18(2), 113-25.	Data on healthcare-specific experiences is only from health professionals, not women/girls
129.	Williams KW. Somali Bantu health experience: Refugee resettlement in South Carolina. PhD Thesis, University of South Carolina, 2006	Data on FGM/C not presented
130.	Williams N, Offer GJ, Williams S and Everson N. Parental attitude in children referred for circumcision. *Br J Surg*. 1997; 84: 1722-3.	Data on FGM/C not presented
131.	Willis S. The cutting season. *Journal of Family Health Care*. 2014; 24: 9-12.	Not empirical research
132.	Wimmer-Puchinger B, Wolf H and Engleder A. [Female migrants in the health care system. Health care utilisation, access barriers and health promotion strategies]. *Migrantinnen im Gesundheitssystem Inanspruchnahme, Zugangsbarrieren und Strategien zur Gesundheitsforderung*. 2006; 49: 884-92.	Not empirical research
133.	Young H and McGrath K. A review of circumcision in New Zealand: 'I never liked doing them and I was pleased to give them up'. *Understanding Circumcision: A Multi-Disciplinary Approach To A Multi-Dimensional Problem*. Kluwer Academic Publishers, 2001.	Data on FGM/C not presented
134.	Yusuf L and Negash S. Vaginal calculus following severe form of female genital mutilation: a case report. *Ethiop Med J*. 2008; 46: 185-8.	Not OECD
135.	Zenner N, Liao LM, Richens Y and Creighton SM. Quality of obstetric and midwifery care for pregnant women who have undergone female genital mutilation. *J Obstet Gynaecol*. 2013; 33: 459-62.	Not qualitative research
