# Supplementary material for: Seeking culturally safe care: a qualitative systematic review of the healthcare experiences of women and girls who have undergone female genital mutilation/cutting
Source: BMJ Open. 2019 May 29;9(5):e027452. doi: 10.1136/bmjopen-2018-027452 (PMC6549627; doi:10.1136/bmjopen-2018-027452)
Supplement: Supplementary data [file bmjopen-2018-027452supp004.pdf]

Supplementary File 4: Theme Matrix (Mapping Included Studies to Themes)

		1. Communication is key			2. Access to care: influenced by an interaction of multi-level community and health service processes				3. Cultural and bodily dissonance: striving for cultural and bodily integrity				4. Disempowering care encounters				5. Positive care encounters	
**No.**	**Study Reference**	**1.1**	**1.2**	**1.3**	**2.1**	**2.2**	**2.3**	**2.4**	**3.1**	**3.2**	**3.3**	**3.4**	**4.1**	**4.2**	**4.3**	**4.4**	**5.1**	**5.2**
**1**	Abdi, R. (2012)[1]			X		X				X			X					
**2**	Abdullahi, A., Copping, J., Kessel, A., Luck, M., & Bonell, C. (2009)[2]	X			X		X			X			X					X
**3**	Ahlberg, B. M., Krantz, I., Lindmark, G., & Warsame, M. (2004)[3]	X												X		X		
**4**	Ahmed, M., (2005)[4]		X	X									X					X
**5**	Ameresekere, M., Borg, R., Frederick, J., Vragovic, O., Saia, K., & Raj, A. (2011)[5]		X		X		X							X	X	X	X	X
**6**	Asefaw, F. (2007)[6]		X	X	X			X	X	X	X	X	X	X	X	X	X	X
**7**	Baldeh, F. (2013)[7]		X	X				X	X	X	X		X	X	X	X		X
**8**	Ballesteros Meseguer, C., Almansa Martinez, P., Pastor Bravo, M. d. M., & Jimenez Ruiz, I. (2014)[8]						X			X						X	X	
**9**	Behrendt, A. (2011)[9]		X	X				X	X				X	X	X	X	X	
**10**	Beine, K., Fullerton, J., Palinkas, L., & Anders, B. (1995)[10]		X	X	X						X	X	X		X	X	X	X
**11**	Berggren, V., Bergstrom, S., & Edberg, A.-K. (2006)[11]	X	X	X	X	X	X	X	X	X	X	X	X	X	X	X	X	X
**12**	Betts, V. (2011)[12]	X	X	X	X	X	X	X	X		X	X	X	X	X	X	X	X

		1. Communication is key			2. Access to care: influenced by an interaction of multi-level community and health service processes				3. Cultural and bodily dissonance: striving for cultural and bodily integrity				4. Disempowering care encounters				5. Positive care encounters	
**No.**	**Study Reference**	**1.1**	**1.2**	**1.3**	**2.1**	**2.2**	**2.3**	**2.4**	**3.1**	**3.2**	**3.3**	**3.4**	**4.1**	**4.2**	**4.3**	**4.4**	**5.1**	**5.2**
**13**	Bravo Pastor del Mar, M. (2014)[13]		X				X	X	X		X	X	X	X		X	X	X
**14**	Bulman, K. H., & McCourt, C. (2002)[14]	X		X	X		X	X						X		X	X	X
**15**	Carroll, J., Epstein, R., Fiscella, K., Gipson, T., Volpe, E., & Jean-Pierre, P. (2007)[15]	X		X	X			X						X		X		X
**16**	Chalmers, B., & Omer-Hashi, K. (2002)[16]	X		X	X		X	X	X	X			X	X	X	X	X	X
**17**	Degni, F., Suominen, S. B., El Ansari, W., Vehviläinen-Julkunen, K., & Essen, B. (2014)[17]	X	X		X	X		X	X		X	X		X			X	X
**18**	d'Entremont, M., Smythe, L., & McAra-Couper, J. (2014)[18]	X	X			X				X			X	X	X	X	X	X
**19**	Essén, B., Johnsdotter, S., Hovelius, B., Gudmundsson, S., Sjöberg, N. O., Friedman, J., & Östergren, P. O. (2000)[19]						X	X							X	X	X	
**20**	Fawcett, L., (2014)[20]	X	X	X	X	X		X	X	X			X	X	X	X		X
**21**	Gali, M. A. (1997)[21]	X		X			X	X	X	X	X	X	X	X	X	X	X	X
**22**	Ghebre, R. G., Sewali, B., Osman, S., Adawe, A., Nguyen, H. T., Okuyemi, K. S., & Joseph, A. (2015)[22]	X			X	X	X			X								
**23**	Glazer, E., (2012)[23]		X	X	X			X	X	X	X		X		X	X	X	X
**24**	Glover, J., Liebling, H., Barrett, H., & Goodman, S. (2017)[24]			X			X	X	X	X	X	X	X	X	X	X	X	X

		1. Communication is key			2. Access to care: influenced by an interaction of multi-level community and health service processes				3. Cultural and bodily dissonance: striving for cultural and bodily integrity				4. Disempowering care encounters				5. Positive care encounters	
**No.**	**Study Reference**	**1.1**	**1.2**	**1.3**	**2.1**	**2.2**	**2.3**	**2.4**	**3.1**	**3.2**	**3.3**	**3.4**	**4.1**	**4.2**	**4.3**	**4.4**	**5.1**	**5.2**
**25**	Guerin, P. B., Allotey, P., Elmi, F. H., & Baho, S. (2006)[25]			X			X		X		X	X	X	X	X	X		X
**26**	Hill, N., Hunt, E., & Hyrkas, K. (2012)[26]	X			X	X	X	X	X		X				X	X	X	X
**27**	Hussein, E., (2010)[27]	X	X	X	X			X	X	X			X	X		X		X
**28**	Hussen, M. A. (2014)[28]	X	X	X	X		X	X	X	X	X		X			X	X	X
**29**	Johansen, R.E. (2017)[29]				X				X		X	X						X
**30**	Jones, A. (2010)[30]		X		X	X	X		X		X			X		X	X	X
**31**	Khaja, K. (2004)[31]		X	X	X	X	X				X				X	X		
**32**	Khaja, K., Lay, K., & Boys, S. (2010)[32]								X				X	X				X
**33**	Lundberg, P. C., & Gerezgiher, A. (2008)[33]		X		X	X		X	X	X	X	X			X	X	X	X
**34**	Maier, C. (2003)[34]				X	X	X	X	X	X	X	X	X	X	X	X	X	X
**35**	Maternity Action. (2014)[35]	X	X	X	X			X	X				X	X	X	X		X
**36**	McNeely, S., & Christie-de Jong, F. (2016)[36]		X					X	X	X			X			X		X
**37**	Moxey, J. M., & Jones, L. L. (2016)[37]	X	X		X		X	X		X	X		X		X	X	X	X
**38**	Murray, L., Windsor, C., Parker, E., & Tewfik, O. (2010)[38]	X	X	X	X		X	X	X				X	X	X	X	X	

		1. Communication is key			2. Access to care: influenced by an interaction of multi-level community and health service processes				3. Cultural and bodily dissonance: striving for cultural and bodily integrity				4. Disempowering care encounters				5. Positive care encounters	
**No.**	**Study Reference**	**1.1**	**1.2**	**1.3**	**2.1**	**2.2**	**2.3**	**2.4**	**3.1**	**3.2**	**3.3**	**3.4**	**4.1**	**4.2**	**4.3**	**4.4**	**5.1**	**5.2**
**39**	Norman, K., Gegzabher, S. B., & Otoo-Oyortey, N. (2016)[39]		X	X			X	X					X	X				X
**40**	Norman, K., Hemmings, J., Hussein, E., & Otoo-Oyortey, N. (2009)[40]	X	X	X	X		X	X	X	X	X		X	X	X	X	X	X
**41**	O'Brien, O., Baldeh, F., Hassan, J., & Baillie, M. (2017)[41]	X	X				X	X	X	X	X	X	X		X	X	X	X
**42**	O'Brien, O., Baldeh, F., Sivapatham, S., Brown, E., & O'May, F. (2016)[42]	X	X	X			X	X	X				X			X	X	X
**43**	Palfreyman, A., Brown, E., & Nam, S. (2011)[43]	X	X	X			X	X	X		X	X	X	X		X	X	X
**44**	Recchia, N., & McGarry, J. (2017)[44]		X	X									X	X		X		X
**45**	Safari, F., (2013)[45]		X		X	X			X		X	X						
**46**	Salad, J., Verdonk, P., de Boer, F., & Abma, T. A. (2015)[46]	X	X		X	X	X		X	X			X			X		
**47**	Shaw, E. (1985)[47]						X	X	X	X	X	X				X		X
**48**	Shermarke, M.A.A. (1996)[48]		X					X	X					X	X	X	X	X
**49**	Straus, L., McEwen, A., & Hussein, F. M. (2009)[49]	X		X	X	X	X	X	X		X		X	X	X	X		X
**50**	Thierfelder, C. (2003)[50]			X								X						
**51**	Thierfelder, C., Tanner, M., & Bodiang, C. M. K. (2005)[51]	X	X		X		X	X	X	X	X		X	X	X	X		X

		1. Communication is key			2. Access to care: influenced by an interaction of multi-level community and health service processes				3. Cultural and bodily dissonance: striving for cultural and bodily integrity				4. Disempowering care encounters				5. Positive care encounters	
**No.**	**Study Reference**	**1.1**	**1.2**	**1.3**	**2.1**	**2.2**	**2.3**	**2.4**	**3.1**	**3.2**	**3.3**	**3.4**	**4.1**	**4.2**	**4.3**	**4.4**	**5.1**	**5.2**
**52**	Upvall, M. J., Mohammed, K., & Dodge, P. D. (2009)[52]	X	X	X	X	X	X		X				X	X		X	X	X
**53**	Vangen, S., Johansen, R. E. B., Sundby, J., Traeen, B., & Stray-Pedersen, B. (2004)[53]	X	X	X	X	X	X	X	X	X			X	X	X	X	X	
**54**	Vaughan, C., White, N., Keogh, L., Tobin, J., Ha, B., Ibrahim, M., & Bayly, C. (2014)[54]	X	X	X	X	X	X	X	X	X	X	X	X	X	X	X	X	X
**55**	Vaughan, C., White, N., Keogh, L., Tobin, J., Murdolo, A., Quiazon, R., & Bayly, C. (2014)[55]	X	X	X	X		X	X	X	X	X	X	X	X	X	X	X	X
**56**	Vloeberghs, E., van der Kwaak, A., Knipscheer, J., & van den Muijsenbergh, M. (2012)[56]	X	X	X	X			X	X	X			X	X	X		X	X
**57**	Wiklund, H., Aden, A. S., Högberg, U., Wikman, M., & Dahlgren, L. (2000)[57]	X		X	X	X	X	X	X				X	X	X	X	X	X
	**Total Studies per Theme**	**31**	**37**	**34**	**35**	**19**	**34**	**38**	**40**	**28**	**28**	**19**	**40**	**36**	**32**	**46**	**34**	**45**

Theme Key – for Theme Matrix

Descriptive Theme No.	Theme Name
**Analytical Theme 1: Communication is key**	
**1.1**	Language barriers and interpretation challenges
**1.2**	"Can't talk, not asked": double silence and cultural taboo
**1.3**	Cultural (in)sensitivity
**Analytical Theme 2: Access to care: influenced by an interaction of multi-level community and health service processes**	
**2.1**	Influence of cultural norms
**2.2**	Influence of the family
**2.3**	Knowledge and information about FGM/C services
**2.4**	'Hit and miss' care
**Analytical Theme 3: Cultural and bodily dissonance: striving for cultural and bodily integrity**	
**3.1**	Moving from normal to different
**3.2**	Threat to the self: re-living FGM/C pain during clinical interventions
**3.3**	Being opened: complexities around deinfibulation
**3.4**	Being changed: complexities around re-infibulation
**Analytical Theme 4: Disempowering care encounters**	
**4.1**	Being exposed and humiliated
**4.2**	Being judged and stereotyped
**4.3**	Lacking choice, power and control
**4.4**	Feeling unsafe and vulnerable
**Analytical Theme 5: Positive care encounters**	
**5.1**	Trusting and appreciating providers and the system
**5.2**	Voicing healthcare needs and preferences

References of Included Studies - Cited in Theme Matrix

1. Abdi R. Carving culture: Creating identity through female genital cutting. *Durham Anthropology Journal* 2012;18(1):115-53.

2. Abdullahi A, Copping J, Kessel A, et al. Cervical screening: perceptions and barriers to uptake among Somali women in Camden. *Public Health* 2009;123(10):680-5. doi: 10.1016/j.puhe.2009.09.011

3. Ahlberg BM, Krantz I, Lindmark G, et al. 'It's only a tradition': making sense of eradication interventions and the persistence of female 'circumcision' within a Swedish context. *Crit Soc Policy* 2004;24(1):50-78. doi: 10.1107/0261018304039679

4. Ahmed M. Attitudes towards FGM among Somali women living in the UK. In: Momoh C, ed. Female Genital Mutilation. London: Radcliffe 2005:95-117.

5. Ameresekere M, Borg R, Frederick J, et al. Somali immigrant women's perceptions of cesarean delivery and patient-provider communication surrounding female circumcision and childbirth in the USA. *Int J Gynecol Obstet* 2011;115(3):227-30. doi: 10.1016/j.ijgo.2011.07.019

6. Asefaw F. [Female Genital Mutilation: A Field Study with Special Consideration of the Background as well as the Health and Psychosexual Consquences for those Affected and their Partners in Eritrea and in Germany] Weibliche Genitalbeschneidung: Eine Feldstudie Unter besonderer Berücksichtigung der Hintergründe Sowie der Gesundheitlichen und Psychosexuellen Folgen für Betroffene und Partner in Eritrea und Deutschland. Humboldt University of Berlin, 2007.

7. Baldeh F. Obstetric Care in Scotland: The Experience of Women who have Undergone Female Genital Mutilation (FGM) [MSc Thesis]. Queen Margaret University, 2013.

8. Ballesteros Meseguer C, Almansa Martinez P, Pastor Bravo MdM, et al. [The voice of women subjected to female genital mutilation in the region of Murcia, Spain] La Voz de las mujeres sometidas a mutilacion genital femenina en la region de Murcia. *Gac Sanit* 2014;28(4):287-91. doi: 10.1016/j.gaceta.2014.02.006

9. Behrendt A. Listening to African Voices: Female Genital Mutilation/Cutting among Immigrants in Hamburg: Knowledge, Attitudes and Practice. Hamburg, Germany: Plan, 2011.

10. Beine K, Fullerton J, Palinkas L, et al. Conceptions of prenatal care among Somali women in San Diego. *J Nurs Midwifery* 1995;40(4):376-81. doi: Doi 10.1016/0091-2182(95)00024-E

11. Berggren V, Bergstrom S, Edberg A-K. Being different and vulnerable: experiences of immigrant African women who have been circumcised and sought maternity care in Sweden. *J Transcult Nurs* 2006;17(1):50-7. doi: 10.1177/1043659605281981

12. Betts V. Transnational Gender, Sexuality and Identity Construction: Ambivalent Subjectivities of Somali Refugee Women in the GTA. MA Thesis, The University of Guelph, 2011.

13. Bravo Pastor del Mar M. [Women Subjected to Female Genital Mutilation: Knowledge for the Nursing Discipline] La Voz de las Mujeres Sometidas a Mutilación Genital Femenina: Saberes Para la Disciplina Enfermera [Tesis Doctoral]. University of Murcia, 2014.

14. Bulman KH, McCourt C. Somali refugee women's experiences of maternity care in west London: a case study. *Crit Public Health* 2002;12(4):365-80.

15. Carroll J, Epstein R, Fiscella K, et al. Caring for Somali women: implications for clinician-patient communication. *Patient Educ Couns* 2007;66(3):337-45. doi: 10.1016/j.pec.2007.01.008

16. Chalmers B, Omer-Hashi K. What Somali women say about giving birth in Canada. *J Reprod Infant Psychol* 2002;20(4):267-82. doi: 10.1080/0264683021000033183

17. Degni F, Suominen SB, El Ansari W, et al. Reproductive and maternity health care services in Finland: perceptions and experiences of Somali-born immigrant women. *Ethn Health* 2014;19(3):348-66. doi: 10.1080/13557858.2013.797567

18. d'Entremont M, Smythe L, McAra-Couper J. The sounds of silence: a hermeneutic interpretation of childbirth post excision. *Health Care Women Int* 2014;35(3):300-19. doi: 10.1080/07399332.2013.838245

19. Essén B, Johnsdotter S, Hovelius B, et al. Qualitative study of pregnancy and childbirth experiences in Somalian women resident in Sweden. *Br J Obstet Gynaecol* 2000;107(12):1507-12.

20. Fawcett L. Somali Refugee Women and their U.S. Healthcare Providers: Knowledge, Perceptions and Experiences of Childbearing [Doctor of Philosophy]. Arizona State University, 2014.

21. Gali MA. Female Circumcision: A Transcultural Study of Attitudes, Identity and Reproductive Health of East African Immigrants [Degree of Doctor of Philosophy in Psychology]. The Wright Institute, University of Berkeley, 1997.

22. Ghebre RG, Sewali B, Osman S, et al. Cervical cancer: barriers to screening in the Somali community in Minnesota. *J Immigr Minor Health* 2015;17(3):722-28. doi: 10.1007/s10903-014-0080-1

23. Glazer E. Embodiment, Pain and Circumcision in Somali-Canadian Women [M.Sc.]. University of Toronto, 2012.

24. Glover J, Liebling H, Barrett H, et al. The psychological and social impact of female genital mutilation: a holistic conceptual framework. *J Int Stud* 2017;10(2):219-38.

25. Guerin PB, Allotey P, Elmi FH, et al. Advocacy as a means to an end: assisting refugee women to take control of their reproductive health needs. *Women Health* 2006;43(4):7-25.

26. Hill N, Hunt E, Hyrkas K. Somali immigrant women's health care experiences and beliefs regarding pregnancy and birth in the United States. *J Transcult Nurs* 2012;23(1):72-81. doi: 10.1177/1043659611423828 [published Online First: 2011/11/05]

27. Hussein E. Women's Experiences, Perceptions and Attitudes of Female Genital Mutilation: The Bristol PEER Study. London: FORWARD, 2010.

28. Hussen MA. Services for Women with Female Genital Mutilation in Christchurch: Perspectives of Women and their Health Providers. University of Canterbury, 2014.

29. Johansen RE. Undoing female genital cutting: perceptions and experiences of infibulation, defibulation and virginity among Somali and Sudanese migrants in Norway. *Cult Health Sex* 2017;19(4):528-42. doi: 10.1080/13691058.2016.1239838

30. Jones A. Working Psychologically with Female Genital Mutilation: An Exploration of the Views and Experiences of Women who have Experienced FGM and of Clinical Psychologists. University of East London, 2010.

31. Khaja K. Female Circumcision: Life Histories of Somali Women [Doctor of Philosophy]. The University of Utah, 2004.

32. Khaja K, Lay K, Boys S. Female circumcision: toward an inclusive practice of care. *Health Care Women Int* 2010;31(8):686-99. doi: 10.1080/07399332.2010.490313

33. Lundberg PC, Gerezgiher A. Experiences from pregnancy and childbirth related to female genital mutilation among Eritrean immigrant women in Sweden. *Midwifery* 2008;24(2):214-25. doi: 10.1016/j.midw.2006.10.003

34. Maier C. [Echoes of Silence: Voices of Concern for Genital Mutilation in African Immigrant Women in Vienna: An Ethnological Study] Echo des Schweigens: Stimmen der Betroffenheit zur Genitalverstümmelung bei Afrikanischen Immigrantinnen in Wien: Ethnologische Studie. University of Vienna, 2003.

35. Maternity Action. Women's Voices on Health: Addressing Barriers to Accessing Primary Care. London: Women’s Health and Equality Consortium, 2014.

36. McNeely S, Christie-de Jong F. Somali refugees' perspectives regarding FGM/C in the US. *Int J Migr Health Soc Care* 2016;12(3):157-69. doi: 10.1108/Ijmhsc-09-2015-0033

37. Moxey JM, Jones LL. A qualitative study exploring how Somali women exposed to female genital mutilation experience and perceive antenatal and intrapartum care in England. *BMJ Open* 2016;6(1):e009846. doi: 10.1136/bmjopen-2015-009846

38. Murray L, Windsor C, Parker E, et al. The experiences of African women giving birth in Brisbane, Australia. *Health Care Women Int* 2010;31(5):458-72. doi: 10.1080/07399330903548928

39. Norman K, Gegzabher SB, Otoo-Oyortey N. "Between Two Cultures": A Rapid PEER Study Exploring Migrant Communities' Views on Female Genital Mutilation in Essex and Norfolk, UK. FORWARD & National FGM Centre Report, , 2016.

40. Norman K, Hemmings J, Hussein E, et al. "FGM is Always With Us": Experiences, Perceptions and Beliefs of Women Affected by Female Genital Mutilation in London: Results from a PEER Study. London: Options Consultancy Services and FORWARD, 2009.

41. O'Brien O, Baldeh F, Hassan J, et al. My Voice: Participatory Action Research Project with Men, Women and Young People on Female Genital Mutilation (FGM) in Scotland: (Phase 2). Waverly Care, Edinburgh: Queen Margaret University, 2017.

42. O'Brien O, Baldeh F, Sivapatham S, et al. Participatory Action Research Project with Men, Women and Young People on Female Genital Mutilation (FGM) in Scotland: (Phase 1). Waverly Care, Edinburgh: Queen Margaret University, 2016.

43. Palfreyman A, Brown E, Nam S. Understanding Female Genital Mutilation in Birmingham: Findings from a PEER Study: Options Consultancy Services and Birmingham & Solihull Women's Aid, 2011.

44. Recchia N, McGarry J. "Don't judge me": narratives of living with FGM. *Int J Hum Rights Healthc* 2017;10(1):4-13. doi: 10.1108/Ijhrh-10-2016-0016

45. Safari F. A qualitative study of women's lived experience after deinfibulation in the UK. *Midwifery* 2013;29(2):154-8. doi: 10.1016/j.midw.2011.12.005

46. Salad J, Verdonk P, de Boer F, et al. "A Somali girl is Muslim and does not have premarital sex - is vaccination really necessary?" A qualitative study into the perceptions of Somali women in the Netherlands about the prevention of cervical cancer. *Int J Equity Health* 2015;14(1):1-13. doi: 10.1186/s12939-015-0198-3

47. Shaw E. Female circumcision: perceptions of clients and caregivers. *J Am Coll Health* 1985;33(5):193-7. doi: 10.1080/07448481.1985.9939604

48. Shermarke MAA. Understanding the Canadian Community Context of Female Circumcision [M.S.W.]. McGill University, 1996.

49. Straus L, McEwen A, Hussein FM. Somali women's experience of childbirth in the UK: perspectives from Somali health workers. *Midwifery* 2009;25(2):181-6. doi: 10.1016/j.midw.2007.02.002

50. Thierfelder C. Female Genital Mutilation and the Swiss Health Care System. University of Basel, 2003.

51. Thierfelder C, Tanner M, Bodiang CMK. Female genital mutilation in the context of migration: experience of African women with the Swiss health care system. *Eur J Public Health* 2005;15(1):86-90. doi: 10.1093/eurpub/cki120

52. Upvall MJ, Mohammed K, Dodge PD. Perspectives of Somali Bantu refugee women living with circumcision in the United States: a focus group approach. *Int J Nurs Stud* 2009;46(3):360-8. doi: 10.1016/j.ijnurstu.2008.04.009

53. Vangen S, Johansen REB, Sundby J, et al. Qualitative study of perinatal care experiences among Somali women and local health care professionals in Norway. *Eur J Obstet Gynecol Reprod Biol* 2004;112(1):29-35. doi: 10.1016/S0301-2115(03)00313-0

54. Vaughan C, White N, Keogh L, et al. Listening to North Yarra Communities about Female Genital Cutting. Melbourne, Australia: The University of Melbourne, 2014.

55. Vaughan C, White N, Keogh L, et al. Female Genital Mutilation/Cutting in Regional Victoria. Research to Practice. Melbourne, Australia: The University of Melbourne, 2014.

56. Vloeberghs E, van der Kwaak A, Knipscheer J, et al. Coping and chronic psychosocial consequences of female genital mutilation in the Netherlands. *Ethn Health* 2012;17(6):677-95. doi: 10.1080/13557858.2013.771148

57. Wiklund H, Aden AS, Högberg U, et al. Somalis giving birth in Sweden: a challenge to culture and gender specific values and behaviours. *Midwifery* 2000;16(2):105-15. doi: http://dx.doi.org/10.1054/midw.1999.0197
