# Supplementary material for: Seeking culturally safe care: a qualitative systematic review of the healthcare experiences of women and girls who have undergone female genital mutilation/cutting
Source: BMJ Open. 2019 May 29;9(5):e027452. doi: 10.1136/bmjopen-2018-027452 (PMC6549627; doi:10.1136/bmjopen-2018-027452)
Supplement: Supplementary data [file bmjopen-2018-027452supp003.pdf]

Supplementary File 3: Coding and Analysis Process

Example of the Analytical Theme of 'Communication is Key'

Below, we describe the analytical process involved in the thematic synthesis, using the Analytical Theme 1 ('Communication is Key') as an exemplar.

A group of rich, relevant and high quality papers was initially coded line by line.[1-13] This approach used 'open coding' (i.e. it was data-driven - there were no pre-defined codes or themes). Two reviewers (RT &CE) undertook this task independently.

The open codes were then reviewed and compared. Where reviewers had identified and coded the same issue, a code for that issue was agreed for use in coding subsequent papers. A standardised name was identified for the code and a description was produced. Where reviewers had applied slightly different codes to a concept indicating a different interpretation of meaning, they discussed it and either agreed a shared code (based on a shared understanding of meaning) or created two different codes to reflect two different potential meanings.

**Example: Straus 2009**[9]<br>*"Lack of interpreters and notably a lack of what was described as ''suitable'' ones were mentioned as a difficulty". P.183*<br><br>Reviewer 1(CE) open code – interpretation problems<br>Reviewer 2 (RT) open code – lack of interpreters<br><br>Initial combined open code 1: Interpretation problems<br>Description: Challenges with using interpreters<br><br>Initial combined open code 2: Lack of interpreters<br>Description: Lack of access to interpreters

As can be seen above, the initial set of open codes were 'free nodes'. These initial sets of codes were regularly examined through team discussion. The codes were then expanded, merged or refined. For example, after coding additional papers, it became clear that the initial combined open code 1 ('interpretation problems') was very broad, so it was decided to code more closely to identify what specific aspects of interpretation were considered challenging. After re-reading the original papers, two refined codes were created, so that there were a total of three codes related to interpretation (all the index papers were re-coded using the refined framework).

Open code 1: Unskilled interpreters<br>Description: When interpreters are untrained or unable to translate the specific terminology around FGM/C<br><br>Open code 2: Male interpreters<br>Description: Cultural sensitivities and challenges with communication from having a male interpreter<br><br>Open code 3: Lack of interpreters

Description: This refers to a lack of access to interpreters or service and reliance on self. Health services not offering interpreting services and women have to find their own to access care.

As the coding progressed, the texts coded to each node were reviewed by the team and the open codes began to be grouped into themes based on similarity in meaning. For example, the three open codes above were grouped under one node entitled 'Interpretation Challenges'.

Theme (Node): Interpretation Challenges<br>Description: Ways in which challenges with interpretation affect communication about FGM/C

The team met regularly to review the codes/nodes and their descriptions (the codebook) and, through discussion, began a process of comparing and refining themes and building a thematic framework. For example, the findings from the papers coded under 'Interpretation Challenges' were compared with findings that had been coded under another theme entitled 'Language Barriers'. This process showed that 'Interpretation Challenges' were one of several consequences of 'Language Barriers', all of which affected health professional and patient communication. Hence, the theme 'Interpretation Challenges' was merged with the theme 'Language Barriers', to create a new descriptive theme called: 'Language Barriers and Interpretation Challenges'.

Further analysis made clear that whilst 'Language Barriers and Interpretation Challenges' were an important aspect of communication problems around FGM/C, there were also other challenges to communication that had been identified and coded under other descriptive themes - around cultural taboos ('Can't talk, not asked') and experiencing culturally insensitive questions ('Cultural In-Sensitivity'). Hence, in the final analysis, the meanings of the three descriptive themes, were interpreted into one analytical theme ('Communication is Key').

References

1. Baldeh F. Obstetric Care in Scotland: The Experience of Women who have Undergone Female Genital Mutilation (FGM) [MSc Thesis]. Queen Margaret University, 2013.
2. d'Entremont M, Smythe L, McAra-Couper J. The sounds of silence: a hermeneutic interpretation of childbirth post excision. *Health Care Women Int* 2014;35(3):300-19. doi: 10.1080/07399332.2013.838245
3. Fawcett L. Somali Refugee Women and their U.S. Healthcare Providers: Knowledge, Perceptions and Experiences of Childbearing [Doctor of Philosophy]. Arizona State University, 2014.
4. Glover J, Liebling H, Barrett H, et al. The psychological and social impact of female genital mutilation: a holistic conceptual framework. *J Int Stud* 2017;10(2):219-38.
5. Jones A. Working Psychologically with Female Genital Mutilation: An Exploration of the Views and Experiences of Women who have Experienced FGM and of Clinical Psychologists. University of East London, 2010.
6. Lundberg PC, Gerezgiher A. Experiences from pregnancy and childbirth related to female genital mutilation among Eritrean immigrant women in Sweden. *Midwifery* 2008;24(2):214-25. doi: 10.1016/j.midw.2006.10.003

7. Moxey JM, Jones LL. A qualitative study exploring how Somali women exposed to female genital mutilation experience and perceive antenatal and intrapartum care in England. *BMJ Open* 2016;6(1):e009846. doi: 10.1136/bmjopen-2015-009846
8. Palfreyman A, Brown E, Nam S. Understanding Female Genital Mutilation in Birmingham: Findings from a PEER Study: Options Consultancy Services and Birmingham & Solihull Women's Aid, 2011.
9. Straus L, McEwen A, Hussein FM. Somali women's experience of childbirth in the UK: perspectives from Somali health workers. *Midwifery* 2009;25(2):181-6. doi: 10.1016/j.midw.2007.02.002
10. Thierfelder C. Female Genital Mutilation and the Swiss Health Care System. University of Basel, 2003.
11. Vangen S, Johansen REB, Sundby J, et al. Qualitative study of perinatal care experiences among Somali women and local health care professionals in Norway. *Eur J Obstet Gynecol Reprod Biol* 2004;112(1):29-35. doi: 10.1016/S0301-2115(03)00313-0
12. Vaughan C, White N, Keogh L, et al. Listening to North Yarra Communities about Female Genital Cutting. Melbourne, Australia: The University of Melbourne, 2014.
13. Vaughan C, White N, Keogh L, et al. Female Genital Mutilation/Cutting in Regional Victoria. Research to Practice. Melbourne, Australia: The University of Melbourne, 2014.
