# Supplementary material for: Seeking culturally safe care: a qualitative systematic review of the healthcare experiences of women and girls who have undergone female genital mutilation/cutting
Source: BMJ Open. 2019 May 29;9(5):e027452. doi: 10.1136/bmjopen-2018-027452 (PMC6549627; doi:10.1136/bmjopen-2018-027452)
Supplement: Author's manuscript [file bmjopen-2018-027452.draft_revisions.pdf]

BMJ Open

BMJ Open is committed to open peer review. As part of this commitment we make the peer review history of every article we publish publicly available.

When an article is published we post the peer reviewers' comments and the authors' responses online. We also post the versions of the paper that were used during peer review. These are the versions that the peer review comments apply to.

The versions of the paper that follow are the versions that were submitted during the peer review process. They are not the versions of record or the final published versions. They should not be cited or distributed as the published version of this manuscript.

BMJ Open is an open access journal and the full, final, typeset and author-corrected version of record of the manuscript is available on our site with no access controls, subscription charges or pay-per-view fees (http://bmjopen.bmj.com).

If you have any questions on BMJ Open's open peer review process please email info.bmjopen@bmj.com

BMJ Open

Seeking Culturally Safe Care: A Qualitative Systematic Review of the Healthcare Experiences of Women and Girls who have Undergone Female Genital Mutilation/Cutting

Journal:	*BMJ Open*
Manuscript ID	bmjopen-2018-027452
Article Type:	Research
Date Submitted by the Author:	23-Oct-2018

Complete List of Authors:	Evans, Catrin; University of Nottingham School of Health Sciences, School of Health Sciences<br>Tweheyo, Ritah; University of Nottingham School of Health Sciences, School of Health Sciences<br>McGarry, Julie; University of Nottingham,<br>Eldridge, Jeanette; University of Nottingham School of Health Sciences, Research and Learning Services,<br>Albert, Juliet; Imperial College Healthcare NHS Trust, Department of Midwifery<br>Nkoyo, Valentine; Mojatu Foundation<br>Higginbottom, Gina; University of Nottingham School of Health Sciences

Keywords:	Female genital mutilation, Female genital cutting, CERQual, Deinfibulation, Qualitative systematic review, Thematic synthesis

SCHOLARONE™
Manuscripts

Seeking Culturally Safe Care: A Qualitative Systematic Review of the Healthcare Experiences of Women and Girls who have Undergone Female Genital Mutilation/Cutting

Evans C,[1*] Tweheyo R,[1] McGarry J,[1] Eldridge J,[1] Albert J,[2] Nkoyo V,[3] Higginbottom G,[1]

[1] School of Health Sciences, University of Nottingham, Nottingham, UK.

[2] Department of Maternity and Obstetrics, Imperial College Healthcare NHS Trust, London, UK.

[3] Mojatu Foundation, Nottingham, UK.

***Corresponding author contact details**

Dr. Catrin Evans, B Floor, School of Health Sciences, University of Nottingham, Queens Medical Centre, Derby Road, Nottingham, NG7 2HA, UK. Tel. 0115 823 0894, Email: Catrin.Evans@nottingham.ac.uk

Word count: 8,241

For peer review only

Seeking Culturally Safe Care: A Qualitative Systematic Review of the Healthcare Experiences of Women and Girls who have Undergone Female Genital Mutilation/Cutting

ABSTRACT

OBJECTIVE

To explore the experiences of accessing and receiving healthcare related to female genital mutilation/cutting (FGM/C) across the life course, from the perspective of women and girls who have undergone FGM/C.

DESIGN

A systematic review of qualitative research studies, using a thematic synthesis approach.

METHODS

Inclusion criteria were qualitative studies (including grey literature) of any design, from OECD countries, of any date and any language. Sixteen electronic databases were searched from inception to December 2017, supplemented by reference list searching. Papers were screened, selected and quality appraised by two reviewers using established tools from the Joanna Briggs Institute (JBI). NVivo software was utilised to extract study characteristics and code study findings. An inductive thematic synthesis approach was undertaken to identify descriptive themes and interpret these into higher order analytical constructs. Confidence in the review findings was assessed using GRADE-CERQual.

RESULTS

Fifty seven papers (from 55 distinct studies) from 14 different OECD countries were included (50% published within the last eight years). One third of studies focused exclusively on maternity care experiences, whereas others covered a range of foci. No studies reported explicitly on girls' experiences or on experiences of health-service led safeguarding interventions. Only three studies addressed psychological care. The synthesis developed 17 descriptive themes, organised into five analytical constructs. These related to: communication; access to care; experiences of cultural dissonance/integrity; disempowering care experiences and positive care encounters. The themes illuminate significant challenges to obtaining timely and holistic care (especially for deinfibulation), and highlight different ways in which women may experience care as disrespectful, unsafe and disempowering. Key elements of 'culturally safe care' are identified.

CONCLUSIONS

This review has highlighted key knowledge gaps, especially around (i) girls'/unmarried women's experiences, and, (ii) the impact of recent safeguarding interventions. There is an ongoing need for community engagement, service development and staff training.

For peer review only

PROSPERO REGISTRATION NO: CRD420150300012015

STRENGTHS AND LIMITATIONS OF THIS STUDY

- This is the first review that goes beyond a maternity care focus to examine women's/girls' experiences of FGM/C-related healthcare across the life course, enabling new insights into care-seeking and care-access, especially around deinfibulation.
- This is an exceptionally comprehensive review, due to its wide focus and its inclusion of grey literature and papers in any language.
- The review has used a theoretical model of 'cultural safety' to inform its analytical approach and has assessed confidence in its findings using GRADE-CERQual.
- The review process has been informed by strong community involvement and input from a multi-disciplinary expert advisory group at every stage.
- Methodological limitations of the included studies make it difficult to develop findings that reflect nuances of experience related to ethnic group, nationality, age or type of FGM/C.

KEYWORDS

Female genital mutilation, female genital cutting, deinfibulation, qualitative systematic review, thematic synthesis, CERQual

For peer review only

INTRODUCTION

Health services in higher income countries are said to be operating in a context of 'super-diversity',[1-5] Whereas in previous decades, in-migration was primarily from a limited number of countries traditionally linked through former colonial ties, contemporary migration involves groups from many diverse countries across the globe. Super-diversity poses significant challenges to host country health services, in terms of adapting services and providing culturally appropriate and accessible care to very many different migrant groups with widely different migration histories, languages, health needs and social vulnerabilities.

Female genital mutilation (or female genital cutting) – hereafter referred to as FGM/C - is practised in 30 countries across North and sub-Saharan Africa and in parts of the Middle East and Asia.[6] In an era of super-diverse migration, European and other high-income countries (e.g. USA, Canada, Australia and New Zealand) are becoming home to increasing numbers of women and girls who have experienced, or who may be at risk of, FGM/C (within Europe alone there are thought to be over half a million women and girls who are FGM/C survivors).[7] These women and girls come from highly diverse regions with different cultural traditions, contexts and beliefs associated with the practise of FGM/C, and where different types of FGM/C are practised. For example, in some communities (e.g. within Somalia, Somaliland, Sudan, Eritrea, Kenya) type 3 FGM/C is common involving infibulation (narrowing of the vaginal opening through the creation of a covering seal, formed by cutting and repositioning the labia minora, or labia majora, sometimes through stitching, with or without removal of the clitoris). In other communities however, type 1 (partial or total clitoridectomy) or type 2 (partial or total clitoridectomy and removal of the labia minora, with or without excision of the labia majora) are more common.[8] However, it should be noted that these typologies represent biomedical categories and that different communities have their own nomenclature. Moreover, the exact type of FGM/C a woman has experienced may depend to some extent on the skills and experience of the 'cutter' and women themselves may not always be aware of which type of FGM/C they have experienced.

FGM/C can involve immediate and long term psychological, sexual, relational and physical health sequelae.[9] Health problems may be particular severe for women with Type 3 FGM/C who

require a degree of deinfibulation in order to have sexual intercourse and to give birth. WHO defines deinfibulation as "*the practice of cutting open the sealed vaginal opening in a woman who has been infibulated, which is often necessary for improving health and well-being as well as to allow intercourse or to facilitate childbirth*".[8] Currently, deinfibulation is recommended for women and girls reporting medical or psychosexual symptoms related to type 3 FGM/C or upon request (i.e. personal choice). In addition, global guidelines specifically recommend that deinfibulation is undertaken to prevent obstetric complications, although the optimal timing for the procedure (antepartum or intrapartum) is unclear.[9-12] [9,13-17] There are compelling clinical reasons however for preferring antepartum deinfibulation, including the fact that it can be performed under local anaesthetic in an out-patient setting, thus reducing costs and risks associated with any emergency procedures that may emerge during labour.[18] In addition, in destination countries where staff may be less familiar with type 3 FGM/C, planned antepartum or pre-marital deinfibulation ensures that it is performed by trained and experienced professionals.[15]

In destination countries, it is essential, therefore, that care pathways are developed that are able to support women's potential psychological and sexual health needs,[9-11,19,20] as well as supporting decision making around deinfibulation. In addition, health professionals are called upon to play a key role in the prevention of FGM/C, through health education with affected communities and identification of girls who may be at risk.[20-22] However, as a cultural phenomenon associated with women's sexuality, gender norms and genital area, FGM/C is a sensitive topic that can be hard for women, communities and health professionals to openly discuss.[23-25] Recent studies of health sector involvement in the management of FGM/C in destination countries, show variable availability of specialist services and staff training.[21,26] Moreover, several systematic reviews indicate that health professionals may lack knowledge, confidence and competence in managing FGM/C.[27-29] In addition, there are on-going calls for greater community involvement in the development of appropriate services related to FGM/C.[30-32]

Aim

This paper reports the findings of a qualitative systematic review that aimed to explore the experiences of accessing and receiving FGM/C-related healthcare across the life course for

For peer review only

women and girls who have undergone FGM/C.[33,34] The review was conceived from an ongoing partnership between an academic team (CE, RT, GH, JMc, JE), clinical experts (JA) and a community organisation working on FGM/C-related issues (VN). By undertaking the review we hoped to illuminate women's voices and experiences in order to inform the provision of FGM/C related healthcare.

Rationale

Several prior reviews have been undertaken in relation to this topic, however we felt a new review was warranted for several reasons. First, most existing reviews have focused upon women's maternity care experiences or maternity-focused interventions.[35-39] However, as indicated above, a key issue for services is the development of joined up care pathways that are able to offer women a range of holistic services at different time points. Hence, our review adopted a focus on the life course. Second, it is well known that migrants experience a range of barriers to accessing health services,[40-44] yet 'access to care' has not been a focus of prior reviews related to FGM/C. Third, several prior reviews have included studies from all over the world, thereby bringing together widely different cultural and health system contexts.[37-39] We felt that a review focused just on 'destination' countries, would provide findings that were more transferable to these specific contexts.[45,46] Finally, prior reviews have often limited their searches to English language papers or to published literature only.[35,36] By contrast, this review has included any language and a wide range of grey literature sources, and thus is able to offer an extremely comprehensive picture.[47,48]

Theoretical perspective

This review is informed by the theoretical construct of 'cultural safety'.[49] Cultural safety derives from critical social theory and argues that, traditional approaches to 'cultural awareness' or 'cultural competence' within healthcare fail to adequately take into account power relations which are historically unequal between migrant or indigenous groups and healthcare providers and services.[50] Within a cultural competence discourse for example, it is argued that "*the power to define the norm and the onus for action to understand and know about another culture fall to the health professional/service*" p.200.[49] As such, migrant groups remain represented as the 'other' and may be viewed within a deficit model - as a problem that needs to be fixed – rather

For peer review only

than as partners whose knowledge and values can contribute equally to a relationship or to service development initiatives. Culturally safe practice, by contrast, is predicated upon relationships of mutual trust and respect. It is a transformative and rights based approach, seeking to uphold the principles of respect, dignity, empowerment, safety and autonomy.[51] Cultural safety envisages the healthcare encounter as a negotiated and equal partnership in which trust plays a central role. The healthcare practitioner's role is to enable patients to say how a service can be adapted and to negotiate an agreed approach – a key aspect of shared decision making.[52] The converse of cultural safety is 'cultural risk'[53] in that culturally unsafe practices are defined as: "*any actions that diminish, demean or disempower the cultural identity and well being of an individual or group*" *p7*.[54] Cultural safety is not just a feature of individual practice however. It is argued that culturally safe practice must be institutionalised through organisational policies and structures so that it becomes part of mainstream healthcare provision rather than being dependent upon individual practitioners who may or may not adopt its values and approach.[55-57] This theoretical perspective is well suited to areas of healthcare involving stigmatised or sensitive topics, such as FGM/C, and where there is a strong need for community engagement in service development and shared decision making in care delivery.

METHODS

This qualitative systematic review is reported following the ENTREQ guidelines.[58] The review was registered in Prospero[33] and the methods are documented in detail in a published protocol.[34]

Search strategy

An exhaustive and sensitive three-step search strategy was designed by an experienced information scientist (JE). First, we searched 11 electronic databases using a combination of index terms and text-based queries. These were searched from inception to a cut-off date of 31-12-17. An example search strategy for Ovid MEDLINE is provided in Supplementary File 1. Second, we searched for relevant grey literature drawing upon five key resources, Google, Google Scholar and suggestions from the project's expert advisory group (see Table 1 for all resources/databases that were searched).[59-62] Finally, we hand searched the reference lists of

For peer review only

related systematic reviews and of all the included studies. All retrieved datasets were downloaded into group sets within an EndNote library and duplicates were removed.

For peer review only

Table 1 List of databases and resources searched

Electronic Databases Searched	Date of Search
1. Ovid multi-file search (Medline, Embase, PsycINFO)	10/03/17
2. POPline (via http://www.popline.org/) 1970 to the present	10/03/17
3. ProQuest multifile search	10/04/17
4. Applied Social Sciences Index Abstracts (ASSIA) on ProQuest 1987–current	26/05/17
5. Ovid MEDLINE 1948– and MEDLINE In-Process and Other Non-Indexed Citations to daily update	26/07/17 with monthly alert thereafter (cut-off date for included results 31/12/17)
6. Ovid EMBASE 1980–2017 Week 11	03/08/17 with monthly alert thereafter (cut-off date for included results 31/12/17)
7. CINAHL Plus with Full Text/EBSCO Host to 2017	11/08/17 with monthly alert thereafter (cut-off date for included results 31/12/17)
8. Ovid PsycINFO 1972–March Week 3 2017	14/08/17 with monthly alert thereafter (cut-off date for included results 31/12/17)
9. MIDIRS on Ovid 1971 to April 2017	18/08/17
10. HMIC on Ovid 1979 to date	18/08/17
11. Thomson Reuters Web of Science 1900–2017	18/08/17
**Grey Literature Sources**	
1. British Library Ethos (ethos.bl.uk)<br>2. Networked Digital Library of Theses and Dissertations (NDLTD) (www.ndltd.org)<br>3. NICE (https://www.nice.org.uk)<br>4. Trove – National Library of Australia (trove.nla.gov.au)<br>5. Open Grey (http://www.opengrey.eu/)<br>6. Google<br>7. Google Scholar<br>8. Experts in the field	

Screening and selection

The review included studies from any date and any language that met the following inclusion criteria: (i) empirical research, (ii) qualitative research of any design/methodology (including qualitative findings from mixed methods studies), (iii) undertaken in an OECD country (OECD was utilised as a proxy for potential and comparable high income 'destination' countries), (iv) needed to explicitly report women's or girl's experiences of seeking, accessing or receiving healthcare associated with FGM/C at any point in the life course and in any clinical setting.

Four team members (RT, CE, JMc, GH) independently assessed all titles and abstracts against the inclusion criteria. Full text versions of papers deemed to be potentially relevant were obtained and assessed. Papers found not to meet the criteria were excluded with reasons noted (see Supplementary File 2). Any areas of ambiguity were discussed with the wider project team. Non-English language papers found to be relevant on the basis of their English abstract were sent for complete academic translation.

For peer review only

Quality assessment

Study quality was assessed by two reviewers using the Joanna Briggs Institute Qualitative Assessment and Review Instrument (JBI-QARI).[63,64] Following the guidance of the Cochrane Qualitative Methods and Implementation Group[65] and the JBI,[47] reports and theses from the grey literature were appraised in the same way as published peer reviewed papers.[59,61,62] Studies were not excluded on the basis of quality, rather, the quality assessment was used to judge the relative contribution of each study to the overall synthesis, and to assess the methodological rigor of each study as part of a process of assessing confidence in the review findings.[66-70]

The JBI-QARI tool was applied to each individual paper and an aggregate score for each was calculated. A criticism of 'scoring' qualitative critical appraisals is that it can be hard to distinguish between the poor conduct of a study and poor reporting, especially where journal word limits constrain the level of detail that can be reported.[71] In addition, there is no consensus regarding the relative importance of any one domain within an assessment tool over another, and, hence, whether they should all be given an equal weight or not. In view of these concerns, we chose to adopt a 'weighting system' used in previous studies by Higginbottom et al[72,73] in which papers were grouped into one of three 'bands' – high, medium or low - to enable a broad brush evaluation to be made of their relative quality – see Table 2.

Table 2 Quality evaluation bands

Quality Evaluation	JBI QARI Tool Aggregate Score	Definition
High	Over 7	A study with a rigorous and robust scientific approach which meets most JBI benchmarks (*perhaps 7 or more 'Yes'*)
Medium	Between 5-7	A study with some flaws but not seriously undermining the quality and scientific value of the research conducted (*perhaps 5-7 'Yes'*)
Low	Under 5	A study with flaws and poor scientific value (*perhaps below 5 of the benchmarks met*)

As an additional strategy for overcoming the potential limitations of solely relying upon a checklist to assess quality, we also assessed the 'richness' of the studies. This is an approach outlined by Popay et al,[74] and subsequently operationalized in Noyes & Popay[75] and Higginbottom et al.[72,73] This approach defines study 'richness' as *"the extent to which the study findings provide explanatory insights that are transferable to other settings"* p.230.[75] 'Thick' papers create or draw upon theory to provide in-depth explanatory insights that can potentially be

For peer review only

transferable to other contexts. By contrast, 'thin' papers provide limited or superficial description and offer little opportunity for generalizing. Each paper was assessed against the criteria as set out in Higginbottom et al, p.5[72] (see Table 3) and categorized as either 'thick' or 'thin'.

Table 3 Assessment of study richness

Richness	Operational Definition
Thick papers	• Offer greater explanatory insights into the outcome of interest<br>• Provide a clear account of the process by which the findings were produced—including the sample, its selection and its size, with any limitations or bias noted—along with clear methods of analysis<br>• Present a developed and plausible interpretation of the analysis based on the data presented.
Thin papers	• Offer only limited insights<br>• Lack a clear account of the process by which the findings were produced<br>• Present an underdeveloped and weak interpretation of the analysis based on the data presented

Data extraction and assessment of relevance

Study characteristics were extracted by one author (RT) using a modified JBI template and double checked by CE. During this process, papers were categorized in terms of their relevance to the review question. This assessment was made in order to gain a better understanding of the nature of the body of evidence, and also to facilitate the coding process, as described further below. Study relevance was defined as high, medium or low as set out in Table 4. PDFs of all the included papers were imported into NVivo software and the 'findings/results' and 'discussion' sections were coded and analysed.[76]

Table 4 Assessment of study relevance

Study Relevance	Definition
High (specific)	FGM/C-specific healthcare (e.g. the study is focused on a specific aspect of care related directly to FGM/C, e.g. deinfibulation, childbirth for women who have had FGM/C, psychological care)
Medium (direct)	Other healthcare context (e.g. where the study focus is on the maternity care experience of a particular group more generally and where some of the findings relate to the experience of FGM/C)
Low (indirect)	Where the study focus is on general attitudes towards FGM/C and/or experiences and consequences of FGM - & where some FGM/C-related healthcare issues are reported, but are not the main focus of the paper

For peer review only - http://bmjopen.bmj.com/site/about/guidelines.xhtml

Data analysis and synthesis

The review adopted a thematic synthesis approach as outlined by Thomas and Harden,[77] involving four iterative stages: (i) in-depth reading of the whole papers, (ii) inductive line by line coding of the findings, (iii) grouping the codes on the basis of shared characteristics into descriptive themes, and, (iv), interpreting higher order analytical themes. Studies categorised as 'thick' and 'highly relevant' were used as index papers to develop an initial code book which was then applied across the remaining papers and expanded as appropriate. Analytical themes were evolved through an in-depth process of comparing and contrasting the meanings of the descriptive themes, analysing these in relation to how they were, or were not, able to illuminate the review questions, and inferring broader phenomena, categories of meaning or social processes that they related to.[78,79]

Rigor within the analytical process

To ensure rigor of the analytical process, the team sought to identify and understand possible 'disconfirming' cases that might challenge emerging interpretations,[80] and to explore possible sub-group or contextual differences. These processes were aided by the creation of a theme matrix (see Supplementary File 5), in which each theme was mapped to its constituent studies. This helped the team to see clearly how common the theme was amongst the studies, what kind of study contexts or samples the theme related to, and to explore why it may have been present in some studies but not in others. The whole team and wider project advisory group contributed to the evolution of the synthesis, through reading of key papers and providing feedback on the emerging interpretations. A national stakeholder event provided further feedback on the findings and helped to shape the recommendations.

Assessment of confidence in the review findings (CERQual)

Assessment of confidence in the findings of the review was undertaken using the CERQual (Confidence in Evidence from Reviews of Qualitative Research) approach.[65,66,81-87] Akin to GRADE, CERQual uses assessments of 'concerns' within four distinct domains (methodological limitations, relevance, coherence and adequacy of data) applied to each individual review finding. The CERQual assessment was applied to the descriptive rather than analytical themes,

For peer review only

as the latter comprise an aggregation of descriptive themes as well as descriptions of patterns or explanations that are inferred as part of the interpretative process. There is, as yet, relatively little guidance for, or experience with, applying CERQual to higher level analytical themes or theories.[84]

RESULTS

Search outcome

The search outcomes are reported in detail in the PRISMA flowchart in Figure 1. Fifty seven papers met the inclusion criteria, representing 55 distinct studies. Fifty three papers were in the English language; two were published in German,[88,89] and two in Spanish.[90,91]

Figure 1: PRISMA Flow diagram

Study characteristics

Due to the large numbers of studies in this review, a highly summarised description of key study characteristics and key methodological assessments is found in Table 5. Full details are provided in Supplementary Files 3 and 4.

For peer review only

Table 5 Summary study characteristics and methodological assessments

No.	Authors/date	Country	Study focus	Eligible participants & country of origin	Quality assessment	Richness	Relevance
**1.**	Abdi, R. (2012)[92]	UK	FGM/C and identity for Somali women.	16 Somali women	High	Thin	Low
**2.**	Abdullahi, A., Copping, J., Kessel, A., et al. (2009)[93]	UK	Cervical screening.	50 Somali women	Med	Thin	Med
**3.**	Ahlberg, B. M., Krantz, I., Lindmark, G., et al. (2004)[23]	Sweden	FGM/C tradition in destination countries.	50 Somali women	High	Thin	Low
**4.**	Ahmed, M., (2005)[94]	UK	Cross-cultural psychology and women's experience of FGM/C.	56 women (from Somalia & Eritrea)	Low	Thin	Low
**5.**	Ameresekere, M., Borg, R., Frederick, J., et al. (2011)[95]	USA	Maternity care experiences.	23 Somali women	Med	Thin	Med
**6.**	Asefaw, F. (2007)[88]	Eritrea and Germany	Post-migration attitudes towards FGM/C.	31 Eritrean women	Med	Thin	Low
**7.**	Baldeh, F. (2013)[96]	Scotland	Maternity care experiences.	7 women (from Gambia, Ghana & Sudan)	High	Thick	High
**8.**	Ballesteros Meseguer, C., Almansa Martinez, P., Pastor Bravo, M. d. M., et al. (2014)[90]	Spain	Health impacts of FGM/C.	9 Senegalese women	Med	Thin	Med
**9.**	Behrendt, A. (2011)[97]	Germany	Views on FGM/C.	91 women (from Ghana, Nigeria & Togo)	Med	Thin	Low
**10.**	Beine, K., Fullerton, J., Palinkas, L., et al. (1995)[98]	USA	Maternity care experiences.	14 Somali women	Med	Thin	Med
**11.**	Berggren, V., Bergstrom, S., & Edberg, A.-K. (2006)[99]	Sweden	Maternity care experiences.	21 women (from Eritrea, Somalia & Sudan)	Med	Thick	High
**12.**	Betts, V. (2011)[100]	Canada	Identity, gender, sexuality and health care experiences.	13 Somali women	Med	Thick	High
**13.**	Bravo Pastor del Mar, M. (2014)[91]	Spain	Views on FGM/C.	24 women (From Senegal, Nigeria & Gambia)	High	Thin	Low
**14.**	Bulman, K. H., & McCourt, C. (2002)[101]	UK	Maternity care experiences.	12 Somali women	Med	Thin	Med
**15.**	Carroll, J., Epstein, R., Fiscella, K., et al. (2007)[102]	USA	Healthcare experiences.	34 Somali women	High	Thick	Med
**16.**	Chalmers, B., & Omer-Hashi, K. (2002)[103]	Canada	Maternity care experiences.	415 Somali women	Med	Thick	Med

For peer review only - http://bmjopen.bmj.com/site/about/guidelines.xhtml

No.	Authors/date	Country	Study focus	Eligible participants & country of origin	Quality assessment	Richness	Relevance
**17.**	Degni, F., Suominen, S. B., El Ansari, W., et al. (2014)[104]	Finland	Reproductive health and maternity care experiences.	70 Somali women	High	Thin	Med
**18.**	d'Entremont, M., Smythe, L., & McAra-Couper, J. (2014)[105]	France	Childbirth experiences.	4 women (from different countries)	High	Thick	High
**19.**	Essén, B., Johnsdotter, S., Hovelius, B., et al. (2000)[106]	Sweden	Maternity care experiences.	15 Somali women	Med	Thin	Low
**20.**	Fawcett, L., (2014)[107]	USA	Views on FGM/C and maternity care.	30 Somali women	High	Thick	High
**21.**	Gali, M. A. (1997)[108]	USA	Reproductive healthcare experiences.	26 women (from Sudan, Eritrea, Ethiopia & Egypt)	Med	Thin	Low
**22.**	Ghebre, R. G., Sewali, B., Osman, S., et al. (2015)[109]	USA	Views on cervical screening.	23 Somali women	High	Thick	Med
**23.**	Glazer, E., (2012)[110]	Canada	Pain and embodiment associated with FGM/C.	14 Somali women	High	Thin	Low
**24.**	Glover, J., Liebling, H., Barrett, H., et al. (2017)[111]	UK	Psychological impact, resilience and experiences of FGM/C.	20 women (from Somalia, Kenya, South Africa & Gambia)	High	Thick	High
**25.**	Guerin, P. B., Allotey, P., Elmi, F. H., et al. (2006)[112]	Australia and New Zealand	Reproductive healthcare experiences.	10 women (from Somalia, Ethiopia, Eritrea, Sudan & Nigeria)	Low	Thin	Low
**26.**	Hill, N., Hunt, E., & Hyrkas, K. (2012)[113]	USA	Maternity care experiences.	18 Somali women	Med	Thick	Low
**27.**	Hussein, E., (2010)[114]	UK	Views on FGM/C & related services.	8 women (from Somalia & Sudan)	Med	Thin	Low
**28.**	Hussen, M. A. (2014)[115]	New Zealand	Views on FGM/C and healthcare experiences.	20 women (from Eritrea, Ethiopia, Somalia & Sudan)	Med	Thick	Med
**29.**	Johansen, R.E. (2017)[116]	Norway	Experiences and perceptions of premarital deinfibulation.	23 women (from Somalia & Sudan)	High	Thick	Med
**30.**	Jones, A. (2010)[117]	UK	Views and experiences of FGM/C.	6 women (from Sudan & Somalia)	High	Thick	High
**31.**	Khaja, K. (2004)[118]	Canada and USA	Views and experiences of FGM/C.	17 Somali women	High	Thin	Low
**32.**	Khaja, K., Lay, K., & Boys, S. (2010)[119]	Canada and USA	Views and experiences of FGM/C.	17 Somali women	Low	Thin	Low
**33.**	Lundberg, P. C., & Gerezgiher, A. (2008)[120]	Sweden	Maternity care experiences.	15 Eritrean women	High	Thick	High

No.	Authors/date	Country	Study focus	Eligible participants & country of origin	Quality assessment	Richness	Relevance
**34.**	Maier, C. (2003)[89]	Austria	Views, experiences and impacts of FGM/C.	3 women (from different countries)	Low	Thin	Low
**35.**	Maternity Action. (2014)[121]	UK	Maternity care experiences.	23 women (from several countries)	Low	Thin	Med
**36.**	McNeely, S., & Christie-de Jong, F. (2016)[122]	USA	Views and experiences of FGM/C.	13 Somali women	High	Thin	Low
**37.**	Moxey, J. M., & Jones, L. L. (2016)[123]	UK	Maternity care experiences.	10 Somali women	High	Thick	High
**38.**	Murray, L., Windsor, C., Parker, E., et al. (2010)[124]	Australia	Birth experiences.	10 women (from Sudan Liberia, Ethiopia & Somalia)	High	Thick	Med
**39.**	Norman, K., Gegzabher, S. B., & Otoo-Oyortey, N. (2016)[125]	UK	Views and experiences of FGM/C and community engagement.	33 women (from various countries)	Med	Thin	Low
**40.**	Norman, K., Hemmings, J., Hussein, E., et al. (2009)[126]	UK	Views and experiences of FGM/C, community engagement and services.	40 women (from Sudan, Eritrea & Somalia)	Med	Thin	Low
**41.**	O'Brien, O., Baldeh, F., Hassan, J., et al. (2017)[127]	Scotland	Views and experiences of FGM/C, community engagement and services.	48 women (from Sudan Nigeria & Zimbabwe)	High	Thin	Low
**42.**	O'Brien, O., Baldeh, F., Sivapatham, S., et al. (2016)[128]	Scotland	Views and experiences of FGM/C, community engagement and services.	39 women (from 17 African countries)	High	Thin	Low
**43.**	Palfreyman, A., Brown, E., & Nam, S. (2011)[129]	UK	Views and experiences of FGM/C, community engagement and services.	105 women (from Somali, Eritrea, Sudan, Sierra Leone & Gambia)	High	Thick	High
**44.**	Recchia, N., & McGarry, J. (2017)[25]	UK	Views on FGM/C and healthcare experiences.	6 women (from Kenya, Nigeria & Ethiopia)	Med	Thick	Low
**45.**	Safari, F., (2013)[130]	UK	Women's experience of deinfibulation.	9 women (from Somalia & Eritrea)	High	Thin	Low
**46.**	Salad, J., Verdonk, P., de Boer, F., et al. (2015)[131]	Netherlands	Cervical screening.	46 Somali women	High	Thick	Med
**47.**	Shaw, E. (1985)[132]	USA	Views on FGM/C and healthcare experiences.	12 women (from Sudan, Egypt & Somalia)	Low	Thin	Low
**48.**	Shermarke, M.A.A. (1996)[133]	Canada	Views on FGM/C.	8 Somali women	High	Thin	Med
**49.**	Straus, L., McEwen, A., & Hussein, F. M. (2009)[134]	UK	Maternity care experiences.	8 Somali women	High	Thick	High

For peer review only - http://bmjopen.bmj.com/site/about/guidelines.xhtml

No.	Authors/date	Country	Study focus	Eligible participants & country of origin	Quality assessment	Richness	Relevance
**50.**	Thierfelder, C. (2003)[135]	Switzerland	Gynaecological and maternity care experiences.	29 women (from Somalia & Eritrea)	High	Thick	High
**51.**	Thierfelder, C., Tanner, M., & Bodiang, C. M. K. (2005)[136]	Switzerland	Gynaecological and maternity care experiences.	29 women (from Somalia & Eritrea)	Med	Thin	Med
**52.**	Upvall, M. J., Mohammed, K., & Dodge, P. D. (2009)[137]	USA	Health experiences.	23 Somali women	Med	Thick	High
**53.**	Vangen, S., Johansen, R. E. B., Sundby, J., et al. (2004)[138]	Norway	Maternity care experiences.	23 Somali women	High	Thick	High
**54.**	Vaughan, C., White, N., Keogh, L., et al. (2014)[139]	Australia	Views and experiences of FGM/C, community engagement and services.	87 women (from Eritrea, Ethiopia, Somali & Sudan)	High	Thick	High
**55.**	Vaughan, C., White, N., Keogh, L., et al. (2014)[140]	Australia	Views and experiences of FGM/C, community engagement and services.	50 women (from Togo, Sudan & Kenya)	High	Thick	High
**56.**	Vloeberghs, E., van der Kwaak, A., Knipscheer, J., et al. (2012)[141]	Netherlands	Psycho-social and health experiences.	66 women (from Somalia, Sudan, Eritrea, Ethiopia & Sierra Leone)	High	Thin	Med
**57.**	Wiklund, H., Aden, A. S., Högberg, U., et al. (2000)[142]	Sweden	Maternity care experiences.	9 Somali women	Med	Thin	Med

For peer review only

The studies represented 14 different countries, including: Australia,[112,124,139,140] Austria,[89] Canada,[100,103,110,118,119,133] Finland,[104] France,[105] Germany,[88,97] Netherlands,[131,141] New Zealand,[112,115] Norway,[116,138] Spain,[90,91] Sweden,[23,99,106,120,142] Switzerland,[135,136] Scotland,[96,127,128] England[25,92-94,101,111,114,117,121,123,125,126,129,130,134] and USA.[95,98,102,107-109,113,122,132,137] The studies were a mixture of older and more recent research, with publication dates ranging from 1985 to 2017. However, 29 papers had been published since 2011, hence half of the papers reflected a more contemporary context. Over half of the included papers (n=33) were peer reviewed journal articles.[23,25,90,92,93,95,98,99,101-106,109,111-113,116,119,120,122-124,130-132,134,136-138,141,142] Other papers included 13 theses,[88,89,91,96,100,107,108,110,115,117,118,133,135] 10 unpublished research reports[97,114,121,125-129,139,140] and one book chapter.[94]

A large proportion of studies (n=18) focused specifically on women's birth/maternity care experiences.[95,96,98,99,101,103,105-107,113,115,120,123,124,134,138,142] Other studies focused on: general views on healthcare,[25,88,89,91,94,114,119,132,137,139,140] general attitudes towards FGM/C,[23,92,97,118,122,125-129,133] experiences of sexual/reproductive health services,[90,104,108,112,135,136] cervical screening,[93,102,109,131] psychological issues,[111,117,141] deinfibulation, [116,130] GP services,[121] identity,[100] pain/embodiment.[110] There were no studies that examined women's experiences of surgical reconstruction following FGM/C and no studies that examined experiences of health-service led safeguarding interventions.

The studies were primarily of adult women. Only one study included girls under the age of 18 years but did not report health-related experiences of this group.[23] There were no studies that focused specifically on healthcare issues related to FGM/C in older (post-child bearing) women. Hence, although the aim of the review was to explore FGM/C across the life course, the studies all focused on generic issues relating to adult women and no further age-specific differentiation could be made. The majority of studies were with women from FGM/C practising countries in sub-Saharan Africa, and the majority of these included women specifically from countries in the Horn of Africa where type 3 FGM/C is most commonly practised. It is likely therefore, that the findings included in the review reflect issues that may be specific to these population groups and to the experience of type 3 FGM/C. Only three studies explicitly reported including women from Egypt/Middle East but did not differentiate these women's experience from the rest of the sample.[94,108,132]

For peer review only

Methodological quality

Full details of assessments of methodological quality, richness (thick/thin) and relevance are provided in Supplementary File 4 (see Table 5 for a summary). Thirty papers were assessed as being of high quality,[23,91,92,96,102,104,105,107,109-111,116-118,120,122-124,127-131,133-135,138-141] 21 as medium quality,[25,88,90,93,95,97-101,103,106,108,113-115,125,126,136,137,142] and six as low quality.[89,94,112,119,121,132]

A methodological weakness across many studies was lack of discussion of the underpinning philosophical standpoint (question one of the QARI tool), making it difficult to assess the congruency of the chosen methodology. Likewise, many studies did not describe any clear methodology (simply stating that they adopted a generic 'qualitative approach'). Such studies tended to have 'thin' or mainly descriptive findings, and it was difficult to judge the congruence of the methodology with the research question and the methods (questions two and three on the QARI tool). Finally, a limitation across many studies was lack of discussion of reflexivity (questions six and seven on the QARI tool). Given the sensitive nature of FGM/C (and sexuality or migrant healthcare) as a topic, the failure to explore the researcher's own theoretical standpoint or their role, professional background, ethnicity, experience of FGM/C or relationship to the participants is a significant weakness, making it hard to judge the dependability of the findings.[143]

Thematic synthesis findings

The findings from 57 papers were synthesised into five analytical themes which represent a synthesis and interpretative analysis of 17 descriptive themes. Table 6 shows their inter-relationship and provides one or two quotes to illustrate each descriptive theme. Hence, the description of themes below does not include quotes. Rather, each analytical theme is explained, followed by an elaboration of its constituent descriptive themes. Due to the large number of studies that contributed findings to each descriptive theme, rather than 'crowd' the text with multiple repetitive references to these individual studies, the reader is referred to a theme matrix (Supplementary File 5) that shows which studies have contributed findings to each theme.

For peer review only

Table 6 Themes, quotations and CERQual assessment

Theme No.	Theme Heading	No. of studies	CERQual Assess-ment	Indicative Quotes
**Analytical Theme 1: Communication is key**				
1.1	Language barriers and interpretation challenges	n=31 23,93,99-105,107-109,113-115,121,123,124,126-129,131,134,136-142	High confidence	*"I wish I had a way to communicate with the doctor directly. There are things that sometimes I want to say or ask but I feel embarrassed saying it through a translator, especially on the phone…..They never translate what I'm saying. They tell me that is a shameful question..." Sweden, p.364*[137]
1.2	"Can't talk, not asked": double silence and cultural taboo	n=37 25,88,91,94-100,104,105,107,110,114,115,117,118,120-131,133,136-142	High confidence	*"If they had asked me I would have told them, but I was never asked……..I was worried and scared during my first pregnancy because I have heard people talk about the pain but I didn't know how to tell them……. I was shy." Scotland, p.23-24*[96]
1.3	Cultural (in)sensitivity	n=34 25,88,91,92,94,96-103,107,108,110-112,114,115,118,119,121,124-126,128,129,134,135,137-142	Moderate confidence	*"The doctor was not comfortable with me. He asked if I enjoy violent sex with my husband. I don't feel comfortable to talk about sex even with females— yet here is a male doctor examining my genitals and making mean comments." USA, p.274*[103]
**Analytical Theme 2: Access to care: influenced by an interaction of multi-level community and health service processes**				
2.1	Influence of cultural norms	n=35 88,89,93,95,98-104,109,110,113-118,120,121,123,124,126,130,131,134,136-142	Moderate confidence	*"Even though we had FGM type 2 or 3, had a small vaginal opening and were virgins, we did not ask for de-infibulations before marriage because that is shameful in our culture. Most of us who have FGM had problems with penetration following marriage." New Zealand, p.43*[115]<br><br>*"In my country normally…………people don't go………usually people go to a psychologist if it is something like erm….maybe chronic issues that they cannot solve at all….or sometimes if it is abnormal…. They think if you go to a psychologist you are mad or crazy." England, p.6*[117]
2.2	Influence of the family	n=19 89,92,99,100,104,105,107,109,113,117,118,120,130,131,134,137-139,142	Moderate confidence	*"I know my friend she wants to do that (deinfibulation) but her husband doesn't let her….. I told her, this is good for you - go and do. She said ok but her husband said no……Maybe if she did this (deinfibulation), it would have been a big problem in the family." England, p.157*[130]
2.3	Knowledge and information about FGM/C services	n=34 89-91,93,95,99-101,103,106,108,109,111-	Moderate confidence	*"They need to have someone that talks to you before and while you have a baby. I would have wanted someone to help me and tell me what was going to happen but I never got that… You know someone who was specially trained so you only see people who understand FGM." England, p.233*[111]

For peer review only

Theme No.	Theme Heading	No. of studies	CERQual Assess-ment	Indicative Quotes
		113,115,117,118,123-129,131,132,134,136-140,142		
2.4	'Hit and miss' care	n= 38<br>88,89,91,96,97,99-104,106-108,110,111,113-115,120-129,132-134,136,138-142	High confidence	*"Sometimes people go to one doctor after another describing their problems to them. They do not understand so they cannot find a solution. There was a woman who was suffering from bleeding and pain. When she went to the GP, he told her it was normal. He did not know how to treat her. She kept going several times and at last they opened her." England, p.41*[126]
**Analytical Theme 3: Cultural and bodily dissonance: striving for cultural and bodily integrity**				
3.1	Moving from normal to different	n=40<br>88,89,91,96,97,99,100,103,104,107,108,110-117,119-122,124,126-134,136-142	High confidence	*"Okay, now I'm missing something that other women has…..Well the first shock was to find out that people here are not circumcised…….You have to deal with that… I'm not the same as women here……….and then I started to kind of - you grieve a bit, that 'what is this thing that has been taken away from me?'……………..For me it has caused me lots of problems because when I went to health services, the minute that I say 'I was circumcised' it just create huge barrier between them and me, like you know - suddenly I'm from another planet" Australia, p.23*[139]
3.2	Threat to the self: re-living FGM/C pain during clinical interventions	n=28<br>88-90,92,93,96,99,103,105,107-111,114,115,120,122,123,126,127,131,132,136,138-141	Moderate confidence	*"You always remember it. They decide to cut me, and when she went to do it she didn't explain to me and I panic. I nearly kick her. Two of the nurses held me down. That was it…I was a little girl again being held down." England, p.228*[111]
3.3	Being opened: complexities around deinfibulation	n= 28<br>88,89,91,96,98-100,104,108,110-113,115-118,120,123,126,127,129,130,132,134,136,139,140	Moderate confidence	*She [Somali lady] hadn't talked to friends about it, just the midwife….. She said: "When I met the midwife she told me a lot about FGM. The midwife said to me 'If you want I can open it for you. If you don't want that I won't'. We talked about it a lot. After four months I tell her 'I want you to open it for me. Do me a small operation." Scotland, p.31*[127]
3.4	Being changed: complexities around re-infibulation	n= 19<br>88,89,91,98-100,104,108,111,112,116,120,127,129,130,132,135,139,140	Moderate confidence	*"I became a victim in Sudan already when I was 4 years old; I had no choice. Now I have to become a victim again after delivery, when the midwives refuse to resuture me. I just ask for a few stitches, not to have an open wound." Sweden, p.53*[99]
**Analytical Theme 4: Disempowering care encounters**				
4.1	Being exposed and humiliated	n=40<br>25,88,89,91-94,96-100,103,105,107,108,110-112,114,115,119,121-129,131,134,136-142	High confidence	*"Me - I was installed with my legs spread apart - they all came to see….and I didn't see any reason why they came to see me except for the fact that I was excised. All those people who came, who entered into my intimacy without my authorization, who didn't ask me if it bothered me.. … I felt that it was an aggression". France, p.308*[105]<br><br>*"They showed no respect to me, I understand now why people don't want to talk about it to the midwife. I will tell anyone not to tell them about being circumcised." Scotland, p.23*[96]

Theme No.	Theme Heading	No. of studies	CERQual Assess-ment	Indicative Quotes
4.2	Being judged and stereotyped	n= 36 23,25,88,89,91,95-97,99-105,107,108,111,112,114,117,119,121,124-126,129,133,134,136-142	High confidence	*One participant….described that a German gynaecologist (a woman) looked at her with so much pity and irritation after the physical examination that she felt for the first time in her life that she was not normal….she felt that she was treated like a victim who could not be talked to rationally. The participant decided…..to abstain from consulting German doctors. Germany, p.83*[97]
4.3	Lacking choice, power and control	n=32 88,89,95-101,103,105-108,110-113,118,120,121,123,124,126,127,133,134,136,138-142	High confidence	*"I can't say that the birth went well. The midwife said that I couldn't give birth without being cut ... otherwise the baby won't come without a caesarean section or I can lose the baby. Nobody asked me what I thought. I was there in pain ... without pain relief, nothing." France, p.308*[105]
4.4	Feeling unsafe and vulnerable	n= 46 23,25,88-91,95-103,105-108,110-115,117,118,120-124,126-129,131-134,136-140,142	High confidence	*"They didn't know what they were doing, and uh, they, you know they had to give me more cuts then in an awkward way… I was telling the doctor to cut the FGM and totally he ignored me. And he was cutting in the sides instead of cutting the front. It was very degrading and I am very distressed to think about it." Australia, p.25*[139]
**Analytical Theme 5: Positive care encounters**				
5.1	Trusting and appreciating providers and the system	n= 34 88-91,95,97-101,103-106,108,110,111,113,115,117,120,123,124,126-129,133,137-142	High confidence	*"I was lucky when I met a midwife in Sweden who knew about circumcised women. This was a great help to make me feel secure because it was my first time to be pregnant and to live far from my parents and family." Sweden, p.219*[120]<br><br>*"I know a friend that had to be opened before her delivery. She was lucky because she had found a midwife who knew about FGM - and she booked her to have the operation before the delivery." England, p.15*[114]
5.2	Voicing healthcare needs and preferences	n=45 25,88,89,91,93-96,98-105,107,108,110-117,119-123,125-129,132-134,136,137,139-142	High confidence	*"A while ago, all the women used to choose caesarean to give birth but now the health services know about women who are circumcised. They know of their situations and the complications they can have during maternity like bleeding and that they need help with the opening before the baby comes. When the health services don't know about your condition, they refer people to other services. There are some who are understanding, especially if different communities come to attend the clinics or hospitals, as they get more oriented on the different cultures of the communities". England, p.43*[126]

For peer review only - http://bmjopen.bmj.com/site/about/guidelines.xhtml

Analytical theme 1 - Communication is key

The synthesis revealed that communication was a key interpersonal process that underpinned women's ability to seek care, to obtain care and to have a positive care experience. Conversely, difficulties in communication negatively impacted all of these dimensions. Communication about FGM/C was characterised by issues that might be expected within migrant women's encounters with new host country healthcare systems - such as language barriers within consultations. However, communication about FGM/C presented an additional layer of complexity related to the fact that FGM/C was seen by both women and healthcare providers as a deeply personal, private and sensitive issue touching upon a range of taboos associated with gender, culture and sexuality – all issues that in many societies are shrouded in secrecy and silence. When women encountered providers who were able to overcome taboos and cultural differences, it forged bonds of trust and facilitated an environment in which women were able to talk about their FGM/C and to explore options for care. However, the majority of studies reported challenges and problems with communication and interpretation which led to women being unable to talk about their problems or to explore appropriate treatment options, led them to avoid healthcare in general and generated a sense of mistrust in the system. This theme mainly related to maternity care interactions, but also to communication about cervical cancer screening.[121,127,131,139,140]

Theme 1.1 Language barriers and interpretation challenges

The majority of studies (n=31) reported that for women who did not speak the host country language well, communication about FGM/C was hindered by language barriers and problems with accessing interpretation support that was appropriate for discussing highly sensitive and personal issues. Language barriers meant that women were unable to form a trusting relationship with their provider, express their needs adequately or understand information or advice. This led to frustration and increased anxiety, especially in a context where consultation times were often limited.[101,115] In some cases, women felt that they had had poor clinical experiences as a direct result of their inability to communicate about FGM/C.[23,115,127] In other examples, women avoided mentioning their FGM/C at all because they knew they lacked the language to explain.[114,115,136]

For peer review only

Theme 1.2 "Can't talk, not asked": double silence and cultural taboo

As a private, sensitive and taboo issue, communication around FGM/C was hindered by a double silence. Women reported that FGM/C was rarely discussed within their own communities and likewise they found it hard to discuss it with health providers, especially if these (or the interpreters) were male. For this reason, women preferred the topic to be raised by health providers, however a commonly reported finding was that, even when women appropriately accessed relevant services (for example, GPs or antenatal services,[123,125,126]) and might appreciate the general care offered,[91,104] healthcare providers often did not ask about FGM/C, even when the consultations were for pregnancy-related check-ups. The consequence was that FGM/C was sometimes not identified or discussed until women presented in the labour room, so opportunities for care planning or birth planning were missed.[96] In addition, some studies reported that women found communication to be easier when supported by a knowledgeable community advocate or confident peer.[96,100,102,121,129] Other studies emphasised that open communication was predicated upon being able to develop a trusting relationship with the health professional which in turn required time and continuity of care.[101,123,139,140]

Theme 1.3 Cultural (in)sensitivity

This theme was reported by 34 studies in which women described experiencing comments and questions from health providers that were perceived as clumsy, insensitive or intrusive. As a result, women reported feeling ashamed, scared or stigmatised. Such encounters inhibited women from talking about their FGM/C and in some cases, led to them avoiding examinations or healthcare services.[88,108] By contrast, culturally sensitive communication was greatly appreciated and enabled women to openly discuss their FGM/C and related questions.[105,123,128]

Analytical theme 2 - Access to care: influenced by an interaction of multi-level community and health service processes

The review found that access to care related to FGM/C was influenced by an interaction of factors operating at individual and community levels as well as at health system and service levels. At individual and community levels, studies showed that care seeking was influenced by cultural norms around health and sexuality, collective approaches to decision making and the

For peer review only - http://bmjopen.bmj.com/site/about/guidelines.xhtml

level of knowledge and information that individuals and communities had about FGM/C and health service availability. These factors influenced whether or not a woman would seek care, at what time period and where. However, the studies also showed that care-seeking choices were shaped by the types of services available and that outcomes were influenced by the extent to which the health system, health services and health practitioners were ready and able to provide the range of support that women may need at different points in their lives. The review showed that there were challenges within communities in terms of seeking care and finding the right services. However, even when services were appropriately accessed, care was sometimes haphazard and sub-optimal due to variable levels of staff expertise and inconsistent and unclear referral pathways, policies and procedures.

Theme 2.1 Influence of cultural norms

Women's care seeking in relation to FGM/C was strongly influenced by wider cultural norms around sexuality and health, including cultural norms on the importance of pre-marital virginity, avoiding male health providers and lack of familiarity with preventive care-seeking. These factors often led women to avoid seeking care unless symptomatic or pregnant. Thirty five studies contributed to this theme.

Women reported that strong cultural imperatives valuing premarital chastity and virginity, meant that they would avoid procedures requiring gynaecological examinations (e.g. pap smears[109,126,131,140]), and, especially for those with type 3 FGM/C, the general norm was to avoid seeking deinfibulation prior to marriage and pregnancy due to the cultural pressure to maintain chastity, prove their virginity and to remain 'closed' for their husbands.[109,115,116,123,132,135] This norm was so strong that some studies reported younger women wishing that they could undergo deinfibulation, experiencing unpleasant symptoms and being aware of services, but feeling unable to resist community pressure.[116,117,130] Some exceptions to this norm were reported, but mainly in the context of needing help for particularly difficult or painful symptoms.[123]

Care seeking in relation to mental health issues associated with FGM/C was infrequently reported and a few studies suggested that this may also be linked to cultural norms in which

For peer review only

mental health is still often viewed as a stigmatised issue and, like FGM/C itself, is seen as hard to talk about.[113,115,117,126,133]

Theme 2.2 Influence of husbands and the family

Nineteen studies reported that access to care in terms of healthcare decision making, especially among Somali women, was strongly influenced by the views and advice of the wider family and peer group. In the maternity context, this had an impact on decision making around uptake of antenatal care and caesarean section.[100,107,109,113,117,123,142] The family also influenced decision making around FGM/C specifically. For example, as mentioned above, women considering premarital deinfibulation reported that they might avoid informing their direct family members as they were aware that they would otherwise come under strong pressure to change their mind due to fears over their future marital prospects.[129,130] For married women with type 3 FGM, studies indicated that the views of husbands were of paramount importance and that women generally felt that they needed their husband's permission to undergo deinfibulation (outside of the context of delivery).[130]

Theme 2.3 Knowledge and information about FGM/C services

Women's knowledge of, and familiarity with, health services was variable. In some cases, women lacked familiarity with the host country health system and this impeded their ability to access care in general. In other cases, women reported being quite familiar with maternity and primary care services, but lacked knowledge and information of FGM/C-specific specialist services (especially non-maternity related services such as counselling) and where and when it may be appropriate to seek help.[117,126,127] One study from the UK suggested that this might be a particular issue for women living in low prevalence areas.[125]

Theme 2.4 'Hit & miss' care

When accessing healthcare, women reported that (i) the identification of FGM/C and (ii) provision of appropriate treatment or referrals could be a "hit and miss" process, depending upon individual provider characteristics and practices - rather than being a result of standardised organisational systems and processes. For example, many studies cited situations where

For peer review only - http://bmjopen.bmj.com/site/about/guidelines.xhtml

opportunities to identify FGM/C during a consultation were missed, which was attributed to providers lacking awareness of FGM (or related specialist services) and hence failing to ask the right questions, or to undertake an examination, or to make timely referrals.[126] Maternity services were more likely to have knowledgeable providers and responsive reporting and follow up systems, but even here, women described situations where they felt their FGM/C had been mismanaged due to lack of awareness and lack of appropriate referrals.[91,121,126,129] The most common issue was failure to have been asked about FGM/C so that it was not included in birth planning discussions and was identified only once labour had started.[111] This theme, was reported in 38 studies, across different countries and time periods.

Analytical theme 3 - Cultural and bodily dissonance: striving for cultural and bodily integrity

This analytical theme refers to changes in and challenges to, women's sense of cultural and bodily identity and integrity as a result of their experiences associated with FGM/C in the host country, and particularly through their encounters with the healthcare system (manifested, for example, during experiences of clinical examinations, childbirth, deinfibulation and reinfibulation). The theme describes women's responses to experiences of dissonance, whereby their decision making and actions can be understood as a desire to maintain a sense of cultural and bodily integrity. This theme highlights differences in, and changes to, cultural meanings and values related to FGM/C and how these differences and changes influence the way that women and health professionals define health issues, make decisions around FGM/C care and experience that care. When mutual interpersonal cultural understanding was not achieved, women perceived sub-optimal care and distrusted the health system and their health providers. If women felt understood, they felt safe and reassured, resulting in a sense of bodily and emotional integrity.

Theme 3.1 Moving from normal to different

Some women described becoming aware of FGM/C as something that is 'different' only once they moved to another culture. At this point, some started to become aware of the suffering and symptoms that their FGM/C may have caused them. They started to question and to resist previously taken for granted aspects of their culture and to feel uncomfortable with their own bodies and sexual identity.[139] However women also reported feeling shame and anger at being

labelled as 'different' and 'mutilated' by the dominant discourse in the host country and by health professionals, and felt that their culture was misunderstood.[92,93,105,129] This experience of dissonance made some women feel reluctant to mention FGM/C to health providers or caused them to feel extremely uncomfortable during healthcare consultations, thereby limiting the opportunity to discuss the issues more openly and to achieve greater mutual understanding.[99,107,139] This theme was very common - reported in 40 studies.

Theme 3.2 Threat to the self: re-living FGM/C pain during clinical interventions

Many studies (n=28) reported women's experiences of gynaecological clinical interventions and childbirth as events which threatened their sense of bodily and cultural integrity. Women described great pain, suffering, fear and apprehension around clinical interventions – and related this to their previous healthcare experiences and, especially, to re-living the original trauma they had experienced during their FGM/C. These emotions were exacerbated in healthcare encounters where women felt a loss of control or lack of respect. Negative experiences could lead to women avoiding attending the hospital until the last minute as a way of avoiding medical interventions.[144]

Theme 3.3 Being opened: complexities of deinfibulation for women with type 3 FGM

The experience of dissonance and quest for integrity was also seen in views and experiences around deinfibulation and was reported by 28 studies. As noted above, for cultural reasons, 'being opened' medically was seen as necessary primarily after marriage and primarily in the context of pregnancy and childbirth - rather than before.[118,119] There were no studies that explicitly or exclusively focused on the views or experiences of unmarried women, but some studies reported examples of unmarried women where the need to maintain cultural and bodily integrity outweighed the experience of physical pain or discomfort, even when women knew about the option to seek surgical deinfibulation.[115-117] There were few reported exceptions to this rule, although one study suggested that for some women, finding out about the option for surgical deinfibulation was empowering and deinfibulation was subsequently seen as a way of asserting control over their bodies and lives.[129]

For peer review only

Most studies reported that women preferred to be 'opened' during labour to avoid the pain and trauma of being cut twice.[107,110,116,123,130,135] However, some studies suggested that this view could change towards the medically preferred option of antepartum deinfibulation, if there were opportunities for women to have appropriate discussions, conducted in a sensitive way in the context of a trusting relationship with a healthcare provider.[99,127] Hence, given that FGM/C is often not discussed during healthcare consultations, women's current reported preferences around deinfibulation timing may also reflect the fact that they may not be receiving the right information at the right time, and thus may not be in a good position to make informed decisions on this issue.[88,115,127,129]

Theme 3.4 Being changed: complexities around reinfibulation

A consistent finding across the studies within this theme (n=19) was that 'being opened' could involve significant emotional, physical, social and relational adjustments as women's bodies became changed. For some women, deinfibulation was firmly welcomed, although still required a period of 'getting used to' a different way of looking and feeling.[130,139] For other women however, (especially women from Sudan where it is a cultural norm in some regions to be closed again after delivery) there was ambivalence about these changes and some women requested to be 'closed again' to varying degrees.[98,100,115,116,120,129,130,139]

In most OECD countries, reinfibulation is illegal and some studies reported women feeling very upset when their requests were denied by health professionals, experiencing this as a denial of their agency and integrity.[88,112] Consequently, women in one study reported seeking medical deinfibulation during holidays back home.[127]

Analytical theme 4 - Disempowering care encounters

In many situations, the care received by women in relation to their FGM/C was experienced as fundamentally disempowering. Women described experiencing negative attitudes or behaviours from health professionals and poorly managed clinical interventions in which they felt they were not respected or listened to. Many studies reported examples of care encounters that left women feeling re-traumatized, voiceless, without power to question interventions, and vulnerable in the hands of unprepared system. Such experiences caused women to feel unsafe and disempowered.

For peer review only

There were many examples where women felt that care had gone wrong or where experiences had been poor, and that these situations could have been avoided with better communication, cross-cultural understanding and sensitivity. This analytical theme is made of four descriptive themes that cut across all countries, time periods, population groups and care contexts.

Theme 4.1 Feeling exposed and humiliated

Across many studies (n=40), women reported that the reactions of healthcare professionals to their FGM/C had left them feeling ashamed, objectified, humiliated and exposed. This was primarily the case with healthcare professionals who were unfamiliar with FGM/C and who were encountering it for the first time. In these situations, women reported that healthcare professionals often reacted with extreme shock when they saw that a woman had been cut. Some tried to hide their shock and avoided mentioning anything at all making the encounter feel uncomfortable. Others openly expressed horror or disgust or reacted insensitively by asking intrusive or inappropriate questions. In many cases, other colleagues were called in to be consulted or to 'have a look' (sometimes without consent [99,103]) causing women to feel that their privacy had been violated and that they had been put on display. Such experiences left women feeling angry, distressed or as if they had been re-violated.[105,107] In some cases, these experiences left women wanting to avoid further contact with services and/or feeling unable to discuss their care any further as it was clear that the provider had limited knowledge about FGM/C.[108] Several studies made the point that negative experiences of a particular service or provider would then be discussed within peer and community networks, and hence could have an impact beyond the individual woman, potentially affecting the way others in the community might engage with care.[88,96,104,107,111,119,141]

Theme 4.2 Feeling judged and stereotyped

In many studies (n=36) women reported feeling judged and discriminated against within some of their healthcare encounters. They reported feeling that health providers made negative assumptions about them and provided sub-optimal care based on racial, religious or other stereotypes and misconceptions about their culture in general as well as about FGM/C. Such experiences caused distress, anger and avoidance of the health provider or service.[99]

Theme 4.3 Lack of choice, power and control

In this theme, reported by 32 studies, women described experiences where they felt they had lacked choice or control within the healthcare encounter, especially in the maternity setting and around key interventions such as caesarean sections or episiotomies. Lack of control was experienced in terms of feeling excluded from healthcare decision making, not being informed, not being listened to, feeling at the mercy of 'the system' and unable to express their needs. Women's attempts to assert control were sometimes construed by health professionals as being 'difficult'.[95,103,107,111,113] In labour, women described that FGM/C was often not discussed at all or that they were not consulted about interventions such as deinfibulation or caesarean section. This led to suspicion and fear and was particularly reported in studies conducted with the Somali community.[105]

Theme 4.4 Feeling unsafe and vulnerable

Many studies (n=46) reported care encounters where women felt unsafe and vulnerable, primarily when they were treated by providers who they perceived to be inexperienced in dealing with FGM/C, and when they faced language barriers or were unable to express their own preferences. Such encounters often led to perceived poor quality care or poor clinical outcomes.[101] The consequences ranged from providers failing to recognise, identify or discuss FGM/C,[96] to situations where women felt their care had been adversely affected as a direct result of poor provider skills (for example having unnecessary caesarean sections or extensive perineal tearing).[23,25,88,91,96,115,120,123,126,137,138] Women endured such situations feeling unsafe and highly vulnerable, often describing painful and traumatic experiences.[139] Such feelings of vulnerability were heightened for women who lacked social support.[99,105,114,123] In contrast, women reported feeling safe and highly reassured when they encountered providers who appeared to be knowledgeable or experienced.[96,101,111,120,126,128,129,139,140]

Analytical theme 5 - Positive care encounters

As seen above, the synthesis has revealed many reports of poor care and difficult experiences in relation to FGM/C. However, many studies reported a mixture of experiences, including both good and poor care. High quality care was conceptualised by women as care that is safe,

For peer review only - http://bmjopen.bmj.com/site/about/guidelines.xhtml

respectful, culturally sensitive and compassionate. The essential process underpinning the experience of 'good' care was having a sense of trust – both in the individual provider and also in the 'system' as a whole. Trust was essential for, and developed from, positive care experiences. Positive experiences were associated with the development of a good relationship with health professionals who were perceived to be clinically knowledgeable and culturally sensitive, facilitating open communication and a sense of being in safe hands. Trust in the 'system' was linked to women's appreciation of the availability and accessibility of services (compared with their home countries) but particularly when services were perceived to be prepared to be responsive to community needs (e.g. provision of appropriate interpretation services) and to manage issues relating to FGM/C (e.g. by having specialist services or specialist providers).

Theme 5.1 Trusting and appreciating providers and the system

This theme, reported in 34 studies showed that women appreciated the good medical services available in the host country. Women also expressed great appreciation for providers who made them feel safe and respected. Such providers were described as knowledgeable and experienced, who treated women with respect, who understood their individual needs and who involved them in their care. Women described these characteristics as facilitating trust and leading to open communication about FGM/C. For example, they were more likely to engage in conversations about their care (e.g. birth planning) and, in some cases, were more likely to follow current medical guidelines, for example, on issues such as deinfibulation timing.[114] A few studies explicitly linked the ability to form good relationships to services that enabled continuity of care.[101,123]

Theme 5.2 Voicing healthcare needs and preferences

Women's recommendations for good quality safe FGM/C related care and services they could trust were reported in 45 studies. They included: (i) interpersonal provider characteristics and behaviours (such as providers being willing and able to talk about FGM/C, providers being skilled and knowledgeable around FGM/C, providers offering culturally sensitive and respectful care) and (ii) service organisation issues (such as having specialist services for FGM/C, being

For peer review only

given information and awareness about FGM/C-related services, and involving women and affected communities in FGM service development).

CONFIDENCE IN THE REVIEW FINDINGS

Confidence in review findings (as assessed by GRADE CERQual) ranged from high to medium and there was strong consistency in the findings across countries, population groups and clinical contexts (see Table 6 for the final CERQual assessment for each theme and Supplementary File 6 for the full details of the CERQual evaluation).

DISCUSSION: ACHIEVING CULTURALLY SAFE CARE FOR WOMEN WITH FGM/C

The review shows that FGM/C - literally and symbolically - embodies complex processes of cultural change and cultural interaction that are occurring as a result of globalisation, migration and super-diversity. These processes manifest themselves very concretely in healthcare in terms of influencing how individuals seek care, how care is delivered and experienced by all involved, and how services are configured.[2,145,146]

This review has shown that, when obtaining care for FGM/C-related issues in a host country, women face common challenges that affect many groups of migrants, but that these are exacerbated.[44,73,147-150] Silence, secrecy, stigma and lack of familiarity with FGM/C within the system combine and act as obstacles to identifying FGM/C or to providing women with appropriate care. The care for FGM/C described in this review exemplifies the challenges of achieving 'cultural safety' in health services. Many of the experiences reported in the review describe a situation of physical but also cultural 'vulnerability' or 'risk' whereby women felt physically unsafe but also disrespected in terms of their cultural identity and bodily integrity. Many of the themes in this review referred specifically to maternity care contexts, and there are striking similarities with themes identified in studies across the world, that highlight disrespect and abuse in maternal healthcare.[35,36,151] However, as this review shows, the same challenges also appear to exist for women with FGM/C in primary care and other health settings.[152-154].

This review has also shown that, in some circumstances, women and providers can overcome

For peer review only

these obstacles. Key to this are knowledge of services, community engagement, the availability of specialist services, continuity of care, shared decision making, addressing language barriers and providing care that is person centred, culturally sensitive and respectful.[42,148,155,156] Similar recommendations have also been made in other studies exploring the key elements of 'respectful care' in a variety of settings.[157,158]

Figure 2 outlines a model, derived from the review findings, of the key elements of 'culturally safe' care for women/girls with FGM/C, set within a wider context of super-diversity.

Figure 2 : Culturally safe care for FGM/C

For peer review only

RECOMMENDATIONS FOR CULTURALLY SAFE CARE

The review recommendations fall into three overlapping areas.

Overcoming barriers: community engagement for knowledge and awareness

The review showed that there is a need for communities to be more aware of FGM/C related services and of potential interventions. However, it also highlighted the challenges that women may have in discussing FGM/C within their family or community and with a health provider. This finding highlights a need for ongoing community involvement and engagement to raise awareness of services and provide support to women who may wish to access help (especially for unmarried or non-pregnant women).[30,31] The review suggests that service models which involve community advocates or community liaison workers may be particularly helpful in encouraging women to access care and to overcome communication difficulties with providers. Similar models have been found to be effective in other areas of healthcare.[159-163]

Breaking the silence: communication, continuity and shared decision making

Given the communication challenges around FGM/C, the review findings strongly suggest that the onus of 'breaking the silence' may lie with the healthcare provider, with women stating that they expected and wanted health professionals to raise the issue. It may be appropriate for services to consider routinizing questions around FGM/C in key clinical settings, as has been done for other 'sensitive' clinical issues such as domestic violence or HIV.[164-167] However, the review also highlighted that providers may lack the confidence or cultural competence to raise the subject in an appropriate and respectful way, potentially missing opportunities to identify FGM/C in a timely manner but also missing opportunities to discuss prevention/safeguarding. Communication challenges can be addressed through staff and interpreter training.[168] However, for such a sensitive topic, the review strongly suggests that respectful open discussions are more likely to happen in a context of a trusting relationship, and that such relationships are facilitated through models such as continuity of care and shared decision making.[152-154,169] Given the particular global concern for women's (and their babies') well-being related to timing of deinfibulation surgery (for women with type 3 FGM/C), the review findings strongly suggest that women appreciate, and would benefit from, more discussion, information and advice – at

For peer review only

different stages in their life (before and after marriage) and both pre- and post- deinfibulation.

Getting care pathways right: clear systems and holistic services

The review suggests that women sometimes receive sub-optimal care due to their FGM/C being missed or because of a lack of appropriate referral and reporting pathways. Some countries have made great strides to develop clear processes in this area (e.g. UK), but in others, service provision remains ad-hoc.[21] As per WHO guidelines,[9,20] the review affirms the ongoing need to develop holistic care pathways.[13,26,170-173] The review findings suggest that women have better experiences in places where there are specialist services with confident and competent staff, and that women in lower prevalence or rural areas may be at a particular disadvantage. Hence, it is important for regions to develop service models that can address these inequalities.

In addition, the review has highlighted the ongoing need for the availability of mental health assessment and support where required. In particular, the review showed that for some women affected by FGM/C, childbirth and other clinical interventions could be traumatising, triggering flashbacks of their original cutting, creating profound anxiety and distress, and potentially leading to poor care experiences or avoidance of services.[174,175] The reviews were unable to illuminate in any further depth, how such women might best be identified or treated, or which women might be most at risk. This is an important agenda for future research.[13,39,176-180] However, most maternity care services have well established processes in place for dealing with highly anxious women or women who have experienced trauma. It would seem important for services to ensure that these interventions are also available for women affected by FGM/C.[35,181]

STRENGTHS AND LIMITATIONS

The review has several strengths. First, it adopted an exceptionally comprehensive and exhaustive search and we are confident that the majority of relevant papers were identified. Second, it has focused exclusively on OECD countries, and its findings are, therefore, highly relevant to health service development in most destination countries. Third, it is the first to have considered all aspects of FGM/C related healthcare at all stages of the life course and in all clinical settings. By moving beyond the usual focus on maternity care contexts,[35,36] the review has been able to advance current knowledge beyond an understanding of experiences (although

For peer review only

this is important) to provide a more analytical and holistic interpretation of factors influencing care-seeking, decision making for deinfibulation surgery, service access and the development of care pathways.

The main limitation of the review is that the findings inevitably reflect the methodological limitations of its included studies. Many papers displayed a homogenising tendency in terms of the affected communities as well as FGM/C types. For example, in papers that had mixed community samples, there was little attempt to explore cultural differences in relation to FGM/C. Likewise, it was not always clear which type of FGM/C was being studied. In research with Somali or Sudanese communities, the assumption can reasonably be made that their focus was FGM/C type 3. However, in studies with mixed samples, there was very little differentiation on the basis of FGM/C type. As a result, there is limited specific knowledge on the needs or experiences of women with other FGM/C types.

EVIDENCE GAPS AND FUTURE RESEARCH

The review has identified four key gaps in the evidence base around FGM/C, all of which are priority areas for future research. First, there were very few studies that focused specifically on mental health needs. Second, there is a lack of studies on girls' or unmarried/non-pregnant women's experiences around FGM. Important areas for future investigation amongst this group are care seeking and decision making (especially around deinfibulation timing). Third, there were no in-depth evaluations of interventions or services which means that development of models of 'good care' (e.g.[114,182]) still need to be inferred rather than being based upon sound evidence. Further mapping and evaluation of models of care would be beneficial to understand better their differential impact on accessibility, outcomes, cost and patient satisfaction. Finally, there was no research that specifically explored women's/girls' experiences of healthcare professional's involvement in implementation of legal safe guarding or prevention interventions, which, in some countries (such as the UK), are inserting new complexities into the patient-professional relationship and reportedly undermining trust.[31,32,183,184]

For peer review only

CONCLUSION

This review has identified key challenges but also opportunities for the development of culturally safe and accessible services to improve care for women affected by FGM/C. Future research should involve communities to evaluate existing models of care in order to inform best practice.

ACKNOWLEDGEMENTS

We would like to thank all those who have contributed time and valuable insights to this project. We would particularly like to thank all those who gave up their day to attend the national stakeholder consultation event. Special thanks go to Carol McCormick (FGM/C specialist midwife) for her support and input at the start of this project. We gratefully acknowledge the invaluable contributions of our project advisory group:

- Kinsi Clarke, Nottingham and Nottinghamshire Refugee Forum, co-founder of Nottingham FGM survivor's group
- Professor Jim Thornton, consultant obstetrician, Nottingham University Hospital Trust and University of Nottingham
- Amanda Wickham (FGM/C specialist midwife), Nottingham University Hospital Trust
- Helen Denness, consultant in public health, Nottingham city council
- Grace Brough, Insight specialist (public health), Nottingham city council, Chair of Nottingham and Nottinghamshire FGM/C Steering Group

CONTRIBUTORS

CE and VN conceived the study. CE obtained funding, oversaw all aspects of the project and contributed to all stages. CE drafted this paper. RT contributed to all stages of the review, including data extraction, coding, quality appraisal and CERQual components. JE designed and executed all the searches. JMc assisted with quality assessment and contributed to the evolving synthesis. GH assisted with quality assessment and contributed to the evolving synthesis. JA provided clinical expertise and contributed to the evolving synthesis and formulation of recommendations/conclusions. VN provided a community perspective and contributed to the evolving synthesis and formulation of recommendations/conclusions.

For peer review only

FUNDING STATEMENT

This project was funded by the NIHR HS&DR Programme (no. 15/137/04). The views expressed in this report are those of the authors and not necessarily those of the NHS, the NIHR or the Department of Health and Social Care.

COMPETING INTERESTS

None declared.

PROVENANCE AND PEER REVIEW

Not commissioned. No additional data available.

DATA SHARING STATEMENT

No additional data are available.

OPEN ACCESS

This is an Open Access article distributed in accordance with the Creative Commons Attribution Non Commercial (CC BY-NC 4.0) license, which permits others to distribute, remix, adapt, build upon this work non-commercially, and license their derivative works on different terms, provided the original work is properly cited and the use is non-commercial.
See: http://creativecommons.org/licenses/by-nc/4.0/–

For peer review only

REFERENCES

1. Zeeb H, Makarova N, Brand T, et al. Super-diversity: a new concept for migrant health? *Public Health Forum* 2015;23:124-25.

2. Vertovec S. Super-diversity and its implications. *Ethn Rac Stud* 2007;30:1024-54.

3. Rechel B, Mladovsky P, Ingleby D, et al. Migration and health in an increasingly diverse Europe. *The Lancet* 2013;381:1235-45.

4. Phillimore J. Approaches to health provision in the age of super-diversity: accessing the NHS in Britain's most diverse city. *Crit Soc Policy* 2011;31:5-29.

5. Phillimore J. Migrant maternity in an era of superdiversity: new migrants' access to, and experience of, antenatal care in the West Midlands, UK. *Soc Sci Med* 2016;148:152-9. doi: 10.1016/j.socscimed.2015.11.030

6. United Nations Children's Fund. Female Genital Mutilation/Cutting: A Global Concern. New York: UNICEF, 2016.

7. Van Baelen L, Ortensi L, Leye E. Estimates of first-generation women and girls with female genital mutilation in the European Union, Norway and Switzerland. *Eur J Contracept Reprod Health Care* 2016;21(6):474-82.

8. WHO. Female Genital Mutilation: Fact Sheet. Geneva: World Health Organisation, 2014.

9. WHO. WHO Guidelines on the Management of Health Complications from Female Genital Mutilation. Geneva: WHO, 2016.

10. Royal College of Obstetricians and Gynaecologists. Green-Top Guideline No.53: Female Genital Mutilation and its Management. London: Royal College of Obstetricians and Gynaecologists, 2015.

11. Royal College of Midwives, Royal College of Nursing, Royal College of Obstetricians and Gynaecologists, et al. Intercollegiate Guidelines Tackling FGM in the UK: Intercollegiate Recommendations for Identifying, Recording and Reporting. London, , 2013.

12. Royal College of Nursing. Female Genital Mutilation: An RCN Resource for Nursing and Midwifery Practice (2nd ed). London: Royal College of Nursing, 2015.

13. Abdulcadir J, Rodriguez M, Say L, et al. Research gaps in the care of women with female genital mutilation: an analysis. *Br J Obstet Gynaecol* 2015;122(3):294-303. doi: 10.1111/1471-0528.13217

14. Esu E, Udo A, Okusanya BO, et al. Antepartum or intrapartum deinfibulation for childbirth in women with type III female genital mutilation: a systematic review and meta-analysis. *Int J Gynaecol Obstet* 2017;136 Suppl 1:21-29.

For peer review only

15. Albert J, Bailey E, Duaso M. Does the timing of deinfibulation for women with type 3 female genital mutilation affect labour outcomes? *Br J Midwifery* 2015;23(6):430-37.

16. Paliwal P, Ali SA, Bradshaw S, et al. Management of type III female genital mutilation in Birmingham, UK: a retrospective audit. *Midwifery* 2014;30(3):282-88.

17. Okusanya BO, Oduwole O, Nwachuku N, et al. Deinfibulation for preventing or treating complications in women living with type III female genital mutilation: a systematic review and meta-analysis. *Int J Gynaecol Obstet* 2017;136 Suppl 1:13-20.

18. Gayle C, Rymer J. Female genital mutilation and pregnancy: associated risks. *Br J Nurs* 2016;25(17):978-83.

19. NHS England. Commissioning Services to Meet the Needs of Women and Girls with FGM. London, , 2018.

20. WHO. Care of Girls and Women Living with Female Genital Mutilation: A Clinical Handbook. Geneva: World Health Organisation, 2018.

21. Johansen RE, Mahgoub-Ziyada M, Shell-Duncan B, et al. Health sector involvement in the management of female genital mutilation/cutting in 30 countries. *BMC Health Serv Res* 2018;18:240.

22. Waigwa S, Doos L, Bradbury-Jones C, et al. Effectiveness of health education as an intervention designed to prevent female genital mutilation/cutting (FGM/C): a systematic review. *Reprod Health* 2018;16:62.

23. Ahlberg BM, Krantz I, Lindmark G, et al. 'It's only a tradition': making sense of eradication interventions and the persistence of female 'circumcision' within a Swedish context. *Crit Soc Policy* 2004;24(1):50-78. doi: 10.1107/0261018304039679

24. Berg RC, Denison E. A tradition in transition: factors perpetuating and hindering the continuance of female genital mutilation/cutting (FGM/C) summarized in a systematic review. *Health Care Women Int* 2013;34(10):837-59.

25. Recchia N, McGarry J. "Don't judge me": narratives of living with FGM. *Int J Hum Rights Healthc* 2017;10(1):4-13. doi: 10.1108/Ijhrh-10-2016-0016

26. Baillot H, Murray N, Connelly E, et al. Addressing female genital mutilation in Europe: a scoping review of approaches to participation, prevention, protection and provision of services. *Int J Equity Health* 2018;17(1):21. doi: 10.1186/s12939-017-0713-9

27. Dawson A, Homer CSE, Turkmani S, et al. A systematic review of doctors' experiences and needs to support the care of women with female genital mutilation. *Int J Gynecol Obstet* 2015;131(1):35-40.

28. Dawson A, Turkmani S, Fray S, et al. Evidence to inform education, training and supportive work environments for midwives involved in the care of women with female genital

For peer review only

mutilation: a review of global experience. *Midwifery* 2015;31(1):229-38. doi: 10.1016/j.midw.2014.08.012

29. Reig-Alcaraz M, Siles-Gonzalez J, Solano-Ruiz C. A mixed-method synthesis of knowledge, experiences and attitudes of health professionals to female genital mutilation. *J Adv Nurs* 2015 doi: 10.1111/jan.12823

30. Connelly E, Murray N, Baillot H, et al. Missing from the debate? a qualitative study exploring the role of communities within interventions to address female genital mutilation in Europe. *BMJ Open* 2018;8:e021430.

31. Dixon S, Agha K, Ali F, et al. Female genital mutilation in the UK- where are we, where do we go next? Involving communities in setting the research agenda. *Res Involv Engagem* 2018;4:29. doi: 10.1186/s40900-018-0103-5 [published Online First: 2018/09/22]

32. Plugge E, Adam S, El Hindi L, et al. The prevention of female genital mutilation in England: what can be done? *J Public Health (Oxf)* 2018 doi: 10.1093/pubmed/fdy128 [published Online First: 2018/07/31]

33. Evans C, McGarry J, McCormick C, et al. Exploring health professionals' views on, and experiences of, providing care for women and girls who have undergone female genital mutilation (FGM): a qualitative systematic review Protocol, PROSPERO, CRD42015030004, 2015.

34. Evans C, Tweheyo R, McGarry J, et al. What are the experiences of seeking, receiving and providing FGM-related healthcare? Perspectives of health professionals and women/girls who have undergone FGM: protocol for a systematic review of qualitative evidence. *BMJ Open* 2017;7:e018170.

35. Hamid A, Grace K, Warren N. A meta-synthesis of the birth experiences of African immigrant women affected by female genital cutting. *J Midwifery Womens Health* 2018;00:1-11.

36. Turkmani S, Homer CSE, Dawson A. Maternity care experiences and health needs of migrant women from female genital mutilation-practicing countries in high-income contexts: A systematic review and meta-synthesis. *Birth* 2018 doi: 10.1111/birt.12367

37. Smith H, Stein K. Health information interventions for female genital mutilation. *Int J Gynaecol Obstet* 2017;136 Suppl 1:79-82.

38. Smith H, Stein K. Surgical or medical interventions for female genital mutilation. *Int J Gynaecol Obstet* 2017;136 Suppl 1:43-46.

39. Smith H, Stein K. Psychological and counselling interventions for female genital mutilation. *Int J Gynaecol Obstet* 2017;136 Suppl 1:60-64.

For peer review only

40. Suphanchaimat R, Kantamaturapoj K, Putthasri W, et al. Challenges in the provision of healthcare services for migrants: a systematic review through providers' lens. *BMC Health Serv Res* 2015;15:390. doi: 10.1186/s12913-015-1065-z

41. Robertshaw L, Dhesi S, Jones L. Challenges and facilitators for health professionals providing primary healthcare for refugees and asylum seekers in high-income countries: a systematic review and thematic synthesis of qualitative research. *BMJ Open* 2017;7:e015981.

42. O'Donnell C, Higgins M, Chauhan R, et al. Aylum seekers' expectations of, and trust in, general practice: a qualitative study. *Br J Gen Pract* 2008;58:870-76.

43. Ochieng B. Black African migrants: the barriers with accessing and utilising health promotion services in the UK. *Eur J Public Health* 2013;23(2):265-69.

44. Agudelo-Suarez A, Gil-Gonzalez D, Vives-Cases C, et al. A metasynthesis of qualitative studies regarding opinions and perceptions about barriers and determinants of health services' accessibility in economic migrants. *BMC Health Serv Res* 2012;12:461.

45. Hannes K, Harden A. Multi-context versus context-specific qualitative evidence syntheses: combining the best of both. *Res Synth Methods* 2011;2(4):271-78. doi: 10.1002/jrsm.55

46. Weir MC, Grimshaw JM, Mayhew A, et al. Decisions about lumping vs. splitting of the scope of systematic reviews of complex interventions are not well justified: a case study in systematic reviews of health care professional reminders. *J Clin Epidemiol* 2012;65(7):756-63. doi: 10.1016/j.jclinepi.2011.12.012 [published Online First: 2012/04/14]

47. Joanna Briggs Institute. JBI Reviewer's Manual https://wiki.joannabriggs.org/display/MANUAL/Chapter+2%3A+Systematic+reviews+of+qualitative+evidence: (accessed 12-07-18); [

48. Noyes J, Hannes K, Booth A, et al. Qualitative and Implementation Evidence and Cochrane Reviews, Version 1.02a. In: Higgins JA, Green S, eds. Cochrane Handbook for Systematic Reviews of Interventions Version 5/3 (updated October 2015): The Cochrane Collaboration, 2015.

49. Ramsden I. Cultural Safety and Nursing Education in Aotearoa and Te Waipounamu. Victoria University, 2002.

50. Browne A, Varcoe C, Smye V, et al. Cultural safety and the challenges of translating critically oriented knowledge into practice. *Nurs Philos* 2009;167-179

51. Phiri J, Dietsch E, Bonner A. Cultural safety and its importance for Australian midwifery practice. *Collegian* 2010;17(3):105-11.

52. Duke J, Connor M, McEldowney R. Becoming a culturally competent health practitioner in the delivery of culturally safe care. *J Cult Divers* 2009;16:40-49.

For peer review only

53. Gerlach A. A critical reflection on the concept of cultural safety. *Can J Occup Ther* 2012;79(3):151-58.

54. Cooney C. A comparative analysis of transcultural nursing and cultural safety. *Nurs Prax N Z* 1994;9(1):6-11.

55. Brascoupé S, Waters C. Cultural safety: exploring the applicability of the concept of cultural safety to aboriginal health and community wellness. *J Aborig Health* 2009(November):6-41.

56. Wepa D. Cultural Safety in Aotearoa New Zealand. North Shore City: Pearson Prentice Hall 2005.

57. Johnston M, Kanitsaki O. An exploration of the notion and nature of the construct of cultural safety and its applicability to the Australian healthcare context. *J Transcult Nurs* 2007;18:247-56.

58. Tong A, Flemming K, McInnes E, et al. Enhancing transparency in reporting the synthesis of qualitative research: ENTREQ. *BMC Med Res Methodol* 2012;12(1):181. doi: 10.1186/1471-2288-12-181

59. National Information Center on Health Services Research and Healthcare Technology (NICHSR). Health Services Research and Health Policy Grey Literature Project: Summary Report: U.S. National Library of Medicine, 2006.

60. Adams J, Hillier-Brown FC, Moore HJ, et al. Searching and synthesising 'grey literature' and 'grey information' in public health: critical reflections on three case studies. *Syst Rev* 2016;5(1):164. doi: 10.1186/s13643-016-0337-y

61. Mahmood Q, Van Eerd D, Irvin E. Searching for grey literature for systematic reviews: challenges and benefits. *Res Synth Method* 2013;5(3):221-34.

62. Adams RJ, Smart P, Huff AS. Shades of grey: guidelines for working with the grey literature in systematic reviews for management and organizational studies. *Int J Manag Rev* 2017;19(4):432-54. doi: 10.1111/ijmr.12102

63. Pearson A, Robertson-Malt S, Rittenmeyer L. Synthesizing Qualitative Evidence. Philadelphia: Lippincott-Joanna Briggs Institute 2011.

64. Joanna Briggs Institute. JBI QARI Critical Appraisal Checklist for Interpretive and Critical Research https://wiki.joannabriggs.org/display/MANUAL/Joanna+Briggs+Institute+Reviewer%27s+Manual: (accessed 08-06-18); [cited included reference from Health FGM 170518.enl. Available from: http://joannabriggs.org/assets/docs/jbc/operations/criticalAppraisalForms/JBC_Form_CritAp_IntCrit.pdf.

For peer review only

65. Munthe-Kaas H, Bohren MA, Glenton C, et al. Applying GRADE-CERQual to qualitative evidence synthesis findings—paper 3: how to assess methodological limitations. *Implement Sci* 2018;13(1):9. doi: 10.1186/s13012-017-0690-9

66. Lewin S, Glenton C, Munthe-Kaas H, et al. Using qualitative evidence in decision making for health and social interventions: an approach to assess confidence in findings from qualitative evidence syntheses (GRADE-CERQUAL). *PLoS Med* 2015;12(10):e1001895.

67. Dixon-Woods M, Sutton A, Shaw R, et al. Appraising qualitative research for inclusion in systematic reviews: a quantitative and qualitative comparison of three methods. *J Health Serv Res Policy* 2007;12 doi: 10.1258/135581907779497486

68. Dixon-Woods M, Booth A, Sutton AJ. Synthesizing qualitative research: a review of published reports. *Qual Res* 2007;7 doi: 10.1177/1468794107078517

69. Dixon-Woods M, Shaw RL, Agarwal S, et al. The problem of appraising qualitative research. *Qual Saf Health Care* 2004;13 doi: 10.1136/qshc.2003.008714

70. Sandelowski M, Barroso J. Reading qualitative studies. *Int J Qual Methods* 2002;1:74-108.

71. Noyes J, Booth A, Flemming K, et al. Cochrane Qualitative and Implementation Methods Group guidance paper 3: methods for assessing methodological limitations, data extraction and synthesis, and confidence in synthesized qualitative findings. *J Clin Epidemiol* 2017 doi: 10.1016/j.jclinepi.2017.06.020

72. Higginbottom G, Morgan M, Alexandre M, et al. Immigrant women's experiences of maternity-care services in Canada: a systematic review using a narrative synthesis. *Syst Rev* 2015;4(1):1-13.

73. Higginbottom GMA, Hadziabdic E, Yohani S, et al. Immigrant women's experience of maternity services in Canada: a meta-ethnography. *Midwifery* 2014;30(5):544-59. doi: 10.1016/j.midw.2013.06.004

74. Popay J, Rogers A, Williams G. Rationale and standards for the systematic review of qualitative literature in health services research. *Qual Health Res* 1998;8 doi: 10.1177/104973239800800305

75. Noyes J, Popay J. Directly observed therapy and tuberculosis: how can a systematic review of qualitative research contribute to improving services? a qualitative meta-synthesis. *J Adv Nurs* 2007;57(227-216)

76. Sandelowski M, Barroso J. Finding the findings in qualitative studies. *J Nurs Scholarsh* 2002;34:213-19.

77. Thomas J, Harden A. Methods for the thematic synthesis of qualitative research in systematic reviews. *BMC Med Res Methodol* 2009;8:45.

For peer review only

78. Boyatzis R. Transforming Qualitative Data: Thematic Analysis and Code Development. London: Sage 1998.

79. Braun V, Clarke V. Using thematic analysis in psychology. *Qual Res Psychol* 2006;3(2):77-101.

80. Booth A, Carroll C, Ilott I, et al. Desperately seeking dissonance: identifying the disconfirming case in qualitative evidence synthesis. *Qual Health Res* 2013;23(1):126-41.

81. Noyes J, Booth A, Lewin S, et al. Applying GRADE-CERQual to qualitative evidence synthesis findings–paper 6: how to assess relevance of the data. *Implement Sci* 2018;13(1):4. doi: 10.1186/s13012-017-0693-6

82. Noyes J, Booth A, Flemming K, et al. Cochrane Qualitative and Implementation Methods Group guidance series-paper 3: methods for assessing methodological limitations, data extraction and synthesis, and confidence in synthesized qualitative findings. *J Clin Epidemiol* 2018;97:49-58. doi: 10.1016/j.jclinepi.2017.06.020

83. Lewin S, Booth A, Glenton C, et al. Applying GRADE-CERQual to qualitative evidence synthesis findings: introduction to the series. *Implement Sci* 2018;13(1):2. doi: 10.1186/s13012-017-0688-3

84. Lewin S, Bohren M, Rashidian A, et al. Applying GRADE-CERQual to qualitative evidence synthesis findings—paper 2: how to make an overall CERQual assessment of confidence and create a Summary of Qualitative Findings table. *Implement Sci* 2018;13(1):10. doi: 10.1186/s13012-017-0689-2

85. Glenton C, Carlsen B, Lewin S, et al. Applying GRADE-CERQual to qualitative evidence synthesis findings—paper 5: how to assess adequacy of data. *Implement Sci* 2018;13(1):14. doi: 10.1186/s13012-017-0692-7

86. Colvin CJ, Garside R, Wainwright M, et al. Applying GRADE-CERQual to qualitative evidence synthesis findings—paper 4: how to assess coherence. *Implement Sci* 2018;13(1):13. doi: 10.1186/s13012-017-0691-8

87. Booth A, Lewin S, Glenton C, et al. Applying GRADE-CERQual to qualitative evidence synthesis findings–paper 7: understanding the potential impacts of dissemination bias. *Implement Sci* 2018;13(1):12. doi: 10.1186/s13012-017-0694-5

88. Asefaw F. [Female Genital Mutilation: A Field Study with Special Consideration of the Background as well as the Health and Psychosexual Consquences for those Affected and their Partners in Eritrea and in Germany] Weibliche Genitalbeschneidung: Eine Feldstudie Unter besonderer Berücksichtigung der Hintergründe Sowie der Gesundheitlichen und Psychosexuellen Folgen für Betroffene und Partner in Eritrea und Deutschland. Humboldt University of Berlin, 2007.

For peer review only

89. Maier C. [Echoes of Silence: Voices of Concern for Genital Mutilation in African Immigrant Women in Vienna: An Ethnological Study] Echo des Schweigens: Stimmen der Betroffenheit zur Genitalverstümmelung bei Afrikanischen Immigrantinnen in Wien: Ethnologische Studie. University of Vienna, 2003.

90. Ballesteros Meseguer C, Almansa Martinez P, Pastor Bravo MdM, et al. [The voice of women subjected to female genital mutilation in the region of Murcia, Spain] La Voz de las mujeres sometidas a mutilacion genital femenina en la region de Murcia. *Gac Sanit* 2014;28(4):287-91. doi: 10.1016/j.gaceta.2014.02.006

91. Bravo Pastor del Mar M. [Women Subjected to Female Genital Mutilation: Knowledge for the Nursing Discipline] La Voz de las Mujeres Sometidas a Mutilación Genital Femenina: Saberes Para la Disciplina Enfermera [Tesis Doctoral]. University of Murcia, 2014.

92. Abdi R. Carving culture: Creating identity through female genital cutting. *Durham Anthropology Journal* 2012;18(1):115-53.

93. Abdullahi A, Copping J, Kessel A, et al. Cervical screening: perceptions and barriers to uptake among Somali women in Camden. *Public Health* 2009;123(10):680-5. doi: 10.1016/j.puhe.2009.09.011

94. Ahmed M. Attitudes towards FGM among Somali women living in the UK. In: Momoh C, ed. Female Genital Mutilation. London: Radcliffe 2005:95-117.

95. Ameresekere M, Borg R, Frederick J, et al. Somali immigrant women's perceptions of cesarean delivery and patient-provider communication surrounding female circumcision and childbirth in the USA. *Int J Gynecol Obstet* 2011;115(3):227-30. doi: 10.1016/j.ijgo.2011.07.019

96. Baldeh F. Obstetric Care in Scotland: The Experience of Women who have Undergone Female Genital Mutilation (FGM) [MSc Thesis]. Queen Margaret University, 2013.

97. Behrendt A. Listening to African Voices: Female Genital Mutilation/Cutting among Immigrants in Hamburg: Knowledge, Attitudes and Practice. Hamburg, Germany: Plan, 2011.

98. Beine K, Fullerton J, Palinkas L, et al. Conceptions of prenatal care among Somali women in San Diego. *J Nurs Midwifery* 1995;40(4):376-81. doi: Doi 10.1016/0091-2182(95)00024-E

99. Berggren V, Bergstrom S, Edberg A-K. Being different and vulnerable: experiences of immigrant African women who have been circumcised and sought maternity care in Sweden. *J Transcult Nurs* 2006;17(1):50-7. doi: 10.1177/1043659605281981

100. Betts V. Transnational Gender, Sexuality and Identity Construction: Ambivalent Subjectivities of Somali Refugee Women in the GTA. MA Thesis, The University of Guelph, 2011.

For peer review only

101. Bulman KH, McCourt C. Somali refugee women's experiences of maternity care in west London: a case study. *Crit Public Health* 2002;12(4):365-80.

102. Carroll J, Epstein R, Fiscella K, et al. Caring for Somali women: implications for clinician-patient communication. *Patient Educ Couns* 2007;66(3):337-45. doi: 10.1016/j.pec.2007.01.008

103. Chalmers B, Omer-Hashi K. What Somali women say about giving birth in Canada. *J Reprod Infant Psychol* 2002;20(4):267-82. doi: 10.1080/0264683021000033183

104. Degni F, Suominen SB, El Ansari W, et al. Reproductive and maternity health care services in Finland: perceptions and experiences of Somali-born immigrant women. *Ethn Health* 2014;19(3):348-66. doi: 10.1080/13557858.2013.797567

105. d'Entremont M, Smythe L, McAra-Couper J. The sounds of silence: a hermeneutic interpretation of childbirth post excision. *Health Care Women Int* 2014;35(3):300-19. doi: 10.1080/07399332.2013.838245

106. Essén B, Johnsdotter S, Hovelius B, et al. Qualitative study of pregnancy and childbirth experiences in Somalian women resident in Sweden. *Br J Obstet Gynaecol* 2000;107(12):1507-12.

107. Fawcett L. Somali Refugee Women and their U.S. Healthcare Providers: Knowledge, Perceptions and Experiences of Childbearing [Doctor of Philosophy]. Arizona State University, 2014.

108. Gali MA. Female Circumcision: A Transcultural Study of Attitudes, Identity and Reproductive Health of East African Immigrants [Degree of Doctor of Philosophy in Psychology]. The Wright Institute, University of Berkeley, 1997.

109. Ghebre RG, Sewali B, Osman S, et al. Cervical cancer: barriers to screening in the Somali community in Minnesota. *J Immigr Minor Health* 2015;17(3):722-28. doi: 10.1007/s10903-014-0080-1

110. Glazer E. Embodiment, Pain and Circumcision in Somali-Canadian Women [M.Sc.]. University of Toronto, 2012.

111. Glover J, Liebling H, Barrett H, et al. The psychological and social impact of female genital mutilation: a holistic conceptual framework. *J Int Stud* 2017;10(2):219-38.

112. Guerin PB, Allotey P, Elmi FH, et al. Advocacy as a means to an end: assisting refugee women to take control of their reproductive health needs. *Women Health* 2006;43(4):7-25.

113. Hill N, Hunt E, Hyrkas K. Somali immigrant women's health care experiences and beliefs regarding pregnancy and birth in the United States. *J Transcult Nurs* 2012;23(1):72-81. doi: 10.1177/1043659611423828 [published Online First: 2011/11/05]

For peer review only

114. Hussein E. Women's Experiences, Perceptions and Attitudes of Female Genital Mutilation: The Bristol PEER Study. London: FORWARD, 2010.

115. Hussen MA. Services for Women with Female Genital Mutilation in Christchurch: Perspectives of Women and their Health Providers. University of Canterbury, 2014.

116. Johansen RE. Undoing female genital cutting: perceptions and experiences of infibulation, defibulation and virginity among Somali and Sudanese migrants in Norway. *Cult Health Sex* 2017;19(4):528-42. doi: 10.1080/13691058.2016.1239838

117. Jones A. Working Psychologically with Female Genital Mutilation: An Exploration of the Views and Experiences of Women who have Experienced FGM and of Clinical Psychologists. University of East London, 2010.

118. Khaja K. Female Circumcision: Life Histories of Somali Women [Doctor of Philosophy]. The University of Utah, 2004.

119. Khaja K, Lay K, Boys S. Female circumcision: toward an inclusive practice of care. *Health Care Women Int* 2010;31(8):686-99. doi: 10.1080/07399332.2010.490313

120. Lundberg PC, Gerezgiher A. Experiences from pregnancy and childbirth related to female genital mutilation among Eritrean immigrant women in Sweden. *Midwifery* 2008;24(2):214-25. doi: 10.1016/j.midw.2006.10.003

121. Maternity Action. Women's Voices on Health: Addressing Barriers to Accessing Primary Care. London: Women’s Health and Equality Consortium, 2014.

122. McNeely S, Christie-de Jong F. Somali refugees' perspectives regarding FGM/C in the US. *Int J Migr Health Soc Care* 2016;12(3):157-69. doi: 10.1108/Ijmhsc-09-2015-0033

123. Moxey JM, Jones LL. A qualitative study exploring how Somali women exposed to female genital mutilation experience and perceive antenatal and intrapartum care in England. *BMJ Open* 2016;6(1):e009846. doi: 10.1136/bmjopen-2015-009846

124. Murray L, Windsor C, Parker E, et al. The experiences of African women giving birth in Brisbane, Australia. *Health Care Women Int* 2010;31(5):458-72. doi: 10.1080/07399330903548928

125. Norman K, Gegzabher SB, Otoo-Oyortey N. "Between Two Cultures": A Rapid PEER Study Exploring Migrant Communities' Views on Female Genital Mutilation in Essex and Norfolk, UK. FORWARD & National FGM Centre Report, , 2016.

126. Norman K, Hemmings J, Hussein E, et al. "FGM is Always With Us": Experiences, Perceptions and Beliefs of Women Affected by Female Genital Mutilation in London: Results from a PEER Study. London: Options Consultancy Services and FORWARD, 2009.

For peer review only

127. O'Brien O, Baldeh F, Hassan J, et al. My Voice: Participatory Action Research Project with Men, Women and Young People on Female Genital Mutilation (FGM) in Scotland: (Phase 2). Waverly Care, Edinburgh: Queen Margaret University, 2017.

128. O'Brien O, Baldeh F, Sivapatham S, et al. Participatory Action Research Project with Men, Women and Young People on Female Genital Mutilation (FGM) in Scotland: (Phase 1). Waverly Care, Edinburgh: Queen Margaret University, 2016.

129. Palfreyman A, Brown E, Nam S. Understanding Female Genital Mutilation in Birmingham: Findings from a PEER Study: Options Consultancy Services and Birmingham & Solihull Women's Aid, 2011.

130. Safari F. A qualitative study of women's lived experience after deinfibulation in the UK. *Midwifery* 2013;29(2):154-8. doi: 10.1016/j.midw.2011.12.005

131. Salad J, Verdonk P, de Boer F, et al. "A Somali girl is Muslim and does not have premarital sex - is vaccination really necessary?" A qualitative study into the perceptions of Somali women in the Netherlands about the prevention of cervical cancer. *Int J Equity Health* 2015;14(1):1-13. doi: 10.1186/s12939-015-0198-3

132. Shaw E. Female circumcision: perceptions of clients and caregivers. *J Am Coll Health* 1985;33(5):193-7. doi: 10.1080/07448481.1985.9939604

133. Shermarke MAA. Understanding the Canadian Community Context of Female Circumcision [M.S.W.]. McGill University, 1996.

134. Straus L, McEwen A, Hussein FM. Somali women's experience of childbirth in the UK: perspectives from Somali health workers. *Midwifery* 2009;25(2):181-6. doi: 10.1016/j.midw.2007.02.002

135. Thierfelder C. Female Genital Mutilation and the Swiss Health Care System. University of Basel, 2003.

136. Thierfelder C, Tanner M, Bodiang CMK. Female genital mutilation in the context of migration: experience of African women with the Swiss health care system. *Eur J Public Health* 2005;15(1):86-90. doi: 10.1093/eurpub/cki120

137. Upvall MJ, Mohammed K, Dodge PD. Perspectives of Somali Bantu refugee women living with circumcision in the United States: a focus group approach. *Int J Nurs Stud* 2009;46(3):360-8. doi: 10.1016/j.ijnurstu.2008.04.009

138. Vangen S, Johansen REB, Sundby J, et al. Qualitative study of perinatal care experiences among Somali women and local health care professionals in Norway. *Eur J Obstet Gynecol Reprod Biol* 2004;112(1):29-35. doi: 10.1016/S0301-2115(03)00313-0

139. Vaughan C, White N, Keogh L, et al. Listening to North Yarra Communities about Female Genital Cutting. Melbourne, Australia: The University of Melbourne, 2014.

For peer review only

140. Vaughan C, White N, Keogh L, et al. Female Genital Mutilation/Cutting in Regional Victoria. Research to Practice. Melbourne, Australia: The University of Melbourne, 2014.

141. Vloeberghs E, van der Kwaak A, Knipscheer J, et al. Coping and chronic psychosocial consequences of female genital mutilation in the Netherlands. *Ethn Health* 2012;17(6):677-95. doi: 10.1080/13557858.2013.771148

142. Wiklund H, Aden AS, Högberg U, et al. Somalis giving birth in Sweden: a challenge to culture and gender specific values and behaviours. *Midwifery* 2000;16(2):105-15. doi: http://dx.doi.org/10.1054/midw.1999.0197

143. Lincoln Y, Guba E. Naturalistic Inquiry. California: Sage 1985.

144. Essen B, Binder P, Johnsdotter S. An anthropological analysis of the perspectives of Somali women in the West and their obstetric care providers on caesarean birth. *J Psychosom Obst Gyn* 2011;32(1):10-18. doi: 10.3109/0167482x.2010.547966

145. Green G, Davison C, Bradby H, et al. Pathways to care: how superdiversity shapes the need for navigational assistance. *Sociol Health Illn* 2014;36:1205-19.

146. Phillimore J, Klaas F, Padilla B, et al. Adaptation of Health Services to Diversity: An Overview of Approaches, Working Paper No.15. University of Birmingham: Institute for Research into Superdiversity, 2016.

147. Benza S, Liamputtong P. Pregnancy, childbirth and motherhood: a meta-synthesis of the lived experiences of immigrant women. *Midwifery* 2014;30(6):575-84.

148. Wikberg A, Bondas T. A patient perspective in research on intercultural caring in maternity care: a meta-ethnography. *Int J Qual Stud Health Well-Being* 2010;5(1):15. doi: 10.3402/qhw.v5i1.4648

149. Balaam M-C, Akerjordet K, Lyberg A, et al. A qualitative review of migrant women's perceptions of their needs and experiences related to pregnancy and childbirth. *J Adv Nurs* 2013;69(9):1919-30. doi: 10.1111/jan.12139

150. Higginbottom GMA, Safipour J, Yohani S, et al. An ethnographic study of communication challenges in maternity care for immigrant women in rural Alberta. *Midwifery* 2015;31(2):297-304. doi: 10.1016/j.midw.2014.09.009

151. Bohren MA, Vogel JP, Hunter EC, et al. The mistreatment of women during childbirth in health facilities globally: a mixed-methods systematic review. *PLoS Med* 2015;12(6):e1001847. doi: 10.1371/journal.pmed.1001847

152. Department of Health. Midwifery 2020: Delivering Expectations. London, , 2010.

153. NHS England. National Maternity Review. Better Births: Improving Outcomes of Maternity Services in England. London, , 2016.

For peer review only

154. Mainous A, Baker R, Love M. Continuity of care and trust in one's physician: evidence from primary care in the United States and the United Kingdom. *Fam Med* 2001;33:22-27.

155. Gilson L. Trust and the development of health care as an institution. *Soc Sci Med* 2003;56:1458-68.

156. Murray B, McCrone S. An integrative review of promoting trust in patient-primary care provider relationships. *J Adv Nurs* 2015;71:3-23.

157. Denny E. Respectful maternity care needs to be the standard for all women worldwide (mini-commentary). *Br J Obstet Gynaecol* 2017;Dec 12 doi: 10.1111/1471-0528.15055

158. Shakibazadeh E, Namadian M, Bohren M, et al. Respectful care during childbirth in health facilities globally: a qualitative evidence synthesis. *Br J Obstet Gynaecol* 2017 doi: https://doi.org/10.1111/1471-0528.15015

159. Martin M, Phelan M. Interpreters and cultural mediators: different but complementary roles. *Migr Health* 2010;6(1):1-8.

160. Akhavan S, Edge D. Foreign born women's experiences of community based Doulas in Sweden: a qualitative study. *Health Care Women Int* 2012;33(9):833-48. doi: http://dx.doi.org/10.1080/07399332.2011.646107

161. Akhavan S, Lundgren I. Midwives' experiences of doula support for immigrant women in Sweden: a qualitative study. *Midwifery* 2012;28(1):80-85. doi: 10.1016/j.midw.2010.11.004

162. Dundek LH. Establishment of a Somali doula program at a large metropolitan hospital. *J Perinat Neonatal Nurs* 2006;20(2):128-37.

163. Shelp A. Women helping women: the Somali doula initiative. *Int J Childbirth Educ* 2004;19(4):4-7.

164. Evans C, Nalubega S, McLuskey J, et al. The views and experiences of nurses and midwives in the provision and management of provider-initiated HIV testing and counseling: a systematic review of qualitative evidence. *JBI Database System Rev Implement Rep* 2016;13:130-286.

165. WHO. Service Delivery Approaches to HIV Testing and Counselling (HTC): A Strategic Programme Framework. Geneva: World Health Organisation, 2012.

166. Hamberger LK, Rhodes K, Brown J. Screening and intervention for intimate partner violence in healthcare settings: creating sustainable system-level programs. *J Womens Health* 2015;24(1):86-91. doi: 10.1089/jwh.2014.4861

167. O'Doherty L, Hegarty K, Ramsay J, et al. Screening women for intimate partner violence in healthcare settings. *Cochrane Database Syst Rev* 2015;7(CD007007)

For peer review only

168. Karliner L, Jacobs E, Chen A. Do professional interpreters improve clinical care for patients with limited English proficiency? a systematic review of the literature. *Health Serv Res* 2007;42:727-54.

169. Saha S, Beach M, Cooper L. Patient centredness, cultural competence and healthcare quality. *J Natl Med Assoc* 2008;100:1275-85.

170. Abdulcadir J, Rodriguez MI, Say L. A systematic review of the evidence on clitoral reconstruction after female genital mutilation/cutting. *Int J Gynaecol Obstet* 2015;129(2):93-97.

171. De Schrijver L, Leye E, Merckx M. A multidisciplinary approach to clitoral reconstruction after female genital mutilation: the crucial role of counselling. *Eur J Contracept Reprod Health Care* 2016;21(4):269-75. doi: http://dx.doi.org/10.3109/13625187.2016.1172063

172. Dominguez Garcia L, Dominguez L, Hopkins K, et al. Meeting the sexual health needs of women with female genital mutilation: an integrated model of care. *Int J STD AIDS* 2013;24:2.

173. Low-Beer N, Dominguez-Garcia L, Alcayde D, et al. Accessible integrated care to meet the complex needs of women with female genital mutilation (FGM). *Br J Obstet Gynaecol* 2013;120:314.

174. Johansen RE. Pain as a counterpoint to culture: toward an analysis of pain associated with infibulation among Somali immigrants in Norway. *Med Anthropol Q* 2002;16(3):312-40.

175. Johansen RE. Care for infibulated women giving birth in Norway: an anthropological analysis of health workers' management of a medically and culturally unfamiliar issue. *Med Anthropol Q* 2006;20(4):516-44.

176. James S. Women's experiences of symptoms of post-traumatic stress disorder (PTSD) after traumatic childbirth: a review and critical appraisal. *Arch Womens Ment Health* 2015;18(6):216-26.

177. Raja S, Hasnain M, Hoersch M, et al. Trauma informed care in medicine: current knowledge and future research directions. *Fam Community Health* 2015;38(3):216-26.

178. Abdulcadir J, Bianchi Demicheli F, Willame A, et al. Posttraumatic stress disorder relapse and clitoral reconstruction after female genital mutilation. *Int J Gynaecol Obstet* 2017;129(2):371-76.

179. Abayomi O, Chibuzor MT, Okusanya BO, et al. Supportive psychotherapy or client education alongside surgical procedures to correct complications of female genital mutilation: A systematic review. *Int J Gynaecol Obstet* 2017;136 Suppl 1:51-55.

180. Adelufosi A, Edet B, Arikpo D, et al. Cognitive behavioral therapy for post-traumatic stress disorder, depression, or anxiety disorders in women and girls living with female genital mutilation: a systematic review. *Int J Gynaecol Obstet* 2017;136 Suppl 1:56-59.

For peer review only

181. Jones A, Byrne A. Working psychologically with female genital mutilation: an exploration of the views and experiences of women who have experienced FGM and of clinical psychologists. *Clin Psychol Forum* 2013(246):26-30.

182. Clayton-Hathway K. A Pilot Evaluation of Health Services for Communities Affected by FGM in Oxfordshire: Final Report. Oxford, UK: Healthwatch Oxfordshire, 2016.

183. Gangoli G, Gill A, Mulvihill N, et al. Perception and barriers: reporting female genital mutilation. *Journal of Aggression, Conflict and Peace Research* 2018 doi: 10.1108/JACPR-09-2017-0323

184. Malik Y, Rowland A, Gerry F, et al. Mandatory reporting of female genital mutilation in children in the UK. *British Journal of Midwifery* 2018;26(6):377-86.

For peer review only

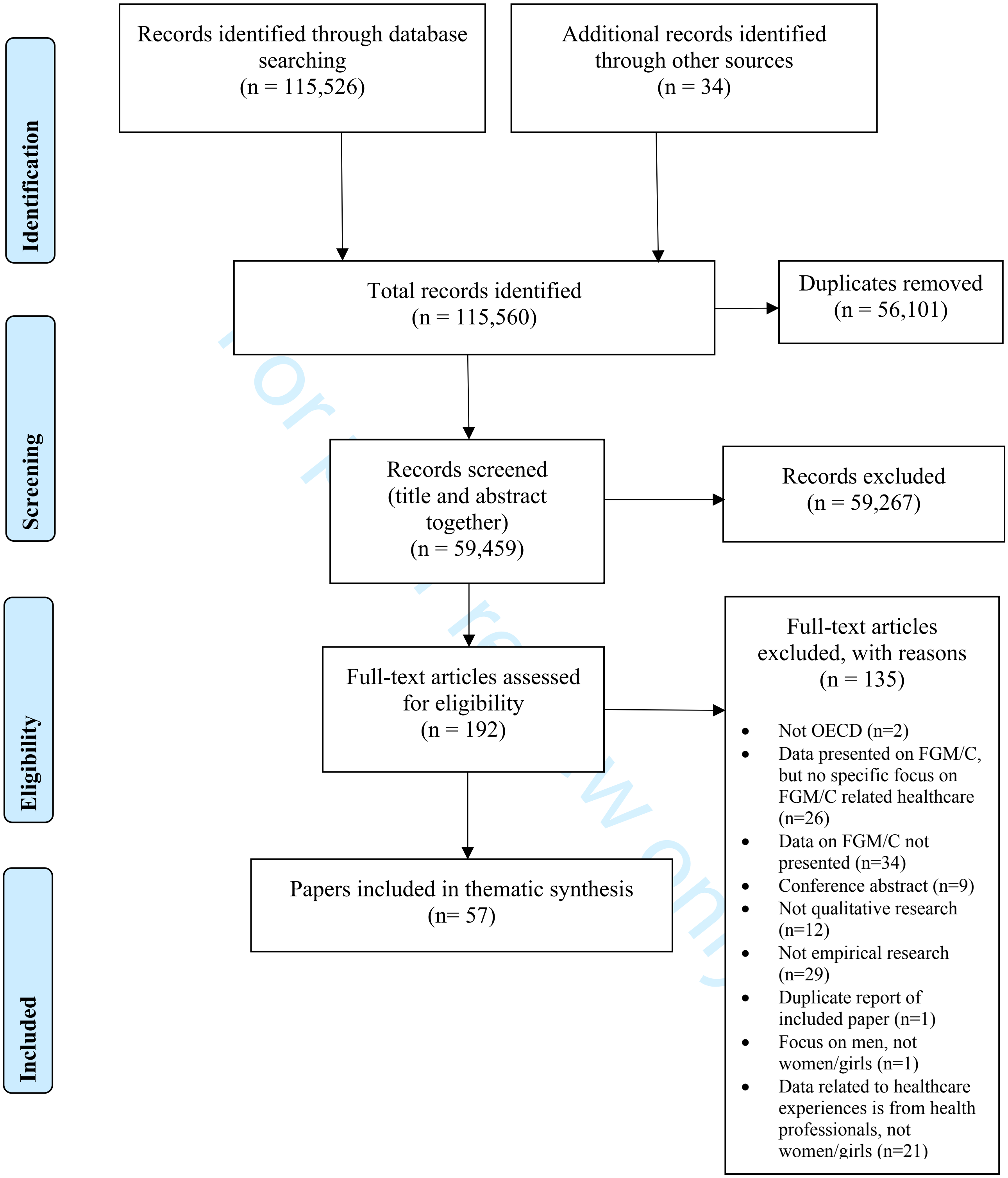
Identification
Screening
Eligibility
Included
Records identified through database searching (n = 115,526)
Additional records identified through other sources (n = 34)
Total records identified (n = 115,560)
Duplicates removed (n = 56,101)
Records screened (title and abstract together) (n = 59,459)
Records excluded (n = 59,267)
Full-text articles assessed for eligibility (n = 192)
Full-text articles excluded, with reasons (n = 135)
Not OECD (n=2)
Data presented on FGM/C, but no specific focus on FGM/C related healthcare (n=26)
Data on FGM/C not presented (n=34)
Conference abstract (n=9)
Not qualitative research (n=12)
Not empirical research (n=29)
Duplicate report of included paper (n=1)
Focus on men, not women/girls (n=1)
Data related to healthcare experiences is from health professionals, not women/girls (n=21)
Papers included in thematic synthesis (n= 57)

For peer review only

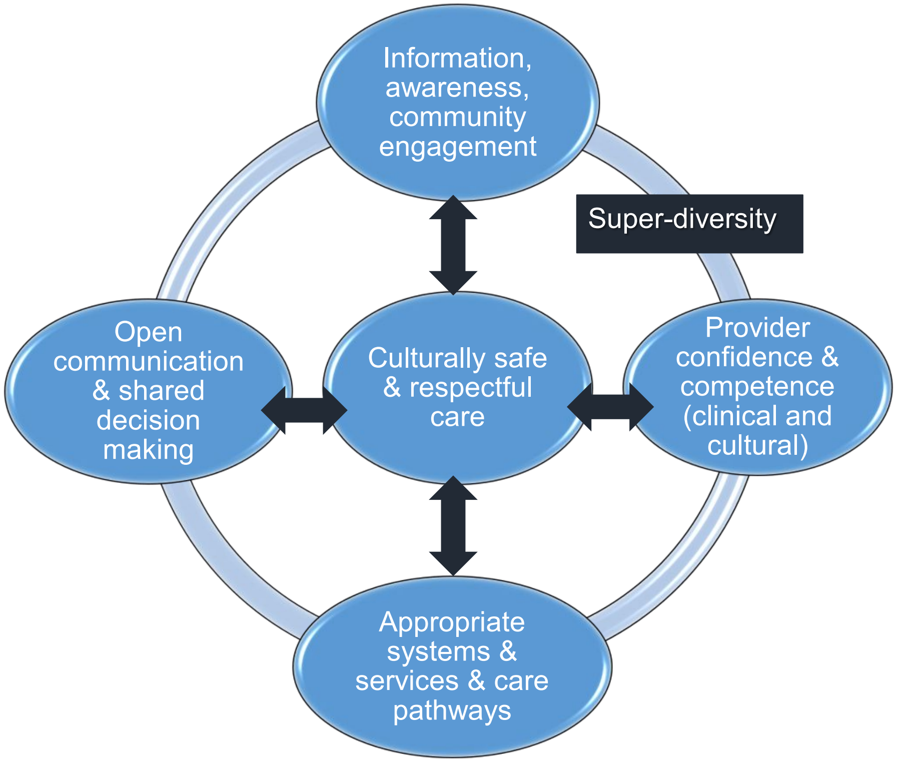
Information, awareness, community engagement
Super-diversity
Open communication & shared decision making
Culturally safe & respectful care
Provider confidence & competence (clinical and cultural)
Appropriate systems & services & care pathways

Supplementary File 1: Medline Search Strategy

Ovid Medline In-Process & Other Non-indexed Citations and Ovid Medline 1946 to present – run on 04/08/2017

Search statement	Search terms	Hits	Notes on strategy
1	exp Circumcision, Female/ or (exp Genitalia, Female/ and exp Medicine, Traditional/)	1367	
2	("female genital mutilation" or "female circumcision" or "female genital cutting" or "traditional female genital surgery" or infibulat* or reinfibulat* or defibulat* or clitoridect*).mp.	1509	
3	((((ritual* or traditional* or ceremon* or sociali#ation*) adj3 (practic* or cut or cutting or surg*)) or circumcis* or excis*) adj3 (female* or wom#n or girl* or child* or adolescen*)).mp.	2924	
4	(FGM or FGC or TFGS or FGMC or "FGM/C").mp.	1133	
5	(thara or tahar or khitan or khifad or khafad or megrez or absum or mekhnishab or kutairi or ibi or ugwu or sunna or bondo or sonde or gudiniin or halalays or qodiin or tahoor or bagne or gadja or ganza or "fanadu di mindjer" or niaka or kuyango or "musolula karoola").mp. not (thara or tahar or khitan or khifad or khafad or megrez or absum or mekhnishab or kutairi or ibi or ugwu or sunna or bondo or sonde or gudiniin or halalays or qodiin or tahoor or bagne or gadja or ganza or "fanadu di mindjer" or niaka or kuyango or "musolula karoola").au,in.	1650	Expanded terms to include country of origin terminology as described in Department of Health guidance.[121]
6	4 or 5	2779	
7	limit 6 to female	1200	In order to exclude irrelevant records from terminology from other disciplines, or male circumcision related hits
8	((FGM or FGC or TFGS or FGMC or "FGM/C" or thara or tahar or khitan or khifad or khafad or	507	Adjacency to “female” terms to

For peer review only

Search statement	Search terms	Hits	Notes on strategy
	megrez or absum or mekhnishab or kutairi or ibi or ugwu or sunna or bondo or sonde or gudiniin or halalays or qodiin or tahoor or bagne or gadja or ganza or "fanadu di mindjer" or niaka or kuyango or "musolula karoola") adj3 (female* or wom#n or girl* or child* or adolescen*)).mp.		complement "limit to female" hit set
9	(egypt* or sudan* or ethiopi* or somali* or djibouti* or eritrea* or burkin* or guinea* or sierra leone or leonean* or mauritan* or mali or malian*).ti,ab.	151913	Country of origin used as proxy for FGM/C terms – as FGM/C is so common, inference is that women from these countries will fit an FGM/C profile even without the term being explicitly mentioned
10	limit 9 to female	46657	
11	((egypt* or sudan* or ethiopi* or somali* or djibouti* or eritrea* or burkin* or guinea* or sierra leone or leonean* or mauritan* or mali or malian*) adj3 (female* or wom#n or girl* or child* or adolescen*)).mp.	5441	
12	1 or 2 or 3 or 7 or 8 or 10 or 11	51901	All women with FGM/C
13	"Emigrants and Immigrants"/ or Refugees/ or "Transients and Migrants"/ or "Emigration and Immigration"/	45756	
14	(immigrant* or immigrat* or emigrant* or emigrat* or emigre* or expat* or (ex adj pat*) or transients or newcomer* or (new adj comer*) or alien* or incomer* or (in adj comer*)).mp.	79630	
15	((immigrant* or immigrat* or emigrant* or emigrat* or emigre* or expat* or (ex adj pat*) or transients or newcomer* or (new adj comer*) or alien* or incomer* or (in adj comer*)) adj3 (female* or wom#n or girl* or child* or adolescen*)).mp.	4382	

For peer review only

Search statement	Search terms	Hits	Notes on strategy
16	(refugee* or (asylum adj seek*) or asylee* or (refused adj3 (asylum* or refugee*)) or (displaced adj person*) or exile* or (new adj arrival) or (country adj2 (birth or origin)) or transnational*).mp.	17539	
17	((refugee* or (asylum adj seek*) or asylee* or (refused adj3 (asylum* or refugee*)) or (displaced adj person*) or exile* or (new adj arrival) or (country adj2 (birth or origin)) or transnational*) adj3 (female* or wom#n or girl* or child* or adolescen*)).mp.	1462	
18	(foreigner* or (foreign adj (born or citizen* or national* or origin*)) or (non adj (citizen* or native*)) or ((adoptive or naturali#ed) adj (citizen* or resident*)) or overstay* or trafficked or "spousal migrant*").mp	11442	
19	((foreigner* or (foreign adj (born or citizen* or national* or origin*)) or (non adj (citizen* or native*)) or ((adoptive or naturali#ed) adj (citizen* or resident*)) or overstay* or trafficked or "spousal migrant*") adj3 (female* or wom#n or girl* or child* or adolescen*)).mp.	753	
20	(((established or long-term or "first generation*" or new* or recent* or current*) adj3 (migrant* or migrat* or immigrant* or immigrat* or emigrant* or emigrat* or emigre* or expat* or (ex adj pat*) or transient* or alien*)) or newcomer* or (new adj comer*) or incomer* or (in adj comer*) or ((international or overseas or foreign) adj2 (student* or employee* or worker*))).mp.	18239	
21	((((established or long-term or "first generation*" or new* or recent* or current*) adj3 (migrant* or migrat* or immigrant* or immigrat* or emigrant* or emigrat* or emigre* or expat* or (ex adj pat*) or transient* or alien*)) or newcomer* or (new adj comer*) or incomer* or (in adj comer*) or ((international or overseas or foreign) adj2 (student* or employee* or worker*))) adj3 (female* or wom#n or girl* or child* or adolescen*)).mp.	559	
22	("non-UK-born" or "born outside the UK" or "length of residence in the UK" or (("not	2887	

For peer review only

Search statement	Search terms	Hits	Notes on strategy
	lawful*" or "not legal*" or unlawful* or illegal* or unauthori#ed* or "not authori#ed" or uncertain or insecure or legal* or legitimate* or permit* or visa* or irregular* or refused or undocumented) adj3 (residen* or student* or worker* or employee* or unemployed or immigrant* or imigrat* or migrant* or migrat*))).mp.		
23	(("non-UK-born" or "born outside the UK" or "length of residence in the UK" or (("not lawful*" or "not legal*" or unlawful* or illegal* or unauthori#ed* or "not authori#ed" or uncertain or insecure or legal* or legitimate* or permit* or visa* or irregular* or refused or undocumented) adj3 (residen* or student* or worker* or employee* or unemployed or immigrant* or imigrat* or migrant* or migrat*))) adj3 (female* or wom#n or girl* or child* or adolescen*)).mp.	136	
24	exp Vulnerable Populations/	8275	
25	((vulnerab* or disadvantag* or minorit*) adj3 (individ* or person* or people* or population* or communit* or group*)).ti,ab.	33112	
26	((vulnerab* or disadvantag* or minorit*) adj3 (individ* or person* or people* or population* or communit* or group*) adj3 (female* or wom#n or girl* or child* or adolescen*)).mp.	1756	
27	exp Ethnic Groups/	139042	
28	(ethnic* or ethno* or race or racial*).mp.	248038	
29	("Black and Minority Ethnic" or "Black & Minority ethnic" or BME or BAME or black african*).mp.	3756	
30	((ethnic* or ethno* or race or racial* or "Black and Minority Ethnic" or "Black & Minority ethnic" or BME or BAME or black african*) adj3 (female* or wom#n or girl* or child* or adolescen*)).mp.	9504	
31	exp african continental ancestry group/ or exp asian continental ancestry group/	136483	
32	(africa* or "middle east*" or asia* or iran* or iraq* or israel* or oman* or "united arab	520081	

For peer review only

Search statement	Search terms	Hits	Notes on strategy
	emirat*" or UAE or "saudi arabia*" or palestin* or india* or indonesia* or malaysia* or pakistan*).ti,ab.		
33	(egypt* or yemen* or sudan* or ethiopi* or somali* or djibouti* or eritrea* or kenya* or uganda* or tanzania* or camero?n* or chad* or niger* or benin* or togo* or ghana* or burkin* or ivory coast* or cote d'ivoire or liberia* or guinea* or sierra leone or leonean* or gambia* or senegal* or mauritan* or mali or malian* or "sara subgroup" or ngama).ti,ab.	259558	
34	((africa* or "middle east*" or asia* or iran* or iraq* or israel* or oman* or "united arab emirat*" or UAE or "saudi arabia*" or palestin* or india* or indonesia* or malaysia* or pakistan* or egypt* or yemen* or sudan* or ethiopi* or somali* or djibouti* or eritrea* or kenya* or uganda* or tanzania* or camero?n* or chad* or niger* or benin* or togo* or ghana* or burkin* or ivory coast* or cote d'ivoire or liberia* or guinea* or sierra leone or leonean* or gambia* or senegal* or mauritan* or mali or malian* or "sara subgroup" or ngama) adj3 (female* or wom#n or girl* or child* or adolescen*)).mp.	58342	
35	13 or 14 or 16 or 18 or 20 or 22 or 24 or 25 or 27 or 28 or 29 or 31 or 32 or 33	1117829	
36	limit 35 to female	491179	Keywords for immigrant populations limited to female (Medline standard limit feature)
37	(13 or 24 or 27 or 31) and (female* or wom#n or girl* or child* or adolescen*).mp.	177101	MeSH Headings for immigrant populations limited by “female” keywords
38	15 or 17 or 19 or 21 or 23 or 26 or 30 or 34 or 36 or 37	526145	Terms for immigrant populations, including specific terms for

For peer review only

Search statement	Search terms	Hits	Notes on strategy
			countries of origin where FGM known to be practised, limited to female
39	exp Community Health Workers/ or exp Volunteers/	23517	All health and allied workers (who may come into contact with women with FGM)
40	exp Occupational Groups/	531564	
41	exp Health Personnel/	454091	
42	exp Midwifery/	17848	
43	exp General Practitioners/ or exp Physicians/	117502	
44	exp Nurses/ or exp Nurses, Community Health/	81947	
45	exp Nurses' Aides/ or exp Nursing Staff, Hospital/ or exp Nursing Staff/	66494	
46	exp Social Workers/ or exp Police/	4605	
47	39 or 40 or 41 or 42 or 43 or 44 or 45 or 46	562187	
48	exp Pregnancy/ or (pregnan* or birth* or childbirth* or matern* or gyn#e* or obstetric* or menstru* or labo#r* or vulv* or vagina* or uter*).ti,ab.	1391519	Physical complications or disorders as identified as health impacts of FGM in Department of Health guidance,[121] already "female" by context of terms – so proxy for FGM.
49	exp Pregnancy Complications/ or exp Pregnancy High Risk/	401801	
50	((pregnan* or birth* or childbirth* or matern* or gyn#e* or obstetric* or menstru* or labo#r* or vulv* or vagina* or uter*) adj3 (poor* or adverse or complicat* or difficult* or disorder* or dysfunction* or disease* or pain* or risk* or danger* or problem* or issue* or concern* or infect* or inflamm*)).ti,ab.	137220	
51	exp Obstetric Labor Complications/	63618	
52	exp Female Urogenital Diseases/	1161123	
53	exp Menstruation Disturbances/	27153	
54	exp Genital Diseases, Female/ or exp Vaginal Diseases/ or exp Vulvar Diseases/	432325	

For peer review only

Search statement	Search terms	Hits	Notes on strategy
55	exp Vaginismus/ or exp Vulvodynia/ or exp Dyspareunia/ or exp Vaginitis/ or exp Vulvovaginitis/	14171	
56	exp Pelvic Inflammatory Disease/	10694	
57	exp Vaginal Fistula/ or exp Rectovaginal Fistula/	4659	
58	exp Genital Diseases, Female/	432325	
59	exp Infertility, Female/	27170	
60	((pelvic* or back* or urolog* or urogenit* or urinat* or genit* or abdomin*) adj3 (poor* or adverse or complicat* or difficult* or disorder* or dysfunction* or disease* or pain* or risk* or danger* or problem* or issue* or concern* or infect* or inflamm*) adj3 (female* or wom#n or girl* or child* or adolescen*)).mp.	29027	
61	exp Urologic Diseases/	698515	Physical complications or disorders as identified as health impacts of FGM in Department of Health guidance,[121] limited to "female" (female genital diseases, etc.) – so proxy for FGM
62	exp Pelvic Pain/	8163	
63	exp Sexual Dysfunctions, Psychological/	24271	
64	exp Pelvic Infection/	5409	
65	exp Kidney Diseases/	474329	
66	exp Urinary Fistula/ or exp Rectal Fistula/ or exp Urinary Bladder Fistula/	11750	
67	61 or 62 or 63 or 64 or 65 or 66	737520	
68	limit 67 to female	352622	
69	67 and (female* or wom#n or girl* or child* or adolescen*).mp.	397916	
70	48 or 49 or 50 or 51 or 52 or 53 or 54 or 55 or 56 or 57 or 58 or 59 or 60 or 68 or 69	2386342	
71	exp Mental Disorders/	1122844	
72	exp Depressive Disorder, Major/ or exp Suicide/ or exp Depression/ or exp Suicidal Ideation/ or exp Depressive Disorder/ or exp Bipolar Disorder/ or exp Suicide, Attempted/	259964	

For peer review only

Search statement	Search terms	Hits	Notes on strategy
73	exp Self-Injurious Behavior/ or exp Substance-Related Disorders/ or exp Social Isolation/	329005	
74	exp Sexually Transmitted Diseases, Viral/ or exp Sexually Transmitted Diseases/	319070	
75	(anxiet* or anxious* or depress* or self-harm* or self-injur* or suicid*).mp.	650968	
76	((anxiet* or anxious* or depress* or self-harm* or self-injur* or suicid*) adj3 (female* or wom#n or girl* or child* or adolescen*)).mp.	30927	
77	(((sex* or viral* or virus* or bacteria*) adj3 diseas*) or HIV or STD* or AIDS).mp.	516543	
78	((((sex* or viral* or virus* or bacteria*) adj3 diseas*) or HIV or STD* or AIDS) adj3 (female* or wom#n or girl* or child* or adolescen*)).mp.	29597	
79	71 or 72 or 73 or 74 or 75 or 77	2107493	
80	limit 79 to female	932164	
81	79 and (female* or wom#n or girl* or child* or adolescen*).mp.	1098666	
82	76 or 78 or 80 or 81	1098666	Psychological or STD related impacts of FGM, as above
83	exp United Kingdom/ or ("united kingdom" or "great britain" or britain or england or scotland or wales or "northern ireland" or british or english or scottish or welsh or "northern irish" or hebrid* or "isle of man").ti,ab,gc,ia,pl,in.	1167455	
84	(AUSTRALIA* or AUSTRIA* or BELGIUM* or belgian* or CANAD* or CHILE* or CZECH* or DENMARK* or danish* or ESTONIA* or FINLAND or finnish or FRANCE or french).mp.	641227	
85	(GERMAN* or GREECE or greek* or HUNGAR* or ICELAND* or IRELAND or irish or ISRAEL* or ITALY or italian* or JAPAN* or KOREA* or LATVIA* or LUXEMB* or MEXIC*).mp.	791022	
86	(NETHERLAND* or holland* or dutch or "low countries" or europe* or "NEW ZEALAND*" or NORWAY or norwegian* or POLAND or polish or PORTUGAL or portuguese).mp.	597994	

For peer review only

Search statement	Search terms	Hits	Notes on strategy
87	(SLOVAK* or SLOVENIA* or SPAIN or spanish or SWED* or SWITZERLAND or swiss or TURK* or "UNITED KINGDOM" or "UK" or britain or british or england or english or scot* or wales or welsh or UNITED STATES or "USA" or america*).mp.	3770430	
88	83 or 84 or 85 or 86 or 87	5147200	UK or other OECD countries
89	randomized controlled trial.pt.	470445	
90	controlled clinical trial.pt.	94472	
91	randomized.ab.	403685	
92	placebo.ab.	189129	
93	clinical trials as topic.sh.	187621	
94	randomly.ab.	280498	
95	trial.ti.	181309	
96	(animals not (humans and animals)).sh.	4412254	
97	89 or 90 or 91 or 92 or 93 or 94 or 95	1138847	
98	97 not 96	1049209	Standard search hedge for randomised clinical trials in Medline – adapted from SIGN.[315]
99	38 and 47 and (70 or 82)	7952	Female immigrants AND Health Workers AND (physical or mental disorders)
100	47 and 12	1144	
101	12 or 99 or 100	59304	Women with FGM or (Female immigrants AND Health Workers AND (physical or

For peer review only

Search statement	Search terms	Hits	Notes on strategy
			mental disorders)) or (Health workers AND FGM)
102	101 and 88	12154	…AND OECD countries
103	102 not 98	11547	EXCLUDE RCTs
104	103 not 96	9261	EXCLUDE animal studies

For peer review only

Supplementary File 2: Excluded Studies (with Reasons)

No.	Study	Reason for Exclusion
1.	Abdel Halim AM. Honorable daughters: The lived experience of circumcised Sudanese women in the United States. PhD Thesis, Ann Arbor: Ohio University, 2003	Data presented on FGM/C, but no specific focus on FGM/C related healthcare
2.	Abdel Magied A and Shareef S. Knowledge, perception and attitudes of a sector of female health providers towards FGM - case study: female doctors. *Ahfad Journal*. 2003; 20: 4-17.	Not OECD
3.	Abdulcadir O, Catania L and Caselli A. Female genital mutilation (FGM): a comparison between the male and the female view. *Int J Gynaecol Obstet*. 2012; 119: S262-S3.	Conference abstract
4.	Alizadeh V, Hylander I, Kocturk T and Tornkvist L. Counselling young immigrant women worried about problems related to the protection of "family honour"-From the perspective of midwives and counsellors at youth health clinics. *Scand J Caring Sci*. 2010; 24: 32-40	Data presented on FGM/C, but no specific focus on FGM/C related healthcare
5.	Alizadeh V, Tornkvist L and Hylander I. Counselling teenage girls on problems related to the 'protection of family honour' from the perspective of school nurses and counsellors. *Health Soc Care Community*. 2011; 19: 476-84.	Data on FGM/C not presented
6.	Allag F, Abboud P, Mansour G, Zanardi M and Quereux C. Female genital mutilation. Women's point of view. *Gynecologie Obstetrique Fertilite*. 2001; 29: 824-8.	Data presented on FGM/C, but no specific focus on FGM/C related healthcare
7.	Aquino M, Edge D and Smith DM. Pregnancy as an ideal time for intervention to address the complex needs of black and minority ethnic women: Views of British midwives. *Midwifery*. 2015; 31: 373-9.	Data on FGM/C not presented
8.	Ariyo D, Ssali R, King-Webb L and Ikpaahindi S. Voices of the community: exploring female genital mutilation in the African community across Greater Manchester. London: AFRUCA, 2015.	Data presented on FGM/C, but no specific focus on FGM/C related healthcare
9.	Beck-Karrer C, Levin T and Levin T. Lion women. Conversations with Somalian women and men about female circumcision. *European Journal of Women's Studies*. 1998; 5: 533-6.	Not empirical research
10.	Bergqvist, H. & Svensson, J. (2016) [Midwives Experiences of Encounters wth Young Women who come from Areas Where the Practice of Genital Mutilation is Common] Barnmorskors Erfarenheter Av Möten Med Unga Kvinnor Som Kommer Från Områden	Data on healthcare-specific experiences is only from health professionals, not women/girls

For peer review only

No.	Study	Reason for Exclusion
	Där Kvinnlig Könsstympning är Aanligt Förekommande. University of Skövde, MSc Thesis, Sweden.	
11.	Bibi, N. & Rahimian, N. (2013) [Nurses' Experience and Knowledge about Female Genital Mutilation] Sjuksköterskans Erfarenheter Och Kunskaper Om Kvinnlig Könssympning. Sophiahemmet University, MSc Thesis, Sweden.	Data on healthcare-specific experiences is only from health professionals, not women/girls
12.	Brodin, E. & Mårtensson, N. (2016) [District Nurses' Knowledge and Experience of Female Genital Mutilation] Distriktssköterskors Kunskap Och Erfarenhet Av Kvinnlig Könsstympning. University of Örebro, MSc Thesis, Sweden.	Data on healthcare-specific experiences is only from health professionals, not women/girls
13.	Brown E, Carroll J, Fogarty C and Holt C. "They get a C-section . . . they gonna die": Somali women's fears of obstetrical interventions in the United States. *J Transcult Nurs*. 2010; 21: 220-7.	Data on FGM/C not presented
14.	Brown E and Porter C. The tackling FGM initiative: evaluation of the second phase (2013-2016). Options UK, Report. 2016.	Not empirical research
15.	Buckland RL. The everyday experience of Somali women in Canada: Implications for health. MSc Thesis, University of Ottawa (Canada), 1997	Data presented on FGM/C, but no specific focus on FGM/C related healthcare
16.	Bulman, K. & McCourt, C. (1997) Report on Somali Womens' Experiences of Maternity Services. Centre for Midwifery Practice, Wolfson Institute of Health Sciences, Thames Valley University and Hammersmith Hospitals National Health Service Trust, London, UK.	Data on healthcare-specific experiences is only from health professionals, not women/girls
17.	Burchill J. Safeguarding vulnerable families: work with refugees and asylum seekers. *Community Practitioner*. 2011; 84: 23-6.	Data presented on FGM/C, but no specific focus on FGM/C related healthcare
18.	Burchill, J. & Pevalin, D.J. (2014) Demonstrating cultural competence within health-visiting practice: working with refugee and asylum-seeking families. *Divers Equal Health Care,* 11(2), 151-159.	Data on healthcare-specific experiences is only from health professionals, not women/girls
19.	Byrskog, U., Olsson, P., Essen, B. & Allvin, M.K. (2015) Being a bridge: Swedish antenatal care midwives' encounters with Somali-born women and questions of violence - a qualitative study. *BMC Pregnancy Childbirth,* 15(1).	Data on healthcare-specific experiences is only from health professionals, not women/girls
20.	Carolan M. Antenatal care perceptions of pregnant African women attending maternity services in Melbourne, Australia. *Midwifery*. 2010; 26: 189-201.	Data on FGM/C not presented
21.	Chalmers B and Hashi KO. 432 Somali women's birth experiences in Canada after earlier female genital mutilation. *Birth*. 2000; 27: 227-34.	Not qualitative research

For peer review only

No.	Study	Reason for Exclusion
22.	Christiansen CD. The lived experience of circumcision in immigrant Somali women: a Heideggerian hermeneutic analysis. MA Thesis, University of Illinois, 1995,	Data presented on FGM/C, but no specific focus on FGM/C related healthcare
23.	Clarke ALF. The process of changed meanings: A study of the Canadian experience of Somali women in the Kitchener-Waterloo area in regard to female circumcision. PhD Thesis, Ann Arbor: Wilfrid Laurier University (Canada), 1995,	Not empirical research
24.	Clayton-Hathway K. A Pilot Evaluation of Health Services for Communities Affected by FGM in Oxfordshire: Final Report. Oxford, UK: Healthwatch Oxfordshire, 2016.	Not qualitative research
25.	Connor JJ, Hunt S, Finsaas M, Ciesinski A, Ahmed A and Robinson BBE. Sexual Health Care, Sexual Behaviors and Functioning, and Female Genital Cutting: Perspectives From Somali Women Living in the United States. *J Sex Res*. 2016; 53: 346-59.	Data presented on FGM/C, but no specific focus on FGM/C related healthcare
26.	Dahlen UM. Female Genital Cutting: Phenomenological Interviews on the Ethiopian Immigrant Mothers' Experience. Masters Thesis, Regent University, 2011,	Data presented on FGM/C, but no specific focus on FGM/C related healthcare
27.	Dawson, A.J., Turkmani, S., Varol, N., Nanayakkara, S., Sullivan, E. & Homer, C.S. (2015) Midwives' experiences of caring for women with female genital mutilation: insights and ways forward for practice in Australia. *Women Birth,* 28(3), 207-14.	Data on healthcare-specific experiences is only from health professionals, not women/girls
28.	Degni F, Suominen S, Essen B, El Ansari W and Vehvilainen-Julkunen K. Communication and cultural issues in providing reproductive health care to immigrant women: health care providers' experiences in meeting the needs of Somali women living in Finland. *Journal of Immigrant and Minority Health*. 2012; 14: 330-43.	Data on FGM/C not presented
29.	Essen B, Binder P and Johnsdotter S. An anthropological analysis of the perspectives of Somali women in the West and their obstetric care providers on caesarean birth. *Journal of Psychosomatic Obstetrics and Gynecology*. 2011; 32: 10-8.	Data on FGM/C not presented
30.	Essen B, Johnsdotter S and Binder P. Not too far to walk but too far for reciprocity: Maternal mortality in a migration context using the 'three delays' framework. *International Journal of Gynecology and Obstetrics*. 2012; 119: S343.	Conference abstract
31.	Feldman R. When maternity doesn't matter: Dispersing pregnant women seeking asylum. *British Journal of Midwifery*. 2014; 22: 23-8.	Data on FGM/C not presented
32.	Finnstrom B and Soderhamn O. Conceptions of pain among Somali women. *J Adv Nurs*. 2006; 54: 418-25.	Data presented on FGM/C, but no specific

For peer review only

No.	Study	Reason for Exclusion
		focus on FGM/C related healthcare
33.	García Aguado S and Sánchez López MI. Knowledge of healthcare professionals about female genital mutilation. *Metas de Enfermería*. 2013; 16: 18-22.	Not qualitative research
34.	Gele AA, Kumar B, Hjelde KH and Sundby J. Attitudes toward female circumcision among Somali immigrants in Oslo: a qualitative study. *International Journal of Women's Health*. 2012; 4: 7-17.	Data presented on FGM/C, but no specific focus on FGM/C related healthcare
35.	Gele AA, Sagbakken M and Kumar B. Is female circumcision evolving or dissolving in Norway? A qualitative study on attitudes toward the practice among young Somalis in the Oslo area. *International Journal of Women's Health*. 2015; 7: 933-43.	Data presented on FGM/C, but no specific focus on FGM/C related healthcare
36.	Gerrish K, Naisby A and Ismail M. Experiences of the diagnosis and management of tuberculosis: A focused ethnography of Somali patients and healthcare professionals in the UK. *J Adv Nurs*. 2013; 69: 2285-94.	Data on FGM/C not presented
37.	Gertsson, M. & Serpan, H. (2009) [Meeting with the Unthinkable: Value Conflicts in Meetings with Women Vulnerable to Female Genital Mutilation] Mötet Med Det Otänkbara: Värdekonflikter I Mötet Med Kvinnor Utsatta för Kvinnlig Könsstympning. University of Kalmar, MSc Thesis, Sweden.	Data on healthcare-specific experiences is only from health professionals, not women/girls
38.	Glavin K and Sæteren B. Cultural Diversity in Perinatal Care: Somali New Mothers' Experiences with Health Care in Norway. *Health Science Journal*. 2016; 10: 1-9.	Data on FGM/C not presented
39.	Glazer E. Gendering chronic pain: Socio-cultural embodiment and circumcision in Somali women in Toronto. *Gend Med*. 2010; 7: 528.	Conference abstract
40.	Goldblatt H. Caring for abused women: Impact on nurses' professional and personal life experiences. *J Adv Nurs*. 2009; 65: 1645-54.	Data on FGM/C not presented
41.	Hai DNT and Ghebre R. Comparison of barriers to cervical cancer screening among Somali and Vietnamese women. *J Womens Health*. 2013; 22: 36.	Conference abstract
42.	Henderson A. Factors influencing nurses' responses to abused women: What they say they do and why they say they do it. *Journal of Interpersonal Violence*. 2001; 16: 1284-306.	Data on FGM/C not presented
43.	Herrel N, Olevitch L, DuBois DK, et al. Somali refugee women speak out about their needs for care during pregnancy and delivery. *Journal of Midwifery & Women's Health*. 2004; 49: 345-9.	Data on FGM/C not presented
44.	Holm, L. & Kammensjö, H. (2012) [School Nurses' Experiences of Female Genital Mutilation among Girls]	Data on healthcare-specific experiences is

For peer review only

No.	Study	Reason for Exclusion
	Skolsköterskors Upplevelser Kring Kvinnlig Könsstympning Bland Flickor. University of Skövde, MSc Thesis, Sweden.	only from health professionals, not women/girls
45.	Houston AR. (In)visible embodiment: Somali perspectives of diabetes and mental health in diaspora. MSc Thesis, Boston University, 2016,	Data presented on FGM/C, but no specific focus on FGM/C related healthcare
46.	Hoyt MA. In her own words: An exploration of immigrant women's experiences of reproductive health care through ethnographic narrative. MA Thesis, University of New Brunswick (Canada), 2010,	Data on FGM/C not presented
47.	Isman E, Ekeus C and Berggren V. Perceptions and experiences of female genital mutilation after immigration to Sweden: an explorative study. *Sexual and Reproductive Healthcare*. 2013; 4: 93-8.	Data presented on FGM/C, but no specific focus on FGM/C related healthcare
48.	Jacoby SD, Lucarelli M, Musse F, Krishnamurthy A and Salyers V. A Mixed-Methods Study of Immigrant Somali Women's Health Literacy and Perinatal Experiences in Maine. *Journal of Midwifery and Women's Health*. 2015; 60: 593-603.	Data presented on FGM/C, but no specific focus on FGM/C related healthcare
49.	Jatau, M. (2011) Living Between Two Cultures: A Reproductive Health Journey of African Refugee Women. Arizona State University, PhD Thesis, USA.	Data on healthcare-specific experiences is only from health professionals, not women/girls
50.	Johansen REB. Experiencing sex in exile: can genitals change their gender? On conceptions and experiences related to female genital cutting (FGC) among Somalis in Norway. In: *Transcultural Bodies: Female Genital Cutting In Global Context Ed Ylva Hernlund & Bettina Shell-Duncan*. Rutgers University Press, 2007, p. 248-77.	Data presented on FGM/C, but no specific focus on FGM/C related healthcare
51.	Johansen, R.E. (2006) Care for infibulated women giving birth in Norway: an anthropological analysis of health workers' management of a medically and culturally unfamiliar issue. *Med. Anthropol. Q.,* 20(4), 516-44.	Data on healthcare-specific experiences is only from health professionals, not women/girls
52.	Johansen, R.E. (2017) Virility, pleasure and female genital mutilation/cutting: a qualitative study of perceptions and experiences of medicalized defibulation among Somali and Sudanese migrants in Norway. *Reprod Health,* 14(1), 25.	Data on healthcare-specific experiences is only from health professionals, not women/girls
53.	Johnsdotter S, Ingvarsdotter K, Ostman M and Carlbom A. Koran reading and negotiation with jinn: strategies to deal with mental ill health among Swedish Somalis. *Mental Health, Religion & Culture*. 2011; 14: 741-55.	Data on FGM/C not presented

For peer review only

No.	Study	Reason for Exclusion
54.	Johnsdotter S and Essén B. It is only a tradition: making sense of Swedish Somalis' narratives of female circumcision and avoiding submission to hegemonic political discourse. *Crit Soc Policy*. 2005; 25: 577-89.	Not empirical research
55.	Johnsdotter S and Essen B. Cultural change after migration: Circumcision of girls in Western migrant communities. *Best Practice and Research: Clinical Obstetrics and Gynaecology*. 2016; 32: 15-25.	Not empirical research
56.	Johnsdotter S, Moussa K, Carlbom A, Aregai R and Essen B. "Never my daughters": A qualitative study regarding attitude change toward female genital cutting among Ethiopian and Eritrean families in Sweden. *Health Care Women Int*. 2009; 30: 114-33.	Data presented on FGM/C, but no specific focus on FGM/C related healthcare
57.	Johnson-Agbakwu CE, Helm T, Killawi A and Padela AI. Perceptions of obstetrical interventions and female genital cutting: insights of men in a Somali refugee community. *Ethn Health*. 2014; 19: 440-5	Focus on men, not women
58.	Kallon I and Dundes L. The cultural context of the Sierra Leonean Mende woman as patient. *J Transcult Nurs*. 2010; 21: 228-36.	Not empirical research
59.	Kaplan-Marcusan A, Del Rio NF, Moreno-Navarro J, et al. Female genital mutilation: perceptions of healthcare professionals and the perspective of the migrant families. *BMC Public Health*. 2010; 10: 193.	Not qualitative research
60.	Kay M, Wijayanayaka S, Cook H and Hollingworth S. Understanding quality use of medicines in refugee communities in Australian primary care: A qualitative study. *Br J Gen Pract*. 2016; 66: e397-e409.	Data on FGM/C not presented
61.	Koukoui S, Hassan G and Guzder J. The mothering experience of women with FGM/C raising 'uncut' daughters, in Ivory Coast and in Canada. *Reproductive Health*. 2017; 14: 1-11.	Data presented on FGM/C, but no specific focus on FGM/C related healthcare
62.	Lazar J, Shipp M and Johnson C. Provider perceptions of sexual desire and dyspareunia among Somali women with female genital cutting. *J Sex Med*. 2010; 7: 148.	Conference abstract
63.	Lazar, J.N., Johnson-Agbakwu, C.E., Davis, O.I. & Shipp, M.P.L. (2013) Providers' perceptions of challenges in obstetrical care for Somali women. *Obstet. Gynecol. Int.,* 2013, 149640.	Data on healthcare-specific experiences is only from health professionals, not women/girls
64.	Lane J and Cole G. Pregnancy, prenatal care and delivery perceptions and beliefs of resettled African refugee women in the western United States. *Contraception*. 2013; 88: 313.	Conference abstract

For peer review only

No.	Study	Reason for Exclusion
65.	Leishman J. Perspectives of cultural competence in health care. *Nurs Stand*. 2004; 19: 33	Data on FGM/C not presented
66.	León-Larios, F. & Casado-Mejía, R. (2012) [Influence of gender on knowledge, perception and approach to harmful traditional practices: female genital mutilation]. *Evidentia,* 9(40), 1-7.	Data on healthcare-specific experiences is only from health professionals, not women/girls
67.	Lephard E and Haith-Cooper M. Pregnant and seeking asylum: Exploring women's experiences 'from booking to baby'. *British Journal of Midwifery*. 2016; 24: 130-6.	Data on FGM/C not presented
68.	Leval, A., Widmark, C., Tishelman, C. & Maina Ahlberg, B. (2004) The encounters that rupture the myth: contradictions in midwives' descriptions and explanations of circumcised women immigrants' sexuality. *Health Care Women Int.,* 25(8), 743-60.	Data on healthcare-specific experiences is only from health professionals, not women/girls
69.	Lewig K, Arney F and Salveron M. Challenges to parenting in a new culture: Implications for child and family welfare. *Eval Program Plann*. 2010; 33: 324-32.	Data on FGM/C not presented
70.	Leye E. Midwifery training needs identified when caring for women with female genital mutilation. *Evid Based Nurs*. 2016; 19: 7.	Not empirical research
71.	Liao LM, Elliott C, Ahmed F and Creighton SM. Adult recall of childhood female genital cutting and perceptions of its effects: a pilot study for service improvement and research feasibility. *J Obstet Gynaecol*. 2013; 33: 292-5.	Not qualitative research
72.	Lien IL and Schultz JH. Internalizing knowledge and changing attitudes to female genital cutting/mutilation. *Obstet Gynecol Int*. 2013; 2013: 467028.	Data presented on FGM/C, but no specific focus on FGM/C related healthcare
73.	McCarthy R and Haith-Cooper M. Evaluating the impact of befriending for pregnant asylum-seeking and refugee women. *British Journal of Midwifery*. 2013; 21: 404-9.	Data on FGM/C not presented
74.	McLeish J. Maternity experiences of asylum seekers in England. *British Journal of Midwifery* 2005; 13: 782-5.	Data on FGM/C not presented
75.	Mitello L and Proietti A. Feminine genitals modification: survey of a new phenomenon for nurse today. Second Part. *Prof Inferm*. 2006; 59: 242-5.	Not qualitative research
76.	Mitello L and Proietti A. Female genital modification: survey of new phenomenon for nurses today. First Part. *Prof Inferm*. 2006; 59: 171-81.	Not empirical research
77.	Moore, K. (2012) Female Genital Mutilation and Cultural Competency: Moving Towards Improved Management of Obstetric Care. Queen Margaret University, MSc Thesis, Edinburgh.	Data on healthcare-specific experiences is only from health professionals, not women/girls

For peer review only

No.	Study	Reason for Exclusion
78.	Morris MD, Popper ST, Rodwell TC, Brodine SK and Brouwer KC. Healthcare barriers of refugees post-resettlement. *J Community Health*. 2009; 34: 529-38.	Data on FGM/C not presented
79.	Morison L and others. How experiences and attitudes relating to female circumcision vary according to age on arrival in Britain: a study among young Somalis in London. *Ethnicity & Health*. 2004; 9: 75-100.	Data presented on FGM/C, but no specific focus on FGM/C related healthcare
80.	Mwangi-Powell F. Female genital mutilation: a case study in Birmingham. FORWARD, Report, 2000.	Data presented on FGM/C, but no specific focus on FGM/C related healthcare
81.	Nash E and Ranka P. Female genital mutilation: Knowledge, confidence, and approach to care in clinical practice of midwives and nurses in the UK. *International Journal of Gynecology and Obstetrics*. 2015; 131: E110.	Not qualitative research
82.	Nur Z. Cultural Competence in Health Care: Exploring the Experiences of Muslim Women within the Ontario Healthcare System. MSc Thesis, University of Ontario Institute of Technology (Canada), 2014,	Data on FGM/C not presented
83.	Ogbagzy RA. The construction of Eritrean women's identity in the Canadian context. MA Thesis, University of Toronto (Canada), 1999, p. 236.	Data presented on FGM/C, but no specific focus on FGM/C related healthcare
84.	Ogunsiji O. Understanding the dilemma of de-infibulation for women living with female genital mutilation. *Australian Nursing & Midwifery Journal*. 2015; 22: 51	Not empirical research
85.	Ogunsiji O and Wilkes L. Cultural explanations and procedures about female genital mutilation. *Australian Nursing & Midwifery Journal*. 2015; 22: 51	Data presented on FGM/C, but no specific focus on FGM/C related healthcare
86.	Ogunsiji O, Wilkes L, Peters K and Jackson D. Knowledge, attitudes and usage of cancer screening among West African migrant women. *J Clin Nurs*. 2013; 22: 1026-33.	Data on FGM/C not presented
87.	Ogunsiji, O. (2015) Female genital mutilation (FGM): Australian midwives' knowledge and attitudes. *Health Care Women Int.,* 36(11), 1179-1193.	Data on healthcare-specific experiences is only from health professionals, not women/girls
88.	Ogunsiji, O. (2016) Australian midwives' perspectives on managing obstetric care of women living with female genital circumcision/mutilation. *Health Care Women Int.,* 37(10), 1156-69.	Data on healthcare-specific experiences is only from health professionals, not women/girls
89.	Olsson E, Lau M, Lifvergren S and Chakhunashvili A. Community collaboration to increase foreign-born women's participation in a cervical cancer screening	Data on FGM/C not presented

For peer review only

No.	Study	Reason for Exclusion
	program in Sweden: A quality improvement project. *International Journal for Equity in Health*. 2014; 13	
90.	Pavlish, C.L., Noor, S. & Brandt, J., Somali immigrant women and the American health care system: discordant beliefs, divergent expectations, and silent worries, *Soc Sci Med.* 2010; 71:2, 353-361	Data on FGM/C not presented
91.	Peters K. Reasons why women choose a medical practice or a women's health centre for routine health screening: worker and client perspectives. *J Clin Nurs*. 2010; 19: 2557-64.	Data on FGM/C not presented
92.	Phillimore J. Migrant maternity in an era of superdiversity: new migrants' access to, and experience of, antenatal care in the West Midlands, UK. *Soc Sci Med*. 2016; 148: 152-9.	Data on FGM/C not presented
93.	Quilliam S. Female genital mutilation: three questions for us to consider. *J Fam Plann Reprod Health Care*. 2015; 41: 235-7.	Not empirical research
94.	Radford K. 'Unkind cuts': health policy and practice versus the health and emotional well-being of asylum-seekers and refugees in Ireland. *Journal of Ethnic and Migration Studies*. 2010; 36: 899-915.	Data on FGM/C not presented
95.	Razzak M. Sexual medicine: Pain and pleasure-reconstruction after female genital mutilation. *Nature Reviews Urology*. 2012; 9.	Not empirical research
96.	Reis E. Culture and cutting. *The Hastings Center Report*. 2012; 42: 3.	Not empirical research
97.	Rimmer SN. [Theme: circumcision. Somali women talk about taboo]. *Sygeplejersken*. 1994; 94: 12-3.	Not empirical research
98.	Roberts TL and Poblete X. Female genital mutilation: A survey of awareness, training and current practice. *Archives of Disease in Childhood: Annual Conference of the Royal College of Paediatrics and Child Health, RCPCH 2015*. Birmingham, UK: BMJ Publishing Group, 2015, p. G55.	Conference abstract
99.	Rogers C and Earnest J. A Cross-Generational Study of Contraception and Reproductive Health Among Sudanese and Eritrean Women in Brisbane, Australia. *Health Care Women Int*. 2014; 35: 334-56.	Data on FGM/C not presented
100.	Rosenberg LB, Gibson K and Shulman JF. When cultures collide: Female genital cutting and U.S. obstetric practice. *Obstet Gynecol*. 2009; 113: 931-4.	Not empirical research
101.	Rosenberg E, Kirmayer LJ, Xenocostas S, Dao MD and Loignon C. GPs' strategies in intercultural clinical encounters. *Fam Pract*. 2007; 24: 145-51.	Data on FGM/C not presented
102.	Roth K. Giving refuge: reflections on working with asylum seekers. *J Pain Symptom Manage*. 2010; 40: 149-54.	Not empirical research

For peer review only

No.	Study	Reason for Exclusion
103.	Rubin, E.A. (2000) When Cultures Collide: An Exploration of Cultural Competence and Cross-Cultural Communication between American Medical Providers and Immigrant Women who have been Circumcised. University of Massachusetts, PhD Thesis, USA.	Data on healthcare-specific experiences is only from health professionals, not women/girls
104.	Sala R and Manara D. Nurses and requests for female genital mutilation: cultural rights versus human rights. *Nurs Ethics*. 2001; 8: 247-58.	Not empirical research
105.	Schuster S. 'Gosh': a cross-cultural encounter with a Somali woman, a male interpreter and a gynecologist on female genital cutting/mutilation. *Patient Educ Couns*. 2015; 98: 127-8.	Not empirical research
106.	Shelp A. Women helping women: the Somali doula initiative. *International Journal of Childbirth Education*. 2004; 19: 4-7.	Not qualitative research
107.	Shipp MPL, Francis SA, Fluegge KR and Asfaw SA. Perceived Health Issues: A perspective from East-African immigrants. *Health, Culture and Society*. 2014; 6: 13-32.	Data on FGM/C not presented
108.	Sigurjonsson H, Jordal M and Lundgren TK. Reconstructive surgery after female genital mutilation. *International Journal of Gynecology and Obstetrics*. 2015; 131: E318-E9.	Conference abstract
109.	Simpson J, Robinson K, Creighton SM and Hodes D. Female genital mutilation: the role of health professionals in prevention, assessment, and management. *Br Med J*. 2012; 344.	Not empirical research
110.	Sokolik L. Somali immigrant and refugee women's experience of pregnancy and childbirth in the American healthcare system. *Commun Nurs Res*. 2007; 40: 538	Conference abstract
111.	Sudbury H and Robinson A. Barriers to sexual and reproductive health care for refugee and asylum-seeking women. *British Journal of Midwifery*. 2016; 24: 275-81.	Not empirical research
112.	Sundby J. Genital mutilation of women--is it a concern for Gynecologists? *Acta Obstet Gynecol Scand*. 1996; 75: 513-5.	Not empirical research
113.	Talle A. From "complete" to "impaired" body: Female circumcision in Somalia and London. *Disability in Local and Global worlds*. Berkeley, CA: University of California Press; US, 2007, p. 56-77.	Not empirical research
114.	Taillens F. ["I will not circumcise my daughter"]. *Krankenpflege - Soins Infirmiers*. 2012; 105: 55.	Not empirical research
115.	Tait P. Refugee Women in Australia and Woman-Centered Midwifery Care. *Nuritinga*. 2013: 31-9.	Not empirical research
116.	Tantet C, Aupiais C, Sorge F, Levy D, Lafon-Desmurs B and Faye A. MIG-01 - Female genital mutilations: Evaluation of the knowledge of general practitioners and	Not qualitative research

No.	Study	Reason for Exclusion
	physicians in travel counseling. *Med Mal Infect*. 2016; 46: 77.	
117.	Tatah EF. Female Circumcision: A Phenomenological Study of Somalian Immigrants to the United States. PhD Thesis, Walden University, 2016,	Data presented on FGM/C, but no specific focus on FGM/C related healthcare
118.	Trueland J. School nurses take lead on FGM. *Nurs Stand*. 2014; 28: 22-3.	Not empirical research
119.	Vercoutere A, Temmerman M and Leye E. Female genital mutilation, a story far away from home? Ethical, legal and clinical aspects of mutilated women in western countries. *Tijdschrift voor Geneeskunde*. 2011; 67: 237-41.	Not empirical research
120.	Villani M. From the “maturity” of a woman to surgery: Conditions for clitoris repair. *Sexologies*. 2009; 18: 259-61.	Not qualitative research
121.	Villani M. The way of speaking about "mutilation" and performing "repairing". *J Sex Med*. 2011; 8: 242.	Not qualitative research
122.	Villani M. Experiences with clitoris repair with counselling or counselling only in France, for effect on sexual satisfaction for women with FGM. *Int J Gynecol Obstet*. 2012; 119: S255.	Conference abstract
123.	Villani M, Griffin J and Bodenmann P. In their own words: the health and sexuality of immigrant women with infibulation living in Switzerland. *Soc Sci*. 2016; 5: 1.	Data presented on FGM/C, but no specific focus on FGM/C related healthcare
124.	Vissandjee B, Denetto S, Migliardi P and Proctor J. Female genital cutting (FGC) and the ethics of care: community engagement and cultural sensitivity at the interface of migration experiences. *BMC Int Health Hum Rights*. 2014; 14: 1-18.	Not empirical research
125.	Vloeberghs E, Knipscheer J, van der Kwaak A, Naleie Z and van den Muijsenbergh M. Veiled Pain: A Study In The Netherlands On The Psychological, Social And Relational Consequences Of Female Genital Mutilation. Pharos–Dutch National Knowledge and Advisory Centre on Refugees and Migrants' Health, 2010.	Duplicate report of included paper
126.	Wade L. The politics of acculturation: Female genital cutting and the challenge of building multicultural democracies. *Soc Probl*. 2011; 58: 518-37.	Data presented on FGM/C, but no specific focus on FGM/C related healthcare
127.	Widmark, C., Leval, A., Tishelman, C. & Ahlberg, B.M. (2010) Obstetric care at the intersection of science and culture: Swedish doctors' perspectives on obstetric care of women who have undergone female genital cutting. *J. Obstet. Gynaecol.,* **30**(6), 553-8.	Study focus is on health professionals, not women/girls

For peer review only

No.	Study	Reason for Exclusion
128.	Widmark, C., Tishelman, C. & Ahlberg, B.M. (2002) A study of Swedish midwives' encounters with infibulated African women in Sweden. *Midwifery,* 18(2), 113-25.	Data on healthcare-specific experiences is only from health professionals, not women/girls
129.	Williams KW. Somali Bantu health experience: Refugee resettlement in South Carolina. PhD Thesis, University of South Carolina, 2006	Data on FGM/C not presented
130.	Williams N, Offer GJ, Williams S and Everson N. Parental attitude in children referred for circumcision. *Br J Surg*. 1997; 84: 1722-3.	Data on FGM/C not presented
131.	Willis S. The cutting season. *Journal of Family Health Care*. 2014; 24: 9-12.	Not empirical research
132.	Wimmer-Puchinger B, Wolf H and Engleder A. [Female migrants in the health care system. Health care utilisation, access barriers and health promotion strategies]. *Migrantinnen im Gesundheitssystem Inanspruchnahme, Zugangsbarrieren und Strategien zur Gesundheitsforderung*. 2006; 49: 884-92.	Not empirical research
133.	Young H and McGrath K. A review of circumcision in New Zealand: 'I never liked doing them and I was pleased to give them up'. *Understanding Circumcision: A Multi-Disciplinary Approach To A Multi-Dimensional Problem*. Kluwer Academic Publishers, 2001.	Data on FGM/C not presented
134.	Yusuf L and Negash S. Vaginal calculus following severe form of female genital mutilation: a case report. *Ethiop Med J*. 2008; 46: 185-8.	Not OECD
135.	Zenner N, Liao LM, Richens Y and Creighton SM. Quality of obstetric and midwifery care for pregnant women who have undergone female genital mutilation. *J Obstet Gynaecol*. 2013; 33: 459-62.	Not qualitative research

For peer review only

Supplementary File 3: Detailed Characteristics of Included Studies

No.	Authors and date	Country	Study aims, focus and/or research question	Region and Setting (where specified)	Methodology and/or theoretical perspective	Methods and recruitment (where stated)	Data analysis	Whole study sample	Eligible participants and country of origin
**1.**	Abdi, R. (2012)[1]	UK	To determine the role of FGM/C in creating a social, individual and gendered identity for a Somali woman.	East and Northeast regions of London.<br><br>African Well Women's Service in North London.	Narrative and life history approach.	In-depth interviews. Recruited women who held a strong anti-FGM/C opinion.<br><br>Snowball sampling used.	Analysis using theoretical concept of the 'three-bodies'.	2 gynaecologists, 1 counsellor and 1 midwife 16 Somali women 2 men<br><br>Age range 20 – 72 years	16 Somali women
**2.**	Abdullahi, A., Copping, J., Kessel, A., Luck, M., & Bonell, C. (2009)[2]	UK	To explore the barriers to uptake of cervical screening and to identify strategies for overcoming these barriers.	London boroughs, Camden UK, North London.<br><br>Somali community centre.	Qualitative methods	Focus groups and in-depth interviews.<br><br>Recruitment from a range of settings.<br><br>Included snowball sampling.	Thematic analysis informed by an interpretivist approach.	50 Somali-born women<br><br>Age range 25-64 years	50 Somali-born women
**3.**	Ahlberg, B. M., Krantz, I., Lindmark, G., & Warsame, M. (2004)[3]	Sweden	Questions why FGM/C persists despite eradication interventions and the migration to non-FGM/C practising areas.	Different forums including a school, youth clinics and a girl's football team.	Emergent research design in the tradition of qualitative research.	Individual and group interviews.<br><br>Snowball sampling used.	Thematic analysis.	30 men, 50 women, 10 girls, 5 boys and 15 professionals	50 women of Somali origin
**4.**	Ahmed, M., (2005)[4]	UK	To explore the influence of cross-cultural psychology, women's experience of FGM/C and their attitudes towards it.	West, East and South London and Sheffield.<br><br>Women's homes,	Qualitative methods	Focus groups recruited via word of mouth, through attending Somali community centres and	Thematic analysis.	56 women<br><br>Age range 18–70 years	56 women. The majority born in Somalia – with some born in UK

No.	Authors and date	Country	Study aims, focus and/or research question	Region and Setting (where specified)	Methodology and/or theoretical perspective	Methods and recruitment (where stated)	Data analysis	Whole study sample	Eligible participants and country of origin
				community centre and a mosque.		verbal advertising of the project			and the Middle East
**5.**	Amereseker e, M., Borg, R., Frederick, J., Vragovic, O., Saia, K., & Raj, A. (2011)[5]	USA	To assess the prevalence of FGM/C in the interviewed sample, gain insight into commonly held beliefs about caesarean delivery, and understand patient perceptions of communication surrounding caesarean delivery and FGM/C by obstetric providers in the USA.	Greater Boston	Exploratory qualitative study	Semi structured in-depth interviews<br><br>Snowball sampling and linkage with the Somali Development Centre (SDC), a local community based organization	Thematic analysis	23 Somali immigrant women<br><br>Age range 25-52 years	23 Somali participants
**6.**	Asefaw, F. (2007)[6]	Eritrea and Germany	To explore whether migration changes their attitude towards FGC. How do circumcised women deal with it in a society that opposes FGC? Can coherence and reasons be determined for why circumcised women subjectively suffer more in migration compared with when they live in their country of origin?	In the migrant's homes, in clinics or as part of awareness campaigns of the woman's organisation FORWARD	Mixed methods. Survey and qualitative methods	Survey and semi-structured qualitative interviews.	Unclear	420 women and 50 men<br><br>Age range 15-70 years	31 Eritrean migrants

No.	Authors and date	Country	Study aims, focus and/or research question	Region and Setting (where specified)	Methodology and/or theoretical perspective	Methods and recruitment (where stated)	Data analysis	Whole study sample	Eligible participants and country of origin
**7.**	Baldeh, F. (2013)[7]	Scotland	To explore the experiences of obstetric care in Scotland among women who have undergone FGM/C. The objectives are to explore: · How women with FGM experience obstetric care in Scotland · The role of health workers in helping or hindering communication; · Potential changes and improvements in services.	Glasgow and Edinburgh<br><br>In participants homes	Qualitative approach interpretivist paradigm and a feminist perspective	In-depth interviews.<br><br>Convenience sampling used.	Thematic analysis	7 women	7 women in total. 2 from The Gambia, 1 from Ghana and 1 from Sudan.
**8.**	Ballesteros Meseguer, C., Almansa Martinez, P., Pastor Bravo, M. d. M., & Jimenez Ruiz, I. (2014)[8]	Spain	To explore the perceptions of a group of women who had experienced FGM/C regarding their sexual and reproductive health, with an aim to consider the impact on their sexual relationships, pregnancy and labour, as well as the social repercussions of this practice.	Region of Murcia, Spain.<br><br>In participants' homes.	Phenomenological qualitative study	In-depth interviews using structured open-ended approach<br><br>Informal conversations with midwives	Discourse analysis	9 sub-Saharan Africa women.<br><br>Mean age 30 years	9 women from Senegal
**9.**	Behrendt, A. (2011)[9]	Germany	To listen to the opinions, perceptions and propositions of immigrants from Sub-Saharan Africa regarding the practice of FGM/C. To develop an effective and locally adapted approach to promote the abandonment of FGM/C among immigrant groups in Hamburg.	Hamburg	Participatory, cooperative approach which engages community members in a joint evaluation research process. Triangulated research design behaviour change model.	Mixed methods.<br><br>Qualitative and quantitative data collection from different target groups (1) key informant (health professional) interviews, (2) semi-structured individual interviews with African	Qualitative data analysed and classified into themes.<br><br>Quantitative data analysed using SPSS	Total number of 1,767 participants with African migration background were interviewed for the quantitative survey<br><br>91 semi structured interviews with African	91 key informant interviews - largely from Ghana, Nigeria and Togo

For peer review only - http://bmjopen.bmj.com/site/about/guidelines.xhtml

No.	Authors and date	Country	Study aims, focus and/or research question	Region and Setting (where specified)	Methodology and/or theoretical perspective	Methods and recruitment (where stated)	Data analysis	Whole study sample	Eligible participants and country of origin
						community members, (3) survey with community members. Snowball sampling		community members<br><br>27 interviews with health professionals<br><br>Age range 15-83 years	
**10.**	Beine, K., Fullerton, J., Palinkas, L., & Anders, B. (1995)[10]	USA	to understand their cultural beliefs and behaviours better during pregnancy and to determine their attitudes toward their prenatal care experiences in San Diego, in order to inform providers and enhance culturally relevant service programs.	Conducted at International Rescue Committee office or private homes of group participants	Exploratory study	Focus group interview.<br><br>Recruitment via community social networks	Content analysis	14 women<br><br>Age range 20 to 42 years	14 Somali women
**11.**	Berggren, V., Bergstrom, S., & Edberg, A.-K. (2006)[11]	Sweden	To explore the experience of FGM and the experiences of encounters with Swedish maternal health care among women from Eritrea, Somalia, and Sudan living in Sweden.	Three different cities in Sweden,<br><br>Conducted in the women's homes	Exploratory qualitative study	26 interviews recruited via snowball sampling or net-work sampling	Latent content analysis	22 women, 6 from Eritrea, 11 from Somalia, and 5 from Sudan (1 excluded as no FGM)<br><br>Age range 24 to 73 years	21 women from Eritrea, Somalia, or Sudan
**12.**	Betts, V. (2011)[12]	Canada	To critically examine popular ideas of Somali women's identity, gender and sexuality in Canada; and, to evaluate Somali women's experiences with Canadian health care professionals, and how it compares with their experiences with medical professionals in Somalia.	Greater Toronto Area, Canada. From Community Centre in a small office	Anthropological methods	Individual and paired interviews one discussion group of fourteen women.	Placed loosely into categories	Thirteen women aged 18-65 years	13 Somali women

For peer review only - http://bmjopen.bmj.com/site/about/guidelines.xhtml

No.	Authors and date	Country	Study aims, focus and/or research question	Region and Setting (where specified)	Methodology and/or theoretical perspective	Methods and recruitment (where stated)	Data analysis	Whole study sample	Eligible participants and country of origin
**13.**	Bravo Pastor del Mar, M. (2014)[13]	Spain	To identify the knowledge, attitudes and experiences regarding genital mutilation of a group of women from Sub-Saharan African countries where FGM/C is a traditional practice.	Region of Murcia, Spain.<br><br>The interviews were held individually at the homes of the participants, at their request, always in the absence of the husband and occasionally in the presence of minor children.	A qualitative study using the life story method	Life Story interviews held until data saturation was reached. Informal conversations including telephone calls, emails, informal chats and informal meetings with key informants. Socio-demographic Questionnaire<br><br>Contact through different associations for African women and snowball sampling	The analysis was carried out using the categories that emerged from the narratives collected in the interviews.	20 Senegalese women, 2 Nigerian women and 2 Gambian women<br><br>Age range 23 - 42 years	20 Senegalese women, 2 Nigerian women and 2 Gambian women
**14.**	Bulman, K. H., & McCourt, C. (2002)[14]	UK	To develop an understanding of the reality faced by Somali women in their contacts with the maternity services in the UK and to explore professionals perceptions of Somali women and their maternity care needs - how they approached providing for these needs and their ideas for improving services.	West London<br><br>Conducted in participants homes or clinics	Qualitative (ethnographic context narrative approach)	6 individual interviews and two focus groups with Somali women, recruited via snowball sampling method<br><br>2 focus groups with midwives, (caseload and hospital midwives). Individual interviews with a Somali health-link worker, a	Thematic analysis	12 Somali women participating in qualitative interviews<br><br>Health workers (number not specified) were also interviewed	12 Somali women

only

No.	Authors and date	Country	Study aims, focus and/or research question	Region and Setting (where specified)	Methodology and/or theoretical perspective	Methods and recruitment (where stated)	Data analysis	Whole study sample	Eligible participants and country of origin
						woman on the Health and Race Working Group, and an obstetric registrar.			
**15.**	Carroll, J., Epstein, R., Fiscella, K., Gipson, T., Volpe, E., & Jean-Pierre, P. (2007)[15]	USA	To explore the range of both Bantu and other Somali women's experiences with communication about preventive health services in the US in order to understand the role communication plays in disparities in this population.	Rochester, NY, USA. At a time and place convenient to the participant	Grounded theory	Individual interviews, a Somali women's focus group was conducted to check thematic validity and clarify questions and concepts. Word of mouth, key informants and primary care provider referrals; and snowballing	Grounded theory, a coding/ editing method that extracts emerging themes	34 women Age range 18 - 53 years (median age 27 years)	34 Somali refugee women
**16.**	Chalmers, B., & Omer-Hashi, K. (2002)[16]	Canada	To explore recent perinatal experiences in Canada	Greater Toronto region, at participants home	Unclear	Sampling from community sites and snowball sampling.<br><br>Structured questionnaire with 4 open ended questions, administered face to face involving reflection and discussion	Unclear	432 Somali women<br><br>Mean age 34 years	415 Somali women<br><br>(who responded to the open ended questions)

No.	Authors and date	Country	Study aims, focus and/or research question	Region and Setting (where specified)	Methodology and/or theoretical perspective	Methods and recruitment (where stated)	Data analysis	Whole study sample	Eligible participants and country of origin
**17.**	Degni, F., Suominen, S. B., El Ansari, W., Vehviläinen -Julkunen, K., & Essen, B. (2014)[17]	Finland	To explore immigrant Somali women's experiences of reproductive and maternity health care services (RMHCS) and their perceptions about the service providers.	Helsinki, Vantaa, Espoo and Turku cities in Finland.<br><br>Conducted at woman's home and swimming pool hall	Exploratory, qualitative study	Focus group discussions via purposeful sampling	Guided by concepts of cultural care	70 women<br><br>Age range 18~50 years	70 Somali women
**18.**	d'Entremont, M., Smythe, L., & McAra-Couper, J. (2014)[18]	France	Explored the experiences of childbirth for women who have been excised.	At a place of the woman's choosing	Hermeneutic phenomenology Gadamerian philosophy	Interviews Purposive sampling methods	Constant, circular process of thematic, textual interpretations	4 women	4 women from different countries
**19.**	Essén, B., Johnsdotter, S., Hovelius, B., Gudmundsson, S., Sjöberg, N. O., Friedman, J., & Östergren, P. O. (2000)[19]	Sweden	To explore the attitudes, strategies and habits of Somalian immigrant women related to pregnancy and childbirth, in order to gain an understanding as to how cultural factors might affect perinatal outcome.	Localities of the different Somalian immigrant associations in their own residential areas	Qualitative	Qualitative in-depth interviews. Recruited from community organisations	Systematic textual analysis	15 women<br><br>Age range 20-55 years	15 Somali women
**20.**	Fawcett, L., (2014)[20]	USA	To identify and measure patterns of cultural knowledge and variations (cross-cultural agreement and disagreement) between Somali resettled refugee women (SRRW) and healthcare providers (HCP) on childbearing models. To investigate	Arizona Metropolitan Phoenix area, in a café or participants' homes	Quantitative survey and ethnographic methodologies and phenomenological approaches using open-ended semi-structured interviews.	Culture consensus questionnaire (CCM), participatory observations, in-depth semi-structured interviews.	Phenomenological analysis and inductive explorative approach	Questionnaires (N=174)<br><br>Interviews; Somali resettled refugee women (SRRW, n=30);	30 Somali resettled refugee women

No.	Authors and date	Country	Study aims, focus and/or research question	Region and Setting (where specified)	Methodology and/or theoretical perspective	Methods and recruitment (where stated)	Data analysis	Whole study sample	Eligible participants and country of origin
			and measure intra-cultural variations in the domain of childbearing knowledge among Somali resettled refugee women. To explore the subjective and intersubjective perceptions of female circumcision.		Used theory of Cultural Consensus Model and concepts of embodiment.	Used snowball sampling via community organisations		Healthcare Providers (HCP, n=10)<br><br>Age range 18-70 years	
**21.**	Gali, M. A. (1997)[21]	USA	To explore the relationship between the psychological and medical concerns circumcised women face and barriers to reproductive health care delivery in the United States.	San Francisco Bay Area.	Qualitative exploratory research	Semi-structured interviews were conducted in person and by mail.<br><br>Recruitment using community organisations	Content analysis	26 women<br><br>Aged 21 -45 years	26 women from Sudan, Eritrea, Ethiopia and Egypt
**22.**	Ghebre, R. G., Sewali, B., Osman, S., Adawe, A., Nguyen, H. T., Okuyemi, K. S., & Joseph, A. (2015)[22]	USA	Exploring suitable language, structure, and context to describe cervical cancer prevention and screening methods among women in Minnesota's Somali community.	Minnesota<br><br>Community locations	Qualitative study with community engagement socio-ecological framework	Informant interviews. Used snowball sampling.	Themes and subthemes using the socio-ecological framework	23 participants<br><br>Age range 26-45 years	23 Somali women
**23.**	Glazer, E., (2012)[23]	Canada	Aimed to explore this possibly primarily through subjective reports of pain discussing testable pain along with interpretation and meaning of bodily pain and FGM/C from the perspective of women with FGM/C	Greater Toronto Area, Canada at community health centre (CHC)	Mixed-methods, including a qualitative component drawing upon interpretive and embodied phenomenology	Qualitative interview semi-structured interviews using a convenience sample	Phenomenological analysis	14 Somali-Canadian participants<br><br>Age range 21-46 years	14 Somali-Canadian participants

For peer review only - http://bmjopen.bmj.com/site/about/guidelines.xhtml

No.	Authors and date	Country	Study aims, focus and/or research question	Region and Setting (where specified)	Methodology and/or theoretical perspective	Methods and recruitment (where stated)	Data analysis	Whole study sample	Eligible participants and country of origin
**24.**	Glover, J., Liebling, H., Barrett, H., & Goodman, S. (2017)[24]	UK	Investigate the experiences of women who had undergone FGM and the psychological effects they had experienced. It also sought to understand the psychological impact of physical health complications, survivor's resilience and their cultural context	Multiple UK settings	A qualitative methodology of grounded theory Principles of feminist research were observed	In-depth interviews from a non-probability convenience sampling frame based on snowballing sampling	Theoretical memos and grounded theory analysis	20 women survivors of FGM<br><br>Mean age 38 years (SD=6.5, range=25-51)	20 women from various countries Somalia (n=10), Kenya (n=2), South Africa (n=6), and The Gambia (n=2)
**25.**	Guerin, P. B., Allotey, P., Elmi, F. H., & Baho, S. (2006)[25]	Australia and New Zealand	To enhance the understanding of the immigration experience on the reproductive health of women from Horn of African and Middle Eastern countries resident in Victoria to provide a safe means by which Somali women could express their views about FGM/C	Melbourne Australia and New Zealand.<br><br>In homes of the participant	Ethnographic cohort design Community-Based Participatory Research	In-depth, open-ended and unstructured interviews, focus group discussions and participant observation. Sampling involved using community networks and snowball approach.	Thematic analysis	Australia (n = 255) and New Zealand (n = 64). African women in the sample (n = 141) Middle Eastern women (n = 114) 54 women; 19 and 50 years	10 women from Somalia, Ethiopia, Eritrea, Sudan and Nigeria
**26.**	Hill, N., Hunt, E., & Hyrkas, K. (2012)[26]	USA	To describe and better understand Somali immigrant women's health care experiences and beliefs regarding pregnancy and birth in the Unites States.	North-eastern United States<br><br>Setting not given	Qualitative study	Focus group convenience sampling and women in turn identified friends and family	Thematic content analysis	18 participants<br><br>Age range 27-42 years	18 Somali women
**27.**	Hussein, E., (2010)[27]	UK	To gain an in-depth understanding of some of the experiences and perceptions of women coming from countries with high prevalence rates of FGM/C living in Bristol.	Setting not given	Participatory Ethnographic Evaluation and Research (PEER) and tenets of social construct	Informal in-depth interviews in third person loosely structured interviews. Equipping members from	Not stated	8 adult women	8 Somali and Sudanese adult women

For peer review only - http://bmjopen.bmj.com/site/about/guidelines.xhtml

No.	Authors and date	Country	Study aims, focus and/or research question	Region and Setting (where specified)	Methodology and/or theoretical perspective	Methods and recruitment (where stated)	Data analysis	Whole study sample	Eligible participants and country of origin
						the community to become 'PEER researchers			
**28.**	Hussen, M. A. (2014)[28]	New Zealand	To understand the situation of both the Christchurch women with FGM and their health providers, in order to increase knowledge of FGM and ensure improved health services for women with this condition.	Christchurch, New Zealand, at community gatherings	Qualitative descriptive research methods	Focus group approach for women from East Africa known to the social worker and researcher<br><br>Individual interviews with health professionals	Thematic analysis	20 focus group participants – mean age 21-65 years<br><br>3 health providers	20 women from Eritrea, Ethiopia, Somalia and Sudan
**29.**	Johansen, R.E. (2017)[29]	Norway	Explores experiences and perceptions of premarital deinfibulation. It explores whether Somali and Sudanese men and women understand deinfibulation as a purely medical issue or whether their use of the services is also affected by the cultural meaning of infibulation.	Oslo and other villages and towns in Norway. From participant home, workplace or public space such as a coffee bar or a park	Qualitative study	In-depth and key informant interviews Participant observation validation seminars. Utilised Snowball sampling	Themes and patterns	23 women and 13 men<br><br>Age range 18-65 years	23 Somali and Sudanese women
**30.**	Jones, A. (2010)[30]	UK	To understand the experiences and views of women who had undergone female genital mutilation (FGM/C).	South England, UK, in participant own home or the community centre	Interpretative phenomenological analysis (IPA) relativist approach.	Semi-structured interview. Recruited from FORWARD, community group	Interpretative Phenomenological Analysis (IPA)	Six participants<br><br>Age range 19-45 years	6 participants from Sudan and Somalia

No.	Authors and date	Country	Study aims, focus and/or research question	Region and Setting (where specified)	Methodology and/or theoretical perspective	Methods and recruitment (where stated)	Data analysis	Whole study sample	Eligible participants and country of origin
**31.**	Khaja, K. (2004)[31]	Canada and USA	To describe their experiences with circumcision and to share the personal and cultural meanings of those experiences	Toronto, Canada, and Salt Lake City, Utah. Setting not stated.	Life history methodology / ethnographic	Face-to-face, individual life history interviews. Purposive sampling recruited with the assistance of targeted Somali community members or service providers	Content analysis	17 women<br><br>Age range 20-79 years	17 Somali women
**32.**	Khaja, K., Lay, K., & Boys, S. (2010)[32]	Canada and USA	Examined the lives of Somali Muslim women who were circumcised.	Ontario Canada and Salt Lake City, Utah North America Setting N/S	Exploratory qualitative	Face-to-face interviews	Not stated	17 circumcised Somali women<br><br>Age range 20-79 years	17 circumcised Somali women
**33.**	Lundberg, P. C., & Gerezgiher, A. (2008)[33]	Sweden	to explore the experiences from pregnancy and childbirth related to FGM/C among Eritrean immigrant women	At informants' homes or in a separate room	Ethnographic method	Semi-structured and open-ended in-depth interview Purposive sampling with snowball sampling technique	Thematic analysis	15 women<br><br>Age range 31-45 years	15 Eritrean women
**34.**	Maier, C. (2003)[34]	Austria	Three questions:<br>· How did the affected woman experience and handle her genital mutilation when she was a girl? How did this influence her life and what are the physical and mental consequences? · In the context of FGM: Did immigration add new problem areas?	Not stated	Qualitative research	Guided interviews with a relatively open design	Not stated	3 women	3 women from different countries

No.	Authors and date	Country	Study aims, focus and/or research question	Region and Setting (where specified)	Methodology and/or theoretical perspective	Methods and recruitment (where stated)	Data analysis	Whole study sample	Eligible participants and country of origin
**35.**	Maternity Action. (2014)[35]	UK	To explore potential barriers to healthcare, both for women in general, and for women who experience comparatively poor health outcomes, in order to contribute to an understanding of how these may be addressed	Leeds, Manchester and London, UK. Community groups were hosted and facilitated Organisations	Online survey, and a series of focus groups	Focus groups recruited from service user networks	Themes from the literature	261 in the survey<br><br>23 women in focus groups.	23 women from several countries
**36.**	McNeely, S., & Christie-de Jong, F. (2016)[36]	USA	To explore perspectives of Somali refugees on FGM/C and potential changes in, these after migration to the USA.	Denver metropolitan area, USA in participants' homes	Descriptive, socio-cultural study	Semi-structured and open-ended interviews snowball sampling with two gatekeepers	Thematic content analysis	13 Somali refugees<br><br>Age range 20-70 years	13 Somali women
**37.**	Moxey, J. M., & Jones, L. L. (2016)[37]	UK	To explore how Somali women exposed to FGM, experience and perceive antenatal and intrapartum care in England	Birmingham, West Midlands<br><br>In a private room at a community centre	Descriptive qualitative methodology	Semi-structured Face-to-face interviews purposively sampled from community centres and using a snowball approach	Inductive thematic analysis	10 women<br><br>Age range 20-49 years	10 Somali women
**38.**	Murray, L., Windsor, C., Parker, E., & Tewfik, O. (2010)[38]	Australia	To uncover first-person descriptions of the birth experiences of African refugee women in Brisbane, Australia, and to explore the common themes that emerged from their experiences.	In room at a clinic or in participant own homes	Phenomenology Husserlian phenomenological framework.	Semi structured interviews purposeful, snowball strategy	Giorgi's phenomenological analysis.	10 women<br><br>Age range 20-40 years	10 women from Sudan Liberia Ethiopia Somalia
**39.**	Norman, K., Gegzabher, S. B., & Otoo-Oyortey, N. (2016)[39]	UK	Rapid participatory study amongst migrant communities in Norfolk and Essex, to help shed more light on this issue and to support their community engagement programme.	Norfolk, and Essex, in the community	Participatory Ethnographic Evaluation and Research (PEER) PEER is a qualitative, participatory	Interviews using snowball sampling	Participatory thematic analysis	18 peer researchers and 33 interviewees (each of the peer researchers chose two	33 women from various countries

No.	Authors and date	Country	Study aims, focus and/or research question	Region and Setting (where specified)	Methodology and/or theoretical perspective	Methods and recruitment (where stated)	Data analysis	Whole study sample	Eligible participants and country of origin
					research methodology			friends/peers to interview)	
**40.**	Norman, K., Hemmings, J., Hussein, E., & Otoo-Oyortey, N. (2009)[40]	UK	To gain in-depth insights from women originally from high-prevalence FGM countries, and currently living in and around Westminster.	Borough of Westminster, London in the community	Participatory Ethnographic Evaluation and Research (PEER) a qualitative, participatory research method	In-depth conversational interviews snowball sampling used	Participatory thematic analysis	10 peer researchers conducted interviews with 3 friends each (n=30). Peer researchers were also interviewed.<br><br>Age range 25 years +	40 women<br><br>(10 peer researchers who were also interviewed and 30 other women) from Sudan, Eritrea and Somalia
**41.**	O'Brien, O., Baldeh, F., Hassan, J., & Baillie, M. (2017)[41]	Scotland	To facilitate community engagement with FGM affected populations, to enable their voices to contribute to the development of awareness raising around FGM and to ensuring culturally appropriate FGM services for Scotland.	Glasgow and Edinburgh Scotland, in the community	Participatory Ethnographic Evaluation Research (PEER) ethnographic research principles	Interviews with contacts in community-based organisations and friends	Participatory analysis	19 peer researchers (who were also interviewed) and who then carried out 29 interviews	48 women<br><br>(19 peer researchers who were also interviewed and 29 women) from Sudan Nigeria Zimbabwe
**42.**	O'Brien, O., Baldeh, F., Sivapatham, S., Brown, E., & O'May, F. (2016)[42]	Scotland	To facilitate community engagement on FGM with women, men, young people and religious leaders to enable their voices to be heard and contribute to the development of awareness raising around FGM as well as ensuring culturally appropriate services for Scotland	Glasgow and Edinburgh Scotland, in the community,	Participatory Ethnographic Evaluation Research (PEER) ethnographic research principles	Interviews and focus groups. Convenience and snowball sampling through contacts	Participatory analysis	28 peer researchers (12 women, 16 men) who carried out 78 interviews	39 women<br><br>(9 female peer researchers who were also interviewed and 30 women) from 17 African countries

No.	Authors and date	Country	Study aims, focus and/or research question	Region and Setting (where specified)	Methodology and/or theoretical perspective	Methods and recruitment (where stated)	Data analysis	Whole study sample	Eligible participants and country of origin
**43.**	Palfreyman, A., Brown, E., & Nam, S. (2011)[43]	UK	To explore FGM within the context of Birmingham.	Birmingham	Participatory Ethnographic Evaluation and Research (PEER)	In-depth semi-structured interviews with and by peers recruited using community organisations and snowball sampling	Participatory thematic analysis	Fifteen peer researchers conducted a total of 90 interviews<br><br>Age range 17 - 48 years	105 women<br><br>(15 peer researchers who were also interviewed, and 90 women) from Somali, Eritrea, Sudan, Sierra Leon and Gambia
**44.**	Recchia, N., & McGarry, J. (2017)[44]	UK	The overall aim of the study was to develop and facilitate a participant led arts-based workshop with women who were survivors of FGM in order to explore their experiences and the impact of FGM on their health and well-being.	Nottingham<br><br>At a local FGM specialist support organisation	Creative narrative approach which included the sharing of personal stories, the creation of pottery models and the sharing of artefacts - arts-based research approach using a feminist theoretical framework	The composition of the workshop essentially encompassed two main strands: the creation of a persona and sharing artefacts. Recruited though a local FGM specialist support organisation	Narrative approach	Six women agreed to take part in the project<br><br>Age range 25-51 years	6 women from Kenya, Nigeria and Ethiopia
**45.**	Safari, F., (2013)[45]	UK	Explored women's experience of deinfibulation and its aftermath.	London<br><br>At NHS hospital, or participant's home	Qualitative approach Interpretive Phenomenological Analysis (IPA)	Semi-structured interview	Interpretive Phenomenologi cal Analysis (IPA)	9 women<br><br>Age range 19-44 years	9 women from Somalia Eritrea
**46.**	Salad, J., Verdonk, P., de Boer, F., & Abma, T. A. (2015)[46]	Netherlands	To explore the perceptions of Somali women living in the Netherlands regarding measures to prevent cervical cancer	In cafés, libraries, schools, and community centres	Qualitative approach Intersectionality is therefore integrated in the HBM in this study	Semi-structured interviews, group discussions, recruited though Purposive	Framework of intersectionalit y enabled a comprehensive thematic	Interviews with 14 young women (aged 17-21) and 6 older women	46 Somali women

No.	Authors and date	Country	Study aims, focus and/or research question	Region and Setting (where specified)	Methodology and/or theoretical perspective	Methods and recruitment (where stated)	Data analysis	Whole study sample	Eligible participants and country of origin
						sampling using Snowball sampling	content analysis	(mothers aged 30-46)<br><br>Group discussions with 26 women (mothers aged 23-66)	
**47.**	Shaw, E. (1985)[47]	USA	To 1) provide a summary of basic information about female circumcision; 2) summarize the results of an exploratory study which identified special needs and concerns of circumcised women who have used the western medical system while living in the United States, and 3) identify problems and concerns student health care providers have encountered while caring for this group of women.	Southwestern University town. Setting not stated.	Exploratory study	Informal interviews	Thematic analysis	12 circumcised women<br>Six Planned Parenthood Centres and four obstetric gynaecological clinics responded	12 circumcised women from Sudan, Egypt, and Somalia
**48.**	Shermarke, M.A.A. (1996)[48]	Canada	Explores female circumcision within the Canadian community perspective	Montreal, Quebec and Ottawa, Ontario.<br><br>In homes of the interviewees or in selected, quiet, public places	Case study and Qualitative methods	Individual interviews selected through personal contacts	Thematic analysis	4 interviewees from Montreal and 4 from Ottawa<br><br>Age range 16 - 58 years	8 Somali women

No.	Authors and date	Country	Study aims, focus and/or research question	Region and Setting (where specified)	Methodology and/or theoretical perspective	Methods and recruitment (where stated)	Data analysis	Whole study sample	Eligible participants and country of origin
**49.**	Straus, L., McEwen, A., & Hussein, F. M. (2009)[49]	UK	To examine cultural and social aspects of childbirth, and to determine how they intersect with the needs and experiences of Somali women in the UK. To explore the experiences and needs of Somali women in the UK during pregnancy and childbirth.	London<br><br>In the community	Ethnographic approach narrative approach	In-depth narrative interviews<br><br>Recruitment from two community Somali women's groups and snowballing	Thematic analysis	8 Somali women<br><br>Age range 23-57 years	8 Somali women
**50.**	Thierfelder, C. (2003)[50]	Switzerland	To analyse the specific situation of genitally mutilated African immigrant women, and the health care providers who encounter them in the Swiss health care system.	Cantons of Geneva, Vaud and Zurich.<br><br>Setting not stated	Qualitative research.	Semi-structured, in-depth interviews and focus group discussions.<br><br>Utilised purposive sampling and snowball sampling drawing on contacts with migrant-support and community organisations.	Thematic content analysis	Women (n=29) and men (n=3) (n=37) with Swiss health care providers (20 physicians and 17 midwives)<br><br>Age range 17-64 years	29 women from Somalia and Eritrea
**51.**	Thierfelder, C., Tanner, M., & Bodiang, C. M. K. (2005)[51]	Switzerland	To analyse how immigrant women with FGM experience gynaecological/obstetrical care in the Swiss health care system, and to investigate if physicians and midwives treat and counsel FGM related complications adequately.	Zurich, Lausanne and Geneva, Switzerland. Setting not stated.	Qualitative methods.	Four focus group discussions and interviews.<br><br>Purposive sampling via migrant-support and community organisations.	Unclear	29 women 37 health care professionals	29 women from Somalia and Eritrea

No.	Authors and date	Country	Study aims, focus and/or research question	Region and Setting (where specified)	Methodology and/or theoretical perspective	Methods and recruitment (where stated)	Data analysis	Whole study sample	Eligible participants and country of origin
**52.**	Upvall, M. J., Mohammed, K., & Dodge, P. D. (2009)[52]	USA	To explore the healthcare perspectives of Somali Bantu refugees in relation to their status as women who have been circumcised and recently resettled in the US.	South-western Pennsylvania, at a local refugee centre	Qualitative study	Focus groups purposive sample identified by a local non-profit organization	Thematic analysis	23 resettled Somali women, a local female physician<br><br>Age range 19-43 years	23 resettled Somali women
**53.**	Vangen, S., Johansen, R. E. B., Sundby, J., Traeen, B., & Stray-Pedersen, B. (2004)[53]	Norway	To explore how perinatal care practice may influence labour outcomes among circumcised women	At women's homes or public cafes, or hospital	Qualitative method drawing on theories of empowerment	In-depth interviews. Recruited from Somali's association and in health facilities and through snowball sampling	Thematic analysis	23 Somali immigrants and 36 Norwegian health care professionals;<br><br>Age range 18-55 years	23 Somali women
**54.**	Vaughan, C., White, N., Keogh, L., Tobin, J., Ha, B., Ibrahim, M., & Bayly, C. (2014)[54]	Australia	To improve understanding of the impacts of FGC and to make suggestions for service development - by engaging with local women, communities and health providers	North Yarra Carlton, Collingwood and Fitzroy<br><br>Research conducted in community centre meeting rooms.	Community based qualitative approach	8 focus group discussions, 4 small group discussions, 10 individual interviews with community members. Recruitment from community consultations, community organisations and networks<br><br>11 interviews with service providers	Thematic analysis	123 individuals<br><br>112 migrant men and women and 11 health providers	87 migrant women from Eritrea, Ethiopia, Somali and Sudan
**55.**	Vaughan, C., White, N., Keogh, L., Tobin, J., Murdolo, A.,	Australia	To identify the FGC-related service needs prioritised by affected communities living in Victorian regional centres, and to build	Australia Regional Victoria Ballarat, Geelong, Latrobe	Community based qualitative approach	Focus group discussions.<br><br>Recruitment from migrant support agencies	Thematic analysis	51 migrant community members 950 women, 1 man)	50 women from Togo Sudan and Kenya

No.	Authors and date	Country	Study aims, focus and/or research question	Region and Setting (where specified)	Methodology and/or theoretical perspective	Methods and recruitment (where stated)	Data analysis	Whole study sample	Eligible participants and country of origin
	Quiazon, R., & Bayly, C. (2014)[55]		evidence as to the training, education and professional development required for service providers working in regional Victoria to be able to meet these needs.	Valley and Shepparton,<br><br>Research conducted in community meeting rooms		and then using snowball sampling		15 health service providers	
**56.**	Vloeberghs, E., van der Kwaak, A., Knipscheer, J., & van den Muijsenbergh, M. (2012)[56]	Netherlands	Explored psychosocial and relational problems of African immigrant women in the Netherlands who underwent female genital mutilation/cutting (FGM/C), the causes they attribute to these problems and the way they cope with these health complaints.	In participants' homes	Mixed-methods study used standardised questionnaires as well as in-depth interviews, and qualitative participatory methods Grounded Theory	In-depth interviews using peer researchers. Snowball sampling used	Thematic framework analysis	66 women;<br><br>Age range 18~69	66 women from Somalia, Sudan, Eritrea, Ethiopia or Sierra Leone
**57.**	Wiklund, H., Aden, A. S., Högberg, U., Wikman, M., & Dahlgren, L. (2000)[57]	Sweden	To study the childbirth experiences of Somali women and men in Sweden.	Umea and nearby municipalities; in women's homes	Grounded theory	Interviews were semi-structured. Recruited from the Swedish Medical Birth Register and `patient' records	Grounded theory analysis	9 women and 4 husbands of the women studied and additional 3 men; 21-55 years	9 Somali women

References of Included Studies

1. Abdi R. Carving culture: Creating identity through female genital cutting. *Durham Anthropology Journal* 2012;18(1):115-53.

2. Abdullahi A, Copping J, Kessel A, et al. Cervical screening: perceptions and barriers to uptake among Somali women in Camden. *Public Health* 2009;123(10):680-5. doi: 10.1016/j.puhe.2009.09.011

3. Ahlberg BM, Krantz I, Lindmark G, et al. 'It's only a tradition': making sense of eradication interventions and the persistence of female 'circumcision' within a Swedish context. *Crit Soc Policy* 2004;24(1):50-78. doi: 10.1107/0261018304039679

4. Ahmed M. Attitudes towards FGM among Somali women living in the UK. In: Momoh C, ed. Female Genital Mutilation. London: Radcliffe 2005:95-117.

5. Ameresekere M, Borg R, Frederick J, et al. Somali immigrant women's perceptions of cesarean delivery and patient-provider communication surrounding female circumcision and childbirth in the USA. *Int J Gynecol Obstet* 2011;115(3):227-30. doi: 10.1016/j.ijgo.2011.07.019

6. Asefaw F. [Female Genital Mutilation: A Field Study with Special Consideration of the Background as well as the Health and Psychosexual Consquences for those Affected and their Partners in Eritrea and in Germany] Weibliche Genitalbeschneidung: Eine Feldstudie Unter besonderer Berücksichtigung der Hintergründe Sowie der Gesundheitlichen und Psychosexuellen Folgen für Betroffene und Partner in Eritrea und Deutschland. Humboldt University of Berlin, 2007.

7. Baldeh F. Obstetric Care in Scotland: The Experience of Women who have Undergone Female Genital Mutilation (FGM) [MSc Thesis]. Queen Margaret University, 2013.

8. Ballesteros Meseguer C, Almansa Martinez P, Pastor Bravo MdM, et al. [The voice of women subjected to female genital mutilation in the region of Murcia, Spain] La Voz de las mujeres sometidas a mutilacion genital femenina en la region de Murcia. *Gac Sanit* 2014;28(4):287-91. doi: 10.1016/j.gaceta.2014.02.006

9. Behrendt A. Listening to African Voices: Female Genital Mutilation/Cutting among Immigrants in Hamburg: Knowledge, Attitudes and Practice. Hamburg, Germany: Plan, 2011.

10. Beine K, Fullerton J, Palinkas L, et al. Conceptions of prenatal care among Somali women in San Diego. *J Nurs Midwifery* 1995;40(4):376-81. doi: Doi 10.1016/0091-2182(95)00024-E

11. Berggren V, Bergstrom S, Edberg A-K. Being different and vulnerable: experiences of immigrant African women who have been circumcised and sought maternity care in Sweden. *J Transcult Nurs* 2006;17(1):50-7. doi: 10.1177/1043659605281981

For peer review only

12. Betts V. Transnational Gender, Sexuality and Identity Construction: Ambivalent Subjectivities of Somali Refugee Women in the GTA. MA Thesis, The University of Guelph, 2011.

13. Bravo Pastor del Mar M. [Women Subjected to Female Genital Mutilation: Knowledge for the Nursing Discipline] La Voz de las Mujeres Sometidas a Mutilación Genital Femenina: Saberes Para la Disciplina Enfermera [Tesis Doctoral]. University of Murcia, 2014.

14. Bulman KH, McCourt C. Somali refugee women's experiences of maternity care in west London: a case study. *Crit Public Health* 2002;12(4):365-80.

15. Carroll J, Epstein R, Fiscella K, et al. Caring for Somali women: implications for clinician-patient communication. *Patient Educ Couns* 2007;66(3):337-45. doi: 10.1016/j.pec.2007.01.008

16. Chalmers B, Omer-Hashi K. What Somali women say about giving birth in Canada. *J Reprod Infant Psychol* 2002;20(4):267-82. doi: 10.1080/0264683021000033183

17. Degni F, Suominen SB, El Ansari W, et al. Reproductive and maternity health care services in Finland: perceptions and experiences of Somali-born immigrant women. *Ethn Health* 2014;19(3):348-66. doi: 10.1080/13557858.2013.797567

18. d'Entremont M, Smythe L, McAra-Couper J. The sounds of silence: a hermeneutic interpretation of childbirth post excision. *Health Care Women Int* 2014;35(3):300-19. doi: 10.1080/07399332.2013.838245

19. Essén B, Johnsdotter S, Hovelius B, et al. Qualitative study of pregnancy and childbirth experiences in Somalian women resident in Sweden. *Br J Obstet Gynaecol* 2000;107(12):1507-12.

20. Fawcett L. Somali Refugee Women and their U.S. Healthcare Providers: Knowledge, Perceptions and Experiences of Childbearing [Doctor of Philosophy]. Arizona State University, 2014.

21. Gali MA. Female Circumcision: A Transcultural Study of Attitudes, Identity and Reproductive Health of East African Immigrants [Degree of Doctor of Philosophy in Psychology]. The Wright Institute, University of Berkeley, 1997.

22. Ghebre RG, Sewali B, Osman S, et al. Cervical cancer: barriers to screening in the Somali community in Minnesota. *J Immigr Minor Health* 2015;17(3):722-28. doi: 10.1007/s10903-014-0080-1

23. Glazer E. Embodiment, Pain and Circumcision in Somali-Canadian Women [M.Sc.]. University of Toronto, 2012.

24. Glover J, Liebling H, Barrett H, et al. The psychological and social impact of female genital mutilation: a holistic conceptual framework. *J Int Stud* 2017;10(2):219-38.

For peer review only

25. Guerin PB, Allotey P, Elmi FH, et al. Advocacy as a means to an end: assisting refugee women to take control of their reproductive health needs. *Women Health* 2006;43(4):7-25.

26. Hill N, Hunt E, Hyrkas K. Somali immigrant women's health care experiences and beliefs regarding pregnancy and birth in the United States. *J Transcult Nurs* 2012;23(1):72-81. doi: 10.1177/1043659611423828 [published Online First: 2011/11/05]

27. Hussein E. Women's Experiences, Perceptions and Attitudes of Female Genital Mutilation: The Bristol PEER Study. London: FORWARD, 2010.

28. Hussen MA. Services for Women with Female Genital Mutilation in Christchurch: Perspectives of Women and their Health Providers. University of Canterbury, 2014.

29. Johansen RE. Undoing female genital cutting: perceptions and experiences of infibulation, defibulation and virginity among Somali and Sudanese migrants in Norway. *Cult Health Sex* 2017;19(4):528-42. doi: 10.1080/13691058.2016.1239838

30. Jones A. Working Psychologically with Female Genital Mutilation: An Exploration of the Views and Experiences of Women who have Experienced FGM and of Clinical Psychologists. University of East London, 2010.

31. Khaja K. Female Circumcision: Life Histories of Somali Women [Doctor of Philosophy]. The University of Utah, 2004.

32. Khaja K, Lay K, Boys S. Female circumcision: toward an inclusive practice of care. *Health Care Women Int* 2010;31(8):686-99. doi: 10.1080/07399332.2010.490313

33. Lundberg PC, Gerezgiher A. Experiences from pregnancy and childbirth related to female genital mutilation among Eritrean immigrant women in Sweden. *Midwifery* 2008;24(2):214-25. doi: 10.1016/j.midw.2006.10.003

34. Maier C. [Echoes of Silence: Voices of Concern for Genital Mutilation in African Immigrant Women in Vienna: An Ethnological Study] Echo des Schweigens: Stimmen der Betroffenheit zur Genitalverstümmelung bei Afrikanischen Immigrantinnen in Wien: Ethnologische Studie. University of Vienna, 2003.

35. Maternity Action. Women's Voices on Health: Addressing Barriers to Accessing Primary Care. London: Women’s Health and Equality Consortium, 2014.

36. McNeely S, Christie-de Jong F. Somali refugees' perspectives regarding FGM/C in the US. *Int J Migr Health Soc Care* 2016;12(3):157-69. doi: 10.1108/Ijmhsc-09-2015-0033

37. Moxey JM, Jones LL. A qualitative study exploring how Somali women exposed to female genital mutilation experience and perceive antenatal and intrapartum care in England. *BMJ Open* 2016;6(1):e009846. doi: 10.1136/bmjopen-2015-009846

For peer review only

38. Murray L, Windsor C, Parker E, et al. The experiences of African women giving birth in Brisbane, Australia. *Health Care Women Int* 2010;31(5):458-72. doi: 10.1080/07399330903548928

39. Norman K, Gegzabher SB, Otoo-Oyortey N. "Between Two Cultures": A Rapid PEER Study Exploring Migrant Communities' Views on Female Genital Mutilation in Essex and Norfolk, UK. FORWARD & National FGM Centre Report, , 2016.

40. Norman K, Hemmings J, Hussein E, et al. "FGM is Always With Us": Experiences, Perceptions and Beliefs of Women Affected by Female Genital Mutilation in London: Results from a PEER Study. London: Options Consultancy Services and FORWARD, 2009.

41. O'Brien O, Baldeh F, Hassan J, et al. My Voice: Participatory Action Research Project with Men, Women and Young People on Female Genital Mutilation (FGM) in Scotland: (Phase 2). Waverly Care, Edinburgh: Queen Margaret University, 2017.

42. O'Brien O, Baldeh F, Sivapatham S, et al. Participatory Action Research Project with Men, Women and Young People on Female Genital Mutilation (FGM) in Scotland: (Phase 1). Waverly Care, Edinburgh: Queen Margaret University, 2016.

43. Palfreyman A, Brown E, Nam S. Understanding Female Genital Mutilation in Birmingham: Findings from a PEER Study: Options Consultancy Services and Birmingham & Solihull Women's Aid, 2011.

44. Recchia N, McGarry J. "Don't judge me": narratives of living with FGM. *Int J Hum Rights Healthc* 2017;10(1):4-13. doi: 10.1108/Ijhrh-10-2016-0016

45. Safari F. A qualitative study of women's lived experience after deinfibulation in the UK. *Midwifery* 2013;29(2):154-8. doi: 10.1016/j.midw.2011.12.005

46. Salad J, Verdonk P, de Boer F, et al. "A Somali girl is Muslim and does not have premarital sex - is vaccination really necessary?" A qualitative study into the perceptions of Somali women in the Netherlands about the prevention of cervical cancer. *Int J Equity Health* 2015;14(1):1-13. doi: 10.1186/s12939-015-0198-3

47. Shaw E. Female circumcision: perceptions of clients and caregivers. *J Am Coll Health* 1985;33(5):193-7. doi: 10.1080/07448481.1985.9939604

48. Shermarke MAA. Understanding the Canadian Community Context of Female Circumcision [M.S.W.]. McGill University, 1996.

49. Straus L, McEwen A, Hussein FM. Somali women's experience of childbirth in the UK: perspectives from Somali health workers. *Midwifery* 2009;25(2):181-6. doi: 10.1016/j.midw.2007.02.002

50. Thierfelder C. Female Genital Mutilation and the Swiss Health Care System. University of Basel, 2003.

For peer review only

51. Thierfelder C, Tanner M, Bodiang CMK. Female genital mutilation in the context of migration: experience of African women with the Swiss health care system. *Eur J Public Health* 2005;15(1):86-90. doi: 10.1093/eurpub/cki120

52. Upvall MJ, Mohammed K, Dodge PD. Perspectives of Somali Bantu refugee women living with circumcision in the United States: a focus group approach. *Int J Nurs Stud* 2009;46(3):360-8. doi: 10.1016/j.ijnurstu.2008.04.009

53. Vangen S, Johansen REB, Sundby J, et al. Qualitative study of perinatal care experiences among Somali women and local health care professionals in Norway. *Eur J Obstet Gynecol Reprod Biol* 2004;112(1):29-35. doi: 10.1016/S0301-2115(03)00313-0

54. Vaughan C, White N, Keogh L, et al. Listening to North Yarra Communities about Female Genital Cutting. Melbourne, Australia: The University of Melbourne, 2014.

55. Vaughan C, White N, Keogh L, et al. Female Genital Mutilation/Cutting in Regional Victoria. Research to Practice. Melbourne, Australia: The University of Melbourne, 2014.

56. Vloeberghs E, van der Kwaak A, Knipscheer J, et al. Coping and chronic psychosocial consequences of female genital mutilation in the Netherlands. *Ethn Health* 2012;17(6):677-95. doi: 10.1080/13557858.2013.771148

57. Wiklund H, Aden AS, Högberg U, et al. Somalis giving birth in Sweden: a challenge to culture and gender specific values and behaviours. *Midwifery* 2000;16(2):105-15. doi: http://dx.doi.org/10.1054/midw.1999.0197

For peer review only

Supplementary File 4: Full Quality Assessment

No	Reference	Q1	Q2	Q3	Q4	Q5	Q6	Q7	Q8	Q9	Q10	Score	Quality Band	Richness: Thick or Thin	Type of Publication	Relevance: High, Med or Low
**1.**	Abdi, R. (2012)[1]	Y	Y	Y	N	Y	Y	Y	N	Y	Y	**8**	High	Thin	Journal	Low
**2.**	Abdullahi, A., Copping, J., Kessel, A., Luck, M., & Bonell, C. (2009)[2]	N	U	Y	Y	Y	N	N	Y	Y	Y	**6**	Med	Thin	Journal	Med
**3.**	Ahlberg, B. M., Krantz, I., Lindmark, G., & Warsame, M. (2004)[3]	U	Y	Y	Y	Y	Y	Y	Y	Y	Y	**9**	High	Thin	Journal	Low
**4.**	Ahmed, M., (2005)[4]	N	N	U	U	Y	N	N	P	Y	Y	**3.5**	Low	Thin	Book chapter	Low
**5.**	Ameresekere, M., Borg, R., Frederick, J., Vragovic, O., Saia, K., & Raj, A. (2011)[5]	U	Y	Y	Y	Y	N	N	P	Y	Y	**6.5**	Med	Thin	Journal	Med
**6.**	Asefaw, F. (2007)[6]	N	U	U	N	Y	Y	Y	Y	Y	Y	**6**	Med	Thin	PhD Thesis	Low
**7.**	Baldeh, F. (2013)[7]	U	Y	Y	Y	Y	Y	P	Y	Y	Y	**8.5**	High	Thick	MSc Thesis	High
**8.**	Ballesteros Meseguer, C., Almansa Martinez, P., Pastor Bravo, M. d. M., & Jimenez Ruiz, I. (2014)[8]	Y	Y	Y	N	Y	N	N	P	Y	Y	**6.5**	Med	Thin	Journal	Med
**9.**	Behrendt, A. (2011)[9]	U	Y	Y	Y	U	N	N	P	Y	Y	**5.5**	Med	Thin	Research Report	Low
**10.**	Beine, K., Fullerton, J., Palinkas, L., & Anders, B. (1995)[10]	N	U	U	Y	Y	P	N	Y	Y	Y	**5.5**	Med	Thin	Journal	Med
**11.**	Berggren, V., Bergstrom, S., & Edberg, A.-K. (2006)[11]	N	U	Y	Y	Y	N	N	Y	Y	Y	**6**	Med	Thick	Journal	High
**12.**	Betts, V. (2011)[12]	U	U	U	N	Y	Y	P	Y	Y	Y	**5.5**	Med	Thick	MA Thesis	High
**13.**	Bravo Pastor del Mar, M. (2014)[13]	Y	Y	Y	Y	Y	Y	N	P	Y	U	**7.5**	High	Thin	PhD Thesis	Low
**14.**	Bulman, K. H., & McCourt, C. (2002)[14]	N	N	Y	Y	P	P	Y	P	Y	U	**5.5**	Med	Thin	Journal	Med
**15.**	Carroll, J., Epstein, R., Fiscella, K., Gipson, T., Volpe, E., & Jean-Pierre, P. (2007)[15]	Y	Y	Y	P	Y	N	P	Y	Y	Y	**8**	High	Thick	Journal	Med
**16.**	Chalmers, B., & Omer-Hashi, K. (2002)[16]	N	U	U	U	Y	P	P	Y	Y	Y	**5**	Med	Thick	Journal	Med

For peer review only - http://bmjopen.bmj.com/site/about/guidelines.xhtml

No	Reference	Q1	Q2	Q3	Q4	Q5	Q6	Q7	Q8	Q9	Q10	Score	Quality Band	Richness: Thick or Thin	Type of Publication	Relevance: High, Med or Low
**17.**	Degni, F., Suominen, S. B., El Ansari, W., Vehviläinen-Julkunen, K., & Essen, B. (2014)[17]	U	Y	Y	Y	Y	P	P	Y	Y	Y	**8**	High	Thin	Journal	Med
**18.**	d'Entremont, M., Smythe, L., & McAra-Couper, J. (2014)[18]	Y	Y	Y	Y	Y	U	P	Y	Y	Y	**8.5**	High	Thick	Journal	High
**19.**	Essén, B., Johnsdotter, S., Hovelius, B., Gudmundsson, S., Sjöberg, N. O., Friedman, J., & Östergren, P. O. (2000)[19]	U	U	U	N	Y	Y	P	P	Y	Y	**5**	Med	Thin	Journal	Low
**20.**	Fawcett, L., (2014)[20]	Y	Y	Y	U	Y	Y	Y	Y	Y	Y	**9**	High	Thick	PhD Thesis	High
**21.**	Gali, M. A. (1997)[21]	U	U	U	U	Y	Y	N	Y	Y	Y	**5**	Med	Thin	PhD Thesis	Low
**22.**	Ghebre, R. G., Sewali, B., Osman, S., Adawe, A., Nguyen, H. T., Okuyemi, K. S., & Joseph, A. (2015)[22]	Y	Y	Y	Y	Y	N	N	P	Y	Y	**7.5**	High	Thick	Journal	Med
**23.**	Glazer, E., (2012)[23]	Y	Y	Y	Y	Y	P	N	Y	Y	Y	**8.5**	High	Thin	MSc Thesis	Low
**24.**	Glover, J., Liebling, H., Barrett, H., & Goodman, S. (2017)[24]	Y	Y	Y	Y	Y	N	N	Y	Y	Y	**8**	High	Thick	Journal	High
**25.**	Guerin, P. B., Allotey, P., Elmi, F. H., & Baho, S. (2006)[25]	U	U	U	Y	P	N	P	N	Y	Y	**4**	Low	Thin	Journal	Low
**26.**	Hill, N., Hunt, E., & Hyrkas, K. (2012)[26]	N	U	U	Y	Y	Y	N	Y	Y	Y	**6**	Med	Thick	Journal	Low
**27.**	Hussein, E., (2010)[27]	Y	Y	Y	N	Y	N	P	Y	N	Y	**6.5**	Med	Thin	Research Report	Low
**28.**	Hussen, M. A. (2014)[28]	N	U	Y	Y	Y	Y	P	Y	U	Y	**6.5**	Med	Thick	MSc Thesis	Med
**29.**	Johansen, R.E. (2017)[29]	U	U	Y	Y	Y	Y	P	Y	Y	Y	**7.5**	High	Thick	Journal	Med
**30.**	Jones, A. (2010)[30]	Y	Y	Y	Y	N	P	P	Y	Y	Y	**8**	High	Thick	PhD Thesis	High
**31.**	Khaja, K. (2004)[31]	U	Y	Y	Y	Y	P	N	Y	Y	Y	**7.5**	High	Thin	PhD Thesis	Low
**32.**	Khaja, K., Lay, K., & Boys, S. (2010)[32]	N	U	Y	N	U	N	N	P	N	Y	**2.5**	Low	Thin	Journal	Low
**33.**	Lundberg, P. C., & Gerezgiher, A. (2008)[33]	U	Y	Y	Y	Y	P	P	Y	Y	Y	**8**	High	Thick	Journal	High
**34.**	Maier, C. (2003)[34]	N	N	N	N	Y	Y	N	Y	U	Y	**4**	Low	Thin	PhD Thesis	Low

For peer review only - http://bmjopen.bmj.com/site/about/guidelines.xhtml

No	Reference	Q1	Q2	Q3	Q4	Q5	Q6	Q7	Q8	Q9	Q10	Score	Quality Band	Richness: Thick or Thin	Type of Publication	Relevance: High, Med or Low
**35.**	Maternity Action. (2014)[35]	U	U	Y	U	Y	N	P	Y	U	U	**3.5**	Low	Thin	Research Report	Med
**36.**	McNeely, S., & Christie-de Jong, F. (2016)[36]	U	Y	Y	Y	Y	Y	P	N	Y	Y	**7.5**	High	Thin	Journal	Low
**37.**	Moxey, J. M., & Jones, L. L. (2016)[37]	U	Y	Y	Y	Y	Y	P	Y	Y	Y	**8.5**	High	Thick	Journal	High
**38.**	Murray, L., Windsor, C., Parker, E., & Tewfik, O. (2010)[38]	Y	Y	Y	Y	Y	Y	P	Y	Y	Y	**9.5**	High	Thick	Journal	Med
**39.**	Norman, K., Gegzabher, S. B., & Otoo-Oyortey, N. (2016)[39]	Y	Y	Y	U	U	P	P	Y	N	Y	**6**	Med	Thin	Research Report	Low
**40.**	Norman, K., Hemmings, J., Hussein, E., & Otoo-Oyortey, N. (2009)[40]	Y	Y	Y	U	U	P	P	Y	N	Y	**6**	Med	Thin	Research Report	Low
**41.**	O'Brien, O., Baldeh, F., Hassan, J., & Baillie, M. (2017)[41]	Y	Y	Y	U	Y	P	P	Y	Y	Y	**8**	High	Thin	Research Report	Low
**42.**	O'Brien, O., Baldeh, F., Sivapatham, S., Brown, E., & O'May, F. (2016)[42]	Y	Y	Y	N	Y	P	P	Y	Y	Y	**8**	High	Thin	Research Report	Low
**43.**	Palfreyman, A., Brown, E., & Nam, S. (2011)[43]	N	Y	Y	Y	Y	P	P	Y	Y	Y	**8**	High	Thick	Research Report	High
**44.**	Recchia, N., & McGarry, J. (2017)[44]	U	Y	Y	U	Y	N	N	Y	U	Y	**5**	Med	Thick	Journal	Low
**45.**	Safari, F., (2013)[45]	Y	Y	Y	Y	Y	N	P	Y	Y	Y	**8.5**	High	Thin	Journal	Low
**46.**	Salad, J., Verdonk, P., de Boer, F., & Abma, T. A. (2015)[46]	U	U	Y	Y	Y	Y	P	Y	Y	Y	**7.5**	High	Thick	Journal	Med
**47.**	Shaw, E. (1985)[47]	N	U	U	Y	Y	N	P	Y	U	P	**4**	Low	Thin	Journal	Low
**48.**	Shermarke, M.A.A. (1996)[48]	Y	Y	Y	Y	Y	Y	Y	Y	U	Y	**9**	High	Thin	MSc Thesis	Med
**49.**	Straus, L., McEwen, A., & Hussein, F. M. (2009)[49]	U	Y	Y	Y	Y	Y	P	Y	Y	Y	**8.5**	High	Thick	Journal	High
**50.**	Thierfelder, C. (2003)[50]	N	N	Y	Y	Y	Y	P	Y	Y	Y	**7.5**	High	Thick	PhD Thesis	High
**51.**	Thierfelder, C., Tanner, M., & Bodiang, C. M. K. (2005)[51]	N	N	Y	U	Y	P	P	Y	Y	Y	**6**	Med	Thin	Journal	Med
**52.**	Upvall, M. J., Mohammed, K., & Dodge, P. D. (2009)[52]	N	N	Y	Y	Y	P	P	Y	Y	Y	**7**	Med	Thick	Journal	High
**53.**	Vangen, S., Johansen, R. E. B., Sundby, J., Traeen, B., & Stray-Pedersen, B. (2004)[53]	Y	Y	Y	Y	Y	P	N	P	Y	Y	**8**	High	Thick	Journal	High

For peer review only - http://bmjopen.bmj.com/site/about/guidelines.xhtml

No	Reference	Q1	Q2	Q3	Q4	Q5	Q6	Q7	Q8	Q9	Q10	Score	Quality Band	Richness: Thick or Thin	Type of Publication	Relevance: High, Med or Low
**54.**	Vaughan, C., White, N., Keogh, L., Tobin, J., Ha, B., Ibrahim, M., & Bayly, C. (2014)[54]	U	Y	Y	Y	Y	Y	P	Y	Y	Y	**8.5**	High	Thick	Research Report	High
**55.**	Vaughan, C., White, N., Keogh, L., Tobin, J., Murdolo, A., Quiazon, R., & Bayly, C. (2014)[55]	U	Y	Y	Y	Y	Y	P	Y	Y	Y	**8.5**	High	Thick	Research Report	High
**56.**	Vloeberghs, E., van der Kwaak, A., Knipscheer, J., & van den Muijsenbergh, M. (2012)[56]	Y	Y	Y	Y	Y	P	P	Y	Y	Y	**9**	High	Thin	Journal	Med
**57.**	Wiklund, H., Aden, A. S., Högberg, U., Wikman, M., & Dahlgren, L. (2000)[57]	Y	Y	U	Y	Y	N	N	Y	Y	Y	**7**	Med	Thin	Journal	Med

For peer review only

References of Studies Included in Quality Assessment

1. Abdi R. Carving culture: Creating identity through female genital cutting. *Durham Anthropology Journal* 2012;18(1):115-53.

2. Abdullahi A, Copping J, Kessel A, et al. Cervical screening: perceptions and barriers to uptake among Somali women in Camden. *Public Health* 2009;123(10):680-5. doi: 10.1016/j.puhe.2009.09.011

3. Ahlberg BM, Krantz I, Lindmark G, et al. 'It's only a tradition': making sense of eradication interventions and the persistence of female 'circumcision' within a Swedish context. *Crit Soc Policy* 2004;24(1):50-78. doi: 10.1107/0261018304039679

4. Ahmed M. Attitudes towards FGM among Somali women living in the UK. In: Momoh C, ed. Female Genital Mutilation. London: Radcliffe 2005:95-117.

5. Ameresekere M, Borg R, Frederick J, et al. Somali immigrant women's perceptions of cesarean delivery and patient-provider communication surrounding female circumcision and childbirth in the USA. *Int J Gynecol Obstet* 2011;115(3):227-30. doi: 10.1016/j.ijgo.2011.07.019

6. Asefaw F. [Female Genital Mutilation: A Field Study with Special Consideration of the Background as well as the Health and Psychosexual Consquences for those Affected and their Partners in Eritrea and in Germany] Weibliche Genitalbeschneidung: Eine Feldstudie Unter besonderer Berücksichtigung der Hintergründe Sowie der Gesundheitlichen und Psychosexuellen Folgen für Betroffene und Partner in Eritrea und Deutschland. Humboldt University of Berlin, 2007.

7. Baldeh F. Obstetric Care in Scotland: The Experience of Women who have Undergone Female Genital Mutilation (FGM) [MSc Thesis]. Queen Margaret University, 2013.

8. Ballesteros Meseguer C, Almansa Martinez P, Pastor Bravo MdM, et al. [The voice of women subjected to female genital mutilation in the region of Murcia, Spain] La Voz de las mujeres sometidas a mutilacion genital femenina en la region de Murcia. *Gac Sanit* 2014;28(4):287-91. doi: 10.1016/j.gaceta.2014.02.006

9. Behrendt A. Listening to African Voices: Female Genital Mutilation/Cutting among Immigrants in Hamburg: Knowledge, Attitudes and Practice. Hamburg, Germany: Plan, 2011.

10. Beine K, Fullerton J, Palinkas L, et al. Conceptions of prenatal care among Somali women in San Diego. *J Nurs Midwifery* 1995;40(4):376-81. doi: Doi 10.1016/0091-2182(95)00024-E

11. Berggren V, Bergstrom S, Edberg A-K. Being different and vulnerable: experiences of immigrant African women who have been circumcised and sought maternity care in Sweden. *J Transcult Nurs* 2006;17(1):50-7. doi: 10.1177/1043659605281981

12. Betts V. Transnational Gender, Sexuality and Identity Construction: Ambivalent Subjectivities of Somali Refugee Women in the GTA. MA Thesis, The University of Guelph, 2011.

13. Bravo Pastor del Mar M. [Women Subjected to Female Genital Mutilation: Knowledge for the Nursing Discipline] La Voz de las Mujeres Sometidas a Mutilación Genital Femenina: Saberes Para la Disciplina Enfermera [Tesis Doctoral]. University of Murcia, 2014.

14. Bulman KH, McCourt C. Somali refugee women's experiences of maternity care in west London: a case study. *Crit Public Health* 2002;12(4):365-80.

15. Carroll J, Epstein R, Fiscella K, et al. Caring for Somali women: implications for clinician-patient communication. *Patient Educ Couns* 2007;66(3):337-45. doi: 10.1016/j.pec.2007.01.008

16. Chalmers B, Omer-Hashi K. What Somali women say about giving birth in Canada. *J Reprod Infant Psychol* 2002;20(4):267-82. doi: 10.1080/0264683021000033183

17. Degni F, Suominen SB, El Ansari W, et al. Reproductive and maternity health care services in Finland: perceptions and experiences of Somali-born immigrant women. *Ethn Health* 2014;19(3):348-66. doi: 10.1080/13557858.2013.797567

18. d'Entremont M, Smythe L, McAra-Couper J. The sounds of silence: a hermeneutic interpretation of childbirth post excision. *Health Care Women Int* 2014;35(3):300-19. doi: 10.1080/07399332.2013.838245

19. Essén B, Johnsdotter S, Hovelius B, et al. Qualitative study of pregnancy and childbirth experiences in Somalian women resident in Sweden. *Br J Obstet Gynaecol* 2000;107(12):1507-12.

20. Fawcett L. Somali Refugee Women and their U.S. Healthcare Providers: Knowledge, Perceptions and Experiences of Childbearing [Doctor of Philosophy]. Arizona State University, 2014.

21. Gali MA. Female Circumcision: A Transcultural Study of Attitudes, Identity and Reproductive Health of East African Immigrants [Degree of Doctor of Philosophy in Psychology]. The Wright Institute, University of Berkeley, 1997.

22. Ghebre RG, Sewali B, Osman S, et al. Cervical cancer: barriers to screening in the Somali community in Minnesota. *J Immigr Minor Health* 2015;17(3):722-28. doi: 10.1007/s10903-014-0080-1

23. Glazer E. Embodiment, Pain and Circumcision in Somali-Canadian Women [M.Sc.]. University of Toronto, 2012.

24. Glover J, Liebling H, Barrett H, et al. The psychological and social impact of female genital mutilation: a holistic conceptual framework. *J Int Stud* 2017;10(2):219-38.

For peer review only

25. Guerin PB, Allotey P, Elmi FH, et al. Advocacy as a means to an end: assisting refugee women to take control of their reproductive health needs. *Women Health* 2006;43(4):7-25.

26. Hill N, Hunt E, Hyrkas K. Somali immigrant women's health care experiences and beliefs regarding pregnancy and birth in the United States. *J Transcult Nurs* 2012;23(1):72-81. doi: 10.1177/1043659611423828 [published Online First: 2011/11/05]

27. Hussein E. Women's Experiences, Perceptions and Attitudes of Female Genital Mutilation: The Bristol PEER Study. London: FORWARD, 2010.

28. Hussen MA. Services for Women with Female Genital Mutilation in Christchurch: Perspectives of Women and their Health Providers. University of Canterbury, 2014.

29. Johansen RE. Undoing female genital cutting: perceptions and experiences of infibulation, defibulation and virginity among Somali and Sudanese migrants in Norway. *Cult Health Sex* 2017;19(4):528-42. doi: 10.1080/13691058.2016.1239838

30. Jones A. Working Psychologically with Female Genital Mutilation: An Exploration of the Views and Experiences of Women who have Experienced FGM and of Clinical Psychologists. University of East London, 2010.

31. Khaja K. Female Circumcision: Life Histories of Somali Women [Doctor of Philosophy]. The University of Utah, 2004.

32. Khaja K, Lay K, Boys S. Female circumcision: toward an inclusive practice of care. *Health Care Women Int* 2010;31(8):686-99. doi: 10.1080/07399332.2010.490313

33. Lundberg PC, Gerezgiher A. Experiences from pregnancy and childbirth related to female genital mutilation among Eritrean immigrant women in Sweden. *Midwifery* 2008;24(2):214-25. doi: 10.1016/j.midw.2006.10.003

34. Maier C. [Echoes of Silence: Voices of Concern for Genital Mutilation in African Immigrant Women in Vienna: An Ethnological Study] Echo des Schweigens: Stimmen der Betroffenheit zur Genitalverstümmelung bei Afrikanischen Immigrantinnen in Wien: Ethnologische Studie. University of Vienna, 2003.

35. Maternity Action. Women's Voices on Health: Addressing Barriers to Accessing Primary Care. London: Women’s Health and Equality Consortium, 2014.

36. McNeely S, Christie-de Jong F. Somali refugees' perspectives regarding FGM/C in the US. *Int J Migr Health Soc Care* 2016;12(3):157-69. doi: 10.1108/Ijmhsc-09-2015-0033

37. Moxey JM, Jones LL. A qualitative study exploring how Somali women exposed to female genital mutilation experience and perceive antenatal and intrapartum care in England. *BMJ Open* 2016;6(1):e009846. doi: 10.1136/bmjopen-2015-009846

For peer review only

38. Murray L, Windsor C, Parker E, et al. The experiences of African women giving birth in Brisbane, Australia. *Health Care Women Int* 2010;31(5):458-72. doi: 10.1080/07399330903548928

39. Norman K, Gegzabher SB, Otoo-Oyortey N. "Between Two Cultures": A Rapid PEER Study Exploring Migrant Communities' Views on Female Genital Mutilation in Essex and Norfolk, UK. FORWARD & National FGM Centre Report, , 2016.

40. Norman K, Hemmings J, Hussein E, et al. "FGM is Always With Us": Experiences, Perceptions and Beliefs of Women Affected by Female Genital Mutilation in London: Results from a PEER Study. London: Options Consultancy Services and FORWARD, 2009.

41. O'Brien O, Baldeh F, Hassan J, et al. My Voice: Participatory Action Research Project with Men, Women and Young People on Female Genital Mutilation (FGM) in Scotland: (Phase 2). Waverly Care, Edinburgh: Queen Margaret University, 2017.

42. O'Brien O, Baldeh F, Sivapatham S, et al. Participatory Action Research Project with Men, Women and Young People on Female Genital Mutilation (FGM) in Scotland: (Phase 1). Waverly Care, Edinburgh: Queen Margaret University, 2016.

43. Palfreyman A, Brown E, Nam S. Understanding Female Genital Mutilation in Birmingham: Findings from a PEER Study: Options Consultancy Services and Birmingham & Solihull Women's Aid, 2011.

44. Recchia N, McGarry J. "Don't judge me": narratives of living with FGM. *Int J Hum Rights Healthc* 2017;10(1):4-13. doi: 10.1108/Ijhrh-10-2016-0016

45. Safari F. A qualitative study of women's lived experience after deinfibulation in the UK. *Midwifery* 2013;29(2):154-8. doi: 10.1016/j.midw.2011.12.005

46. Salad J, Verdonk P, de Boer F, et al. "A Somali girl is Muslim and does not have premarital sex - is vaccination really necessary?" A qualitative study into the perceptions of Somali women in the Netherlands about the prevention of cervical cancer. *Int J Equity Health* 2015;14(1):1-13. doi: 10.1186/s12939-015-0198-3

47. Shaw E. Female circumcision: perceptions of clients and caregivers. *J Am Coll Health* 1985;33(5):193-7. doi: 10.1080/07448481.1985.9939604

48. Shermarke MAA. Understanding the Canadian Community Context of Female Circumcision [M.S.W.]. McGill University, 1996.

49. Straus L, McEwen A, Hussein FM. Somali women's experience of childbirth in the UK: perspectives from Somali health workers. *Midwifery* 2009;25(2):181-6. doi: 10.1016/j.midw.2007.02.002

50. Thierfelder C. Female Genital Mutilation and the Swiss Health Care System. University of Basel, 2003.

For peer review only

51. Thierfelder C, Tanner M, Bodiang CMK. Female genital mutilation in the context of migration: experience of African women with the Swiss health care system. *Eur J Public Health* 2005;15(1):86-90. doi: 10.1093/eurpub/cki120

52. Upvall MJ, Mohammed K, Dodge PD. Perspectives of Somali Bantu refugee women living with circumcision in the United States: a focus group approach. *Int J Nurs Stud* 2009;46(3):360-8. doi: 10.1016/j.ijnurstu.2008.04.009

53. Vangen S, Johansen REB, Sundby J, et al. Qualitative study of perinatal care experiences among Somali women and local health care professionals in Norway. *Eur J Obstet Gynecol Reprod Biol* 2004;112(1):29-35. doi: 10.1016/S0301-2115(03)00313-0

54. Vaughan C, White N, Keogh L, et al. Listening to North Yarra Communities about Female Genital Cutting. Melbourne, Australia: The University of Melbourne, 2014.

55. Vaughan C, White N, Keogh L, et al. Female Genital Mutilation/Cutting in Regional Victoria. Research to Practice. Melbourne, Australia: The University of Melbourne, 2014.

56. Vloeberghs E, van der Kwaak A, Knipscheer J, et al. Coping and chronic psychosocial consequences of female genital mutilation in the Netherlands. *Ethn Health* 2012;17(6):677-95. doi: 10.1080/13557858.2013.771148

57. Wiklund H, Aden AS, Högberg U, et al. Somalis giving birth in Sweden: a challenge to culture and gender specific values and behaviours. *Midwifery* 2000;16(2):105-15. doi: http://dx.doi.org/10.1054/midw.1999.0197

For peer review only

Supplementary File 5: Theme Matrix (Mapping Included Studies to Themes)

		1. Communication is key			2. Access to care: influenced by an interaction of multi-level community and health service processes				3. Cultural and bodily dissonance: striving for cultural and bodily integrity				4. Disempowering care encounters				5. Positive care encounters	
**No.**	**Study Reference**	**1.1**	**1.2**	**1.3**	**2.1**	**2.2**	**2.3**	**2.4**	**3.1**	**3.2**	**3.3**	**3.4**	**4.1**	**4.2**	**4.3**	**4.4**	**5.1**	**5.2**
**1**	Abdi, R. (2012)[1]			X		X				X			X					
**2**	Abdullahi, A., Copping, J., Kessel, A., Luck, M., & Bonell, C. (2009)[2]	X			X		X			X			X					X
**3**	Ahlberg, B. M., Krantz, I., Lindmark, G., & Warsame, M. (2004)[3]	X												X		X		
**4**	Ahmed, M., (2005)[4]		X	X									X					X
**5**	Ameresekere, M., Borg, R., Frederick, J., Vragovic, O., Saia, K., & Raj, A. (2011)[5]		X		X		X							X	X	X	X	X
**6**	Asefaw, F. (2007)[6]		X	X	X			X	X	X	X	X	X	X	X	X	X	X
**7**	Baldeh, F. (2013)[7]		X	X				X	X	X	X		X	X	X	X		X
**8**	Ballesteros Meseguer, C., Almansa Martinez, P., Pastor Bravo, M. d. M., & Jimenez Ruiz, I. (2014)[8]						X			X						X	X	
**9**	Behrendt, A. (2011)[9]		X	X				X	X				X	X	X	X	X	
**10**	Beine, K., Fullerton, J., Palinkas, L., & Anders, B. (1995)[10]		X	X	X						X	X	X		X	X	X	X
**11**	Berggren, V., Bergstrom, S., & Edberg, A.-K. (2006)[11]	X	X	X	X	X	X	X	X	X	X	X	X	X	X	X	X	X
**12**	Betts, V. (2011)[12]	X	X	X	X	X	X	X	X		X	X	X	X	X	X	X	X
**13**	Bravo Pastor del Mar, M. (2014)[13]		X				X	X	X		X	X	X	X		X	X	X

For peer review only

For peer review only - http://bmjopen.bmj.com/site/about/guidelines.xhtml

		1. Communication is key			2. Access to care: influenced by an interaction of multi-level community and health service processes				3. Cultural and bodily dissonance: striving for cultural and bodily integrity				4. Disempowering care encounters				5. Positive care encounters	
**No.**	**Study Reference**	**1.1**	**1.2**	**1.3**	**2.1**	**2.2**	**2.3**	**2.4**	**3.1**	**3.2**	**3.3**	**3.4**	**4.1**	**4.2**	**4.3**	**4.4**	**5.1**	**5.2**
**14**	Bulman, K. H., & McCourt, C. (2002)[14]	X		X	X		X	X						X		X	X	X
**15**	Carroll, J., Epstein, R., Fiscella, K., Gipson, T., Volpe, E., & Jean-Pierre, P. (2007)[15]	X		X	X			X						X		X		X
**16**	Chalmers, B., & Omer-Hashi, K. (2002)[16]	X		X	X		X	X	X	X			X	X	X	X	X	X
**17**	Degni, F., Suominen, S. B., El Ansari, W., Vehviläinen-Julkunen, K., & Essen, B. (2014)[17]	X	X		X	X		X	X		X	X		X			X	X
**18**	d'Entremont, M., Smythe, L., & McAra-Couper, J. (2014)[18]	X	X			X				X			X	X	X	X	X	X
**19**	Essén, B., Johnsdotter, S., Hovelius, B., Gudmundsson, S., Sjöberg, N. O., Friedman, J., & Östergren, P. O. (2000)[19]						X	X							X	X	X	
**20**	Fawcett, L., (2014)[20]	X	X	X	X	X		X	X	X			X	X	X	X		X
**21**	Gali, M. A. (1997)[21]	X		X			X	X	X	X	X	X	X	X	X	X	X	X
**22**	Ghebre, R. G., Sewali, B., Osman, S., Adawe, A., Nguyen, H. T., Okuyemi, K. S., & Joseph, A. (2015)[22]	X			X	X	X			X								
**23**	Glazer, E., (2012)[23]		X	X	X			X	X	X	X		X		X	X	X	X
**24**	Glover, J., Liebling, H., Barrett, H., & Goodman, S. (2017)[24]			X			X	X	X	X	X	X	X	X	X	X	X	X
**25**	Guerin, P. B., Allotey, P., Elmi, F. H., & Baho, S. (2006)[25]			X			X		X		X	X	X	X	X	X		X

For peer review only

		1. Communication is key			2. Access to care: influenced by an interaction of multi-level community and health service processes				3. Cultural and bodily dissonance: striving for cultural and bodily integrity				4. Disempowering care encounters				5. Positive care encounters	
**No.**	**Study Reference**	**1.1**	**1.2**	**1.3**	**2.1**	**2.2**	**2.3**	**2.4**	**3.1**	**3.2**	**3.3**	**3.4**	**4.1**	**4.2**	**4.3**	**4.4**	**5.1**	**5.2**
**26**	Hill, N., Hunt, E., & Hyrkas, K. (2012)[26]	X			X	X	X	X	X		X				X	X	X	X
**27**	Hussein, E., (2010)[27]	X	X	X	X			X	X	X			X	X		X		X
**28**	Hussen, M. A. (2014)[28]	X	X	X	X		X	X	X	X	X		X			X	X	X
**29**	Johansen, R.E. (2017)[29]				X				X		X	X						X
**30**	Jones, A. (2010)[30]		X		X	X	X		X		X			X		X	X	X
**31**	Khaja, K. (2004)[31]		X	X	X	X	X				X				X	X		
**32**	Khaja, K., Lay, K., & Boys, S. (2010)[32]								X				X	X				X
**33**	Lundberg, P. C., & Gerezgiher, A. (2008)[33]		X		X	X		X	X	X	X	X			X	X	X	X
**34**	Maier, C. (2003)[34]				X	X	X	X	X	X	X	X	X	X	X	X	X	X
**35**	Maternity Action. (2014)[35]	X	X	X	X			X	X				X	X	X	X		X
**36**	McNeely, S., & Christie-de Jong, F. (2016)[36]		X					X	X	X			X			X		X
**37**	Moxey, J. M., & Jones, L. L. (2016)[37]	X	X		X		X	X		X	X		X		X	X	X	X
**38**	Murray, L., Windsor, C., Parker, E., & Tewfik, O. (2010)[38]	X	X	X	X		X	X	X				X	X	X	X	X	
**39**	Norman, K., Gegzabher, S. B., & Otoo-Oyortey, N. (2016)[39]		X	X			X	X					X	X				X
**40**	Norman, K., Hemmings, J., Hussein, E., & Otoo-Oyortey, N. (2009)[40]	X	X	X	X		X	X	X	X	X		X	X	X	X	X	X

For peer review only

		1. Communication is key			2. Access to care: influenced by an interaction of multi-level community and health service processes				3. Cultural and bodily dissonance: striving for cultural and bodily integrity				4. Disempowering care encounters				5. Positive care encounters	
**No.**	**Study Reference**	**1.1**	**1.2**	**1.3**	**2.1**	**2.2**	**2.3**	**2.4**	**3.1**	**3.2**	**3.3**	**3.4**	**4.1**	**4.2**	**4.3**	**4.4**	**5.1**	**5.2**
**41**	O'Brien, O., Baldeh, F., Hassan, J., & Baillie, M. (2017)[41]	X	X				X	X	X	X	X	X	X		X	X	X	X
**42**	O'Brien, O., Baldeh, F., Sivapatham, S., Brown, E., & O'May, F. (2016)[42]	X	X	X			X	X	X				X			X	X	X
**43**	Palfreyman, A., Brown, E., & Nam, S. (2011)[43]	X	X	X			X	X	X		X	X	X	X		X	X	X
**44**	Recchia, N., & McGarry, J. (2017)[44]		X	X									X	X		X		X
**45**	Safari, F., (2013)[45]		X		X	X			X		X	X						
**46**	Salad, J., Verdonk, P., de Boer, F., & Abma, T. A. (2015)[46]	X	X		X	X	X		X	X			X			X		
**47**	Shaw, E. (1985)[47]						X	X	X	X	X	X				X		X
**48**	Shermarke, M.A.A. (1996)[48]		X					X	X					X	X	X	X	X
**49**	Straus, L., McEwen, A., & Hussein, F. M. (2009)[49]	X		X	X	X	X	X	X		X		X	X	X	X		X
**50**	Thierfelder, C. (2003)[50]			X								X						
**51**	Thierfelder, C., Tanner, M., & Bodiang, C. M. K. (2005)[51]	X	X		X		X	X	X	X	X		X	X	X	X		X
**52**	Upvall, M. J., Mohammed, K., & Dodge, P. D. (2009)[52]	X	X	X	X	X	X		X				X	X		X	X	X
**53**	Vangen, S., Johansen, R. E. B., Sundby, J., Traeen, B., & Stray-Pedersen, B. (2004)[53]	X	X	X	X	X	X	X	X	X			X	X	X	X	X	

For peer review only

		1. Communication is key			2. Access to care: influenced by an interaction of multi-level community and health service processes				3. Cultural and bodily dissonance: striving for cultural and bodily integrity				4. Disempowering care encounters				5. Positive care encounters	
**No.**	**Study Reference**	**1.1**	**1.2**	**1.3**	**2.1**	**2.2**	**2.3**	**2.4**	**3.1**	**3.2**	**3.3**	**3.4**	**4.1**	**4.2**	**4.3**	**4.4**	**5.1**	**5.2**
**54**	Vaughan, C., White, N., Keogh, L., Tobin, J., Ha, B., Ibrahim, M., & Bayly, C. (2014)[54]	X	X	X	X	X	X	X	X	X	X	X	X	X	X	X	X	X
**55**	Vaughan, C., White, N., Keogh, L., Tobin, J., Murdolo, A., Quiazon, R., & Bayly, C. (2014)[55]	X	X	X	X		X	X	X	X	X	X	X	X	X	X	X	X
**56**	Vloeberghs, E., van der Kwaak, A., Knipscheer, J., & van den Muijsenbergh, M. (2012)[56]	X	X	X	X			X	X	X			X	X	X		X	X
**57**	Wiklund, H., Aden, A. S., Högberg, U., Wikman, M., & Dahlgren, L. (2000)[57]	X		X	X	X	X	X	X				X	X	X	X	X	X
	**Total Studies per Theme**	**31**	**37**	**34**	**35**	**19**	**34**	**38**	**40**	**28**	**28**	**19**	**40**	**36**	**32**	**46**	**34**	**45**

For peer review only

For peer review only - http://bmjopen.bmj.com/site/about/guidelines.xhtml

Theme Key – for Theme Matrix

Descriptive Theme No.	Theme Name
**Analytical Theme 1: Communication is key**	
**1.1**	Language barriers and interpretation challenges
**1.2**	"Can't talk, not asked": double silence and cultural taboo
**1.3**	Cultural (in)sensitivity
**Analytical Theme 2: Access to care: influenced by an interaction of multi-level community and health service processes**	
**2.1**	Influence of cultural norms
**2.2**	Influence of the family
**2.3**	Knowledge and information about FGM/C services
**2.4**	'Hit and miss' care
**Analytical Theme 3: Cultural and bodily dissonance: striving for cultural and bodily integrity**	
**3.1**	Moving from normal to different
**3.2**	Threat to the self: re-living FGM/C pain during clinical interventions
**3.3**	Being opened: complexities around deinfibulation
**3.4**	Being changed: complexities around re-infibulation
**Analytical Theme 4: Disempowering care encounters**	
**4.1**	Being exposed and humiliated
**4.2**	Being judged and stereotyped
**4.3**	Lacking choice, power and control
**4.4**	Feeling unsafe and vulnerable
**Analytical Theme 5: Positive care encounters**	
**5.1**	Trusting and appreciating providers and the system
**5.2**	Voicing healthcare needs and preferences

For peer review only

References of Included Studies - Cited in Theme Matrix

1. Abdi R. Carving culture: Creating identity through female genital cutting. *Durham Anthropology Journal* 2012;18(1):115-53.

2. Abdullahi A, Copping J, Kessel A, et al. Cervical screening: perceptions and barriers to uptake among Somali women in Camden. *Public Health* 2009;123(10):680-5. doi: 10.1016/j.puhe.2009.09.011

3. Ahlberg BM, Krantz I, Lindmark G, et al. 'It's only a tradition': making sense of eradication interventions and the persistence of female 'circumcision' within a Swedish context. *Crit Soc Policy* 2004;24(1):50-78. doi: 10.1107/0261018304039679

4. Ahmed M. Attitudes towards FGM among Somali women living in the UK. In: Momoh C, ed. Female Genital Mutilation. London: Radcliffe 2005:95-117.

5. Ameresekere M, Borg R, Frederick J, et al. Somali immigrant women's perceptions of cesarean delivery and patient-provider communication surrounding female circumcision and childbirth in the USA. *Int J Gynecol Obstet* 2011;115(3):227-30. doi: 10.1016/j.ijgo.2011.07.019

6. Asefaw F. [Female Genital Mutilation: A Field Study with Special Consideration of the Background as well as the Health and Psychosexual Consquences for those Affected and their Partners in Eritrea and in Germany] Weibliche Genitalbeschneidung: Eine Feldstudie Unter besonderer Berücksichtigung der Hintergründe Sowie der Gesundheitlichen und Psychosexuellen Folgen für Betroffene und Partner in Eritrea und Deutschland. Humboldt University of Berlin, 2007.

7. Baldeh F. Obstetric Care in Scotland: The Experience of Women who have Undergone Female Genital Mutilation (FGM) [MSc Thesis]. Queen Margaret University, 2013.

8. Ballesteros Meseguer C, Almansa Martinez P, Pastor Bravo MdM, et al. [The voice of women subjected to female genital mutilation in the region of Murcia, Spain] La Voz de las mujeres sometidas a mutilacion genital femenina en la region de Murcia. *Gac Sanit* 2014;28(4):287-91. doi: 10.1016/j.gaceta.2014.02.006

9. Behrendt A. Listening to African Voices: Female Genital Mutilation/Cutting among Immigrants in Hamburg: Knowledge, Attitudes and Practice. Hamburg, Germany: Plan, 2011.

10. Beine K, Fullerton J, Palinkas L, et al. Conceptions of prenatal care among Somali women in San Diego. *J Nurs Midwifery* 1995;40(4):376-81. doi: Doi 10.1016/0091-2182(95)00024-E

11. Berggren V, Bergstrom S, Edberg A-K. Being different and vulnerable: experiences of immigrant African women who have been circumcised and sought maternity care in Sweden. *J Transcult Nurs* 2006;17(1):50-7. doi: 10.1177/1043659605281981

For peer review only

12. Betts V. Transnational Gender, Sexuality and Identity Construction: Ambivalent Subjectivities of Somali Refugee Women in the GTA. MA Thesis, The University of Guelph, 2011.

13. Bravo Pastor del Mar M. [Women Subjected to Female Genital Mutilation: Knowledge for the Nursing Discipline] La Voz de las Mujeres Sometidas a Mutilación Genital Femenina: Saberes Para la Disciplina Enfermera [Tesis Doctoral]. University of Murcia, 2014.

14. Bulman KH, McCourt C. Somali refugee women's experiences of maternity care in west London: a case study. *Crit Public Health* 2002;12(4):365-80.

15. Carroll J, Epstein R, Fiscella K, et al. Caring for Somali women: implications for clinician-patient communication. *Patient Educ Couns* 2007;66(3):337-45. doi: 10.1016/j.pec.2007.01.008

16. Chalmers B, Omer-Hashi K. What Somali women say about giving birth in Canada. *J Reprod Infant Psychol* 2002;20(4):267-82. doi: 10.1080/026468302100033183

17. Degni F, Suominen SB, El Ansari W, et al. Reproductive and maternity health care services in Finland: perceptions and experiences of Somali-born immigrant women. *Ethn Health* 2014;19(3):348-66. doi: 10.1080/13557858.2013.797567

18. d'Entremont M, Smythe L, McAra-Couper J. The sounds of silence: a hermeneutic interpretation of childbirth post excision. *Health Care Women Int* 2014;35(3):300-19. doi: 10.1080/07399332.2013.838245

19. Essén B, Johnsdotter S, Hovelius B, et al. Qualitative study of pregnancy and childbirth experiences in Somalian women resident in Sweden. *Br J Obstet Gynaecol* 2000;107(12):1507-12.

20. Fawcett L. Somali Refugee Women and their U.S. Healthcare Providers: Knowledge, Perceptions and Experiences of Childbearing [Doctor of Philosophy]. Arizona State University, 2014.

21. Gali MA. Female Circumcision: A Transcultural Study of Attitudes, Identity and Reproductive Health of East African Immigrants [Degree of Doctor of Philosophy in Psychology]. The Wright Institute, University of Berkeley, 1997.

22. Ghebre RG, Sewali B, Osman S, et al. Cervical cancer: barriers to screening in the Somali community in Minnesota. *J Immigr Minor Health* 2015;17(3):722-28. doi: 10.1007/s10903-014-0080-1

23. Glazer E. Embodiment, Pain and Circumcision in Somali-Canadian Women [M.Sc.]. University of Toronto, 2012.

24. Glover J, Liebling H, Barrett H, et al. The psychological and social impact of female genital mutilation: a holistic conceptual framework. *J Int Stud* 2017;10(2):219-38.

For peer review only

25. Guerin PB, Allotey P, Elmi FH, et al. Advocacy as a means to an end: assisting refugee women to take control of their reproductive health needs. *Women Health* 2006;43(4):7-25.

26. Hill N, Hunt E, Hyrkas K. Somali immigrant women's health care experiences and beliefs regarding pregnancy and birth in the United States. *J Transcult Nurs* 2012;23(1):72-81. doi: 10.1177/1043659611423828 [published Online First: 2011/11/05]

27. Hussein E. Women's Experiences, Perceptions and Attitudes of Female Genital Mutilation: The Bristol PEER Study. London: FORWARD, 2010.

28. Hussen MA. Services for Women with Female Genital Mutilation in Christchurch: Perspectives of Women and their Health Providers. University of Canterbury, 2014.

29. Johansen RE. Undoing female genital cutting: perceptions and experiences of infibulation, defibulation and virginity among Somali and Sudanese migrants in Norway. *Cult Health Sex* 2017;19(4):528-42. doi: 10.1080/13691058.2016.1239838

30. Jones A. Working Psychologically with Female Genital Mutilation: An Exploration of the Views and Experiences of Women who have Experienced FGM and of Clinical Psychologists. University of East London, 2010.

31. Khaja K. Female Circumcision: Life Histories of Somali Women [Doctor of Philosophy]. The University of Utah, 2004.

32. Khaja K, Lay K, Boys S. Female circumcision: toward an inclusive practice of care. *Health Care Women Int* 2010;31(8):686-99. doi: 10.1080/07399332.2010.490313

33. Lundberg PC, Gerezgiher A. Experiences from pregnancy and childbirth related to female genital mutilation among Eritrean immigrant women in Sweden. *Midwifery* 2008;24(2):214-25. doi: 10.1016/j.midw.2006.10.003

34. Maier C. [Echoes of Silence: Voices of Concern for Genital Mutilation in African Immigrant Women in Vienna: An Ethnological Study] Echo des Schweigens: Stimmen der Betroffenheit zur Genitalverstümmelung bei Afrikanischen Immigrantinnen in Wien: Ethnologische Studie. University of Vienna, 2003.

35. Maternity Action. Women's Voices on Health: Addressing Barriers to Accessing Primary Care. London: Women’s Health and Equality Consortium, 2014.

36. McNeely S, Christie-de Jong F. Somali refugees' perspectives regarding FGM/C in the US. *Int J Migr Health Soc Care* 2016;12(3):157-69. doi: 10.1108/Ijmhsc-09-2015-0033

37. Moxey JM, Jones LL. A qualitative study exploring how Somali women exposed to female genital mutilation experience and perceive antenatal and intrapartum care in England. *BMJ Open* 2016;6(1):e009846. doi: 10.1136/bmjopen-2015-009846

For peer review only

38. Murray L, Windsor C, Parker E, et al. The experiences of African women giving birth in Brisbane, Australia. *Health Care Women Int* 2010;31(5):458-72. doi: 10.1080/07399330903548928

39. Norman K, Gegzabher SB, Otoo-Oyortey N. "Between Two Cultures": A Rapid PEER Study Exploring Migrant Communities' Views on Female Genital Mutilation in Essex and Norfolk, UK. FORWARD & National FGM Centre Report, , 2016.

40. Norman K, Hemmings J, Hussein E, et al. "FGM is Always With Us": Experiences, Perceptions and Beliefs of Women Affected by Female Genital Mutilation in London: Results from a PEER Study. London: Options Consultancy Services and FORWARD, 2009.

41. O'Brien O, Baldeh F, Hassan J, et al. My Voice: Participatory Action Research Project with Men, Women and Young People on Female Genital Mutilation (FGM) in Scotland: (Phase 2). Waverly Care, Edinburgh: Queen Margaret University, 2017.

42. O'Brien O, Baldeh F, Sivapatham S, et al. Participatory Action Research Project with Men, Women and Young People on Female Genital Mutilation (FGM) in Scotland: (Phase 1). Waverly Care, Edinburgh: Queen Margaret University, 2016.

43. Palfreyman A, Brown E, Nam S. Understanding Female Genital Mutilation in Birmingham: Findings from a PEER Study: Options Consultancy Services and Birmingham & Solihull Women's Aid, 2011.

44. Recchia N, McGarry J. "Don't judge me": narratives of living with FGM. *Int J Hum Rights Healthc* 2017;10(1):4-13. doi: 10.1108/Ijhrh-10-2016-0016

45. Safari F. A qualitative study of women's lived experience after deinfibulation in the UK. *Midwifery* 2013;29(2):154-8. doi: 10.1016/j.midw.2011.12.005

46. Salad J, Verdonk P, de Boer F, et al. "A Somali girl is Muslim and does not have premarital sex - is vaccination really necessary?" A qualitative study into the perceptions of Somali women in the Netherlands about the prevention of cervical cancer. *Int J Equity Health* 2015;14(1):1-13. doi: 10.1186/s12939-015-0198-3

47. Shaw E. Female circumcision: perceptions of clients and caregivers. *J Am Coll Health* 1985;33(5):193-7. doi: 10.1080/07448481.1985.9939604

48. Shermarke MAA. Understanding the Canadian Community Context of Female Circumcision [M.S.W.]. McGill University, 1996.

49. Straus L, McEwen A, Hussein FM. Somali women's experience of childbirth in the UK: perspectives from Somali health workers. *Midwifery* 2009;25(2):181-6. doi: 10.1016/j.midw.2007.02.002

50. Thierfelder C. Female Genital Mutilation and the Swiss Health Care System. University of Basel, 2003.

For peer review only

51. Thierfelder C, Tanner M, Bodiang CMK. Female genital mutilation in the context of migration: experience of African women with the Swiss health care system. *Eur J Public Health* 2005;15(1):86-90. doi: 10.1093/eurpub/cki120

52. Upvall MJ, Mohammed K, Dodge PD. Perspectives of Somali Bantu refugee women living with circumcision in the United States: a focus group approach. *Int J Nurs Stud* 2009;46(3):360-8. doi: 10.1016/j.ijnurstu.2008.04.009

53. Vangen S, Johansen REB, Sundby J, et al. Qualitative study of perinatal care experiences among Somali women and local health care professionals in Norway. *Eur J Obstet Gynecol Reprod Biol* 2004;112(1):29-35. doi: 10.1016/S0301-2115(03)00313-0

54. Vaughan C, White N, Keogh L, et al. Listening to North Yarra Communities about Female Genital Cutting. Melbourne, Australia: The University of Melbourne, 2014.

55. Vaughan C, White N, Keogh L, et al. Female Genital Mutilation/Cutting in Regional Victoria. Research to Practice. Melbourne, Australia: The University of Melbourne, 2014.

56. Vloeberghs E, van der Kwaak A, Knipscheer J, et al. Coping and chronic psychosocial consequences of female genital mutilation in the Netherlands. *Ethn Health* 2012;17(6):677-95. doi: 10.1080/13557858.2013.771148

57. Wiklund H, Aden AS, Högberg U, et al. Somalis giving birth in Sweden: a challenge to culture and gender specific values and behaviours. *Midwifery* 2000;16(2):105-15. doi: http://dx.doi.org/10.1054/midw.1999.0197

For peer review only

Supplementary File 6: Full CERQual Assessment Table

Summary of review finding	Studies coded to finding	Methodological limitations	Coherence	Adequacy	Relevance	CERQual assessment	Explanation of CERQual score
**Analytical theme 1: communication is key**							
**1.1 Language barriers and interpretation challenges** For women who did not speak the host country language well, communication about FGM/C was hindered by language barriers and problems with accessing interpretation support that was appropriate for discussing highly sensitive and personal issues.	n=31 1-31	Moderate concerns regarding methodological limitations<br><br>Most studies contributing to this finding are of high quality (16 studies of high quality, 1 study of low quality, 14 studies of medium quality). However, many studies do not report their theoretical paradigm and present only partial or no reflexivity (n=17). There are serious concerns with 3 studies that do not describe ethics processes, 1 study has an unclear fit of methodology to methods and poor analysis. There are moderate concerns in some studies due to unclear analysis (n=3), and participant voices not well presented (n=2).	No concerns regarding coherence	Minor concerns regarding adequacy<br><br>18 of the 31 studies presented data classified as 'thick' or 'rich'. Over half of the papers (31/57) report this theme.	Minor concerns regarding relevance<br><br>The studies for this finding represented 12 different OECD countries. Many studies (n=12) represented the maternity care contexts, but other contexts were also reflected. Almost half the studies (n=15) were exclusively of Somali women, but other nationalities were also represented.	**High confidence**	There were moderate concerns about methodological limitations, but no (or very minor) concerns about the other domains.
**1.2 "Can't talk, not asked": double silence and cultural taboo** As a private, sensitive and taboo issue, communication around FGM/C was hindered by a double silence. Women reported that it	n=37 3,4,8,9,13-23,25-47	Moderate concerns regarding methodological limitations	No concerns regarding coherence<br><br>2 studies present slightly divergent	Minor concerns regarding adequacy<br><br>The majority (n=23) of studies	No concerns regarding relevance<br><br>Studies come from a wide range of different countries,	**High confidence**	There were moderate concerns about methodological limitations (in particular the

For peer review only

Summary of review finding	Studies coded to finding	Methodological limitations	Coherence	Adequacy	Relevance	CERQual assessment	Explanation of CERQual score
was rarely discussed within their own communities and likewise they found it hard to discuss with health providers, especially if these (or the interpreters) were male. For this reason, women preferred the topic to be raised by health providers.		Most studies are of high quality (n=21); 15 are medium and 1 is low quality. Several studies had unclear, partial or no reflexivity. There were serious concerns for 5 studies with unclear ethics processes, and 7 studies had unclear methodology and data analysis processes.	views but with reasons (based on the relationship established with healthcare providers).	related to this review finding provide thick (rich) data. Over half of the included review papers report on this theme (37/57).	with a range of participant groups. They also reflected a range of different healthcare contexts.		predominance of grey literature reports), but there were no (or very minor) concerns about the other domains.
**1.3 Cultural (in)sensitivity** Clumsy and intrusive questioning and comments made women feel stigmatised and embarrassed and led them to avoid further discussion or healthcare interactions. By contrast, women valued culturally sensitive and respectful communication from health providers around FGM/C and this facilitated open discussion.	n=34 3-7,10,13-16,18,19,21,22,24,26-31,33-38,40,42,44,45,48-52	Moderate concerns regarding methodological limitations<br><br>Three studies are of low methodological quality, 17 of medium quality and 14 of high quality. Seventeen of the studies gave only partial or no detail on methodological perspective or reflexivity, and 6 studies had unclear methods and analysis. In 3 studies, there was inadequate detail about participants' voices.	Moderate concerns regarding coherence<br><br>Some findings were ambiguous and over-lapping with other themes.	Minor concerns regarding adequacy<br><br>Twenty one studies provided rich data and 13 were thin (describing single aspects of this theme or lacking detail in women's voices). Over half the studies in the review reported on this theme (34/57).	No concerns regarding relevance<br><br>Studies related to this theme represented diverse healthcare contexts (10 specific to obstetric care, but other contexts represented), diverse countries (n=9) and diverse population groups.	**Moderate confidence**	Two of the four domains have only moderate confidence. Many studies do report on this theme, but there are some concerns on their quality and some of the findings are ambiguous.
**Analytical theme 2: access to care: influenced by an interaction of multi-level community and health service processes**							
**2.1 Influence of cultural norms** Women's care seeking in relation to FGM/C was strongly influenced by wider cultural norms around sexuality and health, including cultural norms on the importance of pre-marital virginity, avoiding	n=35 1,3-8,11-14,16-19,23-32,35,37-41,46,53,54	Moderate concerns regarding methodological limitations<br><br>There are 6 low quality studies, 13 medium and	Moderate concerns regarding coherence<br><br>Cultural norms relate to, and	Moderate concerns regarding adequacy<br><br>35 papers report on this theme.	Minor concerns regarding relevance<br><br>The studies are primarily from the UK (n=10) and USA (n=8)	**Moderate confidence**	Three of the four domains have moderate concerns related primarily to methodological limitations and

For peer review only

Summary of review finding	Studies coded to finding	Methodological limitations	Coherence	Adequacy	Relevance	CERQual assessment	Explanation of CERQual score
male health providers and lack of familiarity with preventive care-seeking. These factors led women to avoid seeking care unless symptomatic or pregnant.		16 high quality studies. The main methodological limitations relate to unclear fit of methodology, limited ethics descriptions and no reflexivity. Other concerns relate to inadequate representation of participant voices.	influence, a wide range of different healthcare phenomena	Seventeen studies provide thin data. There are 12 studies with medium richness explaining 2 or more aspects of this theme and 8 studies provide very rich data accounting for all aspects of the theme.	contexts, but with good representation of other contexts. The majority of the studies (n=19) are exclusively focused on Somali participants, and almost half are focused on maternity care contexts (n=15). However, other healthcare contexts are also included, and several studies with 'thick' data reflect a mix of participant groups.		'thin-ness' of the data, but also to the fact that cultural norms influence healthcare in many different ways, not all of which were explored in great depth.
**2.2 Influence of the family** Women's decision making in healthcare related to FGM/C was often a collective rather than individual phenomenon, requiring consultation with husbands and other family members.	n=19 3,4,8,9,11,12,15,23,24,26-28,31,39-41,46,51,54	Moderate concerns regarding methodological limitations<br><br>Moderate concerns due to methodology and theoretical framework not stated and/or lack of reflexivity (n=13).	Moderate concerns regarding coherence<br><br>Some findings relate to the importance of social support from the family and provide relatively superficial descriptions about the family's role specifically related to decision making.	Minor concerns regarding adequacy<br><br>Rich data from 13 studies. One third of studies in the review (19/57) report on this theme.	Moderate concerns regarding relevance<br><br>Includes studies from different 10 countries. The theme is reported on from studies with various foci, although mainly with Somali participants (n= 12), and relating mainly to obstetric care situations.	**Moderate confidence**	Moderate confidence due to moderate concerns regarding methodological quality, coherence and relevance.

For peer review only

Summary of review finding	Studies coded to finding	Methodological limitations	Coherence	Adequacy	Relevance	CERQual assessment	Explanation of CERQual score
**2.3 Knowledge and information about FGM/C services** Women's knowledge of, and familiarity with, health services was variable. In some cases, women lacked familiarity with the host country health system and this impeded their ability to access care in general. In other cases, women reported being aware of maternity and primary care services. However, there was a particular lack of knowledge and information of FGM/C-related specialist services and where and when it may be appropriate to seek help.	n=34 1,3-5,7,10-12,14,17-29,31,32,36,39,40,44,48,49,54-57	Moderate concerns regarding methodological limitations<br><br>Two studies had low methodological quality, 18 were designated as medium and 14 were high quality. The majority of the studies are journal articles (n=21). There are serious concerns in two studies where ethical processes are poorly described; there is lack of clarity in the fit of methods to methodology in 3 studies and 16 studies lack a discussion of reflexivity.	Moderate concerns regarding coherence<br><br>There was variability in the extent to which studies reported women as being familiar with the general health system in the host country. However, findings relating to women's lack of awareness of specialist FGM/C services were coherent across the studies.	Minor concerns regarding adequacy<br><br>The majority of studies in the review report on this theme (34/57). Over half of the studies provide 'rich' (thick) data related to this finding (n=23); 11 provide thin data.	Minor concerns regarding relevance<br><br>The contributing studies cover 11 countries. Just under half focus exclusively on Somali women (n=16); one third of studies focus on the maternity context (n=13). However, overall, there is a good mix of focus and participant countries.	**Moderate confidence**	Moderate confidence due to moderate concerns regarding methodological quality and coherence.
**2.4 'Hit and miss' care** When accessing healthcare, women reported that the identification of FGM/C and provision of appropriate treatment or referrals could be a "hit and miss" process, depending upon individual provider characteristics and practices rather than being a result of standardised organisational systems and processes. Continuity of care was rare, but when it occurred, women reported that it helped to facilitate communication and access to appropriate care.	n= 38 3-8,10,12-22,24,25,27-31,33,34,36-38,41,43,44,47,48,54,56,57	Moderate concerns regarding methodological limitations<br><br>There is variable quality of studies relating to this finding: high quality (n=20), medium quality (n=15) and low quality (n=3). There are serious concerns with six studies that report unclear ethical procedures, and moderate concerns with 11 studies that report no or unclear methodological	No concerns regarding coherence	Minor concerns regarding adequacy<br><br>38/57 studies report on this theme. Most studies (n=32) contribute relatively rich data to this review finding.	No concerns regarding relevance<br><br>Studies represent 13 countries and a mixture of participant backgrounds and settings.	**High Confidence**	Three out of four domains have no or only minor concerns. Lack of reflexivity as a methodological limitation is less of a concern for this finding as it is not such a highly sensitive topic.

For peer review only

Summary of review finding	Studies coded to finding	Methodological limitations	Coherence	Adequacy	Relevance	CERQual assessment	Explanation of CERQual score
		perspectives. A large number of studies (n=21) had no or partial reflexivity.					
**Analytical theme 3: cultural and bodily dissonance: striving for cultural and bodily integrity**							
**3.1 Moving from normal to different**<br>Women described a shift from feeling 'normal' to 'different' about FGM/C, their bodies and their culture as part of their transition to living in the host country. Some women started to become aware of the suffering and symptoms that their FGM/C may have caused them. Some women started to question and/or resist previously taken for granted aspects of their culture and to feel uncomfortable with their own bodies. However, women also reported feeling shame and anger at being labelled as different and mutilated and felt that their culture was misunderstood.	n=40<br>3,4,7,8,10,12-16,18-31,33,34,36-39,41,43,46-50,53,54,57	Minor concerns regarding methodological limitations<br><br>Thirty five of the studies are rated as high or medium quality. Five are low quality. However, many studies failed to report issues relating to reflexivity.	No concerns regarding coherence	Minor concerns regarding adequacy<br><br>The majority of studies in the review present rich data on this theme. Eight studies provide only thin data where detailed information is not given.	No concerns regarding relevance<br><br>The studies represented mixed contexts, 13 countries and a range of population groups. Eighteen out of 40 studies had an exclusively Somali sample.	**High Confidence**	All domains report no or only minor concerns.
**3.2 Threat to the self: re-living FGM/C pain during clinical interventions**<br>Many studies reported women's experiences of gynaecological clinical interventions and childbirth as events which threatened their sense of bodily and cultural integrity. Women reported great pain, suffering, fear and apprehension around clinical interventions – and related this to their previous healthcare	n=28<br>1,3,7,9-11,13-15,17,19,20,23,25,27-30,33,37,38,41,43,48,51,54,55,57	Moderate concerns regarding methodological limitations<br><br>Serious concerns for 3 studies due to no ethics statements, and poor descriptions of methodology, and unclear fit of methods. There are moderate concerns for 9 studies	Moderate concerns regarding coherence<br><br>There was variability in the extent to which studies reported direct experiences of re-living trauma as opposed to avoidance of	No concerns regarding adequacy.<br><br>The majority of studies present rich data of women's voices, with just four studies presenting thin data on this particular finding. Almost half the	Minor concerns regarding relevance.<br><br>Data is spread across several countries and focussed on different healthcare settings. Eleven studies have an exclusively Somali sample. The focus is specifically on	**Moderate confidence**	Moderate confidence due to moderate concerns regarding methodological quality and coherence.

For peer review only

Summary of review finding	Studies coded to finding	Methodological limitations	Coherence	Adequacy	Relevance	CERQual assessment	Explanation of CERQual score
experiences and, especially, to re-living the original trauma they had experienced during their FGM/C. These emotions were exacerbated in encounters where women felt a loss of control or lack of respect.		due to lack of stated methodology, inadequate participant voices and lack of reflexivity. Sixteen studies are rated high quality.	care or services due to apprehension of pain, trauma and anxiety.	papers in the review report on this finding.	obstetric care in 10 studies. Nine studies are based in the UK.		
**3.3 Being opened: complexities around deinfibulation** For women with type III FGM/C, decision making around 'being opened' (deinfibulation) was influenced by cultural norms and medical imperatives. Some younger women specifically sought out medical deinfibulation before marriage as a way of asserting control over their bodies and lives, but the majority of women considered medical deinfibulation only in the context of pregnancy, and the majority preferred the procedure to be carried out during labour (rather than antenatally) to avoid additional cutting and pain.	n= 28 3,4,8,10,12,14,17,19,20,22,24,25,28,29,33,35-41,46,48,49,53,54,57	Moderate concerns regarding methodological limitations<br><br>This finding is primarily derived from high quality studies (n=15) even though many are either research reports or theses. Half of the studies are journal articles but these include papers of low and medium methodological quality stemming from poor or no reporting of theoretical perspective and methodology, and poor analysis or presentation of participant voices. There are serious concerns with two papers because of no ethics description (n=1) and very weak methodology (n=1).	Moderate concerns regarding coherence<br><br>This was because some studies reported specifically on deinfibulation related to obstetric care, whereas others included this topic in discussions on other issues, hence the findings were less specific, but generally still consistent with the theme.	Minor concerns regarding adequacy<br><br>Twenty eight studies present this theme. Three of these provide only 'thin' data in relation to this finding. Data from the other studies is relatively rich, providing explanatory insights or in-depth descriptions of women's views.	Moderate concerns regarding relevance<br><br>Although several of these studies are, as expected, in maternity settings (n=8), there is a wide diversity of other study foci presenting on this theme and across different nationalities that practise type III FGM/C. However, there is little in-depth exploration of any potential differences between different ethnic groups.	**Moderate confidence**	Moderate confidence due to moderate concerns regarding methodological quality, coherence and relevance.
**3.4 Being changed: complexities around re-infibulation** For women with type III FGM/C, 'being opened' (deinfibulation) was reported as a process involving significant emotional,	n= 19 3,4,8,10,20,22,28,29,35-37,41,46,48,49,52-54,57	Moderate concerns regarding methodological limitations	Minor concerns regarding coherence<br><br>Not all papers reported on the	Minor concerns regarding adequacy<br><br>There are 19 papers reporting	Moderate concerns regarding relevance<br><br>The data is well spread across the included studies,	**Moderate confidence**	Moderate confidence due to moderate concerns regarding methodological

**Summary of review finding**	**Studies coded to finding**	**Methodological limitations**	**Coherence**	**Adequacy**	**Relevance**	**CERQual assessment**	**Explanation of CERQual score**
physical, social and relational adjustments as their bodies became changed. Some women felt ambivalent about these changes and sometimes wanted to be closed again (reinfibulated) to varying degrees, feeling upset if their requests were denied by health providers.		Moderate concerns due to lack of reflexivity in 13 of the papers.	issue of requesting requests for reinfibulation.	on this theme with the majority contributing rich data (n=16). Three studies present thin data.	representing 11 countries, involving diverse participants groups with FGM/C III. However, there is little in-depth exploration of any potential differences between different ethnic groups.		quality and relevance.
**Analytical theme 4: disempowering care encounters**							
**4.1 Being exposed and humiliated** Many studies reported women feeling exposed, objectified and 'on display' during healthcare encounters – due to the health provider's reactions to their FGM/C. They reported feeling a lack of concern for their privacy or dignity and felt humiliated and stigmatised. These experiences led them to lose trust in the health provider and 'the system' more generally.	n=40 1,3,4,7,9,10, 13-31,33-38,42-45,48-51,54	Moderate concerns regarding methodological limitations<br><br>The majority of studies are high quality (n=28). There are moderate concerns in some studies due to unclear presentation of the research paradigm (n=8) and no discussion of reflexivity (n=8). There are serious concerns regarding the data from 6 papers due to lacking ethics descriptions (n=2), unclear methodological fit with methods (n=3) and weak analysis/detail of participant voices (n=1).	No concerns regarding coherence	Minor no concerns regarding adequacy<br><br>Most papers (n=31) provide rich data in relation to this theme. Nine papers provide only thin data.<br><br>Over half the papers in the review report on this theme (40/57).	Minor concerns regarding relevance<br><br>There is good representation of OECD countries, but 14 of the 40 studies are from the UK. A range of population groups are included, but 17 studies focus exclusively on Somali women. A range of healthcare contexts are covered (13 out of 40 papers focus specifically on obstetric care situations).	**High confidence**	A large of number of papers and highly coherent data is presented in this theme with only a small number of poor quality studies. Data covers a mixture of contexts and not only on maternity settings.

Summary of review finding	Studies coded to finding	Methodological limitations	Coherence	Adequacy	Relevance	CERQual assessment	Explanation of CERQual score
**4.2 Being judged and stereotyped** Women reported feeling judged and discriminated against within some of their healthcare encounters. They reported feeling that health providers made negative assumptions about them and provided sub-optimal care based on racial, religious or other stereotypes and misconceptions about their culture in general as well as about FGM/C. Such experiences caused distress, anger and avoidance of the health provider or service.	n= 36 2-10,13,15,16,18,19,22,24-34,36,37,39,44,45,47-50,54	Moderate concerns regarding methodological quality<br><br>There are methodological concerns across studies due to lack of reflexivity, no stated paradigm and serious concerns resulting from no or inadequate ethics statements (n=4), inadequate participant voices (n=1) and a mismatch between methodology and methods (n=1).	Minor concerns regarding coherence<br><br>There was minor variability across studies in terms of the ways in which this phenomenon was experienced.	Minor concerns regarding adequacy<br><br>36 papers report on this theme. The majority of these papers report relatively rich or in-depth findings with only 2 studies providing thin data.	Minor concerns regarding relevance<br><br>Studies span several contexts and 13 countries, involving mainly Somali participants (n=15) but with several studies involving more than 3 participant groups (n=15) and 2 other participant groups (n=4).	**High confidence**	Even though there are some studies with serious methodological concerns (n=6), there are good quality studies across mixed contexts and participant groups, with very minor concerns on coherence, adequacy and relevance.
**4.3 Lacking choice, power and control** Women reported experiences when they felt that they lacked control within the healthcare encounter, especially in the maternity setting and around key interventions such as caesarean sections or episiotomies. They reported feeling excluded from healthcare decision making, not listened to, at the mercy of the system and unable to express their needs. Such experiences could be highly traumatising. Attempts to assert themselves were sometimes construed as being 'difficult'.	n=32 3-5,7,9,10,12,15-20,24,25,27-35,37,38,40,41,47-49,54,56	Moderate concerns regarding methodological quality<br><br>Overall most studies are high quality. Some studies present moderate concerns (n=15) due to lack of reflexivity, and no stated paradigm. Two studies have serious concerns due to lack of ethics descriptions.	No concerns regarding coherence	Minor concerns regarding adequacy<br><br>A large number of studies report this theme. Most of these provide rich data to this theme, with only 8 studies providing 'thin' accounts.	Minor concerns regarding relevance<br><br>The studies span 13 different OECD countries. The majority of studies involve Somali participants (n=20), and the majority refer to obstetric care contexts (n=18) – as stated in the theme.	**High confidence**	Although there are moderate methodological concerns due to mainly studies not stating their methodology or theoretical paradigm, the conduct of majority studies was good and there are only minor concerns regarding coherence, adequacy and relevance.
**4.4 Feeling unsafe and vulnerable** Women expected health providers to be knowledgeable, competent and confident in managing their	n= 46 2-7,9,10,12-29,31-41,43,45,47-49,54-57	Moderate concerns regarding methodological quality	No concerns regarding coherence	No concerns regarding adequacy	No concerns regarding relevance<br><br>Studies involve mixed participant	**High Confidence**	There are a few studies with serious methodological concerns, and

Summary of review finding	Studies coded to finding	Methodological limitations	Coherence	Adequacy	Relevance	CERQual assessment	Explanation of CERQual score
FGM/C. Some women reported times when this did not seem to be the case and where they felt their care had been adversely affected as a result of poor provider skills. In such situations, women felt unsafe and highly vulnerable, describing painful and traumatic clinical experiences. Feelings of vulnerability were heightened for women who lacked social support.		Half of the papers are high quality with only minor concerns (n= 23). Limitations in studies are mainly due to partial or lack of reflexivity which is deemed to be of particular relevance to the reporting of this theme. Five studies have more serious concerns due to lacking ethics statements, poor methodology and partial participant voices.		This theme has a high number of studies contributing to it (n=46). The majority of these provide rich or in-depth data (n=42) in relation to this finding.	groups, and across a range of health contexts, topics and countries.		some concern regarding the lack of reflexivity. However the data is very rich and consistent across contexts, hence there are no concerns regarding adequacy, coherence or relevance.
**Analytical theme 5: positive care encounters**							
**5.1 Trusting and appreciating providers and the system** Women appreciated the good medical services available in the host country. Women also expressed great appreciation for providers who made them feel safe and respected. Such providers were described as knowledgeable and experienced, who treated women with respect, who understood their individual needs and who involved them in their care. Women described these characteristics as facilitating trust and leading to open communication about FGM/C. This, in turn, enhanced the likelihood of women being involved in, and receiving, appropriate care.	n= 34 3-5,7-10,12,14,17-22,26-32,34-39,41,47,48, 54-56	Moderate concerns regarding methodological limitations<br><br>There are 3 studies with serious concerns for not having clear ethics, no paradigm and poor description of methods. There are moderate concerns in 13 papers due to no stated paradigm, lack of reflexivity or inadequate participant voices.	No concerns regarding coherence	No concerns regarding adequacy<br><br>A large number of papers report this theme (n=34). The majority of papers (n=29) provide rich or in-depth data in relation to this finding. Only 5 papers report thin data	No concerns regarding relevance<br><br>Diverse studies across different countries. Fourteen studies are exclusively with Somali women, but other groups (or mixed samples) are also represented. This theme occurs in relation to a range of health care contexts.	**High Confidence**	There are some concerns due to methodological limitations. However the data is rich and consistent across contexts, and there are no concerns regarding adequacy, coherence or relevance.

For peer review only - http://bmjopen.bmj.com/site/about/guidelines.xhtml

Summary of review finding	Studies coded to finding	Methodological limitations	Coherence	Adequacy	Relevance	CERQual assessment	Explanation of CERQual score
**5.2 Voicing healthcare needs and preferences**<br>Women's recommendations for good quality safe FGM/C related care and services they could trust included: (i) interpersonal provider characteristics and behaviours (such as providers being willing and able to talk about FGM/C, providers being skilled and knowledgeable around FGM/C, providers offering culturally sensitive and respectful care) and (ii) service organisation issues (such as having specialist services for FGM/C, being given information and awareness about FGM/C-related services, and involving women and affected communities in FGM service development).	n=45 [1,3-10,12-17,19-22,24-26,28-33,35-39,41-45,47-50,53,54,57]	Moderate concerns regarding methodological limitations.<br><br>The major limitation across studies relates to lack of reflexivity. It is unclear to what extent this may have impacted upon this particular review finding.	Minor concerns regarding coherence<br><br>Studies report on several aspects of what women would like in regard to healthcare services related to FGM/C. Not all studies report on all the different elements of this theme.	Minor concerns regarding adequacy<br><br>45 studies report this theme. The majority have rich or in-depth data (n=31), but 14 have relatively thin data due to limited representation of participant voices or only small aspects of the theme reported on.	No concerns regarding relevance<br><br>Studies report these recommendations across all care settings for women (including studies which did not specify a care setting). This finding included studies from 13 countries and a good mix of participants, with highest being among Somali only (n=19) and a relatively higher number of studies from the UK (n=16).	**High Confidence**	Lack of reflexivity may an issue, however the data is highly consistent across contexts, hence there are no or only minor concerns regarding adequacy, coherence or relevance.

For peer review only

References for CERQual Assessment

1. Abdullahi A, Copping J, Kessel A, et al. Cervical screening: perceptions and barriers to uptake among Somali women in Camden. *Public Health* 2009;123(10):680-5. doi: 10.1016/j.puhe.2009.09.011

2. Ahlberg BM, Krantz I, Lindmark G, et al. 'It's only a tradition': making sense of eradication interventions and the persistence of female 'circumcision' within a Swedish context. *Crit Soc Policy* 2004;24(1):50-78. doi: 10.1107/0261018304039679

3. Berggren V, Bergstrom S, Edberg A-K. Being different and vulnerable: experiences of immigrant African women who have been circumcised and sought maternity care in Sweden. *J Transcult Nurs* 2006;17(1):50-7. doi: 10.1177/1043659605281981

4. Betts V. Transnational Gender, Sexuality and Identity Construction: Ambivalent Subjectivities of Somali Refugee Women in the GTA. MA Thesis, The University of Guelph, 2011.

5. Bulman KH, McCourt C. Somali refugee women's experiences of maternity care in west London: a case study. *Crit Public Health* 2002;12(4):365-80.

6. Carroll J, Epstein R, Fiscella K, et al. Caring for Somali women: implications for clinician-patient communication. *Patient Educ Couns* 2007;66(3):337-45. doi: 10.1016/j.pec.2007.01.008

7. Chalmers B, Omer-Hashi K. What Somali women say about giving birth in Canada. *J Reprod Infant Psychol* 2002;20(4):267-82. doi: 10.1080/0264683021000033183

8. Degni F, Suominen SB, El Ansari W, et al. Reproductive and maternity health care services in Finland: perceptions and experiences of Somali-born immigrant women. *Ethn Health* 2014;19(3):348-66. doi: 10.1080/13557858.2013.797567

9. d'Entremont M, Smythe L, McAra-Couper J. The sounds of silence: a hermeneutic interpretation of childbirth post excision. *Health Care Women Int* 2014;35(3):300-19. doi: 10.1080/07399332.2013.838245

10. Gali MA. Female Circumcision: A Transcultural Study of Attitudes, Identity and Reproductive Health of East African Immigrants [Degree of Doctor of Philosophy in Psychology]. The Wright Institute, University of Berkeley, 1997.

11. Ghebre RG, Sewali B, Osman S, et al. Cervical cancer: barriers to screening in the Somali community in Minnesota. *J Immigr Minor Health* 2015;17(3):722-28. doi: 10.1007/s10903-014-0080-1

12. Hill N, Hunt E, Hyrkas K. Somali immigrant women's health care experiences and beliefs regarding pregnancy and birth in the United States. *J Transcult Nurs* 2012;23(1):72-81. doi: 10.1177/1043659611423828 [published Online First: 2011/11/05]

For peer review only

13. Hussein E. Women's Experiences, Perceptions and Attitudes of Female Genital Mutilation: The Bristol PEER Study. London: FORWARD, 2010.

14. Hussen MA. Services for Women with Female Genital Mutilation in Christchurch: Perspectives of Women and their Health Providers. University of Canterbury, 2014.

15. Fawcett L. Somali Refugee Women and their U.S. Healthcare Providers: Knowledge, Perceptions and Experiences of Childbearing [Doctor of Philosophy]. Arizona State University, 2014.

16. Maternity Action. Women's Voices on Health: Addressing Barriers to Accessing Primary Care. London: Women's Health and Equality Consortium, 2014.

17. Moxey JM, Jones LL. A qualitative study exploring how Somali women exposed to female genital mutilation experience and perceive antenatal and intrapartum care in England. *BMJ Open* 2016;6(1):e009846. doi: 10.1136/bmjopen-2015-009846

18. Murray L, Windsor C, Parker E, et al. The experiences of African women giving birth in Brisbane, Australia. *Health Care Women Int* 2010;31(5):458-72. doi: 10.1080/07399330903548928

19. Norman K, Hemmings J, Hussein E, et al. "FGM is Always With Us": Experiences, Perceptions and Beliefs of Women Affected by Female Genital Mutilation in London: Results from a PEER Study. London: Options Consultancy Services and FORWARD, 2009.

20. O'Brien O, Baldeh F, Hassan J, et al. My Voice: Participatory Action Research Project with Men, Women and Young People on Female Genital Mutilation (FGM) in Scotland: (Phase 2). Waverly Care, Edinburgh: Queen Margaret University, 2017.

21. O'Brien O, Baldeh F, Sivapatham S, et al. Participatory Action Research Project with Men, Women and Young People on Female Genital Mutilation (FGM) in Scotland: (Phase 1). Waverly Care, Edinburgh: Queen Margaret University, 2016.

22. Palfreyman A, Brown E, Nam S. Understanding Female Genital Mutilation in Birmingham: Findings from a PEER Study: Options Consultancy Services and Birmingham & Solihull Women's Aid, 2011.

23. Salad J, Verdonk P, de Boer F, et al. "A Somali girl is Muslim and does not have premarital sex - is vaccination really necessary?" A qualitative study into the perceptions of Somali women in the Netherlands about the prevention of cervical cancer. *Int J Equity Health* 2015;14(1):1-13. doi: 10.1186/s12939-015-0198-3

24. Straus L, McEwen A, Hussein FM. Somali women's experience of childbirth in the UK: perspectives from Somali health workers. *Midwifery* 2009;25(2):181-6. doi: 10.1016/j.midw.2007.02.002

For peer review only

25. Thierfelder C, Tanner M, Bodiang CMK. Female genital mutilation in the context of migration: experience of African women with the Swiss health care system. *Eur J Public Health* 2005;15(1):86-90. doi: 10.1093/eurpub/cki120

26. Upvall MJ, Mohammed K, Dodge PD. Perspectives of Somali Bantu refugee women living with circumcision in the United States: a focus group approach. *Int J Nurs Stud* 2009;46(3):360-8. doi: 10.1016/j.ijnurstu.2008.04.009

27. Vangen S, Johansen REB, Sundby J, et al. Qualitative study of perinatal care experiences among Somali women and local health care professionals in Norway. *Eur J Obstet Gynecol Reprod Biol* 2004;112(1):29-35. doi: 10.1016/S0301-2115(03)00313-0

28. Vaughan C, White N, Keogh L, et al. Listening to North Yarra Communities about Female Genital Cutting. Melbourne, Australia: The University of Melbourne, 2014.

29. Vaughan C, White N, Keogh L, et al. Female Genital Mutilation/Cutting in Regional Victoria. Research to Practice. Melbourne, Australia: The University of Melbourne, 2014.

30. Vloeberghs E, van der Kwaak A, Knipscheer J, et al. Coping and chronic psychosocial consequences of female genital mutilation in the Netherlands. *Ethn Health* 2012;17(6):677-95. doi: 10.1080/13557858.2013.771148

31. Wiklund H, Aden AS, Högberg U, et al. Somalis giving birth in Sweden: a challenge to culture and gender specific values and behaviours. *Midwifery* 2000;16(2):105-15. doi: http://dx.doi.org/10.1054/midw.1999.0197

32. Ameresekere M, Borg R, Frederick J, et al. Somali immigrant women's perceptions of cesarean delivery and patient-provider communication surrounding female circumcision and childbirth in the USA. *Int J Gynecol Obstet* 2011;115(3):227-30. doi: 10.1016/j.ijgo.2011.07.019

33. Baldeh F. Obstetric Care in Scotland: The Experience of Women who have Undergone Female Genital Mutilation (FGM) [MSc Thesis]. Queen Margaret University, 2013.

34. Behrendt A. Listening to African Voices: Female Genital Mutilation/Cutting among Immigrants in Hamburg: Knowledge, Attitudes and Practice. Hamburg, Germany: Plan, 2011.

35. Beine K, Fullerton J, Palinkas L, et al. Conceptions of prenatal care among Somali women in San Diego. *J Nurs Midwifery* 1995;40(4):376-81. doi: Doi 10.1016/0091-2182(95)00024-E

36. Bravo Pastor del Mar M. [Women Subjected to Female Genital Mutilation: Knowledge for the Nursing Discipline] La Voz de las Mujeres Sometidas a Mutilación Genital Femenina: Saberes Para la Disciplina Enfermera [Tesis Doctoral]. University of Murcia, 2014.

For peer review only

37. Asefaw F. [Female Genital Mutilation: A Field Study with Special Consideration of the Background as well as the Health and Psychosexual Consquences for those Affected and their Partners in Eritrea and in Germany] Weibliche Genitalbeschneidung: Eine Feldstudie Unter besonderer Berücksichtigung der Hintergründe Sowie der Gesundheitlichen und Psychosexuellen Folgen für Betroffene und Partner in Eritrea und Deutschland. Humboldt University of Berlin, 2007.

38. Glazer E. Embodiment, Pain and Circumcision in Somali-Canadian Women [M.Sc.]. University of Toronto, 2012.

39. Jones A. Working Psychologically with Female Genital Mutilation: An Exploration of the Views and Experiences of Women who have Experienced FGM and of Clinical Psychologists. University of East London, 2010.

40. Khaja K. Female Circumcision: Life Histories of Somali Women [Doctor of Philosophy]. The University of Utah, 2004.

41. Lundberg PC, Gerezgiher A. Experiences from pregnancy and childbirth related to female genital mutilation among Eritrean immigrant women in Sweden. *Midwifery* 2008;24(2):214-25. doi: 10.1016/j.midw.2006.10.003

42. Ahmed M. Attitudes towards FGM among Somali women living in the UK. In: Momoh C, ed. Female Genital Mutilation. London: Radcliffe 2005:95-117.

43. McNeely S, Christie-de Jong F. Somali refugees' perspectives regarding FGM/C in the US. *Int J Migr Health Soc Care* 2016;12(3):157-69. doi: 10.1108/Ijmhsc-09-2015-0033

44. Norman K, Gegzabher SB, Otoo-Oyortey N. "Between Two Cultures": A Rapid PEER Study Exploring Migrant Communities' Views on Female Genital Mutilation in Essex and Norfolk, UK. FORWARD & National FGM Centre Report, , 2016.

45. Recchia N, McGarry J. "Don't judge me": narratives of living with FGM. *Int J Hum Rights Healthc* 2017;10(1):4-13. doi: 10.1108/Ijhrh-10-2016-0016

46. Safari F. A qualitative study of women's lived experience after deinfibulation in the UK. *Midwifery* 2013;29(2):154-8. doi: 10.1016/j.midw.2011.12.005

47. Shermarke MAA. Understanding the Canadian Community Context of Female Circumcision [M.S.W.]. McGill University, 1996.

48. Glover J, Liebling H, Barrett H, et al. The psychological and social impact of female genital mutilation: a holistic conceptual framework. *J Int Stud* 2017;10(2):219-38.

49. Guerin PB, Allotey P, Elmi FH, et al. Advocacy as a means to an end: assisting refugee women to take control of their reproductive health needs. *Women Health* 2006;43(4):7-25.

For peer review only - http://bmjopen.bmj.com/site/about/guidelines.xhtml

50. Khaja K, Lay K, Boys S. Female circumcision: toward an inclusive practice of care. *Health Care Women Int* 2010;31(8):686-99. doi: 10.1080/07399332.2010.490313

51. Abdi R. Carving culture: Creating identity through female genital cutting. *Durham Anthropology Journal* 2012;18(1):115-53.

52. Thierfelder C. Female Genital Mutilation and the Swiss Health Care System. University of Basel, 2003.

53. Johansen RE. Undoing female genital cutting: perceptions and experiences of infibulation, defibulation and virginity among Somali and Sudanese migrants in Norway. *Cult Health Sex* 2017;19(4):528-42. doi: 10.1080/13691058.2016.1239838

54. Maier C. [Echoes of Silence: Voices of Concern for Genital Mutilation in African Immigrant Women in Vienna: An Ethnological Study] Echo des Schweigens: Stimmen der Betroffenheit zur Genitalverstümmelung bei Afrikanischen Immigrantinnen in Wien: Ethnologische Studie. University of Vienna, 2003.

55. Ballesteros Meseguer C, Almansa Martinez P, Pastor Bravo MdM, et al. [The voice of women subjected to female genital mutilation in the region of Murcia, Spain] La Voz de las mujeres sometidas a mutilacion genital femenina en la region de Murcia. *Gac Sanit* 2014;28(4):287-91. doi: 10.1016/j.gaceta.2014.02.006

56. Essén B, Johnsdotter S, Hovelius B, et al. Qualitative study of pregnancy and childbirth experiences in Somalian women resident in Sweden. *Br J Obstet Gynaecol* 2000;107(12):1507-12.

57. Shaw E. Female circumcision: perceptions of clients and caregivers. *J Am Coll Health* 1985;33(5):193-7. doi: 10.1080/07448481.1985.9939604

For peer review only

PRISMA 2009 Checklist

Section/topic	#	Checklist item	Reported on page #
**TITLE**			
Title	1	Identify the report as a systematic review, meta-analysis, or both.	1
**ABSTRACT**			
Structured summary	2	Provide a structured summary including, as applicable: background; objectives; data sources; study eligibility criteria, participants, and interventions; study appraisal and synthesis methods; results; limitations; conclusions and implications of key findings; systematic review registration number.	2
**INTRODUCTION**			
Rationale	3	Describe the rationale for the review in the context of what is already known.	4-6
Objectives	4	Provide an explicit statement of questions being addressed with reference to participants, interventions, comparisons, outcomes, and study design (PICOS).	5
**METHODS**			
Protocol and registration	5	Indicate if a review protocol exists, if and where it can be accessed (e.g., Web address), and, if available, provide registration information including registration number.	7
Eligibility criteria	6	Specify study characteristics (e.g., PICOS, length of follow-up) and report characteristics (e.g., years considered, language, publication status) used as criteria for eligibility, giving rationale.	8
Information sources	7	Describe all information sources (e.g., databases with dates of coverage, contact with study authors to identify additional studies) in the search and date last searched.	8
Search	8	Present full electronic search strategy for at least one database, including any limits used, such that it could be repeated.	Supplementary file 1
Study selection	9	State the process for selecting studies (i.e., screening, eligibility, included in systematic review, and, if applicable, included in the meta-analysis).	8
Data collection process	10	Describe method of data extraction from reports (e.g., piloted forms, independently, in duplicate) and any processes for obtaining and confirming data from investigators.	10
Data items	11	List and define all variables for which data were sought (e.g., PICOS, funding sources) and any assumptions and simplifications made.	10-11
Risk of bias in individual studies	12	Describe methods used for assessing risk of bias of individual studies (including specification of whether this was done at the study or outcome level), and how this information is to be used in any data synthesis.	9
Summary measures	13	State the principal summary measures (e.g., risk ratio, difference in means).	10-11
Synthesis of results	14	Describe the methods of handling data and combining results of studies, if done, including measures of consistency (e.g., I^2) for each meta-analysis.	10-11

For peer review only - http://bmjopen.bmj.com/site/about/guidelines.xhtml

PRISMA 2009 Checklist

Section/topic	#	Checklist item	Reported on page #
Risk of bias across studies	15	Specify any assessment of risk of bias that may affect the cumulative evidence (e.g., publication bias, selective reporting within studies).	11 & 31
Additional analyses	16	Describe methods of additional analyses (e.g., sensitivity or subgroup analyses, meta-regression), if done, indicating which were pre-specified.	10-11
**RESULTS**			
Study selection	17	Give numbers of studies screened, assessed for eligibility, and included in the review, with reasons for exclusions at each stage, ideally with a flow diagram.	12
Study characteristics	18	For each study, present characteristics for which data were extracted (e.g., study size, PICOS, follow-up period) and provide the citations.	12-16
Risk of bias within studies	19	Present data on risk of bias of each study and, if available, any outcome level assessment (see item 12).	18 & 13-16
Results of individual studies	20	For all outcomes considered (benefits or harms), present, for each study: (a) simple summary data for each intervention group (b) effect estimates and confidence intervals, ideally with a forest plot.	n/a
Synthesis of results	21	Present results of each meta-analysis done, including confidence intervals and measures of consistency.	22-32
Risk of bias across studies	22	Present results of any assessment of risk of bias across studies (see Item 15).	31
Additional analysis	23	Give results of additional analyses, if done (e.g., sensitivity or subgroup analyses, meta-regression [see Item 16]).	n/a
**DISCUSSION**			
Summary of evidence	24	Summarize the main findings including the strength of evidence for each main outcome; consider their relevance to key groups (e.g., healthcare providers, users, and policy makers).	31-34
Limitations	25	Discuss limitations at study and outcome level (e.g., risk of bias), and at review-level (e.g., incomplete retrieval of identified research, reporting bias).	34-35
Conclusions	26	Provide a general interpretation of the results in the context of other evidence, and implications for future research.	31, 35
**FUNDING**			
Funding	27	Describe sources of funding for the systematic review and other support (e.g., supply of data); role of funders for the systematic review.	37

From: Moher D, Liberati A, Tetzlaff J, Altman DG, The PRISMA Group (2009). Preferred Reporting Items for Systematic Reviews and Meta-Analyses: The PRISMA Statement. PLoS Med 6(6): e1000097. doi:10.1371/journal.pmed1000097

For more information, visit: **www.prisma-statement.org**.

For peer review only - http://bmjopen.bmj.com/site/about/guidelines.xhtml

BMJ Open

Seeking Culturally Safe Care: A Qualitative Systematic Review of the Healthcare Experiences of Women and Girls who have Undergone Female Genital Mutilation/Cutting

Journal:	*BMJ Open*
Manuscript ID	bmjopen-2018-027452.R1
Article Type:	Research
Date Submitted by the Author:	15-Mar-2019
Complete List of Authors:	Evans, Catrin; University of Nottingham School of Health Sciences, School of Health Sciences<br>Tweheyo, Ritah; University of Nottingham School of Health Sciences, School of Health Sciences<br>McGarry, Julie; University of Nottingham,<br>Eldridge, Jeanette; University of Nottingham School of Health Sciences, Research and Learning Services,<br>Albert, Juliet; Imperial College Healthcare NHS Trust, Department of Midwifery<br>Nkoyo, Valentine; Mojatu Foundation<br>Higginbottom, Gina; University of Nottingham School of Health Sciences
**Primary Subject Heading**:	Health services research
Secondary Subject Heading:	Evidence based practice, Global health, Public health, Qualitative research
Keywords:	Female genital mutilation, Female genital cutting, CERQual, Deinfibulation, Qualitative systematic review, Thematic synthesis

SCHOLARONE™
Manuscripts

Seeking Culturally Safe Care: A Qualitative Systematic Review of the Healthcare Experiences of Women and Girls who have Undergone Female Genital Mutilation/Cutting

Evans C,[1*] Tweheyo R,[1] McGarry J,[1] Eldridge J,[2] Albert J,[3] Nkoyo V,[4] Higginbottom G,[1]

[1] School of Health Sciences, University of Nottingham, Nottingham, UK.

[2] Libraries Research and Learning Resources, University of Nottingham, Nottingham, UK.

[3] Department of Maternity and Obstetrics, Imperial College Healthcare NHS Trust, London, UK.

[4] Mojatu Foundation, Nottingham, UK.

***Corresponding author contact details**

Dr. Catrin Evans, B Floor, School of Health Sciences, University of Nottingham, Queens Medical Centre, Derby Road, Nottingham, NG7 2HA, UK. Tel. 0115 823 0894, Email: Catrin.Evans@nottingham.ac.uk

Word count: 8,241

For peer review only

Seeking Culturally Safe Care: A Qualitative Systematic Review of the Healthcare Experiences of Women and Girls who have Undergone Female Genital Mutilation/Cutting

ABSTRACT

OBJECTIVE

To explore the experiences of accessing and receiving healthcare related to female genital mutilation/cutting (FGM/C) across the life course, from the perspective of women and girls who have undergone FGM/C.

DESIGN

A systematic review of qualitative research studies, using a thematic synthesis approach.

METHODS

Inclusion criteria were qualitative studies (including grey literature) of any design, from OECD countries, of any date and any language. Sixteen electronic databases were searched from inception to December 2017, supplemented by reference list searching. Papers were screened, selected and quality appraised by two reviewers using established tools from the Joanna Briggs Institute (JBI). NVivo software was utilised to extract study characteristics and code study findings. An inductive thematic synthesis approach was undertaken to identify descriptive themes and interpret these into higher order analytical constructs. Confidence in the review findings was assessed using GRADE-CERQual.

RESULTS

Fifty seven papers (from 55 distinct studies) from 14 different OECD countries were included (50% published within the last eight years). One third of studies focused exclusively on maternity care experiences, whereas others covered a range of foci. No studies reported explicitly on girls' experiences or on experiences of health-service led safeguarding interventions. Only three studies addressed psychological care. The synthesis developed 17 descriptive themes, organised into five analytical constructs. These related to: communication; access to care; experiences of cultural dissonance/integrity; disempowering care experiences and positive care encounters. The themes illuminate significant challenges to obtaining timely and holistic care (especially for deinfibulation), and highlight different ways in which women may experience care as disrespectful, unsafe and disempowering. Key elements of 'culturally safe care' are identified.

CONCLUSIONS

This review has highlighted key knowledge gaps, especially around (i) girls'/unmarried women's experiences, and, (ii) the impact of recent safeguarding interventions. There is an ongoing need for community engagement, service development and staff training.

For peer review only - http://bmjopen.bmj.com/site/about/guidelines.xhtml

PROSPERO REGISTRATION NO: CRD42015030001 2015

STRENGTHS AND LIMITATIONS OF THIS STUDY

- This is the first review that goes beyond a maternity care focus to examine women's/girls' experiences of FGM/C-related healthcare across the life course, enabling new insights into care-seeking and care-access, especially around deinfibulation.
- This is an exceptionally comprehensive review, due to its wide focus and its inclusion of grey literature and papers in any language.
- The review has used a theoretical model of 'cultural safety' to inform its analytical approach and has assessed confidence in its findings using GRADE-CERQual.
- The review process has been informed by strong community involvement and input from a multi-disciplinary expert advisory group at every stage.
- Methodological limitations of the included studies make it difficult to develop findings that reflect nuances of experience related to ethnic group, nationality, age or type of FGM/C.

KEYWORDS

Female genital mutilation, female genital cutting, deinfibulation, qualitative systematic review, thematic synthesis, CERQual

For peer review only

INTRODUCTION

Health services in higher income countries are said to be operating in a context of 'super-diversity'.[1-5] Whereas in previous decades, in-migration was primarily from a limited number of countries traditionally linked through former colonial ties, contemporary migration involves groups from many diverse countries across the globe. Super-diversity poses significant challenges to host country health services, in terms of adapting services and providing culturally appropriate and accessible care to very many different migrant groups with widely different migration histories, languages, health needs and social vulnerabilities.

Female genital mutilation (or female genital cutting) – hereafter referred to as FGM/C - is practised in 30 countries across North and sub-Saharan Africa and in parts of the Middle East and Asia.[6] In an era of super-diverse migration, European and other high-income countries (e.g. USA, Canada, Australia and New Zealand) are becoming home to increasing numbers of women and girls who have experienced, or who may be at risk of, FGM/C (within Europe alone there are thought to be over half a million women and girls who are FGM/C survivors).[7] These women and girls come from highly diverse regions with different cultural traditions, contexts and beliefs associated with the practise of FGM/C, and where different types of FGM/C are practised. For example, in some communities (e.g. within Somalia, Somaliland, Sudan, Eritrea, Kenya) type 3 FGM/C is common involving infibulation (narrowing of the vaginal opening through the creation of a covering seal, formed by cutting and repositioning the labia minora, or labia majora, sometimes through stitching, with or without removal of the clitoris). In other communities however, type 1 (partial or total clitoridectomy) or type 2 (partial or total clitoridectomy and removal of the labia minora, with or without excision of the labia majora) are more common.[8] However, it should be noted that these typologies represent biomedical categories and that different communities have their own nomenclature. Moreover, the exact type of FGM/C a woman has experienced may depend to some extent on the skills and experience of the 'cutter' and women themselves may not always be aware of which type of FGM/C they have experienced.

FGM/C can involve immediate and long term psychological, sexual, relational and physical health sequelae.[9] Health problems may be particular severe for women with Type 3 FGM/C who require a degree of deinfibulation in order to have sexual intercourse and to give birth. WHO defines deinfibulation as "*the practice of cutting open the sealed vaginal opening in a woman who has been infibulated, which is often necessary for improving health and well-*

For peer review only - http://bmjopen.bmj.com/site/about/guidelines.xhtml

being as well as to allow intercourse or to facilitate childbirth".[8] Currently, deinfibulation is recommended for women and girls reporting medical or psychosexual symptoms related to type 3 FGM/C or upon request (i.e. personal choice). In addition, global guidelines specifically recommend that deinfibulation is undertaken to prevent obstetric complications, although the optimal timing for the procedure (antepartum or intrapartum) is unclear.[9-12] [9,13-17] There are compelling clinical reasons however for preferring antepartum deinfibulation, including the fact that it can be performed under local anaesthetic in an out-patient setting, thus reducing costs and risks associated with any emergency procedures that may emerge during labour.[18] In addition, in destination countries where staff may be less familiar with type 3 FGM/C, planned antepartum or pre-marital deinfibulation ensures that it is performed by trained and experienced professionals.[15]

In destination countries, it is essential, therefore, that care pathways are developed that are able to support women's potential psychological and sexual health needs,[9-11,19,20] as well as supporting decision making around deinfibulation. In addition, health professionals are called upon to play a key role in the prevention of FGM/C, through health education with affected communities and identification of girls who may be at risk.[20-22] However, as a cultural phenomenon associated with women's sexuality, gender norms and genital area, FGM/C is a sensitive topic that can be hard for women, communities and health professionals to openly discuss.[23-25] Recent studies of health sector involvement in the management of FGM/C in destination countries, show variable availability of specialist services and staff training.[21,26] Moreover, several systematic reviews indicate that health professionals may lack knowledge, confidence and competence in managing FGM/C.[27-30] In addition, there are on-going calls for greater community involvement in the development of appropriate services related to FGM/C.[31-33]

Aim

This paper reports the findings of a qualitative systematic review that aimed to explore the experiences of accessing and receiving FGM/C-related healthcare across the life course for women and girls who have undergone FGM/C.[34]

For peer review only - http://bmjopen.bmj.com/site/about/guidelines.xhtml

Rationale

Several prior reviews have been undertaken in relation to this topic, however we felt a new review was warranted for several reasons. First, most existing reviews have focused upon women's maternity care experiences or maternity-focused interventions.[35-41] However, as indicated above, a key issue for services is the development of joined up care pathways that are able to offer women a range of holistic services at different time points. Hence, our review adopted a focus on the life course. Second, it is well known that migrants experience a range of barriers to accessing health services,[42-46] yet 'access to care' has not been a focus of prior reviews related to FGM/C. Third, several prior reviews have included studies from all over the world, thereby bringing together widely different cultural and health system contexts.[37-39] We felt that a review focused just on 'destination' countries, would provide findings that were more transferable to these specific contexts.[47,48] Finally, prior reviews have often limited their searches to English language papers or to published literature only.[35,36] By contrast, this review has included any language and a wide range of grey literature sources, and thus is able to offer an extremely comprehensive picture.[49,50]

Theoretical perspective

This review is broadly informed by the theoretical construct of 'cultural safety'.[51] Cultural safety derives from critical social theory and argues that, traditional approaches to 'cultural awareness' or 'cultural competence' within healthcare fail to adequately take into account power relations which are historically unequal between migrant or indigenous groups and healthcare providers and services.[52] Within a cultural competence discourse for example, it is argued that "*the power to define the norm and the onus for action to understand and know about another culture fall to the health professional/service*" p.200.[51] As such, migrant groups remain represented as the 'other' and may be viewed within a deficit model - as a problem that needs to be fixed – rather than as partners whose knowledge and values can contribute equally to a relationship or to service development initiatives. Culturally safe practice, by contrast, is predicated upon relationships of mutual trust and respect. It is a transformative and rights based approach, seeking to uphold the principles of respect, dignity, empowerment, safety and autonomy.[53] Cultural safety envisages the healthcare encounter as a negotiated and equal partnership in which trust plays a central role. The healthcare practitioner's role is to enable patients to say how a service can be adapted and to negotiate an agreed approach – a key aspect of shared decision making.[54] The converse of cultural

For peer review only - http://bmjopen.bmj.com/site/about/guidelines.xhtml

safety is 'cultural risk'[55] in that culturally unsafe practices are defined as: "*any actions that diminish, demean or disempower the cultural identity and well being of an individual or group*" *p7*.[56] Cultural safety is not just a feature of individual practice however. It is argued that culturally safe practice must be institutionalised through organisational policies and structures so that it becomes part of mainstream healthcare provision rather than being dependent upon individual practitioners who may or may not adopt its values and approach.[57-59] This theoretical perspective is well suited to areas of healthcare involving stigmatised or sensitive topics, such as FGM/C, and where there is a strong need for community engagement in service development and shared decision making in care delivery.

To date, reflexive consideration of the use of theory in qualitative systematic reviews has received minimal attention. Indeed, some authors have referred to the conceptual process of meta-synthesis as a 'black box'[60] and recent research has highlighted the poor reporting of the interpretive work of synthesis.[61] Yet, as noted by Guba and Lincoln (1994),[62] there is no such thing as 'theory-free' research. Some authors report using a 'framework synthesis' approach in which the review is designed to test or expand a pre-existing theoretical framework (which is used as the basis of an initial coding framework to develop key themes).[63] In contrast to this relatively deductive approach, in the present review, the concept of Cultural Safety was used in three ways to support an interpretive inductive approach. Firstly, it acted as one of a variety of 'lenses' and perspectives (along with other aspects of our identities and backgrounds) through which we interpreted the findings of the research studies.[64] As such, it represented what Maxwell (2013)[65:39] has termed an 'idea context' or 'spotlight' for the review process in which a theory can help to identify themes that might otherwise be overlooked. Second, as the review progressed, we felt that concepts related to Cultural Safety remained a 'good fit' for helping us to understand and position the review findings within the wider literature.[66] Hence, we drew upon some of its ideas to inform and structure the Discussion and Recommendations. Finally, we were aware that a common criticism of systematic reviews is their failure to be policy relevant or transferable.[67] We felt that framing the Discussion and Recommendations of the review findings in relation to a theoretical framework might help to enhance the potential transferability of the review findings and guide future research.[66]

For peer review only - http://bmjopen.bmj.com/site/about/guidelines.xhtml

METHODS

This qualitative systematic review is reported following the ENTREQ guidelines.[68] The review was registered in Prospero[69] and the methods are documented in a published protocol.[34]

Search strategy

An exhaustive and sensitive three-step search strategy was designed by an experienced information scientist (JE). First, we searched 11 electronic databases using a combination of index terms and text-based queries. These were searched from inception to a cut-off date of 31-12-17. An example search strategy for Ovid MEDLINE is provided in Supplementary File 1. Second, we searched for relevant grey literature drawing upon five key resources, Google, Google Scholar and suggestions from the project's expert advisory group (see Table 1 for all resources/databases that were searched).[70-73] Finally, we hand searched the reference lists of related systematic reviews and of all the included studies. All retrieved datasets were downloaded into group sets within an EndNote library and duplicates were removed.

For peer review only

For peer review only - http://bmjopen.bmj.com/site/about/guidelines.xhtml

Table 1 List of databases and resources searched

Electronic Databases Searched	Date of Search
1. Ovid multi-file search (Medline, Embase, PsycINFO)	10/03/17
2. POPline (via http://www.popline.org/) 1970 to the present	10/03/17
3. ProQuest multifile search	10/04/17
4. Applied Social Sciences Index Abstracts (ASSIA) on ProQuest 1987–current	26/05/17
5. Ovid MEDLINE 1948– and MEDLINE In-Process and Other Non-Indexed Citations to daily update	26/07/17 with monthly alert thereafter (cut-off date for included results 31/12/17)
6. Ovid EMBASE 1980–2017 Week 11	03/08/17 with monthly alert thereafter (cut-off date for included results 31/12/17)
7. CINAHL Plus with Full Text/EBSCO Host to 2017	11/08/17 with monthly alert thereafter (cut-off date for included results 31/12/17)
8. Ovid PsycINFO 1972–March Week 3 2017	14/08/17 with monthly alert thereafter (cut-off date for included results 31/12/17)
9. MIDIRS on Ovid 1971 to April 2017	18/08/17
10. HMIC on Ovid 1979 to date	18/08/17
11. Thomson Reuters Web of Science 1900–2017	18/08/17
**Grey Literature Sources**	
1. British Library Ethos (ethos.bl.uk)<br>2. Networked Digital Library of Theses and Dissertations (NDLTD) (www.ndltd.org)<br>3. NICE (https://www.nice.org.uk)<br>4. Trove – National Library of Australia (trove.nla.gov.au)<br>5. Open Grey (http://www.opengrey.eu/)<br>6. Google<br>7. Google Scholar<br>8. Experts in the field	

Screening and selection

The review included studies from any date and any language that met the following inclusion criteria: (i) empirical research, (ii) qualitative research of any design/methodology (including qualitative findings from mixed methods studies), (iii) undertaken in an OECD country (OECD was utilised as a proxy for potential and comparable high income 'destination' countries), (iv) needed to explicitly report women's or girl's experiences of seeking, accessing or receiving healthcare associated with FGM/C at any point in the life course and in any clinical setting.

Four team members (RT, CE, JMc, GH) independently assessed all titles and abstracts against the inclusion criteria. Full text versions of papers deemed to be potentially relevant were obtained and assessed. Papers found not to meet the criteria were excluded with reasons noted (see Supplementary File 2). Any areas of ambiguity were discussed with the wider project team. Non-English language papers found to be relevant on the basis of their English abstract were sent for complete academic translation.

For peer review only - http://bmjopen.bmj.com/site/about/guidelines.xhtml

Quality assessment

Study quality was assessed by two reviewers using the Joanna Briggs Institute Qualitative Assessment and Review Instrument (JBI-QARI).[74,75] Following the guidance of the Cochrane Qualitative Methods and Implementation Group[76] and the JBI,[49] reports and theses from the grey literature were appraised in the same way as published peer reviewed papers.[70,72,73] Studies were not excluded on the basis of quality, rather, the quality assessment was used to judge the relative contribution of each study to the overall synthesis, and to assess the methodological rigor of each study as part of a process of assessing confidence in the review findings.[77-81]

The JBI-QARI tool was applied to each individual paper and an aggregate score for each was calculated. A criticism of 'scoring' qualitative critical appraisals is that it can be hard to distinguish between the poor conduct of a study and poor reporting, especially where journal word limits constrain the level of detail that can be reported.[82] In addition, there is no consensus regarding the relative importance of any one domain within an assessment tool over another, and, hence, whether they should all be given an equal weight or not. In view of these concerns, we chose to adopt a 'weighting system' used in previous studies by Higginbottom et al[83,84] in which papers were grouped into one of three 'bands' – high, medium or low - to enable a broad brush evaluation to be made of their relative quality – see Table 2.

Table 2 Quality evaluation bands

Quality Evaluation	JBI QARI Tool Aggregate Score	Definition
High	Over 7	A study with a rigorous and robust scientific approach which meets most JBI benchmarks (*perhaps 7 or more 'Yes'*)
Medium	Between 5-7	A study with some flaws but not seriously undermining the quality and scientific value of the research conducted (*perhaps 5-7 'Yes'*)
Low	Under 5	A study with flaws and poor scientific value (*perhaps below 5 of the benchmarks met*)

As an additional strategy for overcoming the potential limitations of solely relying upon a checklist to assess quality, we also assessed the 'richness' of the studies. This is an approach outlined by Popay et al,[85] and subsequently operationalized in Noyes & Popay[86] and Higginbottom et al.[83,84] This approach defines study 'richness' as *"the extent to which the study findings provide explanatory insights that are transferable to other settings"* p.230.[86] 'Thick' papers create or draw upon theory to provide in-depth explanatory insights that can potentially be transferable to other contexts. By contrast, 'thin' papers provide limited or

For peer review only - http://bmjopen.bmj.com/site/about/guidelines.xhtml

superficial description and offer little opportunity for generalizing. Each paper was assessed against the criteria as set out in Higginbottom et al, p.5[83] (see Table 3) and categorized as either 'thick' or 'thin'.

Table 3 Assessment of study richness

Richness	Operational Definition
Thick papers	• Offer greater explanatory insights into the outcome of interest<br>• Provide a clear account of the process by which the findings were produced—including the sample, its selection and its size, with any limitations or bias noted—along with clear methods of analysis<br>• Present a developed and plausible interpretation of the analysis based on the data presented.
Thin papers	• Offer only limited insights<br>• Lack a clear account of the process by which the findings were produced<br>• Present an underdeveloped and weak interpretation of the analysis based on the data presented

Data extraction and assessment of relevance

Study characteristics were extracted by one author (RT) using a modified JBI template and double checked by CE. During this process, papers were categorized in terms of their relevance to the review question. This assessment was made in order to gain a better understanding of the nature of the body of evidence, and also to facilitate the coding process, as described further below. Study relevance was defined as high, medium or low as set out in Table 4. PDFs of all the included papers were imported into NVivo software and the 'findings/results' and 'discussion' sections were coded and analysed.[87]

Table 4 Assessment of study relevance

Study Relevance	Definition
High (specific)	FGM/C-specific healthcare (e.g. the study is focused on a specific aspect of care related directly to FGM/C, e.g. deinfibulation, childbirth for women who have had FGM/C, psychological care)
Medium (direct)	Other healthcare context (e.g. where the study focus is on the maternity care experience of a particular group more generally and where some of the findings relate to the experience of FGM/C)
Low (indirect)	Where the study focus is on general attitudes towards FGM/C and/or experiences and consequences of FGM - & where some FGM/C-related healthcare issues are reported, but are not the main focus of the paper

Data analysis and synthesis

The review adopted a thematic synthesis approach as outlined by Thomas and Harden[88] involving four iterative stages: (i) in-depth reading of the whole papers, (ii) inductive line by

For peer review only - http://bmjopen.bmj.com/site/about/guidelines.xhtml

line coding of the findings, (iii) grouping the codes on the basis of shared characteristics and meanings into descriptive themes, and, (iv), interpreting higher order analytical themes.

Studies categorised as 'thick', high quality and 'highly relevant' were used as index papers to develop an initial set of 'open codes'.[89-101] Two reviewers (RT&CE) undertook this task independently. The codes were then reviewed and compared. Where reviewers had identified and coded the same issue, a code for that issue was agreed for use in coding subsequent papers. A standardised name was identified for the code and a description was produced. Where reviewers had applied slightly different codes to a concept indicating a different interpretation of meaning, they discussed it and either agreed a shared code (based on a shared understanding of meaning) or created two different codes to reflect two different meanings. The initial set of codes were 'free nodes'. These were compared, analysed and discussed by the whole team. Where meanings appeared to relate to a similar concept, these were grouped into broad descriptive themes and sub-themes. This process created a codebook which was applied to the remaining papers and expanded, refined and modified as appropriate (by identifying new codes and new themes or by merging and re-naming existing codes and themes). The codebook ensured that definitions of codes and themes were explicit and could be easily shared across the team and discussed. More detail on this process is given in Supplementary File 3, using the analytical theme of 'Communication is Key' (see below) as an exemplar. Analytical themes were evolved through an in-depth process of comparing and contrasting the meanings of the descriptive themes, analysing these in relation to how they were, or were not, able to illuminate the review questions, and inferring broader phenomena, categories of meaning or social processes that they related to.[102,103]

Patient and public involvement

Public involvement was an integral part of the review process and was achieved in three ways. Firstly, the review was conceived and co-constructed from an ongoing partnership between an academic team (CE, RT, GH, JMc, JE), clinical experts (JA) and a community organisation working on FGM/C-related issues (VN). By undertaking the review we hoped to illuminate women's voices and experiences in order to inform the provision of FGM/C related healthcare. Secondly, an expert advisory group was established from the outset, which included FGM/C survivors, activists and health professionals. This group helped with identifying relevant literature and participated in project meetings in which the synthesis and recommendations were formulated. Finally, the review findings were shared with a wide

For peer review only - http://bmjopen.bmj.com/site/about/guidelines.xhtml

range of FGM/C activist and survivor groups and community organisations and their feedback helped to shape the final report and recommendations.

Rigor within the analytical process

To ensure rigor of the analytical process, the team sought to identify and understand possible 'disconfirming' cases that might challenge emerging interpretations,[104] and to explore possible sub-group or contextual differences. These processes were aided by the creation of a theme matrix (see Supplementary File 4), in which each theme was mapped to its constituent studies. This helped the team to see clearly how common the theme was amongst the studies, what kind of study contexts or samples the theme related to, and to explore why it may have been present in some studies but not in others. In addition, as described above, the whole team and wider project advisory group contributed to the evolution of the synthesis, through reading of key papers and providing feedback on the emerging interpretations.

Assessment of confidence in the review findings (CERQual)

Assessment of confidence in the findings of the review was undertaken using the CERQual (Confidence in Evidence from Reviews of Qualitative Research) approach.[76,77,105-111] Akin to GRADE, CERQual uses assessments of 'concerns' within four distinct domains (methodological limitations, relevance, coherence and adequacy of data) applied to each individual review finding. The CERQual assessment was applied to the descriptive rather than analytical themes, as the latter comprise an aggregation of descriptive themes as well as descriptions of patterns or explanations that are inferred as part of the interpretative process. There is, as yet, relatively little guidance for, or experience with, applying CERQual to higher level analytical themes or theories.[108]

RESULTS

Search outcome

The search outcomes are reported in detail in the PRISMA flowchart in Figure 1. Fifty seven papers met the inclusion criteria, representing 55 distinct studies. Fifty three papers were in the English language; two were published in German,[112,113] and two in Spanish.[114,115]

Figure 1: PRISMA Flow diagram

For peer review only - http://bmjopen.bmj.com/site/about/guidelines.xhtml

Study characteristics

Due to the large numbers of studies in this review, a highly summarised description of key study characteristics and key methodological assessments is found in Table 5. Full details are provided in Supplementary Files 5 and 6.

For peer review only

For peer review only - http://bmjopen.bmj.com/site/about/guidelines.xhtml

Table 5 Summary study characteristics and methodological assessments

No.	Authors/date	Country	Study focus	Eligible participants & country of origin	Quality assessment	Richness	Relevance
1.	Abdi, R. (2012)[116]	UK	FGM/C and identity for Somali women.	16 Somali women	High	Thin	Low
2.	Abdullahi, A., Copping, J., Kessel, A., et al. (2009)[117]	UK	Cervical screening.	50 Somali women	Med	Thin	Med
3.	Ahlberg, B. M., Krantz, I., Lindmark, G., et al. (2004)[23]	Sweden	FGM/C tradition in destination countries.	50 Somali women	High	Thin	Low
4.	Ahmed, M., (2005)[118]	UK	Cross-cultural psychology and women's experience of FGM/C.	56 women (from Somalia & Eritrea)	Low	Thin	Low
5.	Ameresekere, M., Borg, R., Frederick, J., et al. (2011)[119]	USA	Maternity care experiences.	23 Somali women	Med	Thin	Med
6.	Asefaw, F. (2007)[112]	Eritrea and Germany	Post-migration attitudes towards FGM/C.	31 Eritrean women	Med	Thin	Low
7.	Baldeh, F. (2013)[89]	Scotland	Maternity care experiences.	7 women (from Gambia, Ghana & Sudan)	High	Thick	High
8.	Ballesteros Meseguer, C., Almansa Martinez, P., Pastor Bravo, M. d. M., et al. (2014)[114]	Spain	Health impacts of FGM/C.	9 Senegalese women	Med	Thin	Med
9.	Behrendt, A. (2011)[120]	Germany	Views on FGM/C.	91 women (from Ghana, Nigeria & Togo)	Med	Thin	Low
10.	Beine, K., Fullerton, J., Palinkas, L., et al. (1995)[121]	USA	Maternity care experiences.	14 Somali women	Med	Thin	Med
11.	Berggren, V., Bergstrom, S., & Edberg, A.-K. (2006)[122]	Sweden	Maternity care experiences.	21 women (from Eritrea, Somalia & Sudan)	Med	Thick	High
12.	Betts, V. (2011)[123]	Canada	Identity, gender, sexuality and health care experiences.	13 Somali women	Med	Thick	High
13.	Bravo Pastor del Mar, M. (2014)[115]	Spain	Views on FGM/C.	24 women (From Senegal, Nigeria & Gambia)	High	Thin	Low
14.	Bulman, K. H., & McCourt, C. (2002)[124]	UK	Maternity care experiences.	12 Somali women	Med	Thin	Med
15.	Carroll, J., Epstein, R., Fiscella, K., et al. (2007)[125]	USA	Healthcare experiences.	34 Somali women	High	Thick	Med
16.	Chalmers, B., & Omer-Hashi, K. (2002)[126]	Canada	Maternity care experiences.	415 Somali women	Med	Thick	Med

For peer review only - http://bmjopen.bmj.com/site/about/guidelines.xhtml

No.	Authors/date	Country	Study focus	Eligible participants & country of origin	Quality assessment	Richness	Relevance
**17.**	Degni, F., Suominen, S. B., El Ansari, W., et al. (2014)[127]	Finland	Reproductive health and maternity care experiences.	70 Somali women	High	Thin	Med
**18.**	d'Entremont, M., Smythe, L., & McAra-Couper, J. (2014)[90]	France	Childbirth experiences.	4 women (from different countries)	High	Thick	High
**19.**	Essén, B., Johnsdotter, S., Hovelius, B., et al. (2000)[128]	Sweden	Maternity care experiences.	15 Somali women	Med	Thin	Low
**20.**	Fawcett, L., (2014)[91]	USA	Views on FGM/C and maternity care.	30 Somali women	High	Thick	High
**21.**	Gali, M. A. (1997)[129]	USA	Reproductive healthcare experiences.	26 women (from Sudan, Eritrea, Ethiopia & Egypt)	Med	Thin	Low
**22.**	Ghebre, R. G., Sewali, B., Osman, S., et al. (2015)[130]	USA	Views on cervical screening.	23 Somali women	High	Thick	Med
**23.**	Glazer, E., (2012)[131]	Canada	Pain and embodiment associated with FGM/C.	14 Somali women	High	Thin	Low
**24.**	Glover, J., Liebling, H., Barrett, H., et al. (2017)[92]	UK	Psychological impact, resilience and experiences of FGM/C.	20 women (from Somalia, Kenya, South Africa & Gambia)	High	Thick	High
**25.**	Guerin, P. B., Allotey, P., Elmi, F. H., et al. (2006)[132]	Australia and New Zealand	Reproductive healthcare experiences.	10 women (from Somalia, Ethiopia, Eritrea, Sudan & Nigeria)	Low	Thin	Low
**26.**	Hill, N., Hunt, E., & Hyrkas, K. (2012)[133]	USA	Maternity care experiences.	18 Somali women	Med	Thick	Low
**27.**	Hussein, E., (2010)[134]	UK	Views on FGM/C & related services.	8 women (from Somalia & Sudan)	Med	Thin	Low
**28.**	Hussen, M. A. (2014)[135]	New Zealand	Views on FGM/C and healthcare experiences.	20 women (from Eritrea, Ethiopia, Somalia & Sudan)	Med	Thick	Med
**29.**	Johansen, R.E. (2017)[136]	Norway	Experiences and perceptions of premarital deinfibulation.	23 women (from Somalia & Sudan)	High	Thick	Med
**30.**	Jones, A. (2010)[93]	UK	Views and experiences of FGM/C.	6 women (from Sudan & Somalia)	High	Thick	High
**31.**	Khaja, K. (2004)[137]	Canada and USA	Views and experiences of FGM/C.	17 Somali women	High	Thin	Low
**32.**	Khaja, K., Lay, K., & Boys, S. (2010)[138]	Canada and USA	Views and experiences of FGM/C.	17 Somali women	Low	Thin	Low
**33.**	Lundberg, P. C., & Gerezgiher, A. (2008)[94]	Sweden	Maternity care experiences.	15 Eritrean women	High	Thick	High

For peer review only - http://bmjopen.bmj.com/site/about/guidelines.xhtml

No.	Authors/date	Country	Study focus	Eligible participants & country of origin	Quality assessment	Richness	Relevance
**34.**	Maier, C. (2003)[113]	Austria	Views, experiences and impacts of FGM/C.	3 women (from different countries)	Low	Thin	Low
**35.**	Maternity Action. (2014)[139]	UK	Maternity care experiences.	23 women (from several countries)	Low	Thin	Med
**36.**	McNeely, S., & Christie-de Jong, F. (2016)[140]	USA	Views and experiences of FGM/C.	13 Somali women	High	Thin	Low
**37.**	Moxey, J. M., & Jones, L. L. (2016)[95]	UK	Maternity care experiences.	10 Somali women	High	Thick	High
**38.**	Murray, L., Windsor, C., Parker, E., et al. (2010)[141]	Australia	Birth experiences.	10 women (from Sudan Liberia, Ethiopia & Somalia)	High	Thick	Med
**39.**	Norman, K., Gegzabher, S. B., & Otoo-Oyortey, N. (2016)[142]	UK	Views and experiences of FGM/C and community engagement.	33 women (from various countries)	Med	Thin	Low
**40.**	Norman, K., Hemmings, J., Hussein, E., et al. (2009)[143]	UK	Views and experiences of FGM/C, community engagement and services.	40 women (from Sudan, Eritrea & Somalia)	Med	Thin	Low
**41.**	O'Brien, O., Baldeh, F., Hassan, J., et al. (2017)[144]	Scotland	Views and experiences of FGM/C, community engagement and services.	48 women (from Sudan Nigeria & Zimbabwe)	High	Thin	Low
**42.**	O'Brien, O., Baldeh, F., Sivapatham, S., et al. (2016)[145]	Scotland	Views and experiences of FGM/C, community engagement and services.	39 women (from 17 African countries)	High	Thin	Low
**43.**	Palfreyman, A., Brown, E., & Nam, S. (2011)[96]	UK	Views and experiences of FGM/C, community engagement and services.	105 women (from Somali, Eritrea, Sudan, Sierra Leone & Gambia)	High	Thick	High
**44.**	Recchia, N., & McGarry, J. (2017)[25]	UK	Views on FGM/C and healthcare experiences.	6 women (from Kenya, Nigeria & Ethiopia)	Med	Thick	Low
**45.**	Safari, F., (2013)[146]	UK	Women's experience of deinfibulation.	9 women (from Somalia & Eritrea)	High	Thin	Low
**46.**	Salad, J., Verdonk, P., de Boer, F., et al. (2015)[147]	Netherlands	Cervical screening.	46 Somali women	High	Thick	Med
**47.**	Shaw, E. (1985)[148]	USA	Views on FGM/C and healthcare experiences.	12 women (from Sudan, Egypt & Somalia)	Low	Thin	Low
**48.**	Shermarke, M.A.A. (1996)[149]	Canada	Views on FGM/C.	8 Somali women	High	Thin	Med
**49.**	Straus, L., McEwen, A., & Hussein, F. M. (2009)[97]	UK	Maternity care experiences.	8 Somali women	High	Thick	High

For peer review only - http://bmjopen.bmj.com/site/about/guidelines.xhtml

No.	Authors/date	Country	Study focus	Eligible participants & country of origin	Quality assessment	Richness	Relevance
**50.**	Thierfelder, C. (2003)[98]	Switzerland	Gynaecological and maternity care experiences.	29 women (from Somalia & Eritrea)	High	Thick	High
**51.**	Thierfelder, C., Tanner, M., & Bodiang, C. M. K. (2005)[150]	Switzerland	Gynaecological and maternity care experiences.	29 women (from Somalia & Eritrea)	Med	Thin	Med
**52.**	Upvall, M. J., Mohammed, K., & Dodge, P. D. (2009)[151]	USA	Health experiences.	23 Somali women	Med	Thick	High
**53.**	Vangen, S., Johansen, R. E. B., Sundby, J., et al. (2004)[99]	Norway	Maternity care experiences.	23 Somali women	High	Thick	High
**54.**	Vaughan, C., White, N., Keogh, L., et al. (2014)[100]	Australia	Views and experiences of FGM/C, community engagement and services.	87 women (from Eritrea, Ethiopia, Somali & Sudan)	High	Thick	High
**55.**	Vaughan, C., White, N., Keogh, L., et al. (2014)[101]	Australia	Views and experiences of FGM/C, community engagement and services.	50 women (from Togo, Sudan & Kenya)	High	Thick	High
**56.**	Vloeberghs, E., van der Kwaak, A., Knipscheer, J., et al. (2012)[152]	Netherlands	Psycho-social and health experiences.	66 women (from Somalia, Sudan, Eritrea, Ethiopia & Sierra Leone)	High	Thin	Med
**57.**	Wiklund, H., Aden, A. S., Högberg, U., et al. (2000)[153]	Sweden	Maternity care experiences.	9 Somali women	Med	Thin	Med

For peer review only - http://bmjopen.bmj.com/site/about/guidelines.xhtml

The studies represented 14 different countries, including: Australia,[100,101,132,141] Austria,[113] Canada,[123,126,131,137,138,149] Finland,[127] France,[90] Germany,[112,120] Netherlands,[147,152] New Zealand,[132,135] Norway,[99,136] Spain,[114,115] Sweden,[23,94,122,128,153] Switzerland,[98,150] Scotland,[89,144,145] England[25,92,93,95-97,116-118,124,134,139,142,143,146] and USA.[91,119,121,125,129,130,133,140,148,151] The studies were a mixture of older and more recent research, with publication dates ranging from 1985 to 2017. However, 29 papers had been published since 2011, hence half of the papers reflected a more contemporary context. Over half of the included papers (n=33) were peer reviewed journal articles.[23,25,90,92,94,95,97,99,114,116,117,119,121,122,124-128,130,132,133,136,138,140,141,146-148,150-153] Other papers included 13 theses,[89,91,93,98,112,113,115,123,129,131,135,137,149] 10 unpublished research reports[96,100,101,120,134,139,142-145] and one book chapter.[118]

A large proportion of studies (n=18) focused specifically on women's birth/maternity care experiences.[89-91,94,95,97,99,119,121,122,124,126,128,133,135,141,153] Other studies focused on: general views on healthcare,[25,100,101,112,113,115,118,134,138,148,151] general attitudes towards FGM/C,[23,96,116,120,137,140,142-145,149] experiences of sexual/reproductive health services,[98,114,127,129,132,150] cervical screening,[117,125,130,147] psychological issues,[92,93,152] deinfibulation, [136,146] GP services,[139] identity,[123] pain/embodiment.[131] There were no studies that examined women's experiences of surgical reconstruction following FGM/C and no studies that examined experiences of health-service led safeguarding interventions.

The studies were primarily of adult women. Only one study included girls under the age of 18 years but did not report health-related experiences of this group.[23] There were no studies that focused specifically on healthcare issues related to FGM/C in older (post-child bearing) women. Hence, although the aim of the review was to explore FGM/C across the life course, the studies all focused on generic issues relating to adult women and no further age-specific differentiation could be made. The majority of studies were with women from FGM/C practising countries in sub-Saharan Africa, and the majority of these included women specifically from countries in the Horn of Africa where type 3 FGM/C is most commonly practised. It is likely therefore, that the findings included in the review reflect issues that may be specific to these population groups and to the experience of type 3 FGM/C. Only three studies explicitly reported including women from Egypt/Middle East but did not differentiate these women's experience from the rest of the sample.[118,129,148]

For peer review only - http://bmjopen.bmj.com/site/about/guidelines.xhtml

Methodological quality

Full details of assessments of methodological quality, richness (thick/thin) and relevance are provided in Supplementary File 6 (see Table 5 for a summary). Thirty papers were assessed as being of high quality,[23,89-101,115,116,125,127,130,131,136,137,140,141,144-147,149,152] 21 as medium quality,[25,112,114,117,119-124,126,128,129,133-135,142,143,150,151,153] and six as low quality.[113,118,132,138,139,148]

A methodological weakness across many studies was lack of discussion of the underpinning philosophical standpoint (question one of the QARI tool), making it difficult to assess the congruency of the chosen methodology. Likewise, many studies did not describe any clear methodology (simply stating that they adopted a generic ‘qualitative approach’). Such studies tended to have ‘thin’ or mainly descriptive findings, and it was difficult to judge the congruence of the methodology with the research question and the methods (questions two and three on the QARI tool). Finally, a limitation across many studies was lack of discussion of reflexivity (questions six and seven on the QARI tool). Given the sensitive nature of FGM/C (and sexuality or migrant healthcare) as a topic, the failure to explore the researcher’s own theoretical standpoint or their role, professional background, ethnicity, experience of FGM/C or relationship to the participants is a significant weakness, making it hard to judge the dependability of the findings.[154]

Thematic synthesis findings

The findings from 57 papers were synthesised into five analytical themes which represent a synthesis and interpretative analysis of 17 descriptive themes. Table 6 shows their inter-relationship and provides one or two quotes to illustrate each descriptive theme. Hence, the description of themes below does not include quotes. Rather, each analytical theme is explained, followed by an elaboration of its constituent descriptive themes. Due to the large number of studies that contributed findings to each descriptive theme, rather than ‘crowd’ the text with multiple repetitive references to these individual studies, the reader is referred to a theme matrix (Supplementary File 4) that shows which studies have contributed findings to each theme.

For peer review only - http://bmjopen.bmj.com/site/about/guidelines.xhtml

Table 6 Themes, quotations and CERQual assessment

Theme No.	Theme Heading	No. of studies	CERQual Assess-ment	Indicative Quotes
**Analytical Theme 1: Communication is key**				
1.1	Language barriers and interpretation challenges	n=31 23,90,91,95-97,99-101,117,122-127,129,130,133-135,139,141,143-145,147,150-153	High confidence	*"I wish I had a way to communicate with the doctor directly. There are things that sometimes I want to say or ask but I feel embarrassed saying it through a translator, especially on the phone…..They never translate what I'm saying. They tell me that is a shameful question..." Sweden, p.364*[151]
1.2	"Can't talk, not asked": double silence and cultural taboo	n=37 25,89-91,93-96,99-101,112,115,118-123,127,131,134,135,137,139-147,149-153	High confidence	*"If they had asked me I would have told them, but I was never asked……..I was worried and scared during my first pregnancy because I have heard people talk about the pain but I didn't know how to tell them……. I was shy." Scotland, p.23-24*[89]
1.3	Cultural (in)sensitivity	n=34 25,89,91,92,96-101,112,115,116,118,120-126,129,131,132,134,135,137-139,141-143,145,151-153	Moderate confidence	*"The doctor was not comfortable with me. He asked if I enjoy violent sex with my husband. I don't feel comfortable to talk about sex even with females— yet here is a male doctor examining my genitals and making mean comments." USA, p.274*[126]
**Analytical Theme 2: Access to care: influenced by an interaction of multi-level community and health service processes**				
2.1	Influence of cultural norms	n=35 93-95,97,99-101,112,113,117,119,121-127,130,131,133-137,139,141,143,146,147,150-153	Moderate confidence	*"Even though we had FGM type 2 or 3, had a small vaginal opening and were virgins, we did not ask for de-infibulations before marriage because that is shameful in our culture. Most of us who have FGM had problems with penetration following marriage." New Zealand, p.43*[135]<br><br>*"In my country normally…………people don't go………usually people go to a psychologist if it is something like erm….maybe chronic issues that they cannot solve at all….or sometimes if it is abnormal…. They think if you go to a psychologist you are mad or crazy." England, p.6*[93]
2.2	Influence of the family	n=19 90,91,93,94,97,99,100,113,116,122,123,127,130,133,137,146,147,151,153	Moderate confidence	*"I know my friend she wants to do that (deinfibulation) but her husband doesn't let her….. I told her, this is good for you - go and do. She said ok but her husband said no……Maybe if she did this (deinfibulation), it would have been a big problem in the family." England, p.157*[146]
2.3	Knowledge and information about FGM/C services	n=34 92,93,95-97,99-101,113-115,117,119,122-	Moderate confidence	*"They need to have someone that talks to you before and while you have a baby. I would have wanted someone to help me and tell me what was going to happen but I never got that… You know someone who was specially trained so you only see people who understand FGM." England, p.233*[92]

For peer review only - http://bmjopen.bmj.com/site/about/guidelines.xhtml

Theme No.	Theme Heading	No. of studies	CERQual Assess-ment	Indicative Quotes
		124,126,128-130,132,133,135,137,141-145,147,148,150,151,153		
2.4	'Hit and miss' care	n= 38<br>89,91,92,94-97,99-101,112,113,115,120,122-129,131,133-135,139-145,148-150,152,153	High confidence	*"Sometimes people go to one doctor after another describing their problems to them. They do not understand so they cannot find a solution. There was a woman who was suffering from bleeding and pain. When she went to the GP, he told her it was normal. He did not know how to treat her. She kept going several times and at last they opened her." England, p.41*[143]
**Analytical Theme 3: Cultural and bodily dissonance: striving for cultural and bodily integrity**				
3.1	Moving from normal to different	n=40<br>89,91-94,96,97,99-101,112,113,115,120,122,123,126,127,129,131-136,138-141,143-153	High confidence	*"Okay, now I'm missing something that other women has…..Well the first shock was to find out that people here are not circumcised…….You have to deal with that… I'm not the same as women here……….and then I started to kind of - you grieve a bit, that 'what is this thing that has been taken away from me?'……………..For me it has caused me lots of problems because when I went to health services, the minute that I say 'I was circumcised' it just create huge barrier between them and me, like you know - suddenly I'm from another planet" Australia, p.23*[100]
3.2	Threat to the self: re-living FGM/C pain during clinical interventions	n=28<br>89-92,94,95,99-101,112-114,116,117,122,126,129-131,134,135,140,143,144,147,148,150,152	Moderate confidence	*"You always remember it. They decide to cut me, and when she went to do it she didn't explain to me and I panic. I nearly kick her. Two of the nurses held me down. That was it…I was a little girl again being held down." England, p.228*[92]
3.3	Being opened: complexities around deinfibulation	n= 28<br>89,92-97,100,101,112,113,115,121-123,127,129,131-133,135-137,143,144,146,148,150	Moderate confidence	*She [Somali lady] hadn't talked to friends about it, just the midwife….. She said: "When I met the midwife she told me a lot about FGM. The midwife said to me 'If you want I can open it for you. If you don't want that I won't'. We talked about it a lot. After four months I tell her 'I want you to open it for me. Do me a small operation." Scotland, p.31*[144]
3.4	Being changed: complexities around re-infibulation	n= 19<br>92,94,96,98,100,101,112,113,115,121-123,127,129,132,136,144,146,148	Moderate confidence	*"I became a victim in Sudan already when I was 4 years old; I had no choice. Now I have to become a victim again after delivery, when the midwives refuse to resuture me. I just ask for a few stitches, not to have an open wound." Sweden, p.53*[122]
**Analytical Theme 4: Disempowering care encounters**				
4.1	Being exposed and humiliated	n=40<br>25,89-92,95-97,99-101,112,113,115-118,120-123,126,129,131,132,134,135,138-145,147,150-153	High confidence	*"Me - I was installed with my legs spread apart - they all came to see….and I didn't see any reason why they came to see me except for the fact that I was excised. All those people who came, who entered into my intimacy without my authorization, who didn't ask me if it bothered me.. … I felt that it was an aggression". France, p.308*[90]<br><br>*"They showed no respect to me, I understand now why people don't want to talk about it to the midwife. I*

For peer review only - http://bmjopen.bmj.com/site/about/guidelines.xhtml

Theme No.	Theme Heading	No. of studies	CERQual Assess-ment	Indicative Quotes
				will tell anyone not to tell them about being circumcised." Scotland, p.23[89]
4.2	Being judged and stereotyped	n= 36 23,25,89-93,96,97,99-101,112,113,115,119,120,122-127,129,132,134,138,139,141-143,149-153	High confidence	*One participant….described that a German gynaecologist (a woman) looked at her with so much pity and irritation after the physical examination that she felt for the first time in her life that she was not normal….she felt that she was treated like a victim who could not be talked to rationally. The participant decided…..to abstain from consulting German doctors. Germany, p.83*[120]
4.3	Lacking choice, power and control	n=32 89-92,94,95,97,99-101,112,113,119-124,126,128,129,131-133,137,139,141,143,144,149,150,152,153	High confidence	*"I can't say that the birth went well. The midwife said that I couldn't give birth without being cut ... otherwise the baby won't come without a caesarean section or I can lose the baby. Nobody asked me what I thought. I was there in pain ... without pain relief, nothing." France, p.308*[90]
4.4	Feeling unsafe and vulnerable	n= 46 23,25,89-97,99-101,112-115,119-126,128,129,131-135,137,139-141,143-145,147-151,153	High confidence	*"They didn't know what they were doing, and uh, they, you know they had to give me more cuts then in an awkward way… I was telling the doctor to cut the FGM and totally he ignored me. And he was cutting in the sides instead of cutting the front. It was very degrading and I am very distressed to think about it." Australia, p.25*[100]
**Analytical Theme 5: Positive care encounters**				
5.1	Trusting and appreciating providers and the system	n= 34 90,92-96,99-101,112-115,119-124,126-129,131,133,135,141,143-145,149,151-153	High confidence	*"I was lucky when I met a midwife in Sweden who knew about circumcised women. This was a great help to make me feel secure because it was my first time to be pregnant and to live far from my parents and family." Sweden, p.219*[94]<br><br>*"I know a friend that had to be opened before her delivery. She was lucky because she had found a midwife who knew about FGM - and she booked her to have the operation before the delivery." England, p.15*[134]
5.2	Voicing healthcare needs and preferences	n=45 25,89-97,100,101,112,113,115,117-119,121-127,129,131-136,138-140,142-145,148-153	High confidence	*"A while ago, all the women used to choose caesarean to give birth but now the health services know about women who are circumcised. They know of their situations and the complications they can have during maternity like bleeding and that they need help with the opening before the baby comes. When the health services don't know about your condition, they refer people to other services. There are some who are understanding, especially if different communities come to attend the clinics or hospitals, as they get more oriented on the different cultures of the communities". England, p.43*[143]

For peer review only - http://bmjopen.bmj.com/site/about/guidelines.xhtml

Analytical theme 1 - Communication is key

The synthesis revealed that communication was a key interpersonal process that underpinned women's ability to seek care, to obtain care and to have a positive care experience. Conversely, difficulties in communication negatively impacted all of these dimensions. Communication about FGM/C was characterised by issues that might be expected within migrant women's encounters with new host country healthcare systems - such as language barriers within consultations. However, communication about FGM/C presented an additional layer of complexity related to the fact that FGM/C was seen by both women and healthcare providers as a deeply personal, private and sensitive issue touching upon a range of taboos associated with gender, culture and sexuality – all issues that in many societies are shrouded in secrecy and silence. When women encountered providers who were able to overcome taboos and cultural differences, it forged bonds of trust and facilitated an environment in which women were able to talk about their FGM/C and to explore options for care. However, the majority of studies reported challenges and problems with communication and interpretation which led to women being unable to talk about their problems or to explore appropriate treatment options, led them to avoid healthcare in general and generated a sense of mistrust in the system. This theme mainly related to maternity care interactions, but also to communication about cervical cancer screening.[100,101,139,144,147]

Theme 1.1 Language barriers and interpretation challenges

The majority of studies (n=31) reported that for women who did not speak the host country language well, communication about FGM/C was hindered by language barriers and problems with accessing interpretation support that was appropriate for discussing highly sensitive and personal issues. Language barriers meant that women were unable to form a trusting relationship with their provider, express their needs adequately or understand information or advice. This led to frustration and increased anxiety, especially in a context where consultation times were often limited.[124,135] In some cases, women felt that they had had poor clinical experiences as a direct result of their inability to communicate about FGM/C.[23,135,144] In other examples, women avoided mentioning their FGM/C at all because they knew they lacked the language to explain.[134,135,150]

For peer review only - http://bmjopen.bmj.com/site/about/guidelines.xhtml

Theme 1.2 "Can't talk, not asked": double silence and cultural taboo

As a private, sensitive and taboo issue, communication around FGM/C was hindered by a double silence. Women reported that FGM/C was rarely discussed within their own communities and likewise they found it hard to discuss it with health providers, especially if these (or the interpreters) were male. For this reason, women preferred the topic to be raised by health providers, however a commonly reported finding was that, even when women appropriately accessed relevant services (for example, GPs or antenatal services,[95,142,143]) and might appreciate the general care offered,[115,127] healthcare providers often did not ask about FGM/C, even when the consultations were for pregnancy-related check-ups. The consequence was that FGM/C was sometimes not identified or discussed until women presented in the labour room, so opportunities for care planning or birth planning were missed.[89] In addition, some studies reported that women found communication to be easier when supported by a knowledgeable community advocate or confident peer.[89,96,123,125,139] Other studies emphasised that open communication was predicated upon being able to develop a trusting relationship with the health professional which in turn required time and continuity of care.[95,100,101,124]

Theme 1.3 Cultural (in)sensitivity

This theme was reported by 34 studies in which women described experiencing comments and questions from health providers that were perceived as clumsy, insensitive or intrusive. As a result, women reported feeling ashamed, scared or stigmatised. Such encounters inhibited women from talking about their FGM/C and in some cases, led to them avoiding examinations or healthcare services.[112,129] By contrast, culturally sensitive communication was greatly appreciated and enabled women to openly discuss their FGM/C and related questions.[90,95,145]

Analytical theme 2 - Access to care: influenced by an interaction of multi-level community and health service processes

The review found that access to care related to FGM/C was influenced by an interaction of factors operating at individual and community levels as well as at health system and service levels. At individual and community levels, studies showed that care seeking was influenced by cultural norms around health and sexuality, collective approaches to decision making and

For peer review only - http://bmjopen.bmj.com/site/about/guidelines.xhtml

the level of knowledge and information that individuals and communities had about FGM/C and health service availability. These factors influenced whether or not a woman would seek care, at what time period and where. However, the studies also showed that care-seeking choices were shaped by the types of services available and that outcomes were influenced by the extent to which the health system, health services and health practitioners were ready and able to provide the range of support that women may need at different points in their lives. The review showed that there were challenges within communities in terms of seeking care and finding the right services. However, even when services were appropriately accessed, care was sometimes haphazard and sub-optimal due to variable levels of staff expertise and inconsistent and unclear referral pathways, policies and procedures.

Theme 2.1 Influence of cultural norms

Women's care seeking in relation to FGM/C was strongly influenced by wider cultural norms around sexuality and health, including cultural norms on the importance of pre-marital virginity, avoiding male health providers and lack of familiarity with preventive care-seeking. These factors often led women to avoid seeking care unless symptomatic or pregnant. Thirty five studies contributed to this theme.

Women reported that strong cultural imperatives valuing premarital chastity and virginity, meant that they would avoid procedures requiring gynaecological examinations (e.g. pap smears[101,130,143,147]), and, especially for those with type 3 FGM/C, the general norm was to avoid seeking deinfibulation prior to marriage and pregnancy due to the cultural pressure to maintain chastity, prove their virginity and to remain 'closed' for their husbands.[95,98,130,135,136,148] This norm was so strong that some studies reported younger women wishing that they could undergo deinfibulation, experiencing unpleasant symptoms and being aware of services, but feeling unable to resist community pressure.[93,136,146] Some exceptions to this norm were reported, but mainly in the context of needing help for particularly difficult or painful symptoms.[95]

Care seeking in relation to mental health issues associated with FGM/C was infrequently reported and a few studies suggested that this may also be linked to cultural norms in which mental health is still often viewed as a stigmatised issue and, like FGM/C itself, is seen as hard to talk about.[93,133,135,143,149]

For peer review only - http://bmjopen.bmj.com/site/about/guidelines.xhtml

Theme 2.2 Influence of husbands and the family

Nineteen studies reported that access to care in terms of healthcare decision making, especially among Somali women, was strongly influenced by the views and advice of the wider family and peer group. In the maternity context, this had an impact on decision making around uptake of antenatal care and caesarean section.[91,93,95,123,130,133,153] The family also influenced decision making around FGM/C specifically. For example, as mentioned above, women considering premarital deinfibulation reported that they might avoid informing their direct family members as they were aware that they would otherwise come under strong pressure to change their mind due to fears over their future marital prospects.[96,146] For married women with type 3 FGM, studies indicated that the views of husbands were of paramount importance and that women generally felt that they needed their husband's permission to undergo deinfibulation (outside of the context of delivery).[146]

Theme 2.3 Knowledge and information about FGM/C services

Women's knowledge of, and familiarity with, health services was variable. In some cases, women lacked familiarity with the host country health system and this impeded their ability to access care in general. In other cases, women reported being quite familiar with maternity and primary care services, but lacked knowledge and information of FGM/C-specific specialist services (especially non-maternity related services such as counselling) and where and when it may be appropriate to seek help.[93,143,144] One study from the UK suggested that this might be a particular issue for women living in low prevalence areas.[142]

Theme 2.4 'Hit & miss' care

When accessing healthcare, women reported that (i) the identification of FGM/C and (ii) provision of appropriate treatment or referrals could be a "hit and miss" process, depending upon individual provider characteristics and practices - rather than being a result of standardised organisational systems and processes. For example, many studies cited situations where opportunities to identify FGM/C during a consultation were missed, which was attributed to providers lacking awareness of FGM (or related specialist services) and hence failing to ask the right questions, or to undertake an examination, or to make timely referrals.[143] Maternity services were more likely to have knowledgeable providers and responsive reporting and follow up systems, but even here, women described situations where

For peer review only - http://bmjopen.bmj.com/site/about/guidelines.xhtml

they felt their FGM/C had been mismanaged due to lack of awareness and lack of appropriate referrals.[96,115,139,143] The most common issue was failure to have been asked about FGM/C so that it was not included in birth planning discussions and was identified only once labour had started.[92] This theme, was reported in 38 studies, across different countries and time periods.

Analytical theme 3 - Cultural and bodily dissonance: striving for cultural and bodily integrity

This analytical theme refers to changes in and challenges to, women's sense of cultural and bodily identity and integrity as a result of their experiences associated with FGM/C in the host country, and particularly through their encounters with the healthcare system (manifested, for example, during experiences of clinical examinations, childbirth, deinfibulation and reinfibulation). The theme describes women's responses to experiences of dissonance, whereby their decision making and actions can be understood as a desire to maintain a sense of cultural and bodily integrity. This theme highlights differences in, and changes to, cultural meanings and values related to FGM/C and how these differences and changes influence the way that women and health professionals define health issues, make decisions around FGM/C care and experience that care. When mutual interpersonal cultural understanding was not achieved, women perceived sub-optimal care and distrusted the health system and their health providers. If women felt understood, they felt safe and reassured, resulting in a sense of bodily and emotional integrity.

Theme 3.1 Moving from normal to different

Some women described becoming aware of FGM/C as something that is 'different' only once they moved to another culture. At this point, some started to become aware of the suffering and symptoms that their FGM/C may have caused them. They started to question and to resist previously taken for granted aspects of their culture and to feel uncomfortable with their own bodies and sexual identity.[100] However women also reported feeling shame and anger at being labelled as 'different' and 'mutilated' by the dominant discourse in the host country and by health professionals, and felt that their culture was misunderstood.[90,96,116,117] This experience of dissonance made some women feel reluctant to mention FGM/C to health providers or caused them to feel extremely uncomfortable during healthcare consultations, thereby limiting the opportunity to discuss the issues more openly and to achieve greater mutual understanding.[91,100,122] This theme was very common - reported in 40 studies.

For peer review only - http://bmjopen.bmj.com/site/about/guidelines.xhtml

Theme 3.2 Threat to the self: re-living FGM/C pain during clinical interventions

Many studies (n=28) reported women's experiences of gynaecological clinical interventions and childbirth as events which threatened their sense of bodily and cultural integrity. Women described great pain, suffering, fear and apprehension around clinical interventions – and related this to their previous healthcare experiences and, especially, to re-living the original trauma they had experienced during their FGM/C. These emotions were exacerbated in healthcare encounters where women felt a loss of control or lack of respect. Negative experiences could lead to women avoiding attending the hospital until the last minute as a way of avoiding medical interventions.[155]

Theme 3.3 Being opened: complexities of deinfibulation for women with type 3 FGM

The experience of dissonance and quest for integrity was also seen in views and experiences around deinfibulation and was reported by 28 studies. As noted above, for cultural reasons, 'being opened' medically was seen as necessary primarily after marriage and primarily in the context of pregnancy and childbirth - rather than before.[137,138] There were no studies that explicitly or exclusively focused on the views or experiences of unmarried women, but some studies reported examples of unmarried women where the need to maintain cultural and bodily integrity outweighed the experience of physical pain or discomfort, even when women knew about the option to seek surgical deinfibulation.[93,135,136] There were few reported exceptions to this rule, although one study suggested that for some women, finding out about the option for surgical deinfibulation was empowering and deinfibulation was subsequently seen as a way of asserting control over their bodies and lives.[96]

Most studies reported that women preferred to be 'opened' during labour to avoid the pain and trauma of being cut twice.[91,95,98,131,136,146] However, some studies suggested that this view could change towards the medically preferred option of antepartum deinfibulation, if there were opportunities for women to have appropriate discussions, conducted in a sensitive way in the context of a trusting relationship with a healthcare provider.[122,144] Hence, given that FGM/C is often not discussed during healthcare consultations, women's current reported preferences around deinfibulation timing may also reflect the fact that they may not be receiving the right information at the right time, and thus may not be in a good position to make informed decisions on this issue.[96,112,135,144]

For peer review only - http://bmjopen.bmj.com/site/about/guidelines.xhtml

Theme 3.4 Being changed: complexities around reinfibulation

A consistent finding across the studies within this theme (n=19) was that 'being opened' could involve significant emotional, physical, social and relational adjustments as women's bodies became changed. For some women, deinfibulation was firmly welcomed, although still required a period of 'getting used to' a different way of looking and feeling.[100,146] For other women however, (especially women from Sudan where it is a cultural norm in some regions to be closed again after delivery) there was ambivalence about these changes and some women requested to be 'closed again' to varying degrees.[94,96,100,121,123,135,136,146]

In most OECD countries, reinfibulation is illegal and some studies reported women feeling very upset when their requests were denied by health professionals, experiencing this as a denial of their agency and integrity.[112,132] Consequently, women in one study reported seeking medical deinfibulation during holidays back home.[144]

Analytical theme 4 - Disempowering care encounters

In many situations, the care received by women in relation to their FGM/C was experienced as fundamentally disempowering. Women described experiencing negative attitudes or behaviours from health professionals and poorly managed clinical interventions in which they felt they were not respected or listened to. Many studies reported examples of care encounters that left women feeling re-traumatized, voiceless, without power to question interventions, and vulnerable in the hands of unprepared system. Such experiences caused women to feel unsafe and disempowered. There were many examples where women felt that care had gone wrong or where experiences had been poor, and that these situations could have been avoided with better communication, cross-cultural understanding and sensitivity. This analytical theme is made of four descriptive themes that cut across all countries, time periods, population groups and care contexts.

Theme 4.1 Feeling exposed and humiliated

Across many studies (n=40), women reported that the reactions of healthcare professionals to their FGM/C had left them feeling ashamed, objectified, humiliated and exposed. This was primarily the case with healthcare professionals who were unfamiliar with FGM/C and who were encountering it for the first time. In these situations, women reported that healthcare

For peer review only - http://bmjopen.bmj.com/site/about/guidelines.xhtml

professionals often reacted with extreme shock when they saw that a woman had been cut. Some tried to hide their shock and avoided mentioning anything at all making the encounter feel uncomfortable. Others openly expressed horror or disgust or reacted insensitively by asking intrusive or inappropriate questions. In many cases, other colleagues were called in to be consulted or to 'have a look' (sometimes without consent [122,126]) causing women to feel that their privacy had been violated and that they had been put on display. Such experiences left women feeling angry, distressed or as if they had been re-violated.[90,91] In some cases, these experiences left women wanting to avoid further contact with services and/or feeling unable to discuss their care any further as it was clear that the provider had limited knowledge about FGM/C.[129] Several studies made the point that negative experiences of a particular service or provider would then be discussed within peer and community networks, and hence could have an impact beyond the individual woman, potentially affecting the way others in the community might engage with care.[89,91,92,112,127,138,152]

Theme 4.2 Feeling judged and stereotyped

In many studies (n=36) women reported feeling judged and discriminated against within some of their healthcare encounters. They reported feeling that health providers made negative assumptions about them and provided sub-optimal care based on racial, religious or other stereotypes and misconceptions about their culture in general as well as about FGM/C. Such experiences caused distress, anger and avoidance of the health provider or service.[122]

Theme 4.3 Lack of choice, power and control

In this theme, reported by 32 studies, women described experiences where they felt they had lacked choice or control within the healthcare encounter, especially in the maternity setting and around key interventions such as caesarean sections or episiotomies. Lack of control was experienced in terms of feeling excluded from healthcare decision making, not being informed, not being listened to, feeling at the mercy of 'the system' and unable to express their needs. Women's attempts to assert control were sometimes construed by health professionals as being 'difficult'.[91,92,119,126,133] In labour, women described that FGM/C was often not discussed at all or that they were not consulted about interventions such as deinfibulation or caesarean section. This led to suspicion and fear and was particularly reported in studies conducted with the Somali community.[90]

For peer review only - http://bmjopen.bmj.com/site/about/guidelines.xhtml

Theme 4.4 Feeling unsafe and vulnerable

Many studies (n=46) reported care encounters where women felt unsafe and vulnerable, primarily when they were treated by providers who they perceived to be inexperienced in dealing with FGM/C, and when they faced language barriers or were unable to express their own preferences. Such encounters often led to perceived poor quality care or poor clinical outcomes.[124] The consequences ranged from providers failing to recognise, identify or discuss FGM/C,[89] to situations where women felt their care had been adversely affected as a direct result of poor provider skills (for example having unnecessary caesarean sections or extensive perineal tearing).[23,25,89,94,95,99,112,115,135,143,151] Women endured such situations feeling unsafe and highly vulnerable, often describing painful and traumatic experiences.[100] Such feelings of vulnerability were heightened for women who lacked social support.[90,95,122,134] In contrast, women reported feeling safe and highly reassured when they encountered providers who appeared to be knowledgeable or experienced.[89,92,94,96,100,101,124,143,145]

Analytical theme 5 - Positive care encounters

As seen above, the synthesis has revealed many reports of poor care and difficult experiences in relation to FGM/C. However, many studies reported a mixture of experiences, including both good and poor care. High quality care was conceptualised by women as care that is safe, respectful, culturally sensitive and compassionate. The essential process underpinning the experience of 'good' care was having a sense of trust – both in the individual provider and also in the 'system' as a whole. Trust was essential for, and developed from, positive care experiences. Positive experiences were associated with the development of a good relationship with health professionals who were perceived to be clinically knowledgeable and culturally sensitive, facilitating open communication and a sense of being in safe hands. Trust in the 'system' was linked to women's appreciation of the availability and accessibility of services (compared with their home countries) but particularly when services were perceived to be prepared to be responsive to community needs (e.g. provision of appropriate interpretation services) and to manage issues relating to FGM/C (e.g. by having specialist services or specialist providers).

Theme 5.1 Trusting and appreciating providers and the system

This theme, reported in 34 studies showed that women appreciated the good medical services

For peer review only - http://bmjopen.bmj.com/site/about/guidelines.xhtml

available in the host country. Women also expressed great appreciation for providers who made them feel safe and respected. Such providers were described as knowledgeable and experienced, who treated women with respect, who understood their individual needs and who involved them in their care. Women described these characteristics as facilitating trust and leading to open communication about FGM/C. For example, they were more likely to engage in conversations about their care (e.g. birth planning) and, in some cases, were more likely to follow current medical guidelines, for example, on issues such as deinfibulation timing.[134] A few studies explicitly linked the ability to form good relationships to services that enabled continuity of care.[95,124]

Theme 5.2 Voicing healthcare needs and preferences

Women's recommendations for good quality safe FGM/C related care and services they could trust were reported in 45 studies. They included: (i) interpersonal provider characteristics and behaviours (such as providers being willing and able to talk about FGM/C, providers being skilled and knowledgeable around FGM/C, providers offering culturally sensitive and respectful care) and (ii) service organisation issues (such as having specialist services for FGM/C, being given information and awareness about FGM/C-related services, and involving women and affected communities in FGM service development).

CONFIDENCE IN THE REVIEW FINDINGS

Confidence in review findings (as assessed by GRADE CERQual) ranged from high to medium and there was strong consistency in the findings across countries, population groups and clinical contexts (see Table 6 for the final CERQual assessment for each theme and Supplementary File 7 for the full details of the CERQual evaluation).

DISCUSSION: ACHIEVING CULTURALLY SAFE CARE FOR WOMEN WITH FGM/C

The review shows that FGM/C - literally and symbolically - embodies complex processes of cultural change and cultural interaction that are occurring as a result of globalisation, migration and super-diversity. These processes manifest themselves very concretely in healthcare in terms of influencing how individuals seek care, how care is delivered and experienced by all involved, and how services are configured.[2,156,157]

For peer review only - http://bmjopen.bmj.com/site/about/guidelines.xhtml

This review has shown that, when obtaining care for FGM/C-related issues in a host country, women face common challenges that affect many groups of migrants, but that these are exacerbated.[46,84,158-161] Silence, secrecy, stigma and lack of familiarity with FGM/C within the system combine and act as obstacles to identifying FGM/C or to providing women with appropriate care. The care for FGM/C described in this review exemplifies the challenges of achieving 'cultural safety' in health services. Many of the experiences reported in the review describe a situation of physical but also cultural 'vulnerability' or 'risk' whereby women felt physically unsafe but also disrespected in terms of their cultural identity and bodily integrity and un-involved in their care. Many of the themes in this review referred specifically to maternity care contexts, and there are striking similarities with themes identified in studies across the world, that highlight disrespect and abuse in maternal healthcare.[35,36,162] However, as this review shows, the same challenges also appear to exist for women with FGM/C in primary care and other health settings.[163-165].

This review has also shown that, in some circumstances, women and providers can overcome these obstacles. Key to this are knowledge of services, community engagement, the availability of specialist services, continuity of care, shared decision making, addressing language barriers and providing care that is person centred, culturally sensitive and respectful.[44,159,166,167] Similar recommendations have also been made in other studies exploring the key elements of 'respectful care' in a variety of settings.[168,169]

Figure 2 outlines a model, derived from the review findings, of four key elements of 'culturally safe' care for women/girls with FGM/C, set within a wider context of super-diversity.

Figure 2 : Culturally safe care for FGM/C

For peer review only - http://bmjopen.bmj.com/site/about/guidelines.xhtml

RECOMMENDATIONS FOR CULTURALLY SAFE CARE

As per the model in Figure 2, the review recommendations fall into four overlapping areas.

Overcoming barriers: information, awareness and community engagement

The review showed that there is a need for communities to be more aware of FGM/C related services and of potential interventions. However, it also highlighted the challenges that women may have in discussing FGM/C within their family or community and with a health provider. This finding highlights a need for ongoing community involvement and engagement to raise awareness of services and provide support to women who may wish to access help (especially for unmarried or non-pregnant women).[31,32] The review suggests that service models which involve community advocates or community liaison workers may be particularly helpful in encouraging women to access care and to overcome communication difficulties with providers. Similar models have been found to be effective in other areas of healthcare.[170-174]

Breaking the silence: open communication and shared decision making

Given the communication challenges around FGM/C, the review findings strongly suggest that the onus of ‘breaking the silence’ may lie with the healthcare provider, with women stating that they expected and wanted health professionals to raise the issue. It may be appropriate for services to consider routinizing questions around FGM/C in key clinical settings, as has been done for other ‘sensitive’ clinical issues such as domestic violence or HIV.[175-178] In addition, given the particular global concern for women’s (and their babies’) well-being related to timing of deinfibulation surgery (for women with type 3 FGM/C), the review findings strongly suggest that women appreciate, and would benefit from, more discussion, information and advice – at different stages in their life (before and after marriage) and both pre- and post- deinfibulation.

Provider competence and confidence

The review highlighted that providers may lack the confidence or cultural competence to raise the subject of FGM/C in an appropriate and respectful way, potentially missing opportunities to identify FGM/C in a timely manner but also missing opportunities to discuss prevention/safeguarding.[30] Communication challenges can be addressed through staff and

For peer review only - http://bmjopen.bmj.com/site/about/guidelines.xhtml

interpreter training.[179] Likewise, the review showed that providers would benefit from additional training in associated clinical skills such as deinfibulation. The evidence base for best practice in health worker training around FGM/C is still weak,[180] however, as per WHO guidelines,[20] it is suggested that basic training around FGM/C should be embedded in pre-service curricula for all health professionals, as well as being offered as continuous professional development for providers in more specialised roles.

Getting the model of care right: appropriate systems, services and care pathways

The review suggests that women sometimes receive sub-optimal care due to their FGM/C being missed or because of a lack of appropriate referral and reporting pathways. Some countries have made great strides to develop clear processes in this area (e.g. UK), but in others, service provision remains ad-hoc.[21] As per WHO guidelines,[9,20] the review affirms the ongoing need to develop holistic care pathways.[13,26,181-184] The review findings suggest that women have better experiences in places where there are specialist services with confident and competent staff, and that women in lower prevalence or rural areas may be at a particular disadvantage. Hence, it is important for regions to develop service models that can address these inequalities. In addition, the review strongly suggests that respectful open discussions about FGM/C are more likely to happen in a context of a trusting relationship, and that such relationships are facilitated through service models that implement continuity of care approaches and shared decision making.[163-165,185]

The review has highlighted the ongoing need for the availability of mental health assessment and support where required. In particular, the review showed that for some women affected by FGM/C, childbirth and other clinical interventions could be traumatising, triggering flashbacks of their original cutting, creating profound anxiety and distress, and potentially leading to poor care experiences or avoidance of services.[186,187] The review was unable to illuminate in any further depth, how such women might best be identified or treated, or which women might be most at risk. This is an important agenda for future research.[13,39,188-192] However, most maternity care services have well established processes in place for dealing with highly anxious women or women who have experienced trauma. It would seem important for services to ensure that these interventions are also available for women affected by FGM/C.[35,193]

For peer review only

STRENGTHS AND LIMITATIONS

As mentioned above (under 'Rationale'), previous reviews have primarily focused on care experiences within maternity contexts.[35,36,40,41] Our findings on care experiences resonate strongly with these existing reviews. However, we feel that our review has been able to expand upon current knowledge in several ways. First, it adopted an exceptionally comprehensive and exhaustive search and we are confident that the majority of relevant papers were identified. The inclusion of grey literature as well as studies in other languages significantly broadened the evidence base (for example, the existing reviews on maternity contexts included between four and 16 papers, whereas this review included 57).[35,36] It is our contention that this wider evidence base, as well as the broader review focus on all aspects of the lifecourse has enabled a more nuanced understanding to emerge around FGM/C related health experiences. In particular, our review has identified new findings around: (i) factors that influence care-seeking and access to care (as well as the care experience itself), (ii) decision making around deinfibulation surgery, (iii) community perspectives on positive care experiences and suggestions for service improvement, and, (iv) being able to demonstrate the importance of primary care as well as maternity contexts for discussing and identifying FGM/C. The theme matrix presented in Supplementary File 4 provides a visual means for exploring in more detail how the wider evidence base has contributed to particular review findings and, thus, how it has enabled new or more nuanced themes to be identified.

Another strength of the review is its exclusive focus on OECD countries which ensures that its findings are highly relevant to health service development in most destination countries.

The main limitation of the review is that the findings inevitably reflect the methodological limitations of its included studies. Many papers displayed a homogenising tendency in terms of the affected communities as well as FGM/C types. For example, in papers that had mixed community samples, there was little attempt to explore cultural differences in relation to FGM/C. Likewise, it was not always clear which type of FGM/C was being studied. In research with Somali or Sudanese communities, the assumption can reasonably be made that their focus was FGM/C type 3. However, in studies with mixed samples, there was very little differentiation on the basis of FGM/C type. As a result, there is limited specific knowledge on the needs or experiences of women with other FGM/C types.

For peer review only - http://bmjopen.bmj.com/site/about/guidelines.xhtml

EVIDENCE GAPS AND FUTURE RESEARCH

The review has identified five key gaps in the evidence base around FGM/C, all of which are priority areas for future research. First, there were very few studies that focused specifically on mental health needs. Second, there is a lack of studies on girls' or unmarried/non-pregnant women's experiences around FGM. Important areas for future investigation amongst this group are care seeking and decision making (especially around deinfibulation timing). Third, there is a lack of knowledge on potential health needs or experiences of older women, hence this is an area that may require further investigation. Fourth, there were no in-depth evaluations of interventions or services which means that development of models of 'good care' (e.g.[134,194]) still need to be inferred rather than being based upon sound evidence. Further mapping and evaluation of models of care would be beneficial to understand better their differential impact on accessibility, outcomes, cost and patient satisfaction. Finally, there was no research that specifically explored women's/girls' experiences of healthcare professional's involvement in implementation of legal safe guarding or prevention interventions, which, in some countries (such as the UK), are inserting new complexities into the patient-professional relationship and reportedly undermining trust.[32,33,195-197]

CONCLUSION

This review has identified key challenges but also opportunities for the development of culturally safe and accessible services to improve care for women affected by FGM/C. Future research should involve communities to evaluate existing models of care in order to inform best practice.

ACKNOWLEDGEMENTS

We would like to thank all those who have contributed time and valuable insights to this project. We would particularly like to thank all those who gave up their day to attend the national stakeholder consultation event. Special thanks go to Carol McCormick (FGM/C specialist midwife) for her support and input at the start of this project. We gratefully acknowledge the invaluable contributions of our project advisory group:

- Kinsi Clarke, Nottingham and Nottinghamshire Refugee Forum, co-founder of Nottingham FGM survivor's group

For peer review only - http://bmjopen.bmj.com/site/about/guidelines.xhtml

- Professor Jim Thornton, consultant obstetrician, Nottingham University Hospital Trust and University of Nottingham
- Amanda Wickham (FGM/C specialist midwife), Nottingham University Hospital Trust
- Helen Denness, consultant in public health, Nottingham city council
- Grace Brough, Insight specialist (public health), Nottingham city council, Chair of Nottingham and Nottinghamshire FGM/C Steering Group

CONTRIBUTORS

CE and VN conceived the study. CE obtained funding, oversaw all aspects of the project and contributed to all stages. CE drafted this paper. RT contributed to all stages of the review, including data extraction, coding, quality appraisal and CERQual components. JE designed and executed all the searches. JMc assisted with quality assessment and contributed to the evolving synthesis. GH assisted with quality assessment and contributed to the evolving synthesis. JA provided clinical expertise and contributed to the evolving synthesis and formulation of recommendations/conclusions. VN provided a community perspective and contributed to the evolving synthesis and formulation of recommendations/conclusions.

FUNDING STATEMENT

This project was funded by the NIHR HS&DR Programme (no. 15/137/04). The views expressed in this report are those of the authors and not necessarily those of the NHS, the NIHR or the Department of Health and Social Care.

COMPETING INTERESTS

None declared.

PROVENANCE AND PEER REVIEW

Not commissioned. No additional data available.

DATA SHARING STATEMENT

No additional data are available.

For peer review only

OPEN ACCESS

This is an Open Access article distributed in accordance with the Creative Commons Attribution Non Commercial (CC BY-NC 4.0) license, which permits others to distribute, remix, adapt, build upon this work non-commercially, and license their derivative works on different terms, provided the original work is properly cited and the use is non-commercial. See: http://creativecommons.org/licenses/by-nc/4.0/–

For peer review only

REFERENCES

1. Zeeb H, Makarova N, Brand T, et al. Super-diversity: a new concept for migrant health? *Public Health Forum* 2015;23:124-25.

2. Vertovec S. Super-diversity and its implications. *Ethn Rac Stud* 2007;30:1024-54.

3. Rechel B, Mladovsky P, Ingleby D, et al. Migration and health in an increasingly diverse Europe. *The Lancet* 2013;381:1235-45.

4. Phillimore J. Approaches to health provision in the age of super-diversity: accessing the NHS in Britain's most diverse city. *Crit Soc Policy* 2011;31:5-29.

5. Phillimore J. Migrant maternity in an era of superdiversity: new migrants' access to, and experience of, antenatal care in the West Midlands, UK. *Soc Sci Med* 2016;148:152-9. doi: 10.1016/j.socscimed.2015.11.030

6. United Nations Children's Fund. Female Genital Mutilation/Cutting: A Global Concern. New York: UNICEF, 2016.

7. Van Baelen L, Ortensi L, Leye E. Estimates of first-generation women and girls with female genital mutilation in the European Union, Norway and Switzerland. *Eur J Contracept Reprod Health Care* 2016;21(6):474-82.

8. WHO. Female Genital Mutilation: Fact Sheet. Geneva: World Health Organisation, 2014.

9. WHO. WHO Guidelines on the Management of Health Complications from Female Genital Mutilation. Geneva: WHO, 2016.

10. Royal College of Obstetricians and Gynaecologists. Green-Top Guideline No.53: Female Genital Mutilation and its Management. London: Royal College of Obstetricians and Gynaecologists, 2015.

11. Royal College of Midwives, Royal College of Nursing, Royal College of Obstetricians and Gynaecologists, et al. Intercollegiate Guidelines Tackling FGM in the UK: Intercollegiate Recommendations for Identifying, Recording and Reporting. London, , 2013.

12. Royal College of Nursing. Female Genital Mutilation: An RCN Resource for Nursing and Midwifery Practice (2nd ed). London: Royal College of Nursing, 2015.

13. Abdulcadir J, Rodriguez M, Say L, et al. Research gaps in the care of women with female genital mutilation: an analysis. *Br J Obstet Gynaecol* 2015;122(3):294-303. doi: 10.1111/1471-0528.13217

14. Esu E, Udo A, Okusanya BO, et al. Antepartum or intrapartum deinfibulation for childbirth in women with type III female genital mutilation: a systematic review and meta-analysis. *Int J Gynaecol Obstet* 2017;136 Suppl 1:21-29.

15. Albert J, Bailey E, Duaso M. Does the timing of deinfibulation for women with type 3 female genital mutilation affect labour outcomes? *Br J Midwifery* 2015;23(6):430-37.

For peer review only

For peer review only - http://bmjopen.bmj.com/site/about/guidelines.xhtml

16. Paliwal P, Ali SA, Bradshaw S, et al. Management of type III female genital mutilation in Birmingham, UK: a retrospective audit. *Midwifery* 2014;30(3):282-88.

17. Okusanya BO, Oduwole O, Nwachuku N, et al. Deinfibulation for preventing or treating complications in women living with type III female genital mutilation: a systematic review and meta-analysis. *Int J Gynaecol Obstet* 2017;136 Suppl 1:13-20.

18. Gayle C, Rymer J. Female genital mutilation and pregnancy: associated risks. *Br J Nurs* 2016;25(17):978-83.

19. NHS England. Commissioning Services to Meet the Needs of Women and Girls with FGM. London, 2018.

20. WHO. Care of Girls and Women Living with Female Genital Mutilation: A Clinical Handbook. Geneva: World Health Organisation, 2018.

21. Johansen RE, Mahgoub-Ziyada M, Shell-Duncan B, et al. Health sector involvement in the management of female genital mutilation/cutting in 30 countries. *BMC Health Serv Res* 2018;18:240.

22. Waigwa S, Doos L, Bradbury-Jones C, et al. Effectiveness of health education as an intervention designed to prevent female genital mutilation/cutting (FGM/C): a systematic review. *Reprod Health* 2018;16:62.

23. Ahlberg BM, Krantz I, Lindmark G, et al. 'It's only a tradition': making sense of eradication interventions and the persistence of female 'circumcision' within a Swedish context. *Crit Soc Policy* 2004;24(1):50-78. doi: 10.1107/0261018304039679

24. Berg RC, Denison E. A tradition in transition: factors perpetuating and hindering the continuance of female genital mutilation/cutting (FGM/C) summarized in a systematic review. *Health Care Women Int* 2013;34(10):837-59.

25. Recchia N, McGarry J. "Don't judge me": narratives of living with FGM. *Int J Hum Rights Healthc* 2017;10(1):4-13. doi: 10.1108/Ijhrh-10-2016-0016

26. Baillot H, Murray N, Connelly E, et al. Addressing female genital mutilation in Europe: a scoping review of approaches to participation, prevention, protection and provision of services. *Int J Equity Health* 2018;17(1):21. doi: 10.1186/s12939-017-0713-9

27. Dawson A, Homer CSE, Turkmani S, et al. A systematic review of doctors' experiences and needs to support the care of women with female genital mutilation. *Int J Gynecol Obstet* 2015;131(1):35-40.

28. Dawson A, Turkmani S, Fray S, et al. Evidence to inform education, training and supportive work environments for midwives involved in the care of women with female genital mutilation: a review of global experience. *Midwifery* 2015;31(1):229-38. doi: 10.1016/j.midw.2014.08.012

29. Reig-Alcaraz M, Siles-Gonzalez J, Solano-Ruiz C. A mixed-method synthesis of knowledge, experiences and attitudes of health professionals to female genital mutilation. *J Adv Nurs* 2015 doi: 10.1111/jan.12823

For peer review only - http://bmjopen.bmj.com/site/about/guidelines.xhtml

30. Evans C, Tweheyo R, McGarry J, et al. Crossing cultural divides: A qualitative systematic review of factors influencing the provision of healthcare related to female genital mutilation from the perspective of health professionals. *PLoS One* 2019;14(3):e0211829. doi: 10.1371/journal.pone.0211829 [published Online First: 2019/03/05]

31. Connelly E, Murray N, Baillot H, et al. Missing from the debate? a qualitative study exploring the role of communities within interventions to address female genital mutilation in Europe. *BMJ Open* 2018;8:e021430.

32. Dixon S, Agha K, Ali F, et al. Female genital mutilation in the UK- where are we, where do we go next? Involving communities in setting the research agenda. *Res Involv Engagem* 2018;4:29. doi: 10.1186/s40900-018-0103-5 [published Online First: 2018/09/22]

33. Plugge E, Adam S, El Hindi L, et al. The prevention of female genital mutilation in England: what can be done? *J Public Health (Oxf)* 2018 doi: 10.1093/pubmed/fdy128 [published Online First: 2018/07/31]

34. Evans C, Tweheyo R, McGarry J, et al. What are the experiences of seeking, receiving and providing FGM-related healthcare? Perspectives of health professionals and women/girls who have undergone FGM: protocol for a systematic review of qualitative evidence. *BMJ Open* 2017;7:e018170.

35. Hamid A, Grace K, Warren N. A meta-synthesis of the birth experiences of African immigrant women affected by female genital cutting. *J Midwifery Womens Health* 2018;00:1-11.

36. Turkmani S, Homer CSE, Dawson A. Maternity care experiences and health needs of migrant women from female genital mutilation-practicing countries in high-income contexts: A systematic review and meta-synthesis. *Birth* 2018 doi: 10.1111/birt.12367

37. Smith H, Stein K. Health information interventions for female genital mutilation. *Int J Gynaecol Obstet* 2017;136 Suppl 1:79-82.

38. Smith H, Stein K. Surgical or medical interventions for female genital mutilation. *Int J Gynaecol Obstet* 2017;136 Suppl 1:43-46.

39. Smith H, Stein K. Psychological and counselling interventions for female genital mutilation. *Int J Gynaecol Obstet* 2017;136 Suppl 1:60-64.

40. Jordal M, Wahlberg A. Challenges in providing quality care for women with female genital cutting in Sweden - A literature review. *Sex Reprod Healthc* 2018;17:91-96. doi: 10.1016/j.srhc.2018.07.002 [published Online First: 2018/09/09]

41. Scamell M, Ghumman A. The experience of maternity care for migrant women living with female genital mutilation: A qualitative synthesis. *Birth* 2019;46(1):15-23. doi: 10.1111/birt.12390 [published Online First: 2018/10/09]

42. Suphanchaimat R, Kantamaturapoj K, Putthasri W, et al. Challenges in the provision of healthcare services for migrants: a systematic review through providers' lens. *BMC Health Serv Res* 2015;15:390. doi: 10.1186/s12913-015-1065-z

For peer review only

For peer review only - http://bmjopen.bmj.com/site/about/guidelines.xhtml

43. Robertshaw L, Dhesi S, Jones L. Challenges and facilitators for health professionals providing primary healthcare for refugees and asylum seekers in high-income countries: a systematic review and thematic synthesis of qualitative research. *BMJ Open* 2017;7:e015981.

44. O'Donnell C, Higgins M, Chauhan R, et al. Aylum seekers' expectations of, and trust in, general practice: a qualitative study. *Br J Gen Pract* 2008;58:870-76.

45. Ochieng B. Black African migrants: the barriers with accessing and utilising health promotion services in the UK. *Eur J Public Health* 2013;23(2):265-69.

46. Agudelo-Suarez A, Gil-Gonzalez D, Vives-Cases C, et al. A metasynthesis of qualitative studies regarding opinions and perceptions about barriers and determinants of health services' accessibility in economic migrants. *BMC Health Serv Res* 2012;12:461.

47. Hannes K, Harden A. Multi-context versus context-specific qualitative evidence syntheses: combining the best of both. *Res Synth Methods* 2011;2(4):271-78. doi: 10.1002/jrsm.55

48. Weir MC, Grimshaw JM, Mayhew A, et al. Decisions about lumping vs. splitting of the scope of systematic reviews of complex interventions are not well justified: a case study in systematic reviews of health care professional reminders. *J Clin Epidemiol* 2012;65(7):756-63. doi: 10.1016/j.jclinepi.2011.12.012 [published Online First: 2012/04/14]

49. Joanna Briggs Institute. JBI Reviewer's Manual https://wiki.joannabriggs.org/display/MANUAL/Chapter+2%3A+Systematic+reviews+of+qualitative+evidence: (accessed 12-07-18); [

50. Noyes J, Hannes K, Booth A, et al. Qualitative and Implementation Evidence and Cochrane Reviews, Version 1.02a. In: Higgins JA, Green S, eds. Cochrane Handbook for Systematic Reviews of Interventions Version 5/3 (updated October 2015): The Cochrane Collaboration, 2015.

51. Ramsden I. Cultural Safety and Nursing Education in Aotearoa and Te Waipounamu. Victoria University, 2002.

52. Browne A, Varcoe C, Smye V, et al. Cultural safety and the challenges of translating critically oriented knowledge into practice. *Nurs Philos* 2009;167-179

53. Phiri J, Dietsch E, Bonner A. Cultural safety and its importance for Australian midwifery practice. *Collegian* 2010;17(3):105-11.

54. Duke J, Connor M, McEldowney R. Becoming a culturally competent health practitioner in the delivery of culturally safe care. *J Cult Divers* 2009;16:40-49.

55. Gerlach A. A critical reflection on the concept of cultural safety. *Can J Occup Ther* 2012;79(3):151-58.

56. Cooney C. A comparative analysis of transcultural nursing and cultural safety. *Nurs Prax N Z* 1994;9(1):6-11.

For peer review only - http://bmjopen.bmj.com/site/about/guidelines.xhtml

57. Brascoupé S, Waters C. Cultural safety: exploring the applicability of the concept of cultural safety to aboriginal health and community wellness. *J Aborig Health* 2009(November):6-41.

58. Wepa D. Cultural Safety in Aotearoa New Zealand. North Shore City: Pearson Prentice Hall 2005.

59. Johnston M, Kanitsaki O. An exploration of the notion and nature of the construct of cultural safety and its applicability to the Australian healthcare context. *J Transcult Nurs* 2007;18:247-56.

60. Kinn LG, Holgersen H, Ekeland TJ, et al. Metasynthesis and bricolage: an artistic exercise of creating a collage of meaning. *Qual Health Res* 2013;23(9):1285-92. doi: 10.1177/1049732313502127 [published Online First: 2013/08/22]

61. France EF, Uny I, Ring N, et al. A methodological systematic review of meta-ethnography conduct to articulate the complex analytical phases. *BMC Med Res Methodol* 2019;19(1):35. doi: 10.1186/s12874-019-0670-7

62. Guba E, Lincoln Y. Competing paradigms in qualitative research. In: Denzin N, Lincoln Y, eds. Handbook of Qualitative Research. London: Sage 1994:105-17.

63. Carroll C, Booth A, Leaviss A, et al. “Best fit” framework synthesis: refining the method. *BMC Med Res Methodol* 2013;13(37)

64. Reeves S, Albert M, Kuper A, et al. Why use theories in qualitative research? *BMJ* 2008;337:a949.

65. Maxwell J. Qualitative Research Design: An Interactive Approach. Los Angeles: Sage 2013.

66. Giaconni M. Theory matters in qualitative health research. In: Bourgeault I, Dingwall R, De Vries R, eds. The Sage Handbook of Qualitative Methods in Health Research. London: Sage 2010:125-55.

67. Oliver S, Dickson K, Bangpan M. Systematic reviews: making them policy relevant. A briefing for policy makers and systematic reviewers. London: EPPI-Centre, Social Science Research Unit, UCL Institute of Education, University College London, 2015.

68. Tong A, Flemming K, McInnes E, et al. Enhancing transparency in reporting the synthesis of qualitative research: ENTREQ. *BMC Med Res Methodol* 2012;12(1):181. doi: 10.1186/1471-2288-12-181

69. Evans C, McGarry J, McCormick C, et al. The experiences of healthcare across the life course for women and girls who have undergone female genital mutilation: a qualitative systematic review Protocol, PROSPERO, CRD42015030001, 2015.

70. National Information Center on Health Services Research and Healthcare Technology (NICHSR). Health Services Research and Health Policy Grey Literature Project: Summary Report: U.S. National Library of Medicine, 2006.

For peer review only - http://bmjopen.bmj.com/site/about/guidelines.xhtml

71. Adams J, Hillier-Brown FC, Moore HJ, et al. Searching and synthesising 'grey literature' and 'grey information' in public health: critical reflections on three case studies. *Syst Rev* 2016;5(1):164. doi: 10.1186/s13643-016-0337-y

72. Mahmood Q, Van Eerd D, Irvin E. Searching for grey literature for systematic reviews: challenges and benefits. *Res Synth Method* 2013;5(3):221-34.

73. Adams RJ, Smart P, Huff AS. Shades of grey: guidelines for working with the grey literature in systematic reviews for management and organizational studies. *Int J Manag Rev* 2017;19(4):432-54. doi: 10.1111/ijmr.12102

74. Pearson A, Robertson-Malt S, Rittenmeyer L. Synthesizing Qualitative Evidence. Philadelphia: Lippincott-Joanna Briggs Institute 2011.

75. Joanna Briggs Institute. JBI QARI Critical Appraisal Checklist for Interpretive and Critical Research https://wiki.joannabriggs.org/display/MANUAL/Joanna+Briggs+Institute+Reviewer%27s+Manual: (accessed 08-06-18); [cited included reference from Health FGM 170518.enl. Available from: http://joannabriggs.org/assets/docs/jbc/operations/criticalAppraisalForms/JBC_Form_CritAp_IntCrit.pdf.

76. Munthe-Kaas H, Bohren MA, Glenton C, et al. Applying GRADE-CERQual to qualitative evidence synthesis findings—paper 3: how to assess methodological limitations. *Implement Sci* 2018;13(1):9. doi: 10.1186/s13012-017-0690-9

77. Lewin S, Glenton C, Munthe-Kaas H, et al. Using qualitative evidence in decision making for health and social interventions: an approach to assess confidence in findings from qualitative evidence syntheses (GRADE-CERQUAL). *PLoS Med* 2015;12(10):e1001895.

78. Dixon-Woods M, Sutton A, Shaw R, et al. Appraising qualitative research for inclusion in systematic reviews: a quantitative and qualitative comparison of three methods. *J Health Serv Res Policy* 2007;12 doi: 10.1258/135581907779497486

79. Dixon-Woods M, Booth A, Sutton AJ. Synthesizing qualitative research: a review of published reports. *Qual Res* 2007;7 doi: 10.1177/1468794107078517

80. Dixon-Woods M, Shaw RL, Agarwal S, et al. The problem of appraising qualitative research. *Qual Saf Health Care* 2004;13 doi: 10.1136/qshc.2003.008714

81. Sandelowski M, Barroso J. Reading qualitative studies. *Int J Qual Methods* 2002;1:74-108.

82. Noyes J, Booth A, Flemming K, et al. Cochrane Qualitative and Implementation Methods Group guidance paper 3: methods for assessing methodological limitations, data extraction and synthesis, and confidence in synthesized qualitative findings. *J Clin Epidemiol* 2017 doi: 10.1016/j.jclinepi.2017.06.020

83. Higginbottom G, Morgan M, Alexandre M, et al. Immigrant women's experiences of maternity-care services in Canada: a systematic review using a narrative synthesis. *Syst Rev* 2015;4(1):1-13.

For peer review only - http://bmjopen.bmj.com/site/about/guidelines.xhtml

84. Higginbottom GMA, Hadziabdic E, Yohani S, et al. Immigrant women's experience of maternity services in Canada: a meta-ethnography. *Midwifery* 2014;30(5):544-59. doi: 10.1016/j.midw.2013.06.004

85. Popay J, Rogers A, Williams G. Rationale and standards for the systematic review of qualitative literature in health services research. *Qual Health Res* 1998;8 doi: 10.1177/104973239800800305

86. Noyes J, Popay J. Directly observed therapy and tuberculosis: how can a systematic review of qualitative research contribute to improving services? a qualitative meta-synthesis. *J Adv Nurs* 2007;57(227-216)

87. Sandelowski M, Barroso J. Finding the findings in qualitative studies. *J Nurs Scholarsh* 2002;34:213-19.

88. Thomas J, Harden A. Methods for the thematic synthesis of qualitative research in systematic reviews. *BMC Med Res Methodol* 2009;8:45.

89. Baldeh F. Obstetric Care in Scotland: The Experience of Women who have Undergone Female Genital Mutilation (FGM) [MSc Thesis]. Queen Margaret University, 2013.

90. d'Entremont M, Smythe L, McAra-Couper J. The sounds of silence: a hermeneutic interpretation of childbirth post excision. *Health Care Women Int* 2014;35(3):300-19. doi: 10.1080/07399332.2013.838245

91. Fawcett L. Somali Refugee Women and their U.S. Healthcare Providers: Knowledge, Perceptions and Experiences of Childbearing [Doctor of Philosophy]. Arizona State University, 2014.

92. Glover J, Liebling H, Barrett H, et al. The psychological and social impact of female genital mutilation: a holistic conceptual framework. *J Int Stud* 2017;10(2):219-38.

93. Jones A. Working Psychologically with Female Genital Mutilation: An Exploration of the Views and Experiences of Women who have Experienced FGM and of Clinical Psychologists. University of East London, 2010.

94. Lundberg PC, Gerezgiher A. Experiences from pregnancy and childbirth related to female genital mutilation among Eritrean immigrant women in Sweden. *Midwifery* 2008;24(2):214-25. doi: 10.1016/j.midw.2006.10.003

95. Moxey JM, Jones LL. A qualitative study exploring how Somali women exposed to female genital mutilation experience and perceive antenatal and intrapartum care in England. *BMJ Open* 2016;6(1):e009846. doi: 10.1136/bmjopen-2015-009846

96. Palfreyman A, Brown E, Nam S. Understanding Female Genital Mutilation in Birmingham: Findings from a PEER Study: Options Consultancy Services and Birmingham & Solihull Women’s Aid, 2011.

97. Straus L, McEwen A, Hussein FM. Somali women's experience of childbirth in the UK: perspectives from Somali health workers. *Midwifery* 2009;25(2):181-6. doi: 10.1016/j.midw.2007.02.002

For peer review only

For peer review only - http://bmjopen.bmj.com/site/about/guidelines.xhtml

98. Thierfelder C. Female Genital Mutilation and the Swiss Health Care System. University of Basel, 2003.

99. Vangen S, Johansen REB, Sundby J, et al. Qualitative study of perinatal care experiences among Somali women and local health care professionals in Norway. *Eur J Obstet Gynecol Reprod Biol* 2004;112(1):29-35. doi: 10.1016/S0301-2115(03)00313-0

100. Vaughan C, White N, Keogh L, et al. Listening to North Yarra Communities about Female Genital Cutting. Melbourne, Australia: The University of Melbourne, 2014.

101. Vaughan C, White N, Keogh L, et al. Female Genital Mutilation/Cutting in Regional Victoria. Research to Practice. Melbourne, Australia: The University of Melbourne, 2014.

102. Boyatzis R. Transforming Qualitative Data: Thematic Analysis and Code Development. London: Sage 1998.

103. Braun V, Clarke V. Using thematic analysis in psychology. *Qual Res Psychol* 2006;3(2):77-101.

104. Booth A, Carroll C, Ilott I, et al. Desperately seeking dissonance: identifying the disconfirming case in qualitative evidence synthesis. *Qual Health Res* 2013;23(1):126-41.

105. Noyes J, Booth A, Lewin S, et al. Applying GRADE-CERQual to qualitative evidence synthesis findings–paper 6: how to assess relevance of the data. *Implement Sci* 2018;13(1):4. doi: 10.1186/s13012-017-0693-6

106. Noyes J, Booth A, Flemming K, et al. Cochrane Qualitative and Implementation Methods Group guidance series-paper 3: methods for assessing methodological limitations, data extraction and synthesis, and confidence in synthesized qualitative findings. *J Clin Epidemiol* 2018;97:49-58. doi: 10.1016/j.jclinepi.2017.06.020

107. Lewin S, Booth A, Glenton C, et al. Applying GRADE-CERQual to qualitative evidence synthesis findings: introduction to the series. *Implement Sci* 2018;13(1):2. doi: 10.1186/s13012-017-0688-3

108. Lewin S, Bohren M, Rashidian A, et al. Applying GRADE-CERQual to qualitative evidence synthesis findings—paper 2: how to make an overall CERQual assessment of confidence and create a Summary of Qualitative Findings table. *Implement Sci* 2018;13(1):10. doi: 10.1186/s13012-017-0689-2

109. Glenton C, Carlsen B, Lewin S, et al. Applying GRADE-CERQual to qualitative evidence synthesis findings—paper 5: how to assess adequacy of data. *Implement Sci* 2018;13(1):14. doi: 10.1186/s13012-017-0692-7

110. Colvin CJ, Garside R, Wainwright M, et al. Applying GRADE-CERQual to qualitative evidence synthesis findings—paper 4: how to assess coherence. *Implement Sci* 2018;13(1):13. doi: 10.1186/s13012-017-0691-8

For peer review only

111. Booth A, Lewin S, Glenton C, et al. Applying GRADE-CERQual to qualitative evidence synthesis findings–paper 7: understanding the potential impacts of dissemination bias. *Implement Sci* 2018;13(1):12. doi: 10.1186/s13012-017-0694-5

112. Asefaw F. [Female Genital Mutilation: A Field Study with Special Consideration of the Background as well as the Health and Psychosexual Consquences for those Affected and their Partners in Eritrea and in Germany] Weibliche Genitalbeschneidung: Eine Feldstudie Unter besonderer Berücksichtigung der Hintergründe Sowie der Gesundheitlichen und Psychosexuellen Folgen für Betroffene und Partner in Eritrea und Deutschland. Humboldt University of Berlin, 2007.

113. Maier C. [Echoes of Silence: Voices of Concern for Genital Mutilation in African Immigrant Women in Vienna: An Ethnological Study] Echo des Schweigens: Stimmen der Betroffenheit zur Genitalverstümmelung bei Afrikanischen Immigrantinnen in Wien: Ethnologische Studie. University of Vienna, 2003.

114. Ballesteros Meseguer C, Almansa Martinez P, Pastor Bravo MdM, et al. [The voice of women subjected to female genital mutilation in the region of Murcia, Spain] La Voz de las mujeres sometidas a mutilacion genital femenina en la region de Murcia. *Gac Sanit* 2014;28(4):287-91. doi: 10.1016/j.gaceta.2014.02.006

115. Bravo Pastor del Mar M. [Women Subjected to Female Genital Mutilation: Knowledge for the Nursing Discipline] La Voz de las Mujeres Sometidas a Mutilación Genital Femenina: Saberes Para la Disciplina Enfermera [Tesis Doctoral]. University of Murcia, 2014.

116. Abdi R. Carving culture: Creating identity through female genital cutting. *Durham Anthropology Journal* 2012;18(1):115-53.

117. Abdullahi A, Copping J, Kessel A, et al. Cervical screening: perceptions and barriers to uptake among Somali women in Camden. *Public Health* 2009;123(10):680-5. doi: 10.1016/j.puhe.2009.09.011

118. Ahmed M. Attitudes towards FGM among Somali women living in the UK. In: Momoh C, ed. Female Genital Mutilation. London: Radcliffe 2005:95-117.

119. Ameresekere M, Borg R, Frederick J, et al. Somali immigrant women's perceptions of cesarean delivery and patient-provider communication surrounding female circumcision and childbirth in the USA. *Int J Gynecol Obstet* 2011;115(3):227-30. doi: 10.1016/j.ijgo.2011.07.019

120. Behrendt A. Listening to African Voices: Female Genital Mutilation/Cutting among Immigrants in Hamburg: Knowledge, Attitudes and Practice. Hamburg, Germany: Plan, 2011.

121. Beine K, Fullerton J, Palinkas L, et al. Conceptions of prenatal care among Somali women in San Diego. *J Nurs Midwifery* 1995;40(4):376-81. doi: Doi 10.1016/0091-2182(95)00024-E

122. Berggren V, Bergstrom S, Edberg A-K. Being different and vulnerable: experiences of immigrant African women who have been circumcised and sought maternity care in Sweden. *J Transcult Nurs* 2006;17(1):50-7. doi: 10.1177/1043659605281981

For peer review only - http://bmjopen.bmj.com/site/about/guidelines.xhtml

123. Betts V. Transnational Gender, Sexuality and Identity Construction: Ambivalent Subjectivities of Somali Refugee Women in the GTA. MA Thesis, The University of Guelph, 2011.

124. Bulman KH, McCourt C. Somali refugee women's experiences of maternity care in west London: a case study. *Crit Public Health* 2002;12(4):365-80.

125. Carroll J, Epstein R, Fiscella K, et al. Caring for Somali women: implications for clinician-patient communication. *Patient Educ Couns* 2007;66(3):337-45. doi: 10.1016/j.pec.2007.01.008

126. Chalmers B, Omer-Hashi K. What Somali women say about giving birth in Canada. *J Reprod Infant Psychol* 2002;20(4):267-82. doi: 10.1080/0264683021000033183

127. Degni F, Suominen SB, El Ansari W, et al. Reproductive and maternity health care services in Finland: perceptions and experiences of Somali-born immigrant women. *Ethn Health* 2014;19(3):348-66. doi: 10.1080/13557858.2013.797567

128. Essén B, Johnsdotter S, Hovelius B, et al. Qualitative study of pregnancy and childbirth experiences in Somalian women resident in Sweden. *Br J Obstet Gynaecol* 2000;107(12):1507-12.

129. Gali MA. Female Circumcision: A Transcultural Study of Attitudes, Identity and Reproductive Health of East African Immigrants [Degree of Doctor of Philosophy in Psychology]. The Wright Institute, University of Berkeley, 1997.

130. Ghebre RG, Sewali B, Osman S, et al. Cervical cancer: barriers to screening in the Somali community in Minnesota. *J Immigr Minor Health* 2015;17(3):722-28. doi: 10.1007/s10903-014-0080-1

131. Glazer E. Embodiment, Pain and Circumcision in Somali-Canadian Women [M.Sc.]. University of Toronto, 2012.

132. Guerin PB, Allotey P, Elmi FH, et al. Advocacy as a means to an end: assisting refugee women to take control of their reproductive health needs. *Women Health* 2006;43(4):7-25.

133. Hill N, Hunt E, Hyrkas K. Somali immigrant women's health care experiences and beliefs regarding pregnancy and birth in the United States. *J Transcult Nurs* 2012;23(1):72-81. doi: 10.1177/1043659611423828 [published Online First: 2011/11/05]

134. Hussein E. Women's Experiences, Perceptions and Attitudes of Female Genital Mutilation: The Bristol PEER Study. London: FORWARD, 2010.

135. Hussen MA. Services for Women with Female Genital Mutilation in Christchurch: Perspectives of Women and their Health Providers. University of Canterbury, 2014.

136. Johansen RE. Undoing female genital cutting: perceptions and experiences of infibulation, defibulation and virginity among Somali and Sudanese migrants in Norway. *Cult Health Sex* 2017;19(4):528-42. doi: 10.1080/13691058.2016.1239838

For peer review only

For peer review only - http://bmjopen.bmj.com/site/about/guidelines.xhtml

137. Khaja K. Female Circumcision: Life Histories of Somali Women [Doctor of Philosophy]. The University of Utah, 2004.

138. Khaja K, Lay K, Boys S. Female circumcision: toward an inclusive practice of care. *Health Care Women Int* 2010;31(8):686-99. doi: 10.1080/07399332.2010.490313

139. Maternity Action. Women's Voices on Health: Addressing Barriers to Accessing Primary Care. London: Women's Health and Equality Consortium, 2014.

140. McNeely S, Christie-de Jong F. Somali refugees' perspectives regarding FGM/C in the US. *Int J Migr Health Soc Care* 2016;12(3):157-69. doi: 10.1108/Ijmhsc-09-2015-0033

141. Murray L, Windsor C, Parker E, et al. The experiences of African women giving birth in Brisbane, Australia. *Health Care Women Int* 2010;31(5):458-72. doi: 10.1080/07399330903548928

142. Norman K, Gegzabher SB, Otoo-Oyortey N. "Between Two Cultures": A Rapid PEER Study Exploring Migrant Communities' Views on Female Genital Mutilation in Essex and Norfolk, UK. FORWARD & National FGM Centre Report, , 2016.

143. Norman K, Hemmings J, Hussein E, et al. "FGM is Always With Us": Experiences, Perceptions and Beliefs of Women Affected by Female Genital Mutilation in London: Results from a PEER Study. London: Options Consultancy Services and FORWARD, 2009.

144. O'Brien O, Baldeh F, Hassan J, et al. My Voice: Participatory Action Research Project with Men, Women and Young People on Female Genital Mutilation (FGM) in Scotland: (Phase 2). Waverly Care, Edinburgh: Queen Margaret University, 2017.

145. O'Brien O, Baldeh F, Sivapatham S, et al. Participatory Action Research Project with Men, Women and Young People on Female Genital Mutilation (FGM) in Scotland: (Phase 1). Waverly Care, Edinburgh: Queen Margaret University, 2016.

146. Safari F. A qualitative study of women's lived experience after deinfibulation in the UK. *Midwifery* 2013;29(2):154-8. doi: 10.1016/j.midw.2011.12.005

147. Salad J, Verdonk P, de Boer F, et al. "A Somali girl is Muslim and does not have premarital sex - is vaccination really necessary?" A qualitative study into the perceptions of Somali women in the Netherlands about the prevention of cervical cancer. *Int J Equity Health* 2015;14(1):1-13. doi: 10.1186/s12939-015-0198-3

148. Shaw E. Female circumcision: perceptions of clients and caregivers. *J Am Coll Health* 1985;33(5):193-7. doi: 10.1080/07448481.1985.9939604

149. Shermarke MAA. Understanding the Canadian Community Context of Female Circumcision [M.S.W.]. McGill University, 1996.

150. Thierfelder C, Tanner M, Bodiang CMK. Female genital mutilation in the context of migration: experience of African women with the Swiss health care system. *Eur J Public Health* 2005;15(1):86-90. doi: 10.1093/eurpub/cki120

For peer review only - http://bmjopen.bmj.com/site/about/guidelines.xhtml

151. Upvall MJ, Mohammed K, Dodge PD. Perspectives of Somali Bantu refugee women living with circumcision in the United States: a focus group approach. *Int J Nurs Stud* 2009;46(3):360-8. doi: 10.1016/j.ijnurstu.2008.04.009

152. Vloeberghs E, van der Kwaak A, Knipscheer J, et al. Coping and chronic psychosocial consequences of female genital mutilation in the Netherlands. *Ethn Health* 2012;17(6):677-95. doi: 10.1080/13557858.2013.771148

153. Wiklund H, Aden AS, Högberg U, et al. Somalis giving birth in Sweden: a challenge to culture and gender specific values and behaviours. *Midwifery* 2000;16(2):105-15. doi: http://dx.doi.org/10.1054/midw.1999.0197

154. Lincoln Y, Guba E. Naturalistic Inquiry. California: Sage 1985.

155. Essen B, Binder P, Johnsdotter S. An anthropological analysis of the perspectives of Somali women in the West and their obstetric care providers on caesarean birth. *J Psychosom Obst Gyn* 2011;32(1):10-18. doi: 10.3109/0167482x.2010.547966

156. Green G, Davison C, Bradby H, et al. Pathways to care: how superdiversity shapes the need for navigational assistance. *Sociol Health Illn* 2014;36:1205-19.

157. Phillimore J, Klaas F, Padilla B, et al. Adaptation of Health Services to Diversity: An Overview of Approaches, Working Paper No.15. University of Birmingham: Institute for Research into Superdiversity, 2016.

158. Benza S, Liamputtong P. Pregnancy, childbirth and motherhood: a meta-synthesis of the lived experiences of immigrant women. *Midwifery* 2014;30(6):575-84.

159. Wikberg A, Bondas T. A patient perspective in research on intercultural caring in maternity care: a meta-ethnography. *Int J Qual Stud Health Well-Being* 2010;5(1):15. doi: 10.3402/qhw.v5i1.4648

160. Balaam M-C, Akerjordet K, Lyberg A, et al. A qualitative review of migrant women's perceptions of their needs and experiences related to pregnancy and childbirth. *J Adv Nurs* 2013;69(9):1919-30. doi: 10.1111/jan.12139

161. Higginbottom GMA, Safipour J, Yohani S, et al. An ethnographic study of communication challenges in maternity care for immigrant women in rural Alberta. *Midwifery* 2015;31(2):297-304. doi: 10.1016/j.midw.2014.09.009

162. Bohren MA, Vogel JP, Hunter EC, et al. The mistreatment of women during childbirth in health facilities globally: a mixed-methods systematic review. *PLoS Med* 2015;12(6):e1001847. doi: 10.1371/journal.pmed.1001847

163. Department of Health. Midwifery 2020: Delivering Expectations. London, , 2010.

164. NHS England. National Maternity Review. Better Births: Improving Outcomes of Maternity Services in England. London, , 2016.

165. Mainous A, Baker R, Love M. Continuity of care and trust in one's physician: evidence from primary care in the United States and the United Kingdom. *Fam Med* 2001;33:22-27.

For peer review only - http://bmjopen.bmj.com/site/about/guidelines.xhtml

166. Gilson L. Trust and the development of health care as an institution. *Soc Sci Med* 2003;56:1458-68.

167. Murray B, McCrone S. An integrative review of promoting trust in patient-primary care provider relationships. *J Adv Nurs* 2015;71:3-23.

168. Denny E. Respectful maternity care needs to be the standard for all women worldwide (mini-commentary). *Br J Obstet Gynaecol* 2017;Dec 12 doi: 10.1111/1471-0528.15055

169. Shakibazadeh E, Namadian M, Bohren M, et al. Respectful care during childbirth in health facilities globally: a qualitative evidence synthesis. *Br J Obstet Gynaecol* 2017 doi: https://doi.org/10.1111/1471-0528.15015

170. Martin M, Phelan M. Interpreters and cultural mediators: different but complementary roles. *Migr Health* 2010;6(1):1-8.

171. Akhavan S, Edge D. Foreign born women's experiences of community based Doulas in Sweden: a qualitative study. *Health Care Women Int* 2012;33(9):833-48. doi: http://dx.doi.org/10.1080/07399332.2011.646107

172. Akhavan S, Lundgren I. Midwives' experiences of doula support for immigrant women in Sweden: a qualitative study. *Midwifery* 2012;28(1):80-85. doi: 10.1016/j.midw.2010.11.004

173. Dundek LH. Establishment of a Somali doula program at a large metropolitan hospital. *J Perinat Neonatal Nurs* 2006;20(2):128-37.

174. Shelp A. Women helping women: the Somali doula initiative. *Int J Childbirth Educ* 2004;19(4):4-7.

175. Evans C, Nalubega S, McLuskey J, et al. The views and experiences of nurses and midwives in the provision and management of provider-initiated HIV testing and counseling: a systematic review of qualitative evidence. *JBI Database System Rev Implement Rep* 2016;13:130-286.

176. WHO. Service Delivery Approaches to HIV Testing and Counselling (HTC): A Strategic Programme Framework. Geneva: World Health Organisation, 2012.

177. Hamberger LK, Rhodes K, Brown J. Screening and intervention for intimate partner violence in healthcare settings: creating sustainable system-level programs. *J Womens Health* 2015;24(1):86-91. doi: 10.1089/jwh.2014.4861

178. O'Doherty L, Hegarty K, Ramsay J, et al. Screening women for intimate partner violence in healthcare settings. *Cochrane Database Syst Rev* 2015;7(CD007007)

179. Karliner L, Jacobs E, Chen A. Do professional interpreters improve clinical care for patients with limited English proficiency? a systematic review of the literature. *Health Serv Res* 2007;42:727-54.

180. Abdulcadir J, Say L, Pallitto C. What do we know about assessing healthcare students and professionals' knowledge, attitude and practice regarding female genital

For peer review only - http://bmjopen.bmj.com/site/about/guidelines.xhtml

mutilation? A systematic review. *Reprod Health* 2017;14:1-13. doi: 10.1186/s12978-017-0318-1

181. Abdulcadir J, Rodriguez MI, Say L. A systematic review of the evidence on clitoral reconstruction after female genital mutilation/cutting. *Int J Gynaecol Obstet* 2015;129(2):93-97.

182. De Schrijver L, Leye E, Merckx M. A multidisciplinary approach to clitoral reconstruction after female genital mutilation: the crucial role of counselling. *Eur J Contracept Reprod Health Care* 2016;21(4):269-75. doi: http://dx.doi.org/10.3109/13625187.2016.1172063

183. Dominguez Garcia L, Dominguez L, Hopkins K, et al. Meeting the sexual health needs of women with female genital mutilation: an integrated model of care. *Int J STD AIDS* 2013;24:2.

184. Low-Beer N, Dominguez-Garcia L, Alcayde D, et al. Accessible integrated care to meet the complex needs of women with female genital mutilation (FGM). *Br J Obstet Gynaecol* 2013;120:314.

185. Saha S, Beach M, Cooper L. Patient centredness, cultural competence and healthcare quality. *J Natl Med Assoc* 2008;100:1275-85.

186. Johansen RE. Pain as a counterpoint to culture: toward an analysis of pain associated with infibulation among Somali immigrants in Norway. *Med Anthropol Q* 2002;16(3):312-40.

187. Johansen RE. Care for infibulated women giving birth in Norway: an anthropological analysis of health workers' management of a medically and culturally unfamiliar issue. *Med Anthropol Q* 2006;20(4):516-44.

188. James S. Women's experiences of symptoms of post-traumatic stress disorder (PTSD) after traumatic childbirth: a review and critical appraisal. *Arch Womens Ment Health* 2015;18(6):216-26.

189. Raja S, Hasnain M, Hoersch M, et al. Trauma informed care in medicine: current knowledge and future research directions. *Fam Community Health* 2015;38(3):216-26.

190. Abdulcadir J, Bianchi Demicheli F, Willame A, et al. Posttraumatic stress disorder relapse and clitoral reconstruction after female genital mutilation. *Int J Gynaecol Obstet* 2017;129(2):371-76.

191. Abayomi O, Chibuzor MT, Okusanya BO, et al. Supportive psychotherapy or client education alongside surgical procedures to correct complications of female genital mutilation: A systematic review. *Int J Gynaecol Obstet* 2017;136 Suppl 1:51-55.

192. Adelufosi A, Edet B, Arikpo D, et al. Cognitive behavioral therapy for post-traumatic stress disorder, depression, or anxiety disorders in women and girls living with female genital mutilation: a systematic review. *Int J Gynaecol Obstet* 2017;136 Suppl 1:56-59.

For peer review only - http://bmjopen.bmj.com/site/about/guidelines.xhtml

193. Jones A, Byrne A. Working psychologically with female genital mutilation: an exploration of the views and experiences of women who have experienced FGM and of clinical psychologists. *Clin Psychol Forum* 2013(246):26-30.

194. Clayton-Hathway K. A Pilot Evaluation of Health Services for Communities Affected by FGM in Oxfordshire: Final Report. Oxford, UK: Healthwatch Oxfordshire, 2016.

195. Gangoli G, Gill A, Mulvihill N, et al. Perception and barriers: reporting female genital mutilation. *Journal of Aggression, Conflict and Peace Research* 2018 doi: 10.1108/JACPR-09-2017-0323

196. Malik Y, Rowland A, Gerry F, et al. Mandatory reporting of female genital mutilation in children in the UK. *British Journal of Midwifery* 2018;26(6):377-86.

197. Karlsen S, Carver N, Mogilnicka M, et al. When safeguarding becomes stigmatising: A report on the impact of FGM-safeguarding procedures on people with a Somali heritage living in Bristol, Policy Report. Bristol: University of Bristol, 2019.

For peer review only

For peer review only - http://bmjopen.bmj.com/site/about/guidelines.xhtml

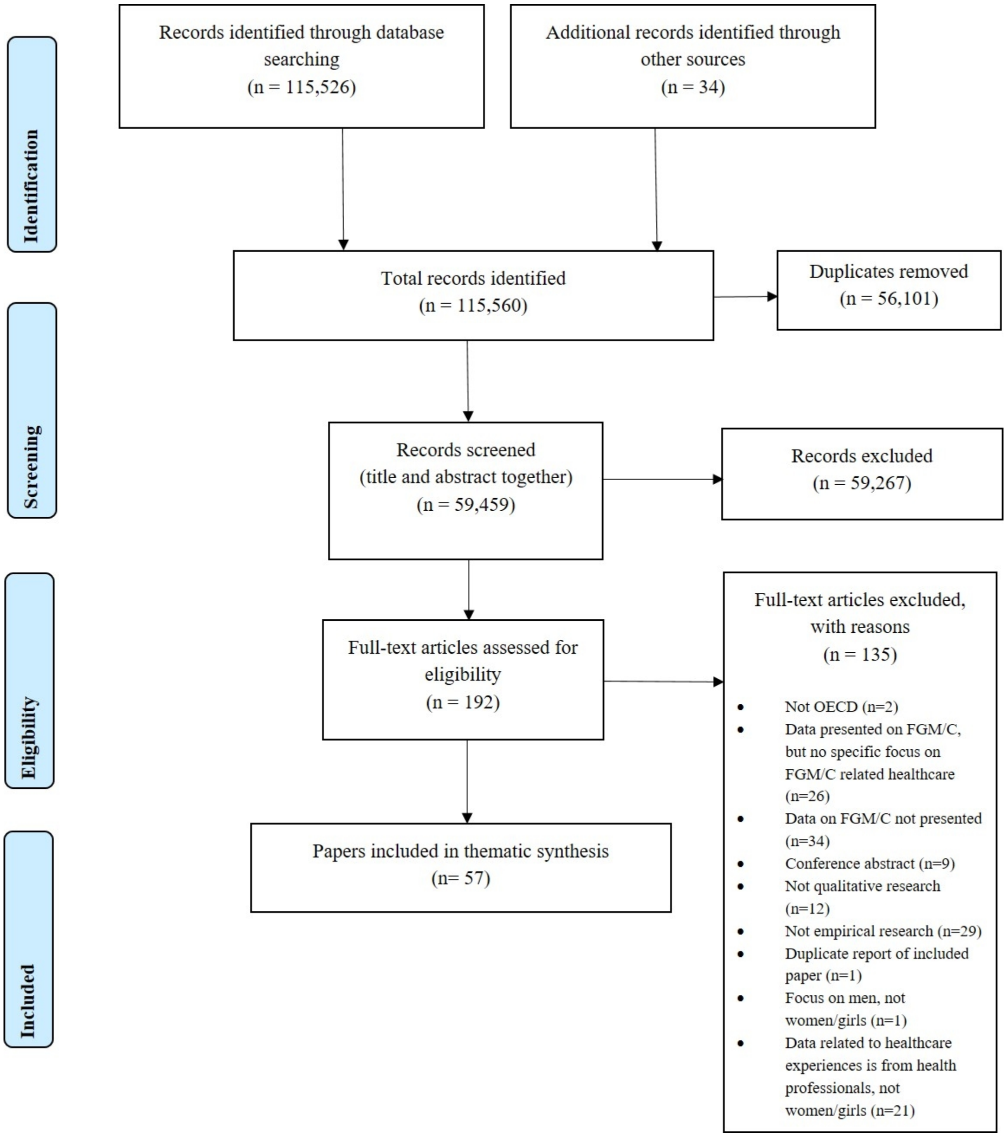

Figure 1: PRISMA Flow diagram

195x220mm (150 x 150 DPI)

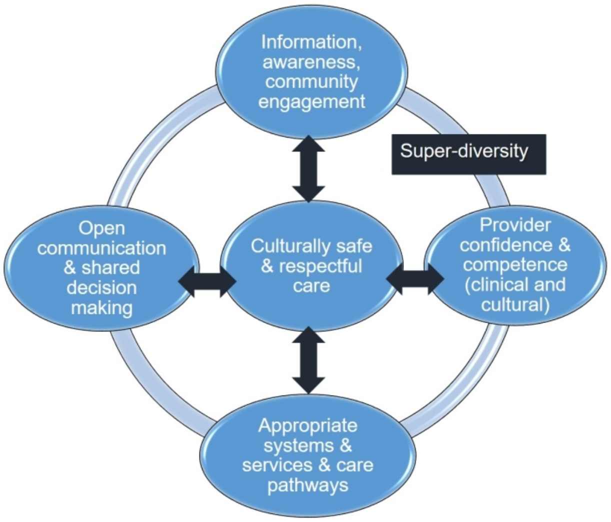

Figure 2 : Culturally safe care for FGM/C

329x174mm (150 x 150 DPI)

Supplementary File 1: Medline Search Strategy

Ovid Medline In-Process & Other Non-indexed Citations and Ovid Medline 1946 to present – run on 04/08/2017

Search statement	Search terms	Hits	Notes on strategy
1	exp Circumcision, Female/ or (exp Genitalia, Female/ and exp Medicine, Traditional/)	1367	
2	("female genital mutilation" or "female circumcision" or "female genital cutting" or "traditional female genital surgery" or infibulat* or reinfibulat* or defibulat* or clitoridect*).mp.	1509	
3	((((ritual* or traditional* or ceremon* or sociali#ation*) adj3 (practic* or cut or cutting or surg*)) or circumcis* or excis*) adj3 (female* or wom#n or girl* or child* or adolescen*)).mp.	2924	
4	(FGM or FGC or TFGS or FGMC or "FGM/C").mp.	1133	
5	(thara or tahar or khitan or khifad or khafad or megrez or absum or mekhnishab or kutairi or ibi or ugwu or sunna or bondo or sonde or gudiniin or halalays or qodiin or tahoor or bagne or gadja or ganza or "fanadu di mindjer" or niaka or kuyango or "musolula karoola").mp. not (thara or tahar or khitan or khifad or khafad or megrez or absum or mekhnishab or kutairi or ibi or ugwu or sunna or bondo or sonde or gudiniin or halalays or qodiin or tahoor or bagne or gadja or ganza or "fanadu di mindjer" or niaka or kuyango or "musolula karoola").au,in.	1650	Expanded terms to include country of origin terminology as described in Department of Health guidance.[121]
6	4 or 5	2779	
7	limit 6 to female	1200	In order to exclude irrelevant records from terminology from other disciplines, or male circumcision related hits
8	((FGM or FGC or TFGS or FGMC or "FGM/C" or thara or tahar or khitan or khifad or khafad or megrez or absum or mekhnishab or kutairi or ibi or ugwu or sunna or bondo or	507	Adjacency to “female” terms to complement

For peer review only

Search statement	Search terms	Hits	Notes on strategy
	sonde or gudiniin or halalays or qodiin or tahoor or bagne or gadja or ganza or "fanadu di mindjer" or niaka or kuyango or "musolula karoola") adj3 (female* or wom#n or girl* or child* or adolescen*)).mp.		"limit to female" hit set
9	(egypt* or sudan* or ethiopi* or somali* or djibouti* or eritrea* or burkin* or guinea* or sierra leone or leonean* or mauritan* or mali or malian*).ti,ab.	151913	Country of origin used as proxy for FGM/C terms – as FGM/C is so common, inference is that women from these countries will fit an FGM/C profile even without the term being explicitly mentioned
10	limit 9 to female	46657	
11	((egypt* or sudan* or ethiopi* or somali* or djibouti* or eritrea* or burkin* or guinea* or sierra leone or leonean* or mauritan* or mali or malian*) adj3 (female* or wom#n or girl* or child* or adolescen*)).mp.	5441	
12	1 or 2 or 3 or 7 or 8 or 10 or 11	51901	All women with FGM/C
13	"Emigrants and Immigrants"/ or Refugees/ or "Transients and Migrants"/ or "Emigration and Immigration"/	45756	
14	(immigrant* or immigrat* or emigrant* or emigrat* or emigre* or expat* or (ex adj pat*) or transients or newcomer* or (new adj comer*) or alien* or incomer* or (in adj comer*)).mp.	79630	
15	((immigrant* or immigrat* or emigrant* or emigrat* or emigre* or expat* or (ex adj pat*) or transients or newcomer* or (new adj comer*) or alien* or incomer* or (in adj comer*)) adj3 (female* or wom#n or girl* or child* or adolescen*)).mp.	4382	
16	(refugee* or (asylum adj seek*) or asylee* or (refused adj3 (asylum* or refugee*)) or (displaced adj person*) or exile* or (new adj	17539	

For peer review only

Search statement	Search terms	Hits	Notes on strategy
	arrival) or (country adj2 (birth or origin)) or transnational*).mp.		
17	((refugee* or (asylum adj seek*) or asylee* or (refused adj3 (asylum* or refugee*)) or (displaced adj person*) or exile* or (new adj arrival) or (country adj2 (birth or origin)) or transnational*) adj3 (female* or wom#n or girl* or child* or adolescen*)).mp.	1462	
18	(foreigner* or (foreign adj (born or citizen* or national* or origin*)) or (non adj (citizen* or native*)) or ((adoptive or naturali#ed) adj (citizen* or resident*)) or overstay* or trafficked or "spousal migrant*").mp	11442	
19	((foreigner* or (foreign adj (born or citizen* or national* or origin*)) or (non adj (citizen* or native*)) or ((adoptive or naturali#ed) adj (citizen* or resident*)) or overstay* or trafficked or "spousal migrant*") adj3 (female* or wom#n or girl* or child* or adolescen*)).mp.	753	
20	(((established or long-term or "first generation*" or new* or recent* or current*) adj3 (migrant* or migrat* or immigrant* or immigrat* or emigrant* or emigrat* or emigre* or expat* or (ex adj pat*) or transient* or alien*)) or newcomer* or (new adj comer*) or incomer* or (in adj comer*) or ((international or overseas or foreign) adj2 (student* or employee* or worker*))).mp.	18239	
21	((((established or long-term or "first generation*" or new* or recent* or current*) adj3 (migrant* or migrat* or immigrant* or immigrat* or emigrant* or emigrat* or emigre* or expat* or (ex adj pat*) or transient* or alien*)) or newcomer* or (new adj comer*) or incomer* or (in adj comer*) or ((international or overseas or foreign) adj2 (student* or employee* or worker*))) adj3 (female* or wom#n or girl* or child* or adolescen*)).mp.	559	
22	("non-UK-born" or "born outside the UK" or "length of residence in the UK" or (("not lawful*" or "not legal*" or unlawful* or illegal* or unauthori#ed* or "not authori#ed" or uncertain or insecure or legal* or legitimate* or permit* or visa* or irregular* or refused or undocumented) adj3 (residen* or student* or worker* or employee* or	2887	

For peer review only

Search statement	Search terms	Hits	Notes on strategy
	unemployed or immigrant* or imigrat* or migrant* or migrat*))).mp.		
23	(("non-UK-born" or "born outside the UK" or "length of residence in the UK" or (("not lawful*" or "not legal*" or unlawful* or illegal* or unauthori#ed* or "not authori#ed" or uncertain or insecure or legal* or legitimate* or permit* or visa* or irregular* or refused or undocumented) adj3 (residen* or student* or worker* or employee* or unemployed or immigrant* or imigrat* or migrant* or migrat*))) adj3 (female* or wom#n or girl* or child* or adolescen*)).mp.	136	
24	exp Vulnerable Populations/	8275	
25	((vulnerab* or disadvantag* or minorit*) adj3 (individ* or person* or people* or population* or communit* or group*)).ti,ab.	33112	
26	((vulnerab* or disadvantag* or minorit*) adj3 (individ* or person* or people* or population* or communit* or group*) adj3 (female* or wom#n or girl* or child* or adolescen*)).mp.	1756	
27	exp Ethnic Groups/	139042	
28	(ethnic* or ethno* or race or racial*).mp.	248038	
29	("Black and Minority Ethnic" or "Black & Minority ethnic" or BME or BAME or black african*).mp.	3756	
30	((ethnic* or ethno* or race or racial* or "Black and Minority Ethnic" or "Black & Minority ethnic" or BME or BAME or black african*) adj3 (female* or wom#n or girl* or child* or adolescen*)).mp.	9504	
31	exp african continental ancestry group/ or exp asian continental ancestry group/	136483	
32	(africa* or "middle east*" or asia* or iran* or iraq* or israel* or oman* or "united arab emirat*" or UAE or "saudi arabia*" or palestin* or india* or indonesia* or malaysia* or pakistan*).ti,ab.	520081	
33	(egypt* or yemen* or sudan* or ethiopi* or somali* or djibouti* or eritrea* or kenya* or uganda* or tanzania* or camero?n* or chad* or niger* or benin* or togo* or ghana* or burkin* or ivory coast* or cote d'ivoire or liberia* or guinea* or sierra leone or leonean*	259558	

For peer review only

Search statement	Search terms	Hits	Notes on strategy
	or gambia* or senegal* or mauritan* or mali or malian* or "sara subgroup" or ngama).ti,ab.		
34	((africa* or "middle east*" or asia* or iran* or iraq* or israel* or oman* or "united arab emirat*" or UAE or "saudi arabia*" or palestin* or india* or indonesia* or malaysia* or pakistan* or egypt* or yemen* or sudan* or ethiopi* or somali* or djibouti* or eritrea* or kenya* or uganda* or tanzania* or camero?n* or chad* or niger* or benin* or togo* or ghana* or burkin* or ivory coast* or cote d'ivoire or liberia* or guinea* or sierra leone or leonean* or gambia* or senegal* or mauritan* or mali or malian* or "sara subgroup" or ngama) adj3 (female* or wom#n or girl* or child* or adolescen*)).mp.	58342	
35	13 or 14 or 16 or 18 or 20 or 22 or 24 or 25 or 27 or 28 or 29 or 31 or 32 or 33	1117829	
36	limit 35 to female	491179	Keywords for immigrant populations limited to female (Medline standard limit feature)
37	(13 or 24 or 27 or 31) and (female* or wom#n or girl* or child* or adolescen*).mp.	177101	MeSH Headings for immigrant populations limited by “female” keywords
38	15 or 17 or 19 or 21 or 23 or 26 or 30 or 34 or 36 or 37	526145	Terms for immigrant populations, including specific terms for countries of origin where FGM known to be practised, limited to female
39	exp Community Health Workers/ or exp Volunteers/	23517	All health and allied workers (who may come into contact with women with FGM)
40	exp Occupational Groups/	531564	
41	exp Health Personnel/	454091	
42	exp Midwifery/	17848	

For peer review only

Search statement	Search terms	Hits	Notes on strategy
43	exp General Practitioners/ or exp Physicians/	117502	
44	exp Nurses/ or exp Nurses, Community Health/	81947	
45	exp Nurses' Aides/ or exp Nursing Staff, Hospital/ or exp Nursing Staff/	66494	
46	exp Social Workers/ or exp Police/	4605	
47	39 or 40 or 41 or 42 or 43 or 44 or 45 or 46	562187	
48	exp Pregnancy/ or (pregnan* or birth* or childbirth* or matern* or gyn#e* or obstetric* or menstru* or labo#r* or vulv* or vagina* or uter*).ti,ab.	1391519	Physical complications or disorders as identified as health impacts of FGM in Department of Health guidance,[121] already "female" by context of terms – so proxy for FGM.
49	exp Pregnancy Complications/ or exp Pregnancy High Risk/	401801	
50	((pregnan* or birth* or childbirth* or matern* or gyn#e* or obstetric* or menstru* or labo#r* or vulv* or vagina* or uter*) adj3 (poor* or adverse or complicat* or difficult* or disorder* or dysfunction* or disease* or pain* or risk* or danger* or problem* or issue* or concern* or infect* or inflamm*)).ti,ab.	137220	
51	exp Obstetric Labor Complications/	63618	
52	exp Female Urogenital Diseases/	1161123	
53	exp Menstruation Disturbances/	27153	
54	exp Genital Diseases, Female/ or exp Vaginal Diseases/ or exp Vulvar Diseases/	432325	
55	exp Vaginismus/ or exp Vulvodynia/ or exp Dyspareunia/ or exp Vaginitis/ or exp Vulvovaginitis/	14171	
56	exp Pelvic Inflammatory Disease/	10694	
57	exp Vaginal Fistula/ or exp Rectovaginal Fistula/	4659	
58	exp Genital Diseases, Female/	432325	
59	exp Infertility, Female/	27170	
60	((pelvic* or back* or urolog* or urogenit* or urinat* or genit* or abdomin*) adj3 (poor* or adverse or complicat* or difficult* or	29027	

For peer review only

Search statement	Search terms	Hits	Notes on strategy
	disorder* or dysfunction* or disease* or pain* or risk* or danger* or problem* or issue* or concern* or infect* or inflamm*) adj3 (female* or wom#n or girl* or child* or adolescen*)).mp.		
61	exp Urologic Diseases/	698515	Physical complications or disorders as identified as health impacts of FGM in Department of Health guidance,[121] limited to "female" (female genital diseases, etc.) – so proxy for FGM
62	exp Pelvic Pain/	8163	
63	exp Sexual Dysfunctions, Psychological/	24271	
64	exp Pelvic Infection/	5409	
65	exp Kidney Diseases/	474329	
66	exp Urinary Fistula/ or exp Rectal Fistula/ or exp Urinary Bladder Fistula/	11750	
67	61 or 62 or 63 or 64 or 65 or 66	737520	
68	limit 67 to female	352622	
69	67 and (female* or wom#n or girl* or child* or adolescen*).mp.	397916	
70	48 or 49 or 50 or 51 or 52 or 53 or 54 or 55 or 56 or 57 or 58 or 59 or 60 or 68 or 69	2386342	
71	exp Mental Disorders/	1122844	
72	exp Depressive Disorder, Major/ or exp Suicide/ or exp Depression/ or exp Suicidal Ideation/ or exp Depressive Disorder/ or exp Bipolar Disorder/ or exp Suicide, Attempted/	259964	
73	exp Self-Injurious Behavior/ or exp Substance-Related Disorders/ or exp Social Isolation/	329005	
74	exp Sexually Transmitted Diseases, Viral/ or exp Sexually Transmitted Diseases/	319070	
75	(anxiet* or anxious* or depress* or self-harm* or self-injur* or suicid*).mp.	650968	
76	((anxiet* or anxious* or depress* or self-harm* or self-injur* or suicid*) adj3 (female* or wom#n or girl* or child* or adolescen*)).mp.	30927	
77	(((sex* or viral* or virus* or bacteria*) adj3 diseas*) or HIV or STD* or AIDS).mp.	516543	
78	((((sex* or viral* or virus* or bacteria*) adj3 diseas*) or HIV or STD* or AIDS) adj3	29597	

For peer review only

Search statement	Search terms	Hits	Notes on strategy
	(female* or wom#n or girl* or child* or adolescen*)).mp.		
79	71 or 72 or 73 or 74 or 75 or 77	2107493	
80	limit 79 to female	932164	
81	79 and (female* or wom#n or girl* or child* or adolescen*).mp.	1098666	
82	76 or 78 or 80 or 81	1098666	Psychological or STD related impacts of FGM, as above
83	exp United Kingdom/ or ("united kingdom" or "great britain" or britain or england or scotland or wales or "northern ireland" or british or english or scottish or welsh or "northern irish" or hebrid* or "isle of man").ti,ab,gc,ia,pl,in.	1167455	
84	(AUSTRALIA* or AUSTRIA* or BELGIUM* or belgian* or CANAD* or CHILE* or CZECH* or DENMARK* or danish* or ESTONIA* or FINLAND or finnish or FRANCE or french).mp.	641227	
85	(GERMAN* or GREECE or greek* or HUNGAR* or ICELAND* or IRELAND or irish or ISRAEL* or ITALY or italian* or JAPAN* or KOREA* or LATVIA* or LUXEMB* or MEXIC*).mp.	791022	
86	(NETHERLAND* or holland* or dutch or "low countries" or europe* or "NEW ZEALAND*" or NORWAY or norwegian* or POLAND or polish or PORTUGAL or portuguese).mp.	597994	
87	(SLOVAK* or SLOVENIA* or SPAIN or spanish or SWED* or SWITZERLAND or swiss or TURK* or "UNITED KINGDOM" or "UK" or britain or british or england or english or scot* or wales or welsh or UNITED STATES or "USA" or america*).mp.	3770430	
88	83 or 84 or 85 or 86 or 87	5147200	UK or other OECD countries
89	randomized controlled trial.pt.	470445	
90	controlled clinical trial.pt.	94472	
91	randomized.ab.	403685	

For peer review only

Search statement	Search terms	Hits	Notes on strategy
92	placebo.ab.	189129	
93	clinical trials as topic.sh.	187621	
94	randomly.ab.	280498	
95	trial.ti.	181309	
96	(animals not (humans and animals)).sh.	4412254	
97	89 or 90 or 91 or 92 or 93 or 94 or 95	1138847	
98	97 not 96	1049209	Standard search hedge for randomised clinical trials in Medline – adapted from SIGN.[315]
99	38 and 47 and (70 or 82)	7952	Female immigrants AND Health Workers AND (physical or mental disorders)
100	47 and 12	1144	
101	12 or 99 or 100	59304	Women with FGM or (Female immigrants AND Health Workers AND (physical or mental disorders)) or (Health workers AND FGM)
102	101 and 88	12154	…AND OECD countries
103	102 not 98	11547	EXCLUDE RCTs
104	103 not 96	9261	EXCLUDE animal studies

For peer review only

Supplementary File 2: Excluded Studies (with Reasons)

No.	Study	Reason for Exclusion
1.	Abdel Halim AM. Honorable daughters: The lived experience of circumcised Sudanese women in the United States. PhD Thesis, Ann Arbor: Ohio University, 2003	Data presented on FGM/C, but no specific focus on FGM/C related healthcare
2.	Abdel Magied A and Shareef S. Knowledge, perception and attitudes of a sector of female health providers towards FGM - case study: female doctors. *Ahfad Journal*. 2003; 20: 4-17.	Not OECD
3.	Abdulcadir O, Catania L and Caselli A. Female genital mutilation (FGM): a comparison between the male and the female view. *Int J Gynaecol Obstet*. 2012; 119: S262-S3.	Conference abstract
4.	Alizadeh V, Hylander I, Kocturk T and Tornkvist L. Counselling young immigrant women worried about problems related to the protection of "family honour"- From the perspective of midwives and counsellors at youth health clinics. *Scand J Caring Sci*. 2010; 24: 32-40	Data presented on FGM/C, but no specific focus on FGM/C related healthcare
5.	Alizadeh V, Tornkvist L and Hylander I. Counselling teenage girls on problems related to the 'protection of family honour' from the perspective of school nurses and counsellors. *Health Soc Care Community*. 2011; 19: 476-84.	Data on FGM/C not presented
6.	Allag F, Abboud P, Mansour G, Zanardi M and Quereux C. Female genital mutilation. Women's point of view. *Gynecologie Obstetrique Fertilite*. 2001; 29: 824-8.	Data presented on FGM/C, but no specific focus on FGM/C related healthcare
7.	Aquino M, Edge D and Smith DM. Pregnancy as an ideal time for intervention to address the complex needs of black and minority ethnic women: Views of British midwives. *Midwifery*. 2015; 31: 373-9.	Data on FGM/C not presented
8.	Ariyo D, Ssali R, King-Webb L and Ikpaahindi S. Voices of the community: exploring female genital mutilation in the African community across Greater Manchester. London: AFRUCA, 2015.	Data presented on FGM/C, but no specific focus on FGM/C related healthcare
9.	Beck-Karrer C, Levin T and Levin T. Lion women. Conversations with Somalian women and men about female circumcision. *European Journal of Women's Studies*. 1998; 5: 533-6.	Not empirical research
10.	Bergqvist, H. & Svensson, J. (2016) [Midwives Experiences of Encounters wth Young Women who come from Areas Where the Practice of Genital Mutilation is Common] Barnmorskors Erfarenheter Av Möten Med Unga Kvinnor Som Kommer Från Områden Där Kvinnlig Könsstympning är Aanligt Förekommande. University of Skövde, MSc Thesis, Sweden.	Data on healthcare-specific experiences is only from health professionals, not women/girls

For peer review only

No.	Study	Reason for Exclusion
11.	Bibi, N. & Rahimian, N. (2013) [Nurses' Experience and Knowledge about Female Genital Mutilation] Sjuksköterskans Erfarenheter Och Kunskaper Om Kvinnlig Könssympning. Sophiahemmet University, MSc Thesis, Sweden.	Data on healthcare-specific experiences is only from health professionals, not women/girls
12.	Brodin, E. & Mårtensson, N. (2016) [District Nurses' Knowledge and Experience of Female Genital Mutilation] Distriktssköterskors Kunskap Och Erfarenhet Av Kvinnlig Könsstympning. University of Örebro, MSc Thesis, Sweden.	Data on healthcare-specific experiences is only from health professionals, not women/girls
13.	Brown E, Carroll J, Fogarty C and Holt C. "They get a C-section . . . they gonna die": Somali women's fears of obstetrical interventions in the United States. *J Transcult Nurs*. 2010; 21: 220-7.	Data on FGM/C not presented
14.	Brown E and Porter C. The tackling FGM initiative: evaluation of the second phase (2013-2016). Options UK, Report. 2016.	Not empirical research
15.	Buckland RL. The everyday experience of Somali women in Canada: Implications for health. MSc Thesis, University of Ottawa (Canada), 1997	Data presented on FGM/C, but no specific focus on FGM/C related healthcare
16.	Bulman, K. & McCourt, C. (1997) Report on Somali Womens' Experiences of Maternity Services. Centre for Midwifery Practice, Wolfson Institute of Health Sciences, Thames Valley University and Hammersmith Hospitals National Health Service Trust, London, UK.	Data on healthcare-specific experiences is only from health professionals, not women/girls
17.	Burchill J. Safeguarding vulnerable families: work with refugees and asylum seekers. *Community Practitioner*. 2011; 84: 23-6.	Data presented on FGM/C, but no specific focus on FGM/C related healthcare
18.	Burchill, J. & Pevalin, D.J. (2014) Demonstrating cultural competence within health-visiting practice: working with refugee and asylum-seeking families. *Divers Equal Health Care,* 11(2), 151-159.	Data on healthcare-specific experiences is only from health professionals, not women/girls
19.	Byrskog, U., Olsson, P., Essen, B. & Allvin, M.K. (2015) Being a bridge: Swedish antenatal care midwives' encounters with Somali-born women and questions of violence - a qualitative study. *BMC Pregnancy Childbirth,* 15(1).	Data on healthcare-specific experiences is only from health professionals, not women/girls
20.	Carolan M. Antenatal care perceptions of pregnant African women attending maternity services in Melbourne, Australia. *Midwifery*. 2010; 26: 189-201.	Data on FGM/C not presented
21.	Chalmers B and Hashi KO. 432 Somali women's birth experiences in Canada after earlier female genital mutilation. *Birth*. 2000; 27: 227-34.	Not qualitative research
22.	Christiansen CD. The lived experience of circumcision in immigrant Somali women: a Heideggerian hermeneutic analysis. MA Thesis, University of Illinois, 1995,	Data presented on FGM/C, but no specific focus on FGM/C related healthcare

For peer review only

No.	Study	Reason for Exclusion
23.	Clarke ALF. The process of changed meanings: A study of the Canadian experience of Somali women in the Kitchener-Waterloo area in regard to female circumcision. PhD Thesis, Ann Arbor: Wilfrid Laurier University (Canada), 1995,	Not empirical research
24.	Clayton-Hathway K. A Pilot Evaluation of Health Services for Communities Affected by FGM in Oxfordshire: Final Report. Oxford, UK: Healthwatch Oxfordshire, 2016.	Not qualitative research
25.	Connor JJ, Hunt S, Finsaas M, Ciesinski A, Ahmed A and Robinson BBE. Sexual Health Care, Sexual Behaviors and Functioning, and Female Genital Cutting: Perspectives From Somali Women Living in the United States. *J Sex Res*. 2016; 53: 346-59.	Data presented on FGM/C, but no specific focus on FGM/C related healthcare
26.	Dahlen UM. Female Genital Cutting: Phenomenological Interviews on the Ethiopian Immigrant Mothers' Experience. Masters Thesis, Regent University, 2011,	Data presented on FGM/C, but no specific focus on FGM/C related healthcare
27.	Dawson, A.J., Turkmani, S., Varol, N., Nanayakkara, S., Sullivan, E. & Homer, C.S. (2015) Midwives' experiences of caring for women with female genital mutilation: insights and ways forward for practice in Australia. *Women Birth,* 28(3), 207-14.	Data on healthcare-specific experiences is only from health professionals, not women/girls
28.	Degni F, Suominen S, Essen B, El Ansari W and Vehvilainen-Julkunen K. Communication and cultural issues in providing reproductive health care to immigrant women: health care providers' experiences in meeting the needs of Somali women living in Finland. *Journal of Immigrant and Minority Health*. 2012; 14: 330-43.	Data on FGM/C not presented
29.	Essen B, Binder P and Johnsdotter S. An anthropological analysis of the perspectives of Somali women in the West and their obstetric care providers on caesarean birth. *Journal of Psychosomatic Obstetrics and Gynecology*. 2011; 32: 10-8.	Data on FGM/C not presented
30.	Essen B, Johnsdotter S and Binder P. Not too far to walk but too far for reciprocity: Maternal mortality in a migration context using the 'three delays' framework. *International Journal of Gynecology and Obstetrics*. 2012; 119: S343.	Conference abstract
31.	Feldman R. When maternity doesn't matter: Dispersing pregnant women seeking asylum. *British Journal of Midwifery*. 2014; 22: 23-8.	Data on FGM/C not presented
32.	Finnstrom B and Soderhamn O. Conceptions of pain among Somali women. *J Adv Nurs*. 2006; 54: 418-25.	Data presented on FGM/C, but no specific focus on FGM/C related healthcare
33.	García Aguado S and Sánchez López MI. Knowledge of healthcare professionals about female genital mutilation. *Metas de Enfermería*. 2013; 16: 18-22.	Not qualitative research
34.	Gele AA, Kumar B, Hjelde KH and Sundby J. Attitudes toward female circumcision among Somali immigrants	Data presented on FGM/C, but no specific

For peer review only

No.	Study	Reason for Exclusion
	in Oslo: a qualitative study. *International Journal of Women's Health*. 2012; 4: 7-17.	focus on FGM/C related healthcare
35.	Gele AA, Sagbakken M and Kumar B. Is female circumcision evolving or dissolving in Norway? A qualitative study on attitudes toward the practice among young Somalis in the Oslo area. *International Journal of Women's Health*. 2015; 7: 933-43.	Data presented on FGM/C, but no specific focus on FGM/C related healthcare
36.	Gerrish K, Naisby A and Ismail M. Experiences of the diagnosis and management of tuberculosis: A focused ethnography of Somali patients and healthcare professionals in the UK. *J Adv Nurs*. 2013; 69: 2285-94.	Data on FGM/C not presented
37.	Gertsson, M. & Serpan, H. (2009) [Meeting with the Unthinkable: Value Conflicts in Meetings with Women Vulnerable to Female Genital Mutilation] Mötet Med Det Otänkbara: Värdekonflikter I Mötet Med Kvinnor Utsatta för Kvinnlig Könsstympning. University of Kalmar, MSc Thesis, Sweden.	Data on healthcare-specific experiences is only from health professionals, not women/girls
38.	Glavin K and Sæteren B. Cultural Diversity in Perinatal Care: Somali New Mothers' Experiences with Health Care in Norway. *Health Science Journal*. 2016; 10: 1-9.	Data on FGM/C not presented
39.	Glazer E. Gendering chronic pain: Socio-cultural embodiment and circumcision in Somali women in Toronto. *Gend Med*. 2010; 7: 528.	Conference abstract
40.	Goldblatt H. Caring for abused women: Impact on nurses' professional and personal life experiences. *J Adv Nurs*. 2009; 65: 1645-54.	Data on FGM/C not presented
41.	Hai DNT and Ghebre R. Comparison of barriers to cervical cancer screening among Somali and Vietnamese women. *J Womens Health*. 2013; 22: 36.	Conference abstract
42.	Henderson A. Factors influencing nurses' responses to abused women: What they say they do and why they say they do it. *Journal of Interpersonal Violence*. 2001; 16: 1284-306.	Data on FGM/C not presented
43.	Herrel N, Olevitch L, DuBois DK, et al. Somali refugee women speak out about their needs for care during pregnancy and delivery. *Journal of Midwifery & Women's Health*. 2004; 49: 345-9.	Data on FGM/C not presented
44.	Holm, L. & Kammensjö, H. (2012) [School Nurses' Experiences of Female Genital Mutilation among Girls] Skolsköterskors Upplevelser Kring Kvinnlig Könsstympning Bland Flickor. University of Skövde, MSc Thesis, Sweden.	Data on healthcare-specific experiences is only from health professionals, not women/girls
45.	Houston AR. (In)visible embodiment: Somali perspectives of diabetes and mental health in diaspora. MSc Thesis, Boston University, 2016,	Data presented on FGM/C, but no specific focus on FGM/C related healthcare
46.	Hoyt MA. In her own words: An exploration of immigrant women's experiences of reproductive health	Data on FGM/C not presented

For peer review only

No.	Study	Reason for Exclusion
	care through ethnographic narrative. MA Thesis, University of New Brunswick (Canada), 2010,	
47.	Isman E, Ekeus C and Berggren V. Perceptions and experiences of female genital mutilation after immigration to Sweden: an explorative study. *Sexual and Reproductive Healthcare*. 2013; 4: 93-8.	Data presented on FGM/C, but no specific focus on FGM/C related healthcare
48.	Jacoby SD, Lucarelli M, Musse F, Krishnamurthy A and Salyers V. A Mixed-Methods Study of Immigrant Somali Women's Health Literacy and Perinatal Experiences in Maine. *Journal of Midwifery and Women's Health*. 2015; 60: 593-603.	Data presented on FGM/C, but no specific focus on FGM/C related healthcare
49.	Jatau, M. (2011) Living Between Two Cultures: A Reproductive Health Journey of African Refugee Women. Arizona State University, PhD Thesis, USA.	Data on healthcare-specific experiences is only from health professionals, not women/girls
50.	Johansen REB. Experiencing sex in exile: can genitals change their gender? On conceptions and experiences related to female genital cutting (FGC) among Somalis in Norway. In: *Transcultural Bodies: Female Genital Cutting In Global Context Ed Ylva Hernlund & Bettina Shell-Duncan*. Rutgers University Press, 2007, p. 248-77.	Data presented on FGM/C, but no specific focus on FGM/C related healthcare
51.	Johansen, R.E. (2006) Care for infibulated women giving birth in Norway: an anthropological analysis of health workers' management of a medically and culturally unfamiliar issue. *Med. Anthropol. Q.*, 20(4), 516-44.	Data on healthcare-specific experiences is only from health professionals, not women/girls
52.	Johansen, R.E. (2017) Virility, pleasure and female genital mutilation/cutting: a qualitative study of perceptions and experiences of medicalized defibulation among Somali and Sudanese migrants in Norway. *Reprod Health,* 14(1), 25.	Data on healthcare-specific experiences is only from health professionals, not women/girls
53.	Johnsdotter S, Ingvarsdotter K, Ostman M and Carlbom A. Koran reading and negotiation with jinn: strategies to deal with mental ill health among Swedish Somalis. *Mental Health, Religion & Culture*. 2011; 14: 741-55.	Data on FGM/C not presented
54.	Johnsdotter S and Essén B. It is only a tradition: making sense of Swedish Somalis' narratives of female circumcision and avoiding submission to hegemonic political discourse. *Crit Soc Policy*. 2005; 25: 577-89.	Not empirical research
55.	Johnsdotter S and Essen B. Cultural change after migration: Circumcision of girls in Western migrant communities. *Best Practice and Research: Clinical Obstetrics and Gynaecology*. 2016; 32: 15-25.	Not empirical research
56.	Johnsdotter S, Moussa K, Carlbom A, Aregai R and Essen B. "Never my daughters": A qualitative study	Data presented on FGM/C, but no specific

For peer review only

No.	Study	Reason for Exclusion
	regarding attitude change toward female genital cutting among Ethiopian and Eritrean families in Sweden. *Health Care Women Int*. 2009; 30: 114-33.	focus on FGM/C related healthcare
57.	Johnson-Agbakwu CE, Helm T, Killawi A and Padela AI. Perceptions of obstetrical interventions and female genital cutting: insights of men in a Somali refugee community. *Ethn Health*. 2014; 19: 440-5	Focus on men, not women
58.	Kallon I and Dundes L. The cultural context of the Sierra Leonean Mende woman as patient. *J Transcult Nurs*. 2010; 21: 228-36.	Not empirical research
59.	Kaplan-Marcusan A, Del Rio NF, Moreno-Navarro J, et al. Female genital mutilation: perceptions of healthcare professionals and the perspective of the migrant families. *BMC Public Health*. 2010; 10: 193.	Not qualitative research
60.	Kay M, Wijayanayaka S, Cook H and Hollingworth S. Understanding quality use of medicines in refugee communities in Australian primary care: A qualitative study. *Br J Gen Pract*. 2016; 66: e397-e409.	Data on FGM/C not presented
61.	Koukoui S, Hassan G and Guzder J. The mothering experience of women with FGM/C raising 'uncut' daughters, in Ivory Coast and in Canada. *Reproductive Health*. 2017; 14: 1-11.	Data presented on FGM/C, but no specific focus on FGM/C related healthcare
62.	Lazar J, Shipp M and Johnson C. Provider perceptions of sexual desire and dyspareunia among Somali women with female genital cutting. *J Sex Med*. 2010; 7: 148.	Conference abstract
63.	Lazar, J.N., Johnson-Agbakwu, C.E., Davis, O.I. & Shipp, M.P.L. (2013) Providers' perceptions of challenges in obstetrical care for Somali women. *Obstet. Gynecol. Int.,* 2013, 149640.	Data on healthcare-specific experiences is only from health professionals, not women/girls
64.	Lane J and Cole G. Pregnancy, prenatal care and delivery perceptions and beliefs of resettled African refugee women in the western United States. *Contraception*. 2013; 88: 313.	Conference abstract
65.	Leishman J. Perspectives of cultural competence in health care. *Nurs Stand*. 2004; 19: 33	Data on FGM/C not presented
66.	León-Larios, F. & Casado-Mejía, R. (2012) [Influence of gender on knowledge, perception and approach to harmful traditional practices: female genital mutilation]. *Evidentia,* 9(40), 1-7.	Data on healthcare-specific experiences is only from health professionals, not women/girls
67.	Lephard E and Haith-Cooper M. Pregnant and seeking asylum: Exploring women's experiences 'from booking to baby'. *British Journal of Midwifery*. 2016; 24: 130-6.	Data on FGM/C not presented
68.	Leval, A., Widmark, C., Tishelman, C. & Maina Ahlberg, B. (2004) The encounters that rupture the myth: contradictions in midwives' descriptions and explanations of circumcised women immigrants' sexuality. *Health Care Women Int.,* 25(8), 743-60.	Data on healthcare-specific experiences is only from health professionals, not women/girls

For peer review only

No.	Study	Reason for Exclusion
69.	Lewig K, Arney F and Salveron M. Challenges to parenting in a new culture: Implications for child and family welfare. *Eval Program Plann*. 2010; 33: 324-32.	Data on FGM/C not presented
70.	Leye E. Midwifery training needs identified when caring for women with female genital mutilation. *Evid Based Nurs*. 2016; 19: 7.	Not empirical research
71.	Liao LM, Elliott C, Ahmed F and Creighton SM. Adult recall of childhood female genital cutting and perceptions of its effects: a pilot study for service improvement and research feasibility. *J Obstet Gynaecol*. 2013; 33: 292-5.	Not qualitative research
72.	Lien IL and Schultz JH. Internalizing knowledge and changing attitudes to female genital cutting/mutilation. *Obstet Gynecol Int*. 2013; 2013: 467028.	Data presented on FGM/C, but no specific focus on FGM/C related healthcare
73.	McCarthy R and Haith-Cooper M. Evaluating the impact of befriending for pregnant asylum-seeking and refugee women. *British Journal of Midwifery*. 2013; 21: 404-9.	Data on FGM/C not presented
74.	McLeish J. Maternity experiences of asylum seekers in England. *British Journal of Midwifery* 2005; 13: 782-5.	Data on FGM/C not presented
75.	Mitello L and Proietti A. Feminine genitals modification: survey of a new phenomenon for nurse today. Second Part. *Prof Inferm*. 2006; 59: 242-5.	Not qualitative research
76.	Mitello L and Proietti A. Female genital modification: survey of new phenomenon for nurses today. First Part. *Prof Inferm*. 2006; 59: 171-81.	Not empirical research
77.	Moore, K. (2012) Female Genital Mutilation and Cultural Competency: Moving Towards Improved Management of Obstetric Care. Queen Margaret University, MSc Thesis, Edinburgh.	Data on healthcare-specific experiences is only from health professionals, not women/girls
78.	Morris MD, Popper ST, Rodwell TC, Brodine SK and Brouwer KC. Healthcare barriers of refugees post-resettlement. *J Community Health*. 2009; 34: 529-38.	Data on FGM/C not presented
79.	Morison L and others. How experiences and attitudes relating to female circumcision vary according to age on arrival in Britain: a study among young Somalis in London. *Ethnicity & Health*. 2004; 9: 75-100.	Data presented on FGM/C, but no specific focus on FGM/C related healthcare
80.	Mwangi-Powell F. Female genital mutilation: a case study in Birmingham. FORWARD, Report, 2000.	Data presented on FGM/C, but no specific focus on FGM/C related healthcare
81.	Nash E and Ranka P. Female genital mutilation: Knowledge, confidence, and approach to care in clinical practice of midwives and nurses in the UK. *International Journal of Gynecology and Obstetrics*. 2015; 131: E110.	Not qualitative research
82.	Nur Z. Cultural Competence in Health Care: Exploring the Experiences of Muslim Women within the Ontario	Data on FGM/C not presented

For peer review only

No.	Study	Reason for Exclusion
	Healthcare System. MSc Thesis, University of Ontario Institute of Technology (Canada), 2014,	
83.	Ogbagzy RA. The construction of Eritrean women's identity in the Canadian context. MA Thesis, University of Toronto (Canada), 1999, p. 236.	Data presented on FGM/C, but no specific focus on FGM/C related healthcare
84.	Ogunsiji O. Understanding the dilemma of de-infibulation for women living with female genital mutilation. *Australian Nursing & Midwifery Journal*. 2015; 22: 51	Not empirical research
85.	Ogunsiji O and Wilkes L. Cultural explanations and procedures about female genital mutilation. *Australian Nursing & Midwifery Journal*. 2015; 22: 51	Data presented on FGM/C, but no specific focus on FGM/C related healthcare
86.	Ogunsiji O, Wilkes L, Peters K and Jackson D. Knowledge, attitudes and usage of cancer screening among West African migrant women. *J Clin Nurs*. 2013; 22: 1026-33.	Data on FGM/C not presented
87.	Ogunsiji, O. (2015) Female genital mutilation (FGM): Australian midwives' knowledge and attitudes. *Health Care Women Int.,* 36(11), 1179-1193.	Data on healthcare-specific experiences is only from health professionals, not women/girls
88.	Ogunsiji, O. (2016) Australian midwives' perspectives on managing obstetric care of women living with female genital circumcision/mutilation. *Health Care Women Int.,* 37(10), 1156-69.	Data on healthcare-specific experiences is only from health professionals, not women/girls
89.	Olsson E, Lau M, Lifvergren S and Chakhunashvili A. Community collaboration to increase foreign-born women's participation in a cervical cancer screening program in Sweden: A quality improvement project. *International Journal for Equity in Health*. 2014; 13	Data on FGM/C not presented
90.	Pavlish, C.L., Noor, S. & Brandt, J., Somali immigrant women and the American health care system: discordant beliefs, divergent expectations, and silent worries, *Soc Sci Med.* 2010; 71:2, 353-361	Data on FGM/C not presented
91.	Peters K. Reasons why women choose a medical practice or a women's health centre for routine health screening: worker and client perspectives. *J Clin Nurs*. 2010; 19: 2557-64.	Data on FGM/C not presented
92.	Phillimore J. Migrant maternity in an era of superdiversity: new migrants' access to, and experience of, antenatal care in the West Midlands, UK. *Soc Sci Med*. 2016; 148: 152-9.	Data on FGM/C not presented
93.	Quilliam S. Female genital mutilation: three questions for us to consider. *J Fam Plann Reprod Health Care*. 2015; 41: 235-7.	Not empirical research
94.	Radford K. 'Unkind cuts': health policy and practice versus the health and emotional well-being of asylum-	Data on FGM/C not presented

For peer review only

No.	Study	Reason for Exclusion
	seekers and refugees in Ireland. *Journal of Ethnic and Migration Studies*. 2010; 36: 899-915.	
95.	Razzak M. Sexual medicine: Pain and pleasure-reconstruction after female genital mutilation. *Nature Reviews Urology*. 2012; 9.	Not empirical research
96.	Reis E. Culture and cutting. *The Hastings Center Report*. 2012; 42: 3.	Not empirical research
97.	Rimmer SN. [Theme: circumcision. Somali women talk about taboo]. *Sygeplejersken*. 1994; 94: 12-3.	Not empirical research
98.	Roberts TL and Poblete X. Female genital mutilation: A survey of awareness, training and current practice. *Archives of Disease in Childhood: Annual Conference of the Royal College of Paediatrics and Child Health, RCPCH 2015*. Birmingham, UK: BMJ Publishing Group, 2015, p. G55.	Conference abstract
99.	Rogers C and Earnest J. A Cross-Generational Study of Contraception and Reproductive Health Among Sudanese and Eritrean Women in Brisbane, Australia. *Health Care Women Int*. 2014; 35: 334-56.	Data on FGM/C not presented
100.	Rosenberg LB, Gibson K and Shulman JF. When cultures collide: Female genital cutting and U.S. obstetric practice. *Obstet Gynecol*. 2009; 113: 931-4.	Not empirical research
101.	Rosenberg E, Kirmayer LJ, Xenocostas S, Dao MD and Loignon C. GPs' strategies in intercultural clinical encounters. *Fam Pract*. 2007; 24: 145-51.	Data on FGM/C not presented
102.	Roth K. Giving refuge: reflections on working with asylum seekers. *J Pain Symptom Manage*. 2010; 40: 149-54.	Not empirical research
103.	Rubin, E.A. (2000) When Cultures Collide: An Exploration of Cultural Competence and Cross-Cultural Communication between American Medical Providers and Immigrant Women who have been Circumcised. University of Massachusetts, PhD Thesis, USA.	Data on healthcare-specific experiences is only from health professionals, not women/girls
104.	Sala R and Manara D. Nurses and requests for female genital mutilation: cultural rights versus human rights. *Nurs Ethics*. 2001; 8: 247-58.	Not empirical research
105.	Schuster S. 'Gosh': a cross-cultural encounter with a Somali woman, a male interpreter and a gynecologist on female genital cutting/mutilation. *Patient Educ Couns*. 2015; 98: 127-8.	Not empirical research
106.	Shelp A. Women helping women: the Somali doula initiative. *International Journal of Childbirth Education*. 2004; 19: 4-7.	Not qualitative research
107.	Shipp MPL, Francis SA, Fluegge KR and Asfaw SA. Perceived Health Issues: A perspective from East-African immigrants. *Health, Culture and Society*. 2014; 6: 13-32.	Data on FGM/C not presented
108.	Sigurjonsson H, Jordal M and Lundgren TK. Reconstructive surgery after female genital mutilation. *International Journal of Gynecology and Obstetrics*. 2015; 131: E318-E9.	Conference abstract

For peer review only

No.	Study	Reason for Exclusion
109.	Simpson J, Robinson K, Creighton SM and Hodes D. Female genital mutilation: the role of health professionals in prevention, assessment, and management. *Br Med J*. 2012; 344.	Not empirical research
110.	Sokolik L. Somali immigrant and refugee women's experience of pregnancy and childbirth in the American healthcare system. *Commun Nurs Res*. 2007; 40: 538	Conference abstract
111.	Sudbury H and Robinson A. Barriers to sexual and reproductive health care for refugee and asylum-seeking women. *British Journal of Midwifery*. 2016; 24: 275-81.	Not empirical research
112.	Sundby J. Genital mutilation of women--is it a concern for Gynecologists? *Acta Obstet Gynecol Scand*. 1996; 75: 513-5.	Not empirical research
113.	Talle A. From "complete" to "impaired" body: Female circumcision in Somalia and London. *Disability in Local and Global worlds*. Berkeley, CA: University of California Press; US, 2007, p. 56-77.	Not empirical research
114.	Taillens F. ["I will not circumcise my daughter"]. *Krankenpflege - Soins Infirmiers*. 2012; 105: 55.	Not empirical research
115.	Tait P. Refugee Women in Australia and Woman-Centered Midwifery Care. *Nuritinga*. 2013: 31-9.	Not empirical research
116.	Tantet C, Aupiais C, Sorge F, Levy D, Lafon-Desmurs B and Faye A. MIG-01 - Female genital mutilations: Evaluation of the knowledge of general practitioners and physicians in travel counseling. *Med Mal Infect*. 2016; 46: 77.	Not qualitative research
117.	Tatah EF. Female Circumcision: A Phenomenological Study of Somalian Immigrants to the United States. PhD Thesis, Walden University, 2016,	Data presented on FGM/C, but no specific focus on FGM/C related healthcare
118.	Trueland J. School nurses take lead on FGM. *Nurs Stand*. 2014; 28: 22-3.	Not empirical research
119.	Vercoutere A, Temmerman M and Leye E. Female genital mutilation, a story far away from home? Ethical, legal and clinical aspects of mutilated women in western countries. *Tijdschrift voor Geneeskunde*. 2011; 67: 237-41.	Not empirical research
120.	Villani M. From the “maturity” of a woman to surgery: Conditions for clitoris repair. *Sexologies*. 2009; 18: 259-61.	Not qualitative research
121.	Villani M. The way of speaking about "mutilation" and performing "repairing". *J Sex Med*. 2011; 8: 242.	Not qualitative research
122.	Villani M. Experiences with clitoris repair with counselling or counselling only in France, for effect on sexual satisfaction for women with FGM. *Int J Gynecol Obstet*. 2012; 119: S255.	Conference abstract
123.	Villani M, Griffin J and Bodenmann P. In their own words: the health and sexuality of immigrant women with infibulation living in Switzerland. *Soc Sci*. 2016; 5: 1.	Data presented on FGM/C, but no specific focus on FGM/C related healthcare

For peer review only

No.	Study	Reason for Exclusion
124.	Vissandjee B, Denetto S, Migliardi P and Proctor J. Female genital cutting (FGC) and the ethics of care: community engagement and cultural sensitivity at the interface of migration experiences. *BMC Int Health Hum Rights*. 2014; 14: 1-18.	Not empirical research
125.	Vloeberghs E, Knipscheer J, van der Kwaak A, Naleie Z and van den Muijsenbergh M. Veiled Pain: A Study In The Netherlands On The Psychological, Social And Relational Consequences Of Female Genital Mutilation. Pharos–Dutch National Knowledge and Advisory Centre on Refugees and Migrants' Health, 2010.	Duplicate report of included paper
126.	Wade L. The politics of acculturation: Female genital cutting and the challenge of building multicultural democracies. *Soc Probl*. 2011; 58: 518-37.	Data presented on FGM/C, but no specific focus on FGM/C related healthcare
127.	Widmark, C., Leval, A., Tishelman, C. & Ahlberg, B.M. (2010) Obstetric care at the intersection of science and culture: Swedish doctors' perspectives on obstetric care of women who have undergone female genital cutting. *J. Obstet. Gynaecol.,* **30**(6), 553-8.	Study focus is on health professionals, not women/girls
128.	Widmark, C., Tishelman, C. & Ahlberg, B.M. (2002) A study of Swedish midwives' encounters with infibulated African women in Sweden. *Midwifery,* 18(2), 113-25.	Data on healthcare-specific experiences is only from health professionals, not women/girls
129.	Williams KW. Somali Bantu health experience: Refugee resettlement in South Carolina. PhD Thesis, University of South Carolina, 2006	Data on FGM/C not presented
130.	Williams N, Offer GJ, Williams S and Everson N. Parental attitude in children referred for circumcision. *Br J Surg*. 1997; 84: 1722-3.	Data on FGM/C not presented
131.	Willis S. The cutting season. *Journal of Family Health Care*. 2014; 24: 9-12.	Not empirical research
132.	Wimmer-Puchinger B, Wolf H and Engleder A. [Female migrants in the health care system. Health care utilisation, access barriers and health promotion strategies]. *Migrantinnen im Gesundheitssystem Inanspruchnahme, Zugangsbarrieren und Strategien zur Gesundheitsforderung*. 2006; 49: 884-92.	Not empirical research
133.	Young H and McGrath K. A review of circumcision in New Zealand: 'I never liked doing them and I was pleased to give them up'. *Understanding Circumcision: A Multi-Disciplinary Approach To A Multi-Dimensional Problem*. Kluwer Academic Publishers, 2001.	Data on FGM/C not presented
134.	Yusuf L and Negash S. Vaginal calculus following severe form of female genital mutilation: a case report. *Ethiop Med J*. 2008; 46: 185-8.	Not OECD
135.	Zenner N, Liao LM, Richens Y and Creighton SM. Quality of obstetric and midwifery care for pregnant women who have undergone female genital mutilation. *J Obstet Gynaecol*. 2013; 33: 459-62.	Not qualitative research

For peer review only

For peer review only

Supplementary File 3: Coding and Analysis Process

Example of the Analytical Theme of 'Communication is Key'

Below, we describe the analytical process involved in the thematic synthesis, using the Analytical Theme 1 ('Communication is Key') as an exemplar.

A group of rich, relevant and high quality papers was initially coded line by line.[1-13] This approach used 'open coding' (i.e. it was data-driven - there were no pre-defined codes or themes). Two reviewers (RT &CE) undertook this task independently.

The open codes were then reviewed and compared. Where reviewers had identified and coded the same issue, a code for that issue was agreed for use in coding subsequent papers. A standardised name was identified for the code and a description was produced. Where reviewers had applied slightly different codes to a concept indicating a different interpretation of meaning, they discussed it and either agreed a shared code (based on a shared understanding of meaning) or created two different codes to reflect two different potential meanings.

Example: Straus 2009[9]
"Lack of interpreters and notably a lack of what was described as ''suitable'' ones were mentioned as a difficulty". P.183

Reviewer 1(CE) open code – interpretation problems
Reviewer 2 (RT) open code – lack of interpreters

Initial combined open code 1: Interpretation problems
Description: Challenges with using interpreters

Initial combined open code 2: Lack of interpreters
Description: Lack of access to interpreters

As can be seen above, the initial set of open codes were 'free nodes'. These initial sets of codes were regularly examined through team discussion. The codes were then expanded, merged or refined. For example, after coding additional papers, it became clear that the initial combined open code 1 ('interpretation problems') was very broad, so it was decided to code more closely to identify what specific aspects of interpretation were considered challenging. After re-reading the original papers, two refined codes were created, so that there were a total of three codes related to interpretation (all the index papers were re-coded using the refined framework).

Open code 1: Unskilled interpreters
Description: When interpreters are untrained or unable to translate the specific terminology around FGM/C

Open code 2: Male interpreters
Description: Cultural sensitivities and challenges with communication from having a male interpreter

Open code 3: Lack of interpreters

For peer review only

Description: This refers to a lack of access to interpreters or service and reliance on self. Health services not offering interpreting services and women have to find their own to access care.

As the coding progressed, the texts coded to each node were reviewed by the team and the open codes began to be grouped into themes based on similarity in meaning. For example, the three open codes above were grouped under one node entitled 'Interpretation Challenges'.

Theme (Node): Interpretation Challenges<br>Description: Ways in which challenges with interpretation affect communication about FGM/C

The team met regularly to review the codes/nodes and their descriptions (the codebook) and, through discussion, began a process of comparing and refining themes and building a thematic framework. For example, the findings from the papers coded under 'Interpretation Challenges' were compared with findings that had been coded under another theme entitled 'Language Barriers'. This process showed that 'Interpretation Challenges' were one of several consequences of 'Language Barriers', all of which affected health professional and patient communication. Hence, the theme 'Interpretation Challenges' was merged with the theme 'Language Barriers', to create a new descriptive theme called: 'Language Barriers and Interpretation Challenges'.

Further analysis made clear that whilst 'Language Barriers and Interpretation Challenges' were an important aspect of communication problems around FGM/C, there were also other challenges to communication that had been identified and coded under other descriptive themes - around cultural taboos ('Can't talk, not asked') and experiencing culturally insensitive questions ('Cultural In-Sensitivity'). Hence, in the final analysis, the meanings of the three descriptive themes, were interpreted into one analytical theme ('Communication is Key').

References

1. Baldeh F. Obstetric Care in Scotland: The Experience of Women who have Undergone Female Genital Mutilation (FGM) [MSc Thesis]. Queen Margaret University, 2013.
2. d'Entremont M, Smythe L, McAra-Couper J. The sounds of silence: a hermeneutic interpretation of childbirth post excision. *Health Care Women Int* 2014;35(3):300-19. doi: 10.1080/07399332.2013.838245
3. Fawcett L. Somali Refugee Women and their U.S. Healthcare Providers: Knowledge, Perceptions and Experiences of Childbearing [Doctor of Philosophy]. Arizona State University, 2014.
4. Glover J, Liebling H, Barrett H, et al. The psychological and social impact of female genital mutilation: a holistic conceptual framework. *J Int Stud* 2017;10(2):219-38.
5. Jones A. Working Psychologically with Female Genital Mutilation: An Exploration of the Views and Experiences of Women who have Experienced FGM and of Clinical Psychologists. University of East London, 2010.
6. Lundberg PC, Gerezgiher A. Experiences from pregnancy and childbirth related to female genital mutilation among Eritrean immigrant women in Sweden. *Midwifery* 2008;24(2):214-25. doi: 10.1016/j.midw.2006.10.003

For peer review only

7. Moxey JM, Jones LL. A qualitative study exploring how Somali women exposed to female genital mutilation experience and perceive antenatal and intrapartum care in England. *BMJ Open* 2016;6(1):e009846. doi: 10.1136/bmjopen-2015-009846
8. Palfreyman A, Brown E, Nam S. Understanding Female Genital Mutilation in Birmingham: Findings from a PEER Study: Options Consultancy Services and Birmingham & Solihull Women's Aid, 2011.
9. Straus L, McEwen A, Hussein FM. Somali women's experience of childbirth in the UK: perspectives from Somali health workers. *Midwifery* 2009;25(2):181-6. doi: 10.1016/j.midw.2007.02.002
10. Thierfelder C. Female Genital Mutilation and the Swiss Health Care System. University of Basel, 2003.
11. Vangen S, Johansen REB, Sundby J, et al. Qualitative study of perinatal care experiences among Somali women and local health care professionals in Norway. *Eur J Obstet Gynecol Reprod Biol* 2004;112(1):29-35. doi: 10.1016/S0301-2115(03)00313-0
12. Vaughan C, White N, Keogh L, et al. Listening to North Yarra Communities about Female Genital Cutting. Melbourne, Australia: The University of Melbourne, 2014.
13. Vaughan C, White N, Keogh L, et al. Female Genital Mutilation/Cutting in Regional Victoria. Research to Practice. Melbourne, Australia: The University of Melbourne, 2014.

For peer review only

Supplementary File 4: Theme Matrix (Mapping Included Studies to Themes)

		1. Communication is key			2. Access to care: influenced by an interaction of multi-level community and health service processes				3. Cultural and bodily dissonance: striving for cultural and bodily integrity				4. Disempowering care encounters				5. Positive care encounters	
**No.**	**Study Reference**	**1.1**	**1.2**	**1.3**	**2.1**	**2.2**	**2.3**	**2.4**	**3.1**	**3.2**	**3.3**	**3.4**	**4.1**	**4.2**	**4.3**	**4.4**	**5.1**	**5.2**
**1**	Abdi, R. (2012)[1]			X		X				X			X					
**2**	Abdullahi, A., Copping, J., Kessel, A., Luck, M., & Bonell, C. (2009)[2]	X			X		X			X			X					X
**3**	Ahlberg, B. M., Krantz, I., Lindmark, G., & Warsame, M. (2004)[3]	X												X		X		
**4**	Ahmed, M., (2005)[4]		X	X									X					X
**5**	Ameresekere, M., Borg, R., Frederick, J., Vragovic, O., Saia, K., & Raj, A. (2011)[5]		X		X		X							X	X	X	X	X
**6**	Asefaw, F. (2007)[6]		X	X	X			X	X	X	X	X	X	X	X	X	X	X
**7**	Baldeh, F. (2013)[7]		X	X				X	X	X	X		X	X	X	X		X
**8**	Ballesteros Meseguer, C., Almansa Martinez, P., Pastor Bravo, M. d. M., & Jimenez Ruiz, I. (2014)[8]						X			X						X	X	
**9**	Behrendt, A. (2011)[9]		X	X				X	X				X	X	X	X	X	
**10**	Beine, K., Fullerton, J., Palinkas, L., & Anders, B. (1995)[10]		X	X	X						X	X	X		X	X	X	X
**11**	Berggren, V., Bergstrom, S., & Edberg, A.-K. (2006)[11]	X	X	X	X	X	X	X	X	X	X	X	X	X	X	X	X	X
**12**	Betts, V. (2011)[12]	X	X	X	X	X	X	X	X		X	X	X	X	X	X	X	X

For peer review only

		1. Communication is key			2. Access to care: influenced by an interaction of multi-level community and health service processes				3. Cultural and bodily dissonance: striving for cultural and bodily integrity				4. Disempowering care encounters				5. Positive care encounters	
**No.**	**Study Reference**	**1.1**	**1.2**	**1.3**	**2.1**	**2.2**	**2.3**	**2.4**	**3.1**	**3.2**	**3.3**	**3.4**	**4.1**	**4.2**	**4.3**	**4.4**	**5.1**	**5.2**
**13**	Bravo Pastor del Mar, M. (2014)[13]		X				X	X	X		X	X	X	X		X	X	X
**14**	Bulman, K. H., & McCourt, C. (2002)[14]	X		X	X		X	X						X		X	X	X
**15**	Carroll, J., Epstein, R., Fiscella, K., Gipson, T., Volpe, E., & Jean-Pierre, P. (2007)[15]	X		X	X			X						X		X		X
**16**	Chalmers, B., & Omer-Hashi, K. (2002)[16]	X		X	X		X	X	X	X			X	X	X	X	X	X
**17**	Degni, F., Suominen, S. B., El Ansari, W., Vehviläinen-Julkunen, K., & Essen, B. (2014)[17]	X	X		X	X		X	X		X	X		X			X	X
**18**	d'Entremont, M., Smythe, L., & McAra-Couper, J. (2014)[18]	X	X			X				X			X	X	X	X	X	X
**19**	Essén, B., Johnsdotter, S., Hovelius, B., Gudmundsson, S., Sjöberg, N. O., Friedman, J., & Östergren, P. O. (2000)[19]						X	X							X	X	X	
**20**	Fawcett, L., (2014)[20]	X	X	X	X	X		X	X	X			X	X	X	X		X
**21**	Gali, M. A. (1997)[21]	X		X			X	X	X	X	X	X	X	X	X	X	X	X
**22**	Ghebre, R. G., Sewali, B., Osman, S., Adawe, A., Nguyen, H. T., Okuyemi, K. S., & Joseph, A. (2015)[22]	X			X	X	X			X								
**23**	Glazer, E., (2012)[23]		X	X	X			X	X	X	X		X		X	X	X	X
**24**	Glover, J., Liebling, H., Barrett, H., & Goodman, S. (2017)[24]			X			X	X	X	X	X	X	X	X	X	X	X	X

For peer review only

		1. Communication is key			2. Access to care: influenced by an interaction of multi-level community and health service processes				3. Cultural and bodily dissonance: striving for cultural and bodily integrity				4. Disempowering care encounters				5. Positive care encounters	
**No.**	**Study Reference**	**1.1**	**1.2**	**1.3**	**2.1**	**2.2**	**2.3**	**2.4**	**3.1**	**3.2**	**3.3**	**3.4**	**4.1**	**4.2**	**4.3**	**4.4**	**5.1**	**5.2**
**25**	Guerin, P. B., Allotey, P., Elmi, F. H., & Baho, S. (2006)[25]			X			X		X		X	X	X	X	X	X		X
**26**	Hill, N., Hunt, E., & Hyrkas, K. (2012)[26]	X			X	X	X	X	X		X				X	X	X	X
**27**	Hussein, E., (2010)[27]	X	X	X	X			X	X	X			X	X		X		X
**28**	Hussen, M. A. (2014)[28]	X	X	X	X		X	X	X	X	X		X			X	X	X
**29**	Johansen, R.E. (2017)[29]				X				X		X	X						X
**30**	Jones, A. (2010)[30]		X		X	X	X		X		X			X		X	X	X
**31**	Khaja, K. (2004)[31]		X	X	X	X	X				X				X	X		
**32**	Khaja, K., Lay, K., & Boys, S. (2010)[32]								X				X	X				X
**33**	Lundberg, P. C., & Gerezgiher, A. (2008)[33]		X		X	X		X	X	X	X	X			X	X	X	X
**34**	Maier, C. (2003)[34]				X	X	X	X	X	X	X	X	X	X	X	X	X	X
**35**	Maternity Action. (2014)[35]	X	X	X	X			X	X				X	X	X	X		X
**36**	McNeely, S., & Christie-de Jong, F. (2016)[36]		X					X	X	X			X			X		X
**37**	Moxey, J. M., & Jones, L. L. (2016)[37]	X	X		X		X	X		X	X		X		X	X	X	X
**38**	Murray, L., Windsor, C., Parker, E., & Tewfik, O. (2010)[38]	X	X	X	X		X	X	X				X	X	X	X	X	

For peer review only

		1. Communication is key			2. Access to care: influenced by an interaction of multi-level community and health service processes				3. Cultural and bodily dissonance: striving for cultural and bodily integrity				4. Disempowering care encounters				5. Positive care encounters	
**No.**	**Study Reference**	**1.1**	**1.2**	**1.3**	**2.1**	**2.2**	**2.3**	**2.4**	**3.1**	**3.2**	**3.3**	**3.4**	**4.1**	**4.2**	**4.3**	**4.4**	**5.1**	**5.2**
**39**	Norman, K., Gegzabher, S. B., & Otoo-Oyortey, N. (2016)[39]		X	X			X	X					X	X				X
**40**	Norman, K., Hemmings, J., Hussein, E., & Otoo-Oyortey, N. (2009)[40]	X	X	X	X		X	X	X	X	X		X	X	X	X	X	X
**41**	O'Brien, O., Baldeh, F., Hassan, J., & Baillie, M. (2017)[41]	X	X				X	X	X	X	X	X	X		X	X	X	X
**42**	O'Brien, O., Baldeh, F., Sivapatham, S., Brown, E., & O'May, F. (2016)[42]	X	X	X			X	X	X				X			X	X	X
**43**	Palfreyman, A., Brown, E., & Nam, S. (2011)[43]	X	X	X			X	X	X		X	X	X	X		X	X	X
**44**	Recchia, N., & McGarry, J. (2017)[44]		X	X									X	X		X		X
**45**	Safari, F., (2013)[45]		X		X	X			X		X	X						
**46**	Salad, J., Verdonk, P., de Boer, F., & Abma, T. A. (2015)[46]	X	X		X	X	X		X	X			X			X		
**47**	Shaw, E. (1985)[47]						X	X	X	X	X	X				X		X
**48**	Shermarke, M.A.A. (1996)[48]		X					X	X					X	X	X	X	X
**49**	Straus, L., McEwen, A., & Hussein, F. M. (2009)[49]	X		X	X	X	X	X	X		X		X	X	X	X		X
**50**	Thierfelder, C. (2003)[50]			X								X						
**51**	Thierfelder, C., Tanner, M., & Bodiang, C. M. K. (2005)[51]	X	X		X		X	X	X	X	X		X	X	X	X		X

For peer review only

		1. Communication is key			2. Access to care: influenced by an interaction of multi-level community and health service processes				3. Cultural and bodily dissonance: striving for cultural and bodily integrity				4. Disempowering care encounters				5. Positive care encounters	
**No.**	**Study Reference**	**1.1**	**1.2**	**1.3**	**2.1**	**2.2**	**2.3**	**2.4**	**3.1**	**3.2**	**3.3**	**3.4**	**4.1**	**4.2**	**4.3**	**4.4**	**5.1**	**5.2**
**52**	Upvall, M. J., Mohammed, K., & Dodge, P. D. (2009)[52]	X	X	X	X	X	X		X				X	X		X	X	X
**53**	Vangen, S., Johansen, R. E. B., Sundby, J., Traeen, B., & Stray-Pedersen, B. (2004)[53]	X	X	X	X	X	X	X	X	X			X	X	X	X	X	
**54**	Vaughan, C., White, N., Keogh, L., Tobin, J., Ha, B., Ibrahim, M., & Bayly, C. (2014)[54]	X	X	X	X	X	X	X	X	X	X	X	X	X	X	X	X	X
**55**	Vaughan, C., White, N., Keogh, L., Tobin, J., Murdolo, A., Quiazon, R., & Bayly, C. (2014)[55]	X	X	X	X		X	X	X	X	X	X	X	X	X	X	X	X
**56**	Vloeberghs, E., van der Kwaak, A., Knipscheer, J., & van den Muijsenbergh, M. (2012)[56]	X	X	X	X			X	X	X			X	X	X		X	X
**57**	Wiklund, H., Aden, A. S., Högberg, U., Wikman, M., & Dahlgren, L. (2000)[57]	X		X	X	X	X	X	X				X	X	X	X	X	X
	**Total Studies per Theme**	**31**	**37**	**34**	**35**	**19**	**34**	**38**	**40**	**28**	**28**	**19**	**40**	**36**	**32**	**46**	**34**	**45**

For peer review only

Theme Key – for Theme Matrix

Descriptive Theme No.	Theme Name
**Analytical Theme 1: Communication is key**	
**1.1**	Language barriers and interpretation challenges
**1.2**	"Can't talk, not asked": double silence and cultural taboo
**1.3**	Cultural (in)sensitivity
**Analytical Theme 2: Access to care: influenced by an interaction of multi-level community and health service processes**	
**2.1**	Influence of cultural norms
**2.2**	Influence of the family
**2.3**	Knowledge and information about FGM/C services
**2.4**	'Hit and miss' care
**Analytical Theme 3: Cultural and bodily dissonance: striving for cultural and bodily integrity**	
**3.1**	Moving from normal to different
**3.2**	Threat to the self: re-living FGM/C pain during clinical interventions
**3.3**	Being opened: complexities around deinfibulation
**3.4**	Being changed: complexities around re-infibulation
**Analytical Theme 4: Disempowering care encounters**	
**4.1**	Being exposed and humiliated
**4.2**	Being judged and stereotyped
**4.3**	Lacking choice, power and control
**4.4**	Feeling unsafe and vulnerable
**Analytical Theme 5: Positive care encounters**	
**5.1**	Trusting and appreciating providers and the system
**5.2**	Voicing healthcare needs and preferences

For peer review only

References of Included Studies - Cited in Theme Matrix

1. Abdi R. Carving culture: Creating identity through female genital cutting. *Durham Anthropology Journal* 2012;18(1):115-53.

2. Abdullahi A, Copping J, Kessel A, et al. Cervical screening: perceptions and barriers to uptake among Somali women in Camden. *Public Health* 2009;123(10):680-5. doi: 10.1016/j.puhe.2009.09.011

3. Ahlberg BM, Krantz I, Lindmark G, et al. 'It's only a tradition': making sense of eradication interventions and the persistence of female 'circumcision' within a Swedish context. *Crit Soc Policy* 2004;24(1):50-78. doi: 10.1107/0261018304039679

4. Ahmed M. Attitudes towards FGM among Somali women living in the UK. In: Momoh C, ed. Female Genital Mutilation. London: Radcliffe 2005:95-117.

5. Ameresekere M, Borg R, Frederick J, et al. Somali immigrant women's perceptions of cesarean delivery and patient-provider communication surrounding female circumcision and childbirth in the USA. *Int J Gynecol Obstet* 2011;115(3):227-30. doi: 10.1016/j.ijgo.2011.07.019

6. Asefaw F. [Female Genital Mutilation: A Field Study with Special Consideration of the Background as well as the Health and Psychosexual Consquences for those Affected and their Partners in Eritrea and in Germany] Weibliche Genitalbeschneidung: Eine Feldstudie Unter besonderer Berücksichtigung der Hintergründe Sowie der Gesundheitlichen und Psychosexuellen Folgen für Betroffene und Partner in Eritrea und Deutschland. Humboldt University of Berlin, 2007.

7. Baldeh F. Obstetric Care in Scotland: The Experience of Women who have Undergone Female Genital Mutilation (FGM) [MSc Thesis]. Queen Margaret University, 2013.

8. Ballesteros Meseguer C, Almansa Martinez P, Pastor Bravo MdM, et al. [The voice of women subjected to female genital mutilation in the region of Murcia, Spain] La Voz de las mujeres sometidas a mutilacion genital femenina en la region de Murcia. *Gac Sanit* 2014;28(4):287-91. doi: 10.1016/j.gaceta.2014.02.006

9. Behrendt A. Listening to African Voices: Female Genital Mutilation/Cutting among Immigrants in Hamburg: Knowledge, Attitudes and Practice. Hamburg, Germany: Plan, 2011.

10. Beine K, Fullerton J, Palinkas L, et al. Conceptions of prenatal care among Somali women in San Diego. *J Nurs Midwifery* 1995;40(4):376-81. doi: Doi 10.1016/0091-2182(95)00024-E

11. Berggren V, Bergstrom S, Edberg A-K. Being different and vulnerable: experiences of immigrant African women who have been circumcised and sought maternity care in Sweden. *J Transcult Nurs* 2006;17(1):50-7. doi: 10.1177/1043659605281981

For peer review only

12. Betts V. Transnational Gender, Sexuality and Identity Construction: Ambivalent Subjectivities of Somali Refugee Women in the GTA. MA Thesis, The University of Guelph, 2011.

13. Bravo Pastor del Mar M. [Women Subjected to Female Genital Mutilation: Knowledge for the Nursing Discipline] La Voz de las Mujeres Sometidas a Mutilación Genital Femenina: Saberes Para la Disciplina Enfermera [Tesis Doctoral]. University of Murcia, 2014.

14. Bulman KH, McCourt C. Somali refugee women's experiences of maternity care in west London: a case study. *Crit Public Health* 2002;12(4):365-80.

15. Carroll J, Epstein R, Fiscella K, et al. Caring for Somali women: implications for clinician-patient communication. *Patient Educ Couns* 2007;66(3):337-45. doi: 10.1016/j.pec.2007.01.008

16. Chalmers B, Omer-Hashi K. What Somali women say about giving birth in Canada. *J Reprod Infant Psychol* 2002;20(4):267-82. doi: 10.1080/0264683021000033183

17. Degni F, Suominen SB, El Ansari W, et al. Reproductive and maternity health care services in Finland: perceptions and experiences of Somali-born immigrant women. *Ethn Health* 2014;19(3):348-66. doi: 10.1080/13557858.2013.797567

18. d'Entremont M, Smythe L, McAra-Couper J. The sounds of silence: a hermeneutic interpretation of childbirth post excision. *Health Care Women Int* 2014;35(3):300-19. doi: 10.1080/07399332.2013.838245

19. Essén B, Johnsdotter S, Hovelius B, et al. Qualitative study of pregnancy and childbirth experiences in Somalian women resident in Sweden. *Br J Obstet Gynaecol* 2000;107(12):1507-12.

20. Fawcett L. Somali Refugee Women and their U.S. Healthcare Providers: Knowledge, Perceptions and Experiences of Childbearing [Doctor of Philosophy]. Arizona State University, 2014.

21. Gali MA. Female Circumcision: A Transcultural Study of Attitudes, Identity and Reproductive Health of East African Immigrants [Degree of Doctor of Philosophy in Psychology]. The Wright Institute, University of Berkeley, 1997.

22. Ghebre RG, Sewali B, Osman S, et al. Cervical cancer: barriers to screening in the Somali community in Minnesota. *J Immigr Minor Health* 2015;17(3):722-28. doi: 10.1007/s10903-014-0080-1

23. Glazer E. Embodiment, Pain and Circumcision in Somali-Canadian Women [M.Sc.]. University of Toronto, 2012.

24. Glover J, Liebling H, Barrett H, et al. The psychological and social impact of female genital mutilation: a holistic conceptual framework. *J Int Stud* 2017;10(2):219-38.

25. Guerin PB, Allotey P, Elmi FH, et al. Advocacy as a means to an end: assisting refugee women to take control of their reproductive health needs. *Women Health* 2006;43(4):7-25.

For peer review only

26. Hill N, Hunt E, Hyrkas K. Somali immigrant women's health care experiences and beliefs regarding pregnancy and birth in the United States. *J Transcult Nurs* 2012;23(1):72-81. doi: 10.1177/1043659611423828 [published Online First: 2011/11/05]

27. Hussein E. Women's Experiences, Perceptions and Attitudes of Female Genital Mutilation: The Bristol PEER Study. London: FORWARD, 2010.

28. Hussen MA. Services for Women with Female Genital Mutilation in Christchurch: Perspectives of Women and their Health Providers. University of Canterbury, 2014.

29. Johansen RE. Undoing female genital cutting: perceptions and experiences of infibulation, defibulation and virginity among Somali and Sudanese migrants in Norway. *Cult Health Sex* 2017;19(4):528-42. doi: 10.1080/13691058.2016.1239838

30. Jones A. Working Psychologically with Female Genital Mutilation: An Exploration of the Views and Experiences of Women who have Experienced FGM and of Clinical Psychologists. University of East London, 2010.

31. Khaja K. Female Circumcision: Life Histories of Somali Women [Doctor of Philosophy]. The University of Utah, 2004.

32. Khaja K, Lay K, Boys S. Female circumcision: toward an inclusive practice of care. *Health Care Women Int* 2010;31(8):686-99. doi: 10.1080/07399332.2010.490313

33. Lundberg PC, Gerezgiher A. Experiences from pregnancy and childbirth related to female genital mutilation among Eritrean immigrant women in Sweden. *Midwifery* 2008;24(2):214-25. doi: 10.1016/j.midw.2006.10.003

34. Maier C. [Echoes of Silence: Voices of Concern for Genital Mutilation in African Immigrant Women in Vienna: An Ethnological Study] Echo des Schweigens: Stimmen der Betroffenheit zur Genitalverstümmelung bei Afrikanischen Immigrantinnen in Wien: Ethnologische Studie. University of Vienna, 2003.

35. Maternity Action. Women's Voices on Health: Addressing Barriers to Accessing Primary Care. London: Women's Health and Equality Consortium, 2014.

36. McNeely S, Christie-de Jong F. Somali refugees' perspectives regarding FGM/C in the US. *Int J Migr Health Soc Care* 2016;12(3):157-69. doi: 10.1108/Ijmhsc-09-2015-0033

37. Moxey JM, Jones LL. A qualitative study exploring how Somali women exposed to female genital mutilation experience and perceive antenatal and intrapartum care in England. *BMJ Open* 2016;6(1):e009846. doi: 10.1136/bmjopen-2015-009846

38. Murray L, Windsor C, Parker E, et al. The experiences of African women giving birth in Brisbane, Australia. *Health Care Women Int* 2010;31(5):458-72. doi: 10.1080/07399330903548928

39. Norman K, Gegzabher SB, Otoo-Oyortey N. "Between Two Cultures": A Rapid PEER Study Exploring Migrant Communities' Views on Female Genital Mutilation in Essex and Norfolk, UK. FORWARD & National FGM Centre Report, , 2016.

For peer review only

40. Norman K, Hemmings J, Hussein E, et al. "FGM is Always With Us": Experiences, Perceptions and Beliefs of Women Affected by Female Genital Mutilation in London: Results from a PEER Study. London: Options Consultancy Services and FORWARD, 2009.

41. O'Brien O, Baldeh F, Hassan J, et al. My Voice: Participatory Action Research Project with Men, Women and Young People on Female Genital Mutilation (FGM) in Scotland: (Phase 2). Waverly Care, Edinburgh: Queen Margaret University, 2017.

42. O'Brien O, Baldeh F, Sivapatham S, et al. Participatory Action Research Project with Men, Women and Young People on Female Genital Mutilation (FGM) in Scotland: (Phase 1). Waverly Care, Edinburgh: Queen Margaret University, 2016.

43. Palfreyman A, Brown E, Nam S. Understanding Female Genital Mutilation in Birmingham: Findings from a PEER Study: Options Consultancy Services and Birmingham & Solihull Women's Aid, 2011.

44. Recchia N, McGarry J. "Don't judge me": narratives of living with FGM. *Int J Hum Rights Healthc* 2017;10(1):4-13. doi: 10.1108/Ijhrh-10-2016-0016

45. Safari F. A qualitative study of women's lived experience after deinfibulation in the UK. *Midwifery* 2013;29(2):154-8. doi: 10.1016/j.midw.2011.12.005

46. Salad J, Verdonk P, de Boer F, et al. "A Somali girl is Muslim and does not have premarital sex - is vaccination really necessary?" A qualitative study into the perceptions of Somali women in the Netherlands about the prevention of cervical cancer. *Int J Equity Health* 2015;14(1):1-13. doi: 10.1186/s12939-015-0198-3

47. Shaw E. Female circumcision: perceptions of clients and caregivers. *J Am Coll Health* 1985;33(5):193-7. doi: 10.1080/07448481.1985.9939604

48. Shermarke MAA. Understanding the Canadian Community Context of Female Circumcision [M.S.W.]. McGill University, 1996.

49. Straus L, McEwen A, Hussein FM. Somali women's experience of childbirth in the UK: perspectives from Somali health workers. *Midwifery* 2009;25(2):181-6. doi: 10.1016/j.midw.2007.02.002

50. Thierfelder C. Female Genital Mutilation and the Swiss Health Care System. University of Basel, 2003.

51. Thierfelder C, Tanner M, Bodiang CMK. Female genital mutilation in the context of migration: experience of African women with the Swiss health care system. *Eur J Public Health* 2005;15(1):86-90. doi: 10.1093/eurpub/cki120

52. Upvall MJ, Mohammed K, Dodge PD. Perspectives of Somali Bantu refugee women living with circumcision in the United States: a focus group approach. *Int J Nurs Stud* 2009;46(3):360-8. doi: 10.1016/j.ijnurstu.2008.04.009

53. Vangen S, Johansen REB, Sundby J, et al. Qualitative study of perinatal care experiences among Somali women and local health care professionals in Norway. *Eur J Obstet Gynecol Reprod Biol* 2004;112(1):29-35. doi: 10.1016/S0301-2115(03)00313-0

For peer review only

54. Vaughan C, White N, Keogh L, et al. Listening to North Yarra Communities about Female Genital Cutting. Melbourne, Australia: The University of Melbourne, 2014.

55. Vaughan C, White N, Keogh L, et al. Female Genital Mutilation/Cutting in Regional Victoria. Research to Practice. Melbourne, Australia: The University of Melbourne, 2014.

56. Vloeberghs E, van der Kwaak A, Knipscheer J, et al. Coping and chronic psychosocial consequences of female genital mutilation in the Netherlands. *Ethn Health* 2012;17(6):677-95. doi: 10.1080/13557858.2013.771148

57. Wiklund H, Aden AS, Högberg U, et al. Somalis giving birth in Sweden: a challenge to culture and gender specific values and behaviours. *Midwifery* 2000;16(2):105-15. doi: http://dx.doi.org/10.1054/midw.1999.0197

For peer review only

Supplementary File 5: Detailed Characteristics of Included Studies

No.	Authors and date	Country	Study aims, focus and/or research question	Region and Setting (where specified)	Methodology and/or theoretical perspective	Methods and recruitment (where stated)	Data analysis	Whole study sample	Eligible participants and country of origin
**1.**	Abdi, R. (2012)[1]	UK	To determine the role of FGM/C in creating a social, individual and gendered identity for a Somali woman.	East and Northeast regions of London.<br><br>African Well Women's Service in North London.	Narrative and life history approach.	In-depth interviews. Recruited women who held a strong anti-FGM/C opinion.<br><br>Snowball sampling used.	Analysis using theoretical concept of the 'three-bodies'.	2 gynaecologists, 1 counsellor and 1 midwife<br>16 Somali women<br>2 men<br><br>Age range 20 – 72 years	16 Somali women
**2.**	Abdullahi, A., Copping, J., Kessel, A., Luck, M., & Bonell, C. (2009)[2]	UK	To explore the barriers to uptake of cervical screening and to identify strategies for overcoming these barriers.	London boroughs, Camden UK, North London.<br><br>Somali community centre.	Qualitative methods	Focus groups and in-depth interviews.<br><br>Recruitment from a range of settings.<br><br>Included snowball sampling.	Thematic analysis informed by an interpretivist approach.	50 Somali-born women<br><br>Age range 25-64 years	50 Somali-born women
**3.**	Ahlberg, B. M., Krantz, I., Lindmark, G., & Warsame, M. (2004)[3]	Sweden	Questions why FGM/C persists despite eradication interventions and the migration to non-FGM/C practising areas.	Different forums including a school, youth clinics and a girl's football team.	Emergent research design in the tradition of qualitative research.	Individual and group interviews.<br><br>Snowball sampling used.	Thematic analysis.	30 men, 50 women, 10 girls, 5 boys and 15 professionals	50 women of Somali origin
**4.**	Ahmed, M., (2005)[4]	UK	To explore the influence of cross-cultural psychology, women's experience of FGM/C and their attitudes towards it.	West, East and South London and Sheffield.<br><br>Women's homes, community centre and a mosque.	Qualitative methods	Focus groups recruited via word of mouth, through attending Somali community centres and verbal advertising of the project	Thematic analysis.	56 women<br><br>Age range 18–70 years	56 women. The majority born in Somalia – with some born in UK and the Middle East

For peer review only - http://bmjopen.bmj.com/site/about/guidelines.xhtml

No.	Authors and date	Country	Study aims, focus and/or research question	Region and Setting (where specified)	Methodology and/or theoretical perspective	Methods and recruitment (where stated)	Data analysis	Whole study sample	Eligible participants and country of origin
**5.**	Ameresekere , M., Borg, R., Frederick, J., Vragovic, O., Saia, K., & Raj, A. (2011)[5]	USA	To assess the prevalence of FGM/C in the interviewed sample, gain insight into commonly held beliefs about caesarean delivery, and understand patient perceptions of communication surrounding caesarean delivery and FGM/C by obstetric providers in the USA.	Greater Boston	Exploratory qualitative study	Semi structured in-depth interviews<br><br>Snowball sampling and linkage with the Somali Development Centre (SDC), a local community based organization	Thematic analysis	23 Somali immigrant women<br><br>Age range 25-52 years	23 Somali participants
**6.**	Asefaw, F. (2007)[6]	Eritrea and Germany	To explore whether migration changes their attitude towards FGC. How do circumcised women deal with it in a society that opposes FGC? Can coherence and reasons be determined for why circumcised women subjectively suffer more in migration compared with when they live in their country of origin?	In the migrant's homes, in clinics or as part of awareness campaigns of the woman's organisation FORWARD	Mixed methods. Survey and qualitative methods	Survey and semi-structured qualitative interviews.	Unclear	420 women and 50 men<br><br>Age range 15-70 years	31 Eritrean migrants
**7.**	Baldeh, F. (2013)[7]	Scotland	To explore the experiences of obstetric care in Scotland among women who have undergone FGM/C. The objectives are to explore: · How women with FGM experience obstetric care in Scotland · The role of health workers in helping or hindering communication; · Potential changes and improvements in services.	Glasgow and Edinburgh<br><br>In participants homes	Qualitative approach interpretivist paradigm and a feminist perspective	In-depth interviews.<br><br>Convenience sampling used.	Thematic analysis	7 women	7 women in total. 2 from The Gambia, 1 from Ghana and 1 from Sudan.

No.	Authors and date	Country	Study aims, focus and/or research question	Region and Setting (where specified)	Methodology and/or theoretical perspective	Methods and recruitment (where stated)	Data analysis	Whole study sample	Eligible participants and country of origin
**8.**	Ballesteros Meseguer, C., Almansa Martinez, P., Pastor Bravo, M. d. M., & Jimenez Ruiz, I. (2014)[8]	Spain	To explore the perceptions of a group of women who had experienced FGM/C regarding their sexual and reproductive health, with an aim to consider the impact on their sexual relationships, pregnancy and labour, as well as the social repercussions of this practice.	Region of Murcia, Spain.<br><br>In participants' homes.	Phenomenological qualitative study	In-depth interviews using structured open-ended approach<br><br>Informal conversations with midwives	Discourse analysis	9 sub-Saharan Africa women.<br><br>Mean age 30 years	9 women from Senegal
**9.**	Behrendt, A. (2011)[9]	Germany	To listen to the opinions, perceptions and propositions of immigrants from Sub-Saharan Africa regarding the practice of FGM/C. To develop an effective and locally adapted approach to promote the abandonment of FGM/C among immigrant groups in Hamburg.	Hamburg	Participatory, cooperative approach which engages community members in a joint evaluation research process. Triangulated research design behaviour change model.	Mixed methods.<br><br>Qualitative and quantitative data collection from different target groups (1) key informant (health professional) interviews, (2) semi-structured individual interviews with African community members, (3) survey with community members. Snowball sampling	Qualitative data analysed and classified into themes.<br><br>Quantitative data analysed using SPSS	Total number of 1,767 participants with African migration background were interviewed for the quantitative survey<br><br>91 semi structured interviews with African community members<br><br>27 interviews with health professionals<br><br>Age range 15-83 years	91 key informant interviews - largely from Ghana, Nigeria and Togo
**10.**	Beine, K., Fullerton, J., Palinkas, L., & Anders, B. (1995)[10]	USA	to understand their cultural beliefs and behaviours better during pregnancy and to determine their attitudes toward their prenatal care experiences in San Diego,	Conducted at International Rescue Committee office or private	Exploratory study	Focus group interview.<br><br>Recruitment via community social networks	Content analysis	14 women<br><br>Age range 20 to 42 years	14 Somali women

No.	Authors and date	Country	Study aims, focus and/or research question	Region and Setting (where specified)	Methodology and/or theoretical perspective	Methods and recruitment (where stated)	Data analysis	Whole study sample	Eligible participants and country of origin
			in order to inform providers and enhance culturally relevant service programs.	homes of group participants					
**11.**	Berggren, V., Bergstrom, S., & Edberg, A.-K. (2006)[11]	Sweden	To explore the experience of FGM and the experiences of encounters with Swedish maternal health care among women from Eritrea, Somalia, and Sudan living in Sweden.	Three different cities in Sweden,<br><br>Conducted in the women's homes	Exploratory qualitative study	26 interviews recruited via snowball sampling or net-work sampling	Latent content analysis	22 women, 6 from Eritrea, 11 from Somalia, and 5 from Sudan (1 excluded as no FGM)<br><br>Age range 24 to 73 years	21 women from Eritrea, Somalia, or Sudan
**12.**	Betts, V. (2011)[12]	Canada	To critically examine popular ideas of Somali women's identity, gender and sexuality in Canada; and, to evaluate Somali women's experiences with Canadian health care professionals, and how it compares with their experiences with medical professionals in Somalia.	Greater Toronto Area, Canada. From Community Centre in a small office	Anthropological methods	Individual and paired interviews one discussion group of fourteen women.	Placed loosely into categories	Thirteen women aged 18-65 years	13 Somali women

No.	Authors and date	Country	Study aims, focus and/or research question	Region and Setting (where specified)	Methodology and/or theoretical perspective	Methods and recruitment (where stated)	Data analysis	Whole study sample	Eligible participants and country of origin
**13.**	Bravo Pastor del Mar, M. (2014)[13]	Spain	To identify the knowledge, attitudes and experiences regarding genital mutilation of a group of women from Sub-Saharan African countries where FGM/C is a traditional practice.	Region of Murcia, Spain.<br><br>The interviews were held individually at the homes of the participants, at their request, always in the absence of the husband and occasionally in the presence of minor children.	A qualitative study using the life story method	Life Story interviews held until data saturation was reached. Informal conversations including telephone calls, emails, informal chats and informal meetings with key informants. Socio-demographic Questionnaire<br><br>Contact through different associations for African women and snowball sampling	The analysis was carried out using the categories that emerged from the narratives collected in the interviews.	20 Senegalese women, 2 Nigerian women and 2 Gambian women<br><br>Age range 23 - 42 years	20 Senegalese women, 2 Nigerian women and 2 Gambian women
**14.**	Bulman, K. H., & McCourt, C. (2002)[14]	UK	To develop an understanding of the reality faced by Somali women in their contacts with the maternity services in the UK and<br>to explore professionals perceptions of Somali women and their maternity care needs - how they approached providing for these needs and their ideas for improving services.	West London<br><br>Conducted in participants homes or clinics	Qualitative (ethnographic context narrative approach)	6 individual interviews and two focus groups with Somali women, recruited via snowball sampling method<br><br>2 focus groups with midwives, (caseload and hospital midwives). Individual interviews with a Somali health-link worker, a woman on the Health and Race Working	Thematic analysis	12 Somali women participating in qualitative interviews<br><br>Health workers (number not specified) were also interviewed	12 Somali women

No.	Authors and date	Country	Study aims, focus and/or research question	Region and Setting (where specified)	Methodology and/or theoretical perspective	Methods and recruitment (where stated)	Data analysis	Whole study sample	Eligible participants and country of origin
						Group, and an obstetric registrar.			
**15.**	Carroll, J., Epstein, R., Fiscella, K., Gipson, T., Volpe, E., & Jean-Pierre, P. (2007)[15]	USA	To explore the range of both Bantu and other Somali women's experiences with communication about preventive health services in the US in order to understand the role communication plays in disparities in this population.	Rochester, NY, USA. At a time and place convenient to the participant	Grounded theory	Individual interviews, a Somali women's focus group was conducted to check thematic validity and clarify questions and concepts. Word of mouth, key informants and primary care provider referrals; and snowballing	Grounded theory, a coding/ editing method that extracts emerging themes	34 women Age range 18 - 53 years (median age 27 years)	34 Somali refugee women
**16.**	Chalmers, B., & Omer-Hashi, K. (2002)[16]	Canada	To explore recent perinatal experiences in Canada	Greater Toronto region, at participants home	Unclear	Sampling from community sites and snowball sampling.<br><br>Structured questionnaire with 4 open ended questions, administered face to face involving reflection and discussion	Unclear	432 Somali women<br><br>Mean age 34 years	415 Somali women<br><br>(who responded to the open ended questions)
**17.**	Degni, F., Suominen, S. B., El Ansari, W., Vehviläinen-Julkunen, K., & Essen, B. (2014)[17]	Finland	To explore immigrant Somali women's experiences of reproductive and maternity health care services (RMHCS) and their perceptions about the service providers.	Helsinki, Vantaa, Espoo and Turku cities in Finland.<br><br>Conducted at	Exploratory, qualitative study	Focus group discussions via purposeful sampling	Guided by concepts of cultural care	70 women<br><br>Age range 18~50 years	70 Somali women

No.	Authors and date	Country	Study aims, focus and/or research question	Region and Setting (where specified)	Methodology and/or theoretical perspective	Methods and recruitment (where stated)	Data analysis	Whole study sample	Eligible participants and country of origin
				woman's home and swimming pool hall					
**18.**	d'Entremont, M., Smythe, L., & McAra-Couper, J. (2014)[18]	France	Explored the experiences of childbirth for women who have been excised.	At a place of the woman's choosing	Hermeneutic phenomenology Gadamerian philosophy	Interviews Purposive sampling methods	Constant, circular process of thematic, textual interpretations	4 women	4 women from different countries
**19.**	Essén, B., Johnsdotter, S., Hovelius, B., Gudmundsson, S., Sjöberg, N. O., Friedman, J., & Östergren, P. O. (2000)[19]	Sweden	To explore the attitudes, strategies and habits of Somalian immigrant women related to pregnancy and childbirth, in order to gain an understanding as to how cultural factors might affect perinatal outcome.	Localities of the different Somalian immigrant associations in their own residential areas	Qualitative	Qualitative in-depth interviews. Recruited from community organisations	Systematic textual analysis	15 women<br><br>Age range 20-55 years	15 Somali women
**20.**	Fawcett, L., (2014)[20]	USA	To identify and measure patterns of cultural knowledge and variations (cross-cultural agreement and disagreement) between Somali resettled refugee women (SRRW) and healthcare providers (HCP) on childbearing models. To investigate and measure intra-cultural variations in the domain of childbearing knowledge among Somali resettled refugee women. To explore the subjective	Arizona Metropolitan Phoenix area, in a café or participants' homes	Quantitative survey and ethnographic methodologies and phenomenological approaches using open-ended semi-structured interviews.<br><br>Used theory of Cultural Consensus Model and concepts of embodiment.	Culture consensus questionnaire (CCM), participatory observations, in-depth semi-structured interviews.<br><br>Used snowball sampling via community organisations	Phenomenological analysis and inductive explorative approach	Questionnaires (N=174)<br><br>Interviews; Somali resettled refugee women (SRRW, n=30); Healthcare Providers (HCP, n=10)<br><br>Age range 18-70 years	30 Somali resettled refugee women

No.	Authors and date	Country	Study aims, focus and/or research question	Region and Setting (where specified)	Methodology and/or theoretical perspective	Methods and recruitment (where stated)	Data analysis	Whole study sample	Eligible participants and country of origin
			and intersubjective perceptions of female circumcision.						
**21.**	Gali, M. A. (1997)[21]	USA	To explore the relationship between the psychological and medical concerns circumcised women face and barriers to reproductive health care delivery in the United States.	San Francisco Bay Area.	Qualitative exploratory research	Semi-structured interviews were conducted in person and by mail.<br><br>Recruitment using community organisations	Content analysis	26 women<br><br>Aged 21 -45 years	26 women from Sudan, Eritrea, Ethiopia and Egypt
**22.**	Ghebre, R. G., Sewali, B., Osman, S., Adawe, A., Nguyen, H. T., Okuyemi, K. S., & Joseph, A. (2015)[22]	USA	Exploring suitable language, structure, and context to describe cervical cancer prevention and screening methods among women in Minnesota's Somali community.	Minnesota<br><br>Community locations	Qualitative study with community engagement socio-ecological framework	Informant interviews. Used snowball sampling.	Themes and subthemes using the socio-ecological framework	23 participants<br><br>Age range 26-45 years	23 Somali women
**23.**	Glazer, E., (2012)[23]	Canada	Aimed to explore this possibly primarily through subjective reports of pain discussing testable pain along with interpretation and meaning of bodily pain and FGM/C from the perspective of women with FGM/C	Greater Toronto Area, Canada at community health centre (CHC)	Mixed-methods, including a qualitative component drawing upon interpretive and embodied phenomenology	Qualitative interview semi-structured interviews using a convenience sample	Phenomenological analysis	14 Somali-Canadian participants<br><br>Age range 21-46 years	14 Somali-Canadian participants

No.	Authors and date	Country	Study aims, focus and/or research question	Region and Setting (where specified)	Methodology and/or theoretical perspective	Methods and recruitment (where stated)	Data analysis	Whole study sample	Eligible participants and country of origin
**24.**	Glover, J., Liebling, H., Barrett, H., & Goodman, S. (2017)[24]	UK	Investigate the experiences of women who had undergone FGM and the psychological effects they had experienced. It also sought to understand the psychological impact of physical health complications, survivor's resilience and their cultural context	Multiple UK settings	A qualitative methodology of grounded theory Principles of feminist research were observed	In-depth interviews from a non-probability convenience sampling frame based on snowballing sampling	Theoretical memos and grounded theory analysis	20 women survivors of FGM<br><br>Mean age 38 years (SD=6.5, range=25-51)	20 women from various countries Somalia (n=10), Kenya (n=2), South Africa (n=6), and The Gambia (n=2)
**25.**	Guerin, P. B., Allotey, P., Elmi, F. H., & Baho, S. (2006)[25]	Australia and New Zealand	To enhance the understanding of the immigration experience on the reproductive health of women from Horn of African and Middle Eastern countries resident in Victoria to provide a safe means by which Somali women could express their views about FGM/C	Melbourne Australia and New Zealand.<br><br>In homes of the participant	Ethnographic cohort design Community-Based Participatory Research	In-depth, open-ended and unstructured interviews, focus group discussions and participant observation. Sampling involved using community networks and snowball approach.	Thematic analysis	Australia (n = 255) and New Zealand (n = 64). African women in the sample (n = 141) Middle Eastern women (n = 114) 54 women; 19 and 50 years	10 women from Somalia, Ethiopia, Eritrea, Sudan and Nigeria
**26.**	Hill, N., Hunt, E., & Hyrkas, K. (2012)[26]	USA	To describe and better understand Somali immigrant women's health care experiences and beliefs regarding pregnancy and birth in the Unites States.	North-eastern United States<br><br>Setting not given	Qualitative study	Focus group convenience sampling and women in turn identified friends and family	Thematic content analysis	18 participants<br><br>Age range 27-42 years	18 Somali women
**27.**	Hussein, E., (2010)[27]	UK	To gain an in-depth understanding of some of the experiences and perceptions of women coming from countries with high prevalence rates of FGM/C living in Bristol.	Setting not given	Participatory Ethnographic Evaluation and Research (PEER) and tenets of social construct	Informal in-depth interviews in third person loosely structured interviews. Equipping members from the community to become 'PEER researchers	Not stated	8 adult women	8 Somali and Sudanese adult women

No.	Authors and date	Country	Study aims, focus and/or research question	Region and Setting (where specified)	Methodology and/or theoretical perspective	Methods and recruitment (where stated)	Data analysis	Whole study sample	Eligible participants and country of origin
**28.**	Hussen, M. A. (2014)[28]	New Zealand	To understand the situation of both the Christchurch women with FGM and their health providers, in order to increase knowledge of FGM and ensure improved health services for women with this condition.	Christchurch, New Zealand, at community gatherings	Qualitative descriptive research methods	Focus group approach for women from East Africa known to the social worker and researcher<br><br>Individual interviews with health professionals	Thematic analysis	20 focus group participants – mean age 21-65 years<br><br>3 health providers	20 women from Eritrea, Ethiopia, Somalia and Sudan
**29.**	Johansen, R.E. (2017)[29]	Norway	Explores experiences and perceptions of premarital deinfibulation. It explores whether Somali and Sudanese men and women understand deinfibulation as a purely medical issue or whether their use of the services is also affected by the cultural meaning of infibulation.	Oslo and other villages and towns in Norway. From participant home, workplace or public space such as a coffee bar or a park	Qualitative study	In-depth and key informant interviews Participant observation validation seminars. Utilised Snowball sampling	Themes and patterns	23 women and 13 men<br><br>Age range 18-65 years	23 Somali and Sudanese women
**30.**	Jones, A. (2010)[30]	UK	To understand the experiences and views of women who had undergone female genital mutilation (FGM/C).	South England, UK, in participant own home or the community centre	Interpretative phenomenological analysis (IPA) relativist approach.	Semi-structured interview. Recruited from FORWARD, community group	Interpretative Phenomenological Analysis (IPA)	Six participants<br><br>Age range 19-45 years	6 participants from Sudan and Somalia
**31.**	Khaja, K. (2004)[31]	Canada and USA	To describe their experiences with circumcision and to share the personal and cultural meanings of those experiences	Toronto, Canada, and Salt Lake City, Utah. Setting not stated.	Life history methodology / ethnographic	Face-to-face, individual life history interviews. Purposive sampling recruited with the assistance of targeted Somali community members or service providers	Content analysis	17 women<br><br>Age range 20-79 years	17 Somali women

For peer review only - http://bmjopen.bmj.com/site/about/guidelines.xhtml

No.	Authors and date	Country	Study aims, focus and/or research question	Region and Setting (where specified)	Methodology and/or theoretical perspective	Methods and recruitment (where stated)	Data analysis	Whole study sample	Eligible participants and country of origin
**32.**	Khaja, K., Lay, K., & Boys, S. (2010)[32]	Canada and USA	Examined the lives of Somali Muslim women who were circumcised.	Ontario Canada and Salt Lake City, Utah North America Setting N/S	Exploratory qualitative	Face-to-face interviews	Not stated	17 circumcised Somali women<br><br>Age range 20-79 years	17 circumcised Somali women
**33.**	Lundberg, P. C., & Gerezgiher, A. (2008)[33]	Sweden	to explore the experiences from pregnancy and childbirth related to FGM/C among Eritrean immigrant women	At informants' homes or in a separate room	Ethnographic method	Semi-structured and open-ended in-depth interview Purposive sampling with snowball sampling technique	Thematic analysis	15 women<br><br>Age range 31-45 years	15 Eritrean women
**34.**	Maier, C. (2003)[34]	Austria	Three questions: · How did the affected woman experience and handle her genital mutilation when she was a girl? How did this influence her life and what are the physical and mental consequences? · In the context of FGM: Did immigration add new problem areas?	Not stated	Qualitative research	Guided interviews with a relatively open design	Not stated	3 women	3 women from different countries
**35.**	Maternity Action. (2014)[35]	UK	To explore potential barriers to healthcare, both for women in general, and for women who experience comparatively poor health outcomes, in order to contribute to an understanding of how these may be addressed	Leeds, Manchester and London, UK. Community groups were hosted and facilitated Organisations	Online survey, and a series of focus groups	Focus groups recruited from service user networks	Themes from the literature	261 in the survey<br><br>23 women in focus groups.	23 women from several countries
**36.**	McNeely, S., & Christie-de Jong, F. (2016)[36]	USA	To explore perspectives of Somali refugees on FGM/C and potential changes in, these after migration to the USA.	Denver metropolitan area, USA in participants' homes	Descriptive, socio-cultural study	Semi-structured and open-ended interviews snowball sampling with two gatekeepers	Thematic content analysis	13 Somali refugees<br><br>Age range 20-70 years	13 Somali women

No.	Authors and date	Country	Study aims, focus and/or research question	Region and Setting (where specified)	Methodology and/or theoretical perspective	Methods and recruitment (where stated)	Data analysis	Whole study sample	Eligible participants and country of origin
**37.**	Moxey, J. M., & Jones, L. L. (2016)[37]	UK	To explore how Somali women exposed to FGM, experience and perceive antenatal and intrapartum care in England	Birmingham, West Midlands<br><br>In a private room at a community centre	Descriptive qualitative methodology	Semi-structured Face-to-face interviews purposively sampled from community centres and using a snowball approach	Inductive thematic analysis	10 women<br><br>Age range 20-49 years	10 Somali women
**38.**	Murray, L., Windsor, C., Parker, E., & Tewfik, O. (2010)[38]	Australia	To uncover first-person descriptions of the birth experiences of African refugee women in Brisbane, Australia, and to explore the common themes that emerged from their experiences.	In room at a clinic or in participant own homes	Phenomenology Husserlian phenomenological framework.	Semi structured interviews purposeful, snowball strategy	Giorgi's phenomenological analysis.	10 women<br><br>Age range 20-40 years	10 women from Sudan Liberia Ethiopia Somalia
**39.**	Norman, K., Gegzabher, S. B., & Otoo-Oyortey, N. (2016)[39]	UK	Rapid participatory study amongst migrant communities in Norfolk and Essex, to help shed more light on this issue and to support their community engagement programme.	Norfolk, and Essex, in the community	Participatory Ethnographic Evaluation and Research (PEER) PEER is a qualitative, participatory research methodology	Interviews using snowball sampling	Participatory thematic analysis	18 peer researchers and 33 interviewees (each of the peer researchers chose two friends/peers to interview)	33 women from various countries
**40.**	Norman, K., Hemmings, J., Hussein, E., & Otoo-Oyortey, N. (2009)[40]	UK	To gain in-depth insights from women originally from high-prevalence FGM countries, and currently living in and around Westminster.	Borough of Westminster, London in the community	Participatory Ethnographic Evaluation and Research (PEER) a qualitative, participatory research method	In-depth conversational interviews snowball sampling used	Participatory thematic analysis	10 peer researchers conducted interviews with 3 friends each (n=30). Peer researchers were also interviewed.<br><br>Age range 25 years +	40 women<br><br>(10 peer researchers who were also interviewed and 30 other women) from Sudan, Eritrea and Somalia

No.	Authors and date	Country	Study aims, focus and/or research question	Region and Setting (where specified)	Methodology and/or theoretical perspective	Methods and recruitment (where stated)	Data analysis	Whole study sample	Eligible participants and country of origin
**41.**	O'Brien, O., Baldeh, F., Hassan, J., & Baillie, M. (2017)[41]	Scotland	To facilitate community engagement with FGM affected populations, to enable their voices to contribute to the development of awareness raising around FGM and to ensuring culturally appropriate FGM services for Scotland.	Glasgow and Edinburgh Scotland, in the community	Participatory Ethnographic Evaluation Research (PEER) ethnographic research principles	Interviews with contacts in community-based organisations and friends	Participatory analysis	19 peer researchers (who were also interviewed) and who then carried out 29 interviews	48 women<br><br>(19 peer researchers who were also interviewed and 29 women) from Sudan Nigeria Zimbabwe
**42.**	O'Brien, O., Baldeh, F., Sivapatham, S., Brown, E., & O'May, F. (2016)[42]	Scotland	To facilitate community engagement on FGM with women, men, young people and religious leaders to enable their voices to be heard and contribute to the development of awareness raising around FGM as well as ensuring culturally appropriate services for Scotland	Glasgow and Edinburgh Scotland, in the community,	Participatory Ethnographic Evaluation Research (PEER) ethnographic research principles	Interviews and focus groups. Convenience and snowball sampling through contacts	Participatory analysis	28 peer researchers (12 women, 16 men) who carried out 78 interviews	39 women<br><br>(9 female peer researchers who were also interviewed and 30 women) from 17 African countries
**43.**	Palfreyman, A., Brown, E., & Nam, S. (2011)[43]	UK	To explore FGM within the context of Birmingham.	Birmingham	Participatory Ethnographic Evaluation and Research (PEER)	In-depth semi-structured interviews with and by peers recruited using community organisations and snowball sampling	Participatory thematic analysis	Fifteen peer researchers conducted a total of 90 interviews<br><br>Age range 17 - 48 years	105 women<br><br>(15 peer researchers who were also interviewed, and 90 women) from Somali, Eritrea, Sudan, Sierra Leon and Gambia

No.	Authors and date	Country	Study aims, focus and/or research question	Region and Setting (where specified)	Methodology and/or theoretical perspective	Methods and recruitment (where stated)	Data analysis	Whole study sample	Eligible participants and country of origin
**44.**	Recchia, N., & McGarry, J. (2017)[44]	UK	The overall aim of the study was to develop and facilitate a participant led arts-based workshop with women who were survivors of FGM in order to explore their experiences and the impact of FGM on their health and well-being.	Nottingham<br><br>At a local FGM specialist support organisation	Creative narrative approach which included the sharing of personal stories, the creation of pottery models and the sharing of artefacts - arts-based research approach using a feminist theoretical framework	The composition of the workshop essentially encompassed two main strands: the creation of a persona and sharing artefacts. Recruited though a local FGM specialist support organisation	Narrative approach	Six women agreed to take part in the project<br><br>Age range 25-51 years	6 women from Kenya, Nigeria and Ethiopia
**45.**	Safari, F., (2013)[45]	UK	Explored women's experience of deinfibulation and its aftermath.	London<br><br>At NHS hospital, or participant's home	Qualitative approach Interpretive Phenomenological Analysis (IPA)	Semi-structured interview	Interpretive Phenomenologic al Analysis (IPA)	9 women<br><br>Age range 19-44 years	9 women from Somalia Eritrea
**46.**	Salad, J., Verdonk, P., de Boer, F., & Abma, T. A. (2015)[46]	Netherlands	To explore the perceptions of Somali women living in the Netherlands regarding measures to prevent cervical cancer	In cafés, libraries, schools, and community centres	Qualitative approach Intersectionality is therefore integrated in the HBM in this study	Semi-structured interviews, group discussions, recruited though Purposive sampling using Snowball sampling	Framework of intersectionality enabled a comprehensive thematic content analysis	Interviews with 14 young women (aged 17-21) and 6 older women (mothers aged 30-46)<br><br>Group discussions with 26 women (mothers aged 23-66)	46 Somali women

No.	Authors and date	Country	Study aims, focus and/or research question	Region and Setting (where specified)	Methodology and/or theoretical perspective	Methods and recruitment (where stated)	Data analysis	Whole study sample	Eligible participants and country of origin
**47.**	Shaw, E. (1985)[47]	USA	To 1) provide a summary of basic information about female circumcision; 2) summarize the results of an exploratory study which identified special needs and concerns of circumcised women who have used the western medical system while living in the United States, and 3) identify problems and concerns student health care providers have encountered while caring for this group of women.	Southwestern University town. Setting not stated.	Exploratory study	Informal interviews	Thematic analysis	12 circumcised women<br>Six Planned Parenthood Centres and four obstetric gynaecological clinics responded	12 circumcised women from Sudan, Egypt, and Somalia
**48.**	Shermarke, M.A.A. (1996)[48]	Canada	Explores female circumcision within the Canadian community perspective	Montreal, Quebec and Ottawa, Ontario.<br><br>In homes of the interviewees or in selected, quiet, public places	Case study and Qualitative methods	Individual interviews selected through personal contacts	Thematic analysis	4 interviewees from Montreal and 4 from Ottawa<br><br>Age range 16 - 58 years	8 Somali women
**49.**	Straus, L., McEwen, A., & Hussein, F. M. (2009)[49]	UK	To examine cultural and social aspects of childbirth, and to determine how they intersect with the needs and experiences of Somali women in the UK. To explore the experiences and needs of Somali women in the UK during pregnancy and childbirth.	London<br><br>In the community	Ethnographic approach narrative approach	In-depth narrative interviews<br><br>Recruitment from two community Somali women's groups and snowballing	Thematic analysis	8 Somali women<br><br>Age range 23-57 years	8 Somali women

No.	Authors and date	Country	Study aims, focus and/or research question	Region and Setting (where specified)	Methodology and/or theoretical perspective	Methods and recruitment (where stated)	Data analysis	Whole study sample	Eligible participants and country of origin
**50.**	Thierfelder, C. (2003)[50]	Switzerland	To analyse the specific situation of genitally mutilated African immigrant women, and the health care providers who encounter them in the Swiss health care system.	Cantons of Geneva, Vaud and Zurich.<br><br>Setting not stated	Qualitative research.	Semi-structured, in-depth interviews and focus group discussions.<br><br>Utilised purposive sampling and snowball sampling drawing on contacts with migrant-support and community organisations.	Thematic content analysis	Women (n=29) and men (n=3) (n=37) with Swiss health care providers (20 physicians and 17 midwives)<br><br>Age range 17-64 years	29 women from Somalia and Eritrea
**51.**	Thierfelder, C., Tanner, M., & Bodiang, C. M. K. (2005)[51]	Switzerland	To analyse how immigrant women with FGM experience gynaecological/obstetrical care in the Swiss health care system, and to investigate if physicians and midwives treat and counsel FGM related complications adequately.	Zurich, Lausanne and Geneva, Switzerland. Setting not stated.	Qualitative methods.	Four focus group discussions and interviews.<br><br>Purposive sampling via migrant-support and community organisations.	Unclear	29 women<br>37 health care professionals	29 women from Somalia and Eritrea
**52.**	Upvall, M. J., Mohammed, K., & Dodge, P. D. (2009)[52]	USA	To explore the healthcare perspectives of Somali Bantu refugees in relation to their status as women who have been circumcised and recently resettled in the US.	South-western Pennsylvania, at a local refugee centre	Qualitative study	Focus groups purposive sample identified by a local non-profit organization	Thematic analysis	23 resettled Somali women, a local female physician<br><br>Age range 19-43 years	23 resettled Somali women
**53.**	Vangen, S., Johansen, R. E. B., Sundby, J., Traeen, B., & Stray-Pedersen, B. (2004)[53]	Norway	To explore how perinatal care practice may influence labour outcomes among circumcised women	At women's homes or public cafes, or hospital	Qualitative method drawing on theories of empowerment	In-depth interviews. Recruited from Somali's association and in health facilities and through snowball sampling	Thematic analysis	23 Somali immigrants and 36 Norwegian health care professionals;<br><br>Age range 18-55 years	23 Somali women

No.	Authors and date	Country	Study aims, focus and/or research question	Region and Setting (where specified)	Methodology and/or theoretical perspective	Methods and recruitment (where stated)	Data analysis	Whole study sample	Eligible participants and country of origin
**54.**	Vaughan, C., White, N., Keogh, L., Tobin, J., Ha, B., Ibrahim, M., & Bayly, C. (2014)[54]	Australia	To improve understanding of the impacts of FGC and to make suggestions for service development - by engaging with local women, communities and health providers	North Yarra Carlton, Collingwood and Fitzroy<br><br>Research conducted in community centre meeting rooms.	Community based qualitative approach	8 focus group discussions, 4 small group discussions, 10 individual interviews with community members. Recruitment from community consultations, community organisations and networks<br><br>11 interviews with service providers	Thematic analysis	123 individuals<br><br>112 migrant men and women and 11 health providers	87 migrant women from Eritrea, Ethiopia, Somali and Sudan
**55.**	Vaughan, C., White, N., Keogh, L., Tobin, J., Murdolo, A., Quiazon, R., & Bayly, C. (2014)[55]	Australia	To identify the FGC-related service needs prioritised by affected communities living in Victorian regional centres, and to build evidence as to the training, education and professional development required for service providers working in regional Victoria to be able to meet these needs.	Australia Regional Victoria Ballarat, Geelong, Latrobe Valley and Shepparton,<br><br>Research conducted in community meeting rooms	Community based qualitative approach	Focus group discussions.<br><br>Recruitment from migrant support agencies and then using snowball sampling	Thematic analysis	51 migrant community members 950 women, 1 man)<br><br>15 health service providers	50 women from Togo Sudan and Kenya
**56.**	Vloeberghs, E., van der Kwaak, A., Knipscheer, J., & van den Muijsenberg h, M. (2012)[56]	Netherlands	Explored psychosocial and relational problems of African immigrant women in the Netherlands who underwent female genital mutilation/cutting (FGM/C), the causes they attribute to these problems and the way they cope with these health complaints.	In participants' homes	Mixed-methods study used standardised questionnaires as well as in-depth interviews, and qualitative participatory methods Grounded Theory	In-depth interviews using peer researchers. Snowball sampling used	Thematic framework analysis	66 women;<br><br>Age range 18~69	66 women from Somalia, Sudan, Eritrea, Ethiopia or Sierra Leone

For peer review

No.	Authors and date	Country	Study aims, focus and/or research question	Region and Setting (where specified)	Methodology and/or theoretical perspective	Methods and recruitment (where stated)	Data analysis	Whole study sample	Eligible participants and country of origin
**57.**	Wiklund, H., Aden, A. S., Högberg, U., Wikman, M., & Dahlgren, L. (2000)[57]	Sweden	To study the childbirth experiences of Somali women and men in Sweden.	Umea and nearby municipalities; in women's homes	Grounded theory	Interviews were semi-structured. Recruited from the Swedish Medical Birth Register and `patient' records	Grounded theory analysis	9 women and 4 husbands of the women studied and additional 3 men; 21-55 years	9 Somali women

For peer review only

References of Included Studies

1. Abdi R. Carving culture: Creating identity through female genital cutting. *Durham Anthropology Journal* 2012;18(1):115-53.

2. Abdullahi A, Copping J, Kessel A, et al. Cervical screening: perceptions and barriers to uptake among Somali women in Camden. *Public Health* 2009;123(10):680-5. doi: 10.1016/j.puhe.2009.09.011

3. Ahlberg BM, Krantz I, Lindmark G, et al. 'It's only a tradition': making sense of eradication interventions and the persistence of female 'circumcision' within a Swedish context. *Crit Soc Policy* 2004;24(1):50-78. doi: 10.1107/0261018304039679

4. Ahmed M. Attitudes towards FGM among Somali women living in the UK. In: Momoh C, ed. Female Genital Mutilation. London: Radcliffe 2005:95-117.

5. Ameresekere M, Borg R, Frederick J, et al. Somali immigrant women's perceptions of cesarean delivery and patient-provider communication surrounding female circumcision and childbirth in the USA. *Int J Gynecol Obstet* 2011;115(3):227-30. doi: 10.1016/j.ijgo.2011.07.019

6. Asefaw F. [Female Genital Mutilation: A Field Study with Special Consideration of the Background as well as the Health and Psychosexual Consquences for those Affected and their Partners in Eritrea and in Germany] Weibliche Genitalbeschneidung: Eine Feldstudie Unter besonderer Berücksichtigung der Hintergründe Sowie der Gesundheitlichen und Psychosexuellen Folgen für Betroffene und Partner in Eritrea und Deutschland. Humboldt University of Berlin, 2007.

7. Baldeh F. Obstetric Care in Scotland: The Experience of Women who have Undergone Female Genital Mutilation (FGM) [MSc Thesis]. Queen Margaret University, 2013.

8. Ballesteros Meseguer C, Almansa Martinez P, Pastor Bravo MdM, et al. [The voice of women subjected to female genital mutilation in the region of Murcia, Spain] La Voz de las mujeres sometidas a mutilacion genital femenina en la region de Murcia. *Gac Sanit* 2014;28(4):287-91. doi: 10.1016/j.gaceta.2014.02.006

9. Behrendt A. Listening to African Voices: Female Genital Mutilation/Cutting among Immigrants in Hamburg: Knowledge, Attitudes and Practice. Hamburg, Germany: Plan, 2011.

10. Beine K, Fullerton J, Palinkas L, et al. Conceptions of prenatal care among Somali women in San Diego. *J Nurs Midwifery* 1995;40(4):376-81. doi: Doi 10.1016/0091-2182(95)00024-E

11. Berggren V, Bergstrom S, Edberg A-K. Being different and vulnerable: experiences of immigrant African women who have been circumcised and sought maternity care in Sweden. *J Transcult Nurs* 2006;17(1):50-7. doi: 10.1177/1043659605281981

12. Betts V. Transnational Gender, Sexuality and Identity Construction: Ambivalent Subjectivities of Somali Refugee Women in the GTA. MA Thesis, The University of Guelph, 2011.

For peer review only

13. Bravo Pastor del Mar M. [Women Subjected to Female Genital Mutilation: Knowledge for the Nursing Discipline] La Voz de las Mujeres Sometidas a Mutilación Genital Femenina: Saberes Para la Disciplina Enfermera [Tesis Doctoral]. University of Murcia, 2014.

14. Bulman KH, McCourt C. Somali refugee women's experiences of maternity care in west London: a case study. *Crit Public Health* 2002;12(4):365-80.

15. Carroll J, Epstein R, Fiscella K, et al. Caring for Somali women: implications for clinician-patient communication. *Patient Educ Couns* 2007;66(3):337-45. doi: 10.1016/j.pec.2007.01.008

16. Chalmers B, Omer-Hashi K. What Somali women say about giving birth in Canada. *J Reprod Infant Psychol* 2002;20(4):267-82. doi: 10.1080/0264683021000033183

17. Degni F, Suominen SB, El Ansari W, et al. Reproductive and maternity health care services in Finland: perceptions and experiences of Somali-born immigrant women. *Ethn Health* 2014;19(3):348-66. doi: 10.1080/13557858.2013.797567

18. d'Entremont M, Smythe L, McAra-Couper J. The sounds of silence: a hermeneutic interpretation of childbirth post excision. *Health Care Women Int* 2014;35(3):300-19. doi: 10.1080/07399332.2013.838245

19. Essén B, Johnsdotter S, Hovelius B, et al. Qualitative study of pregnancy and childbirth experiences in Somalian women resident in Sweden. *Br J Obstet Gynaecol* 2000;107(12):1507-12.

20. Fawcett L. Somali Refugee Women and their U.S. Healthcare Providers: Knowledge, Perceptions and Experiences of Childbearing [Doctor of Philosophy]. Arizona State University, 2014.

21. Gali MA. Female Circumcision: A Transcultural Study of Attitudes, Identity and Reproductive Health of East African Immigrants [Degree of Doctor of Philosophy in Psychology]. The Wright Institute, University of Berkeley, 1997.

22. Ghebre RG, Sewali B, Osman S, et al. Cervical cancer: barriers to screening in the Somali community in Minnesota. *J Immigr Minor Health* 2015;17(3):722-28. doi: 10.1007/s10903-014-0080-1

23. Glazer E. Embodiment, Pain and Circumcision in Somali-Canadian Women [M.Sc.]. University of Toronto, 2012.

24. Glover J, Liebling H, Barrett H, et al. The psychological and social impact of female genital mutilation: a holistic conceptual framework. *J Int Stud* 2017;10(2):219-38.

25. Guerin PB, Allotey P, Elmi FH, et al. Advocacy as a means to an end: assisting refugee women to take control of their reproductive health needs. *Women Health* 2006;43(4):7-25.

26. Hill N, Hunt E, Hyrkas K. Somali immigrant women's health care experiences and beliefs regarding pregnancy and birth in the United States. *J Transcult Nurs* 2012;23(1):72-81. doi: 10.1177/1043659611423828 [published Online First: 2011/11/05]

For peer review only

27. Hussein E. Women's Experiences, Perceptions and Attitudes of Female Genital Mutilation: The Bristol PEER Study. London: FORWARD, 2010.

28. Hussen MA. Services for Women with Female Genital Mutilation in Christchurch: Perspectives of Women and their Health Providers. University of Canterbury, 2014.

29. Johansen RE. Undoing female genital cutting: perceptions and experiences of infibulation, defibulation and virginity among Somali and Sudanese migrants in Norway. *Cult Health Sex* 2017;19(4):528-42. doi: 10.1080/13691058.2016.1239838

30. Jones A. Working Psychologically with Female Genital Mutilation: An Exploration of the Views and Experiences of Women who have Experienced FGM and of Clinical Psychologists. University of East London, 2010.

31. Khaja K. Female Circumcision: Life Histories of Somali Women [Doctor of Philosophy]. The University of Utah, 2004.

32. Khaja K, Lay K, Boys S. Female circumcision: toward an inclusive practice of care. *Health Care Women Int* 2010;31(8):686-99. doi: 10.1080/07399332.2010.490313

33. Lundberg PC, Gerezgiher A. Experiences from pregnancy and childbirth related to female genital mutilation among Eritrean immigrant women in Sweden. *Midwifery* 2008;24(2):214-25. doi: 10.1016/j.midw.2006.10.003

34. Maier C. [Echoes of Silence: Voices of Concern for Genital Mutilation in African Immigrant Women in Vienna: An Ethnological Study] Echo des Schweigens: Stimmen der Betroffenheit zur Genitalverstümmelung bei Afrikanischen Immigrantinnen in Wien: Ethnologische Studie. University of Vienna, 2003.

35. Maternity Action. Women's Voices on Health: Addressing Barriers to Accessing Primary Care. London: Women’s Health and Equality Consortium, 2014.

36. McNeely S, Christie-de Jong F. Somali refugees' perspectives regarding FGM/C in the US. *Int J Migr Health Soc Care* 2016;12(3):157-69. doi: 10.1108/Ijmhsc-09-2015-0033

37. Moxey JM, Jones LL. A qualitative study exploring how Somali women exposed to female genital mutilation experience and perceive antenatal and intrapartum care in England. *BMJ Open* 2016;6(1):e009846. doi: 10.1136/bmjopen-2015-009846

38. Murray L, Windsor C, Parker E, et al. The experiences of African women giving birth in Brisbane, Australia. *Health Care Women Int* 2010;31(5):458-72. doi: 10.1080/07399330903548928

39. Norman K, Gegzabher SB, Otoo-Oyortey N. "Between Two Cultures": A Rapid PEER Study Exploring Migrant Communities' Views on Female Genital Mutilation in Essex and Norfolk, UK. FORWARD & National FGM Centre Report, , 2016.

40. Norman K, Hemmings J, Hussein E, et al. "FGM is Always With Us": Experiences, Perceptions and Beliefs of Women Affected by Female Genital Mutilation in London: Results from a PEER Study. London: Options Consultancy Services and FORWARD, 2009.

For peer review only

41. O'Brien O, Baldeh F, Hassan J, et al. My Voice: Participatory Action Research Project with Men, Women and Young People on Female Genital Mutilation (FGM) in Scotland: (Phase 2). Waverly Care, Edinburgh: Queen Margaret University, 2017.

42. O'Brien O, Baldeh F, Sivapatham S, et al. Participatory Action Research Project with Men, Women and Young People on Female Genital Mutilation (FGM) in Scotland: (Phase 1). Waverly Care, Edinburgh: Queen Margaret University, 2016.

43. Palfreyman A, Brown E, Nam S. Understanding Female Genital Mutilation in Birmingham: Findings from a PEER Study: Options Consultancy Services and Birmingham & Solihull Women's Aid, 2011.

44. Recchia N, McGarry J. "Don't judge me": narratives of living with FGM. *Int J Hum Rights Healthc* 2017;10(1):4-13. doi: 10.1108/Ijhrh-10-2016-0016

45. Safari F. A qualitative study of women's lived experience after deinfibulation in the UK. *Midwifery* 2013;29(2):154-8. doi: 10.1016/j.midw.2011.12.005

46. Salad J, Verdonk P, de Boer F, et al. "A Somali girl is Muslim and does not have premarital sex - is vaccination really necessary?" A qualitative study into the perceptions of Somali women in the Netherlands about the prevention of cervical cancer. *Int J Equity Health* 2015;14(1):1-13. doi: 10.1186/s12939-015-0198-3

47. Shaw E. Female circumcision: perceptions of clients and caregivers. *J Am Coll Health* 1985;33(5):193-7. doi: 10.1080/07448481.1985.9939604

48. Shermarke MAA. Understanding the Canadian Community Context of Female Circumcision [M.S.W.]. McGill University, 1996.

49. Straus L, McEwen A, Hussein FM. Somali women's experience of childbirth in the UK: perspectives from Somali health workers. *Midwifery* 2009;25(2):181-6. doi: 10.1016/j.midw.2007.02.002

50. Thierfelder C. Female Genital Mutilation and the Swiss Health Care System. University of Basel, 2003.

51. Thierfelder C, Tanner M, Bodiang CMK. Female genital mutilation in the context of migration: experience of African women with the Swiss health care system. *Eur J Public Health* 2005;15(1):86-90. doi: 10.1093/eurpub/cki120

52. Upvall MJ, Mohammed K, Dodge PD. Perspectives of Somali Bantu refugee women living with circumcision in the United States: a focus group approach. *Int J Nurs Stud* 2009;46(3):360-8. doi: 10.1016/j.ijnurstu.2008.04.009

53. Vangen S, Johansen REB, Sundby J, et al. Qualitative study of perinatal care experiences among Somali women and local health care professionals in Norway. *Eur J Obstet Gynecol Reprod Biol* 2004;112(1):29-35. doi: 10.1016/S0301-2115(03)00313-0

54. Vaughan C, White N, Keogh L, et al. Listening to North Yarra Communities about Female Genital Cutting. Melbourne, Australia: The University of Melbourne, 2014.

For peer review only

55. Vaughan C, White N, Keogh L, et al. Female Genital Mutilation/Cutting in Regional Victoria. Research to Practice. Melbourne, Australia: The University of Melbourne, 2014.

56. Vloeberghs E, van der Kwaak A, Knipscheer J, et al. Coping and chronic psychosocial consequences of female genital mutilation in the Netherlands. *Ethn Health* 2012;17(6):677-95. doi: 10.1080/13557858.2013.771148

57. Wiklund H, Aden AS, Högberg U, et al. Somalis giving birth in Sweden: a challenge to culture and gender specific values and behaviours. *Midwifery* 2000;16(2):105-15. doi: http://dx.doi.org/10.1054/midw.1999.0197

For peer review only

Supplementary File 6: Full Quality Assessment

No	Reference	Q1	Q2	Q3	Q4	Q5	Q6	Q7	Q8	Q9	Q10	Score	Quality Band	Richness: Thick or Thin	Type of Publication	Relevance: High, Med or Low
**1.**	Abdi, R. (2012)[1]	Y	Y	Y	N	Y	Y	Y	N	Y	Y	**8**	High	Thin	Journal	Low
**2.**	Abdullahi, A., Copping, J., Kessel, A., Luck, M., & Bonell, C. (2009)[2]	N	U	Y	Y	Y	N	N	Y	Y	Y	**6**	Med	Thin	Journal	Med
**3.**	Ahlberg, B. M., Krantz, I., Lindmark, G., & Warsame, M. (2004)[3]	U	Y	Y	Y	Y	Y	Y	Y	Y	Y	**9**	High	Thin	Journal	Low
**4.**	Ahmed, M., (2005)[4]	N	N	U	U	Y	N	N	P	Y	Y	**3.5**	Low	Thin	Book chapter	Low
**5.**	Ameresekere, M., Borg, R., Frederick, J., Vragovic, O., Saia, K., & Raj, A. (2011)[5]	U	Y	Y	Y	Y	N	N	P	Y	Y	**6.5**	Med	Thin	Journal	Med
**6.**	Asefaw, F. (2007)[6]	N	U	U	N	Y	Y	Y	Y	Y	Y	**6**	Med	Thin	PhD Thesis	Low
**7.**	Baldeh, F. (2013)[7]	U	Y	Y	Y	Y	Y	P	Y	Y	Y	**8.5**	High	Thick	MSc Thesis	High
**8.**	Ballesteros Meseguer, C., Almansa Martinez, P., Pastor Bravo, M. d. M., & Jimenez Ruiz, I. (2014)[8]	Y	Y	Y	N	Y	N	N	P	Y	Y	**6.5**	Med	Thin	Journal	Med
**9.**	Behrendt, A. (2011)[9]	U	Y	Y	Y	U	N	N	P	Y	Y	**5.5**	Med	Thin	Research Report	Low
**10.**	Beine, K., Fullerton, J., Palinkas, L., & Anders, B. (1995)[10]	N	U	U	Y	Y	P	N	Y	Y	Y	**5.5**	Med	Thin	Journal	Med
**11.**	Berggren, V., Bergstrom, S., & Edberg, A.-K. (2006)[11]	N	U	Y	Y	Y	N	N	Y	Y	Y	**6**	Med	Thick	Journal	High
**12.**	Betts, V. (2011)[12]	U	U	U	N	Y	Y	P	Y	Y	Y	**5.5**	Med	Thick	MA Thesis	High
**13.**	Bravo Pastor del Mar, M. (2014)[13]	Y	Y	Y	Y	Y	Y	N	P	Y	U	**7.5**	High	Thin	PhD Thesis	Low
**14.**	Bulman, K. H., & McCourt, C. (2002)[14]	N	N	Y	Y	P	P	Y	P	Y	U	**5.5**	Med	Thin	Journal	Med
**15.**	Carroll, J., Epstein, R., Fiscella, K., Gipson, T., Volpe, E., & Jean-Pierre, P. (2007)[15]	Y	Y	Y	P	Y	N	P	Y	Y	Y	**8**	High	Thick	Journal	Med
**16.**	Chalmers, B., & Omer-Hashi, K. (2002)[16]	N	U	U	U	Y	P	P	Y	Y	Y	**5**	Med	Thick	Journal	Med

For peer review only

No	Reference	Q1	Q2	Q3	Q4	Q5	Q6	Q7	Q8	Q9	Q10	Score	Quality Band	Richness: Thick or Thin	Type of Publication	Relevance: High, Med or Low
**17.**	Degni, F., Suominen, S. B., El Ansari, W., Vehviläinen-Julkunen, K., & Essen, B. (2014)[17]	U	Y	Y	Y	Y	P	P	Y	Y	Y	**8**	High	Thin	Journal	Med
**18.**	d'Entremont, M., Smythe, L., & McAra-Couper, J. (2014)[18]	Y	Y	Y	Y	Y	U	P	Y	Y	Y	**8.5**	High	Thick	Journal	High
**19.**	Essén, B., Johnsdotter, S., Hovelius, B., Gudmundsson, S., Sjöberg, N. O., Friedman, J., & Östergren, P. O. (2000)[19]	U	U	U	N	Y	Y	P	P	Y	Y	**5**	Med	Thin	Journal	Low
**20.**	Fawcett, L., (2014)[20]	Y	Y	Y	U	Y	Y	Y	Y	Y	Y	**9**	High	Thick	PhD Thesis	High
**21.**	Gali, M. A. (1997)[21]	U	U	U	U	Y	Y	N	Y	Y	Y	**5**	Med	Thin	PhD Thesis	Low
**22.**	Ghebre, R. G., Sewali, B., Osman, S., Adawe, A., Nguyen, H. T., Okuyemi, K. S., & Joseph, A. (2015)[22]	Y	Y	Y	Y	Y	N	N	P	Y	Y	**7.5**	High	Thick	Journal	Med
**23.**	Glazer, E., (2012)[23]	Y	Y	Y	Y	Y	P	N	Y	Y	Y	**8.5**	High	Thin	MSc Thesis	Low
**24.**	Glover, J., Liebling, H., Barrett, H., & Goodman, S. (2017)[24]	Y	Y	Y	Y	Y	N	N	Y	Y	Y	**8**	High	Thick	Journal	High
**25.**	Guerin, P. B., Allotey, P., Elmi, F. H., & Baho, S. (2006)[25]	U	U	U	Y	P	N	P	N	Y	Y	**4**	Low	Thin	Journal	Low
**26.**	Hill, N., Hunt, E., & Hyrkas, K. (2012)[26]	N	U	U	Y	Y	Y	N	Y	Y	Y	**6**	Med	Thick	Journal	Low
**27.**	Hussein, E., (2010)[27]	Y	Y	Y	N	Y	N	P	Y	N	Y	**6.5**	Med	Thin	Research Report	Low
**28.**	Hussen, M. A. (2014)[28]	N	U	Y	Y	Y	Y	P	Y	U	Y	**6.5**	Med	Thick	MSc Thesis	Med
**29.**	Johansen, R.E. (2017)[29]	U	U	Y	Y	Y	Y	P	Y	Y	Y	**7.5**	High	Thick	Journal	Med
**30.**	Jones, A. (2010)[30]	Y	Y	Y	Y	N	P	P	Y	Y	Y	**8**	High	Thick	PhD Thesis	High
**31.**	Khaja, K. (2004)[31]	U	Y	Y	Y	Y	P	N	Y	Y	Y	**7.5**	High	Thin	PhD Thesis	Low
**32.**	Khaja, K., Lay, K., & Boys, S. (2010)[32]	N	U	Y	N	U	N	N	P	N	Y	**2.5**	Low	Thin	Journal	Low
**33.**	Lundberg, P. C., & Gerezgiher, A. (2008)[33]	U	Y	Y	Y	Y	P	P	Y	Y	Y	**8**	High	Thick	Journal	High
**34.**	Maier, C. (2003)[34]	N	N	N	N	Y	Y	N	Y	U	Y	**4**	Low	Thin	PhD Thesis	Low

No	Reference	Q1	Q2	Q3	Q4	Q5	Q6	Q7	Q8	Q9	Q10	Score	Quality Band	Richness: Thick or Thin	Type of Publication	Relevance: High, Med or Low
**35.**	Maternity Action. (2014)[35]	U	U	Y	U	Y	N	P	Y	U	U	**3.5**	Low	Thin	Research Report	Med
**36.**	McNeely, S., & Christie-de Jong, F. (2016)[36]	U	Y	Y	Y	Y	Y	P	N	Y	Y	**7.5**	High	Thin	Journal	Low
**37.**	Moxey, J. M., & Jones, L. L. (2016)[37]	U	Y	Y	Y	Y	Y	P	Y	Y	Y	**8.5**	High	Thick	Journal	High
**38.**	Murray, L., Windsor, C., Parker, E., & Tewfik, O. (2010)[38]	Y	Y	Y	Y	Y	Y	P	Y	Y	Y	**9.5**	High	Thick	Journal	Med
**39.**	Norman, K., Gegzabher, S. B., & Otoo-Oyortey, N. (2016)[39]	Y	Y	Y	U	U	P	P	Y	N	Y	**6**	Med	Thin	Research Report	Low
**40.**	Norman, K., Hemmings, J., Hussein, E., & Otoo-Oyortey, N. (2009)[40]	Y	Y	Y	U	U	P	P	Y	N	Y	**6**	Med	Thin	Research Report	Low
**41.**	O'Brien, O., Baldeh, F., Hassan, J., & Baillie, M. (2017)[41]	Y	Y	Y	U	Y	P	P	Y	Y	Y	**8**	High	Thin	Research Report	Low
**42.**	O'Brien, O., Baldeh, F., Sivapatham, S., Brown, E., & O'May, F. (2016)[42]	Y	Y	Y	N	Y	P	P	Y	Y	Y	**8**	High	Thin	Research Report	Low
**43.**	Palfreyman, A., Brown, E., & Nam, S. (2011)[43]	N	Y	Y	Y	Y	P	P	Y	Y	Y	**8**	High	Thick	Research Report	High
**44.**	Recchia, N., & McGarry, J. (2017)[44]	U	Y	Y	U	Y	N	N	Y	U	Y	**5**	Med	Thick	Journal	Low
**45.**	Safari, F., (2013)[45]	Y	Y	Y	Y	Y	N	P	Y	Y	Y	**8.5**	High	Thin	Journal	Low
**46.**	Salad, J., Verdonk, P., de Boer, F., & Abma, T. A. (2015)[46]	U	U	Y	Y	Y	Y	P	Y	Y	Y	**7.5**	High	Thick	Journal	Med
**47.**	Shaw, E. (1985)[47]	N	U	U	Y	Y	N	P	Y	U	P	**4**	Low	Thin	Journal	Low
**48.**	Shermarke, M.A.A. (1996)[48]	Y	Y	Y	Y	Y	Y	Y	Y	U	Y	**9**	High	Thin	MSc Thesis	Med
**49.**	Straus, L., McEwen, A., & Hussein, F. M. (2009)[49]	U	Y	Y	Y	Y	Y	P	Y	Y	Y	**8.5**	High	Thick	Journal	High
**50.**	Thierfelder, C. (2003)[50]	N	N	Y	Y	Y	Y	P	Y	Y	Y	**7.5**	High	Thick	PhD Thesis	High
**51.**	Thierfelder, C., Tanner, M., & Bodiang, C. M. K. (2005)[51]	N	N	Y	U	Y	P	P	Y	Y	Y	**6**	Med	Thin	Journal	Med
**52.**	Upvall, M. J., Mohammed, K., & Dodge, P. D. (2009)[52]	N	N	Y	Y	Y	P	P	Y	Y	Y	**7**	Med	Thick	Journal	High
**53.**	Vangen, S., Johansen, R. E. B., Sundby, J., Traeen, B., & Stray-Pedersen, B. (2004)[53]	Y	Y	Y	Y	Y	P	N	P	Y	Y	**8**	High	Thick	Journal	High

For peer review only - http://bmjopen.bmj.com/site/about/guidelines.xhtml

No	Reference	Q1	Q2	Q3	Q4	Q5	Q6	Q7	Q8	Q9	Q10	Score	Quality Band	Richness: Thick or Thin	Type of Publication	Relevance: High, Med or Low
**54.**	Vaughan, C., White, N., Keogh, L., Tobin, J., Ha, B., Ibrahim, M., & Bayly, C. (2014)[54]	U	Y	Y	Y	Y	Y	P	Y	Y	Y	**8.5**	High	Thick	Research Report	High
**55.**	Vaughan, C., White, N., Keogh, L., Tobin, J., Murdolo, A., Quiazon, R., & Bayly, C. (2014)[55]	U	Y	Y	Y	Y	Y	P	Y	Y	Y	**8.5**	High	Thick	Research Report	High
**56.**	Vloeberghs, E., van der Kwaak, A., Knipscheer, J., & van den Muijsenbergh, M. (2012)[56]	Y	Y	Y	Y	Y	P	P	Y	Y	Y	**9**	High	Thin	Journal	Med
**57.**	Wiklund, H., Aden, A. S., Högberg, U., Wikman, M., & Dahlgren, L. (2000)[57]	Y	Y	U	Y	Y	N	N	Y	Y	Y	**7**	Med	Thin	Journal	Med

For peer review only

References of Studies Included in Quality Assessment

1. Abdi R. Carving culture: Creating identity through female genital cutting. *Durham Anthropology Journal* 2012;18(1):115-53.

2. Abdullahi A, Copping J, Kessel A, et al. Cervical screening: perceptions and barriers to uptake among Somali women in Camden. *Public Health* 2009;123(10):680-5. doi: 10.1016/j.puhe.2009.09.011

3. Ahlberg BM, Krantz I, Lindmark G, et al. 'It's only a tradition': making sense of eradication interventions and the persistence of female 'circumcision' within a Swedish context. *Crit Soc Policy* 2004;24(1):50-78. doi: 10.1107/0261018304039679

4. Ahmed M. Attitudes towards FGM among Somali women living in the UK. In: Momoh C, ed. Female Genital Mutilation. London: Radcliffe 2005:95-117.

5. Ameresekere M, Borg R, Frederick J, et al. Somali immigrant women's perceptions of cesarean delivery and patient-provider communication surrounding female circumcision and childbirth in the USA. *Int J Gynecol Obstet* 2011;115(3):227-30. doi: 10.1016/j.ijgo.2011.07.019

6. Asefaw F. [Female Genital Mutilation: A Field Study with Special Consideration of the Background as well as the Health and Psychosexual Consquences for those Affected and their Partners in Eritrea and in Germany] Weibliche Genitalbeschneidung: Eine Feldstudie Unter besonderer Berücksichtigung der Hintergründe Sowie der Gesundheitlichen und Psychosexuellen Folgen für Betroffene und Partner in Eritrea und Deutschland. Humboldt University of Berlin, 2007.

7. Baldeh F. Obstetric Care in Scotland: The Experience of Women who have Undergone Female Genital Mutilation (FGM) [MSc Thesis]. Queen Margaret University, 2013.

8. Ballesteros Meseguer C, Almansa Martinez P, Pastor Bravo MdM, et al. [The voice of women subjected to female genital mutilation in the region of Murcia, Spain] La Voz de las mujeres sometidas a mutilacion genital femenina en la region de Murcia. *Gac Sanit* 2014;28(4):287-91. doi: 10.1016/j.gaceta.2014.02.006

9. Behrendt A. Listening to African Voices: Female Genital Mutilation/Cutting among Immigrants in Hamburg: Knowledge, Attitudes and Practice. Hamburg, Germany: Plan, 2011.

10. Beine K, Fullerton J, Palinkas L, et al. Conceptions of prenatal care among Somali women in San Diego. *J Nurs Midwifery* 1995;40(4):376-81. doi: Doi 10.1016/0091-2182(95)00024-E

11. Berggren V, Bergstrom S, Edberg A-K. Being different and vulnerable: experiences of immigrant African women who have been circumcised and sought maternity care in Sweden. *J Transcult Nurs* 2006;17(1):50-7. doi: 10.1177/1043659605281981

For peer review only

12. Betts V. Transnational Gender, Sexuality and Identity Construction: Ambivalent Subjectivities of Somali Refugee Women in the GTA. MA Thesis, The University of Guelph, 2011.

13. Bravo Pastor del Mar M. [Women Subjected to Female Genital Mutilation: Knowledge for the Nursing Discipline] La Voz de las Mujeres Sometidas a Mutilación Genital Femenina: Saberes Para la Disciplina Enfermera [Tesis Doctoral]. University of Murcia, 2014.

14. Bulman KH, McCourt C. Somali refugee women's experiences of maternity care in west London: a case study. *Crit Public Health* 2002;12(4):365-80.

15. Carroll J, Epstein R, Fiscella K, et al. Caring for Somali women: implications for clinician-patient communication. *Patient Educ Couns* 2007;66(3):337-45. doi: 10.1016/j.pec.2007.01.008

16. Chalmers B, Omer-Hashi K. What Somali women say about giving birth in Canada. *J Reprod Infant Psychol* 2002;20(4):267-82. doi: 10.1080/0264683021000033183

17. Degni F, Suominen SB, El Ansari W, et al. Reproductive and maternity health care services in Finland: perceptions and experiences of Somali-born immigrant women. *Ethn Health* 2014;19(3):348-66. doi: 10.1080/13557858.2013.797567

18. d'Entremont M, Smythe L, McAra-Couper J. The sounds of silence: a hermeneutic interpretation of childbirth post excision. *Health Care Women Int* 2014;35(3):300-19. doi: 10.1080/07399332.2013.838245

19. Essén B, Johnsdotter S, Hovelius B, et al. Qualitative study of pregnancy and childbirth experiences in Somalian women resident in Sweden. *Br J Obstet Gynaecol* 2000;107(12):1507-12.

20. Fawcett L. Somali Refugee Women and their U.S. Healthcare Providers: Knowledge, Perceptions and Experiences of Childbearing [Doctor of Philosophy]. Arizona State University, 2014.

21. Gali MA. Female Circumcision: A Transcultural Study of Attitudes, Identity and Reproductive Health of East African Immigrants [Degree of Doctor of Philosophy in Psychology]. The Wright Institute, University of Berkeley, 1997.

22. Ghebre RG, Sewali B, Osman S, et al. Cervical cancer: barriers to screening in the Somali community in Minnesota. *J Immigr Minor Health* 2015;17(3):722-28. doi: 10.1007/s10903-014-0080-1

23. Glazer E. Embodiment, Pain and Circumcision in Somali-Canadian Women [M.Sc.]. University of Toronto, 2012.

24. Glover J, Liebling H, Barrett H, et al. The psychological and social impact of female genital mutilation: a holistic conceptual framework. *J Int Stud* 2017;10(2):219-38.

25. Guerin PB, Allotey P, Elmi FH, et al. Advocacy as a means to an end: assisting refugee women to take control of their reproductive health needs. *Women Health* 2006;43(4):7-25.

For peer review only

26. Hill N, Hunt E, Hyrkas K. Somali immigrant women's health care experiences and beliefs regarding pregnancy and birth in the United States. *J Transcult Nurs* 2012;23(1):72-81. doi: 10.1177/1043659611423828 [published Online First: 2011/11/05]

27. Hussein E. Women's Experiences, Perceptions and Attitudes of Female Genital Mutilation: The Bristol PEER Study. London: FORWARD, 2010.

28. Hussen MA. Services for Women with Female Genital Mutilation in Christchurch: Perspectives of Women and their Health Providers. University of Canterbury, 2014.

29. Johansen RE. Undoing female genital cutting: perceptions and experiences of infibulation, defibulation and virginity among Somali and Sudanese migrants in Norway. *Cult Health Sex* 2017;19(4):528-42. doi: 10.1080/13691058.2016.1239838

30. Jones A. Working Psychologically with Female Genital Mutilation: An Exploration of the Views and Experiences of Women who have Experienced FGM and of Clinical Psychologists. University of East London, 2010.

31. Khaja K. Female Circumcision: Life Histories of Somali Women [Doctor of Philosophy]. The University of Utah, 2004.

32. Khaja K, Lay K, Boys S. Female circumcision: toward an inclusive practice of care. *Health Care Women Int* 2010;31(8):686-99. doi: 10.1080/07399332.2010.490313

33. Lundberg PC, Gerezgiher A. Experiences from pregnancy and childbirth related to female genital mutilation among Eritrean immigrant women in Sweden. *Midwifery* 2008;24(2):214-25. doi: 10.1016/j.midw.2006.10.003

34. Maier C. [Echoes of Silence: Voices of Concern for Genital Mutilation in African Immigrant Women in Vienna: An Ethnological Study] Echo des Schweigens: Stimmen der Betroffenheit zur Genitalverstümmelung bei Afrikanischen Immigrantinnen in Wien: Ethnologische Studie. University of Vienna, 2003.

35. Maternity Action. Women's Voices on Health: Addressing Barriers to Accessing Primary Care. London: Women’s Health and Equality Consortium, 2014.

36. McNeely S, Christie-de Jong F. Somali refugees' perspectives regarding FGM/C in the US. *Int J Migr Health Soc Care* 2016;12(3):157-69. doi: 10.1108/Ijmhsc-09-2015-0033

37. Moxey JM, Jones LL. A qualitative study exploring how Somali women exposed to female genital mutilation experience and perceive antenatal and intrapartum care in England. *BMJ Open* 2016;6(1):e009846. doi: 10.1136/bmjopen-2015-009846

38. Murray L, Windsor C, Parker E, et al. The experiences of African women giving birth in Brisbane, Australia. *Health Care Women Int* 2010;31(5):458-72. doi: 10.1080/07399330903548928

39. Norman K, Gegzabher SB, Otoo-Oyortey N. "Between Two Cultures": A Rapid PEER Study Exploring Migrant Communities' Views on Female Genital Mutilation in Essex and Norfolk, UK. FORWARD & National FGM Centre Report, , 2016.

For peer review only

40. Norman K, Hemmings J, Hussein E, et al. "FGM is Always With Us": Experiences, Perceptions and Beliefs of Women Affected by Female Genital Mutilation in London: Results from a PEER Study. London: Options Consultancy Services and FORWARD, 2009.

41. O'Brien O, Baldeh F, Hassan J, et al. My Voice: Participatory Action Research Project with Men, Women and Young People on Female Genital Mutilation (FGM) in Scotland: (Phase 2). Waverly Care, Edinburgh: Queen Margaret University, 2017.

42. O'Brien O, Baldeh F, Sivapatham S, et al. Participatory Action Research Project with Men, Women and Young People on Female Genital Mutilation (FGM) in Scotland: (Phase 1). Waverly Care, Edinburgh: Queen Margaret University, 2016.

43. Palfreyman A, Brown E, Nam S. Understanding Female Genital Mutilation in Birmingham: Findings from a PEER Study: Options Consultancy Services and Birmingham & Solihull Women's Aid, 2011.

44. Recchia N, McGarry J. "Don't judge me": narratives of living with FGM. *Int J Hum Rights Healthc* 2017;10(1):4-13. doi: 10.1108/Ijhrh-10-2016-0016

45. Safari F. A qualitative study of women's lived experience after deinfibulation in the UK. *Midwifery* 2013;29(2):154-8. doi: 10.1016/j.midw.2011.12.005

46. Salad J, Verdonk P, de Boer F, et al. "A Somali girl is Muslim and does not have premarital sex - is vaccination really necessary?" A qualitative study into the perceptions of Somali women in the Netherlands about the prevention of cervical cancer. *Int J Equity Health* 2015;14(1):1-13. doi: 10.1186/s12939-015-0198-3

47. Shaw E. Female circumcision: perceptions of clients and caregivers. *J Am Coll Health* 1985;33(5):193-7. doi: 10.1080/07448481.1985.9939604

48. Shermarke MAA. Understanding the Canadian Community Context of Female Circumcision [M.S.W.]. McGill University, 1996.

49. Straus L, McEwen A, Hussein FM. Somali women's experience of childbirth in the UK: perspectives from Somali health workers. *Midwifery* 2009;25(2):181-6. doi: 10.1016/j.midw.2007.02.002

50. Thierfelder C. Female Genital Mutilation and the Swiss Health Care System. University of Basel, 2003.

51. Thierfelder C, Tanner M, Bodiang CMK. Female genital mutilation in the context of migration: experience of African women with the Swiss health care system. *Eur J Public Health* 2005;15(1):86-90. doi: 10.1093/eurpub/cki120

52. Upvall MJ, Mohammed K, Dodge PD. Perspectives of Somali Bantu refugee women living with circumcision in the United States: a focus group approach. *Int J Nurs Stud* 2009;46(3):360-8. doi: 10.1016/j.ijnurstu.2008.04.009

53. Vangen S, Johansen REB, Sundby J, et al. Qualitative study of perinatal care experiences among Somali women and local health care professionals in Norway. *Eur J Obstet Gynecol Reprod Biol* 2004;112(1):29-35. doi: 10.1016/S0301-2115(03)00313-0

For peer review only

54. Vaughan C, White N, Keogh L, et al. Listening to North Yarra Communities about Female Genital Cutting. Melbourne, Australia: The University of Melbourne, 2014.

55. Vaughan C, White N, Keogh L, et al. Female Genital Mutilation/Cutting in Regional Victoria. Research to Practice. Melbourne, Australia: The University of Melbourne, 2014.

56. Vloeberghs E, van der Kwaak A, Knipscheer J, et al. Coping and chronic psychosocial consequences of female genital mutilation in the Netherlands. *Ethn Health* 2012;17(6):677-95. doi: 10.1080/13557858.2013.771148

57. Wiklund H, Aden AS, Högberg U, et al. Somalis giving birth in Sweden: a challenge to culture and gender specific values and behaviours. *Midwifery* 2000;16(2):105-15. doi: http://dx.doi.org/10.1054/midw.1999.0197

For peer review only

Supplementary File 7: Full CERQual Assessment Table

Summary of review finding	Studies coded to finding	Methodological limitations	Coherence	Adequacy	Relevance	CERQual assessment	Explanation of CERQual score
**Analytical theme 1: communication is key**							
**1.1 Language barriers and interpretation challenges** For women who did not speak the host country language well, communication about FGM/C was hindered by language barriers and problems with accessing interpretation support that was appropriate for discussing highly sensitive and personal issues.	n=31 [1-31]	Moderate concerns regarding methodological limitations<br><br>Most studies contributing to this finding are of high quality (16 studies of high quality, 1 study of low quality, 14 studies of medium quality). However, many studies do not report their theoretical paradigm and present only partial or no reflexivity (n=17). There are serious concerns with 3 studies that do not describe ethics processes, 1 study has an unclear fit of methodology to methods and poor analysis. There are moderate concerns in some studies due to unclear analysis (n=3), and participant voices not well presented (n=2).	No concerns regarding coherence	Minor concerns regarding adequacy<br><br>18 of the 31 studies presented data classified as 'thick' or 'rich'. Over half of the papers (31/57) report this theme.	Minor concerns regarding relevance<br><br>The studies for this finding represented 12 different OECD countries. Many studies (n=12) represented the maternity care contexts, but other contexts were also reflected. Almost half the studies (n=15) were exclusively of Somali women, but other nationalities were also represented.	**High confidence**	There were moderate concerns about methodological limitations, but no (or very minor) concerns about the other domains.
**1.2 "Can't talk, not asked": double silence and cultural taboo** As a private, sensitive and taboo issue, communication around FGM/C was hindered by a double silence. Women reported that it was rarely discussed within their own communities and likewise they found it hard to discuss with health	n=37 [3,4,8,9,13-23,25-47]	Moderate concerns regarding methodological limitations<br><br>Most studies are of high quality (n=21); 15 are medium and 1 is low quality. Several studies had unclear, partial or no	No concerns regarding coherence<br><br>2 studies present slightly divergent views but with reasons (based on the relationship established with	Minor concerns regarding adequacy<br><br>The majority (n=23) of studies related to this review finding provide thick (rich) data. Over half of	No concerns regarding relevance<br><br>Studies come from a wide range of different countries, with a range of participant groups. They also reflected a	**High confidence**	There were moderate concerns about methodological limitations (in particular the predominance of grey literature reports), but there

Summary of review finding	Studies coded to finding	Methodological limitations	Coherence	Adequacy	Relevance	CERQual assessment	Explanation of CERQual score
providers, especially if these (or the interpreters) were male. For this reason, women preferred the topic to be raised by health providers.		reflexivity. There were serious concerns for 5 studies with unclear ethics processes, and 7 studies had unclear methodology and data analysis processes.	healthcare providers).	the included review papers report on this theme (37/57).	range of different healthcare contexts.		were no (or very minor) concerns about the other domains.
**1.3 Cultural (in)sensitivity** Clumsy and intrusive questioning and comments made women feel stigmatised and embarrassed and led them to avoid further discussion or healthcare interactions. By contrast, women valued culturally sensitive and respectful communication from health providers around FGM/C and this facilitated open discussion.	n=34 [3-7,10,13-16,18,19,21,22,24,26-31,33-38,40,42,44,45,48-52]	Moderate concerns regarding methodological limitations<br><br>Three studies are of low methodological quality, 17 of medium quality and 14 of high quality. Seventeen of the studies gave only partial or no detail on methodological perspective or reflexivity, and 6 studies had unclear methods and analysis. In 3 studies, there was inadequate detail about participants' voices.	Moderate concerns regarding coherence<br><br>Some findings were ambiguous and over-lapping with other themes.	Minor concerns regarding adequacy<br><br>Twenty one studies provided rich data and 13 were thin (describing single aspects of this theme or lacking detail in women's voices). Over half the studies in the review reported on this theme (34/57).	No concerns regarding relevance<br><br>Studies related to this theme represented diverse healthcare contexts (10 specific to obstetric care, but other contexts represented), diverse countries (n=9) and diverse population groups.	**Moderate confidence**	Two of the four domains have only moderate confidence. Many studies do report on this theme, but there are some concerns on their quality and some of the findings are ambiguous.
**Analytical theme 2: access to care: influenced by an interaction of multi-level community and health service processes**							
**2.1 Influence of cultural norms** Women's care seeking in relation to FGM/C was strongly influenced by wider cultural norms around sexuality and health, including cultural norms on the importance of pre-marital virginity, avoiding male health providers and lack of familiarity with preventive care-seeking. These factors led women to avoid seeking care unless symptomatic or pregnant.	n=35 [1,3-8,11-14,16-19,23-32,35,37-41,46,53,54]	Moderate concerns regarding methodological limitations<br><br>There are 6 low quality studies, 13 medium and 16 high quality studies. The main methodological limitations relate to unclear fit of methodology, limited ethics descriptions and no reflexivity. Other concerns relate to inadequate	Moderate concerns regarding coherence<br><br>Cultural norms relate to, and influence, a wide range of different healthcare phenomena	Moderate concerns regarding adequacy<br><br>35 papers report on this theme. Seventeen studies provide thin data. There are 12 studies with medium richness explaining 2 or more aspects of this theme and 8 studies provide very rich data accounting	Minor concerns regarding relevance<br><br>The studies are primarily from the UK (n=10) and USA (n=8) contexts, but with good representation of other contexts. The majority of the studies (n=19) are exclusively focused on Somali	**Moderate confidence**	Three of the four domains have moderate concerns related primarily to methodological limitations and 'thin-ness' of the data, but also to the fact that cultural norms influence healthcare in many different ways, not all of which were

For peer review only

Summary of review finding	Studies coded to finding	Methodological limitations	Coherence	Adequacy	Relevance	CERQual assessment	Explanation of CERQual score
		representation of participant voices.		for all aspects of the theme.	participants, and almost half are focused on maternity care contexts (n=15). However, other healthcare contexts are also included, and several studies with 'thick' data reflect a mix of participant groups.		explored in great depth.
**2.2 Influence of the family**<br>Women's decision making in healthcare related to FGM/C was often a collective rather than individual phenomenon, requiring consultation with husbands and other family members.	n=19 [3,4,8,9,11,12,15,23,24,26-28,31,39-41,46,51,54]	Moderate concerns regarding methodological limitations<br><br>Moderate concerns due to methodology and theoretical framework not stated and/or lack of reflexivity (n=13).	Moderate concerns regarding coherence<br><br>Some findings relate to the importance of social support from the family and provide relatively superficial descriptions about the family's role specifically related to decision making.	Minor concerns regarding adequacy<br><br>Rich data from 13 studies. One third of studies in the review (19/57) report on this theme.	Moderate concerns regarding relevance<br><br>Includes studies from different 10 countries. The theme is reported on from studies with various foci, although mainly with Somali participants (n= 12), and relating mainly to obstetric care situations.	**Moderate confidence**	Moderate confidence due to moderate concerns regarding methodological quality, coherence and relevance.
**2.3 Knowledge and information about FGM/C services**<br>Women's knowledge of, and familiarity with, health services was variable. In some cases, women lacked familiarity with the host country health system and this impeded their ability to access care in general. In other cases, women reported being aware of maternity and primary care services. However, there was a particular lack of knowledge and information of FGM/C-related specialist services	n=34 [1,3-5,7,10-12,14,17-29,31,32,36,39,40,44,48,49,54-57]	Moderate concerns regarding methodological limitations<br><br>Two studies had low methodological quality, 18 were designated as medium and 14 were high quality. The majority of the studies are journal articles (n=21). There are serious concerns in two studies where ethical processes are poorly	Moderate concerns regarding coherence<br><br>There was variability in the extent to which studies reported women as being familiar with the general health system in the host country. However, findings relating to women's lack of	Minor concerns regarding adequacy<br><br>The majority of studies in the review report on this theme (34/57). Over half of the studies provide 'rich' (thick) data related to this finding (n=23); 11 provide thin data.	Minor concerns regarding relevance<br><br>The contributing studies cover 11 countries. Just under half focus exclusively on Somali women (n=16); one third of studies focus on the maternity context (n=13). However, overall, there is a good mix of focus	**Moderate confidence**	Moderate confidence due to moderate concerns regarding methodological quality and coherence.

For peer review only

For peer review only - http://bmjopen.bmj.com/site/about/guidelines.xhtml

Summary of review finding	Studies coded to finding	Methodological limitations	Coherence	Adequacy	Relevance	CERQual assessment	Explanation of CERQual score
and where and when it may be appropriate to seek help.		described; there is lack of clarity in the fit of methods to methodology in 3 studies and 16 studies lack a discussion of reflexivity.	awareness of specialist FGM/C services were coherent across the studies.		and participant countries.		
**2.4 'Hit and miss' care** When accessing healthcare, women reported that the identification of FGM/C and provision of appropriate treatment or referrals could be a "hit and miss" process, depending upon individual provider characteristics and practices rather than being a result of standardised organisational systems and processes. Continuity of care was rare, but when it occurred, women reported that it helped to facilitate communication and access to appropriate care.	n= 38 3-8,10,12-22,24,25,27-31,33,34,36-38,41,43,44,47,48,54,56,57	Moderate concerns regarding methodological limitations<br><br>There is variable quality of studies relating to this finding: high quality (n=20), medium quality (n=15) and low quality (n=3). There are serious concerns with six studies that report unclear ethical procedures, and moderate concerns with 11 studies that report no or unclear methodological perspectives. A large number of studies (n=21) had no or partial reflexivity.	No concerns regarding coherence	Minor concerns regarding adequacy<br><br>38/57 studies report on this theme. Most studies (n=32) contribute relatively rich data to this review finding.	No concerns regarding relevance<br><br>Studies represent 13 countries and a mixture of participant backgrounds and settings.	**High Confidence**	Three out of four domains have no or only minor concerns. Lack of reflexivity as a methodological limitation is less of a concern for this finding as it is not such a highly sensitive topic.
**Analytical theme 3: cultural and bodily dissonance: striving for cultural and bodily integrity**							
**3.1 Moving from normal to different** Women described a shift from feeling 'normal' to 'different' about FGM/C, their bodies and their culture as part of their transition to living in the host country. Some women started to become aware of the suffering and symptoms that their FGM/C may have caused them. Some women started to question and/or resist previously taken for granted aspects	n=40 3,4,7,8,10,12-16,18-31,33,34,36-39,41,43,46-50,53,54,57	Minor concerns regarding methodological limitations<br><br>Thirty five of the studies are rated as high or medium quality. Five are low quality. However, many studies failed to report issues relating to reflexivity.	No concerns regarding coherence	Minor concerns regarding adequacy<br><br>The majority of studies in the review present rich data on this theme. Eight studies provide only thin data where detailed information is not given.	No concerns regarding relevance<br><br>The studies represented mixed contexts, 13 countries and a range of population groups. Eighteen out of 40 studies had an exclusively Somali sample.	**High Confidence**	All domains report no or only minor concerns.

For peer review only

Summary of review finding	Studies coded to finding	Methodological limitations	Coherence	Adequacy	Relevance	CERQual assessment	Explanation of CERQual score
of their culture and to feel uncomfortable with their own bodies. However, women also reported feeling shame and anger at being labelled as different and mutilated and felt that their culture was misunderstood.							
**3.2 Threat to the self: re-living FGM/C pain during clinical interventions**<br>Many studies reported women's experiences of gynaecological clinical interventions and childbirth as events which threatened their sense of bodily and cultural integrity. Women reported great pain, suffering, fear and apprehension around clinical interventions – and related this to their previous healthcare experiences and, especially, to re-living the original trauma they had experienced during their FGM/C. These emotions were exacerbated in encounters where women felt a loss of control or lack of respect.	n=28<br>1,3,7,9-11,13-15,17,19,20,23,25,27-30,33,37,38,41,43,48,51,54,55,57	Moderate concerns regarding methodological limitations<br><br>Serious concerns for 3 studies due to no ethics statements, and poor descriptions of methodology, and unclear fit of methods.<br>There are moderate concerns for 9 studies due to lack of stated methodology, inadequate participant voices and lack of reflexivity. Sixteen studies are rated high quality.	Moderate concerns regarding coherence<br><br>There was variability in the extent to which studies reported direct experiences of re-living trauma as opposed to avoidance of care or services due to apprehension of pain, trauma and anxiety.	No concerns regarding adequacy.<br><br>The majority of studies present rich data of women's voices, with just four studies presenting thin data on this particular finding. Almost half the papers in the review report on this finding.	Minor concerns regarding relevance.<br><br>Data is spread across several countries and focussed on different healthcare settings. Eleven studies have an exclusively Somali sample. The focus is specifically on obstetric care in 10 studies. Nine studies are based in the UK.	**Moderate confidence**	Moderate confidence due to moderate concerns regarding methodological quality and coherence.
**3.3 Being opened: complexities around deinfibulation**<br>For women with type III FGM/C, decision making around 'being opened' (deinfibulation) was influenced by cultural norms and medical imperatives. Some younger women specifically sought out medical deinfibulation before marriage as a way of asserting control over their bodies and lives, but the majority of women considered medical deinfibulation	n= 28<br>3,4,8,10,12,14,17,19,20,22,24,25,28,29,33,35-41,46,48,49,53,54,57	Moderate concerns regarding methodological limitations<br><br>This finding is primarily derived from high quality studies (n=15) even though many are either research reports or theses. Half of the studies are journal articles but these include papers of low and medium methodological quality	Moderate concerns regarding coherence<br><br>This was because some studies reported specifically on deinfibulation related to obstetric care, whereas others included this topic in discussions on other issues, hence	Minor concerns regarding adequacy<br><br>Twenty eight studies present this theme. Three of these provide only 'thin' data in relation to this finding. Data from the other studies is relatively rich, providing	Moderate concerns regarding relevance<br><br>Although several of these studies are, as expected, in maternity settings (n=8), there is a wide diversity of other study foci presenting on this theme and across different nationalities that	**Moderate confidence**	Moderate confidence due to moderate concerns regarding methodological quality, coherence and relevance.

For peer review only

Summary of review finding	Studies coded to finding	Methodological limitations	Coherence	Adequacy	Relevance	CERQual assessment	Explanation of CERQual score
only in the context of pregnancy, and the majority preferred the procedure to be carried out during labour (rather than antenatally) to avoid additional cutting and pain.		stemming from poor or no reporting of theoretical perspective and methodology, and poor analysis or presentation of participant voices. There are serious concerns with two papers because of no ethics description (n=1) and very weak methodology (n=1).	the findings were less specific, but generally still consistent with the theme.	explanatory insights or in-depth descriptions of women's views.	practise type III FGM/C. However, there is little in-depth exploration of any potential differences between different ethnic groups.		
**3.4 Being changed: complexities around re-infibulation** For women with type III FGM/C, 'being opened' (deinfibulation) was reported as a process involving significant emotional, physical, social and relational adjustments as their bodies became changed. Some women felt ambivalent about these changes and sometimes wanted to be closed again (reinfibulated) to varying degrees, feeling upset if their requests were denied by health providers.	n= 19 3,4,8,10,20,22,28,29,35-37,41,46,48,49,52-54,57	Moderate concerns regarding methodological limitations<br><br>Moderate concerns due to lack of reflexivity in 13 of the papers.	Minor concerns regarding coherence<br><br>Not all papers reported on the issue of requesting requests for reinfibulation.	Minor concerns regarding adequacy<br><br>There are 19 papers reporting on this theme with the majority contributing rich data (n=16). Three studies present thin data.	Moderate concerns regarding relevance<br><br>The data is well spread across the included studies, representing 11 countries, involving diverse participants groups with FGM/C III. However, there is little in-depth exploration of any potential differences between different ethnic groups.	**Moderate confidence**	Moderate confidence due to moderate concerns regarding methodological quality and relevance.
**Analytical theme 4: disempowering care encounters**							
**4.1 Being exposed and humiliated** Many studies reported women feeling exposed, objectified and 'on display' during healthcare encounters – due to the health provider's reactions to their FGM/C. They reported feeling a lack of concern for their privacy or dignity and felt humiliated and stigmatised. These experiences led	n=40 1,3,4,7,9,10,13-31,33-38,42-45,48-51,54	Moderate concerns regarding methodological limitations<br><br>The majority of studies are high quality (n=28). There are moderate concerns in some studies due to unclear presentation of the	No concerns regarding coherence	Minor no concerns regarding adequacy<br><br>Most papers (n=31) provide rich data in relation to this theme. Nine papers provide only thin data.	Minor concerns regarding relevance<br><br>There is good representation of OECD countries, but 14 of the 40 studies are from the UK. A range of population	**High confidence**	A large of number of papers and highly coherent data is presented in this theme with only a small number of poor quality studies. Data covers a

Summary of review finding	Studies coded to finding	Methodological limitations	Coherence	Adequacy	Relevance	CERQual assessment	Explanation of CERQual score
them to lose trust in the health provider and 'the system' more generally.		research paradigm (n=8) and no discussion of reflexivity (n=8). There are serious concerns regarding the data from 6 papers due to lacking ethics descriptions (n=2), unclear methodological fit with methods (n=3) and weak analysis/detail of participant voices (n=1).		Over half the papers in the review report on this theme (40/57).	groups are included, but 17 studies focus exclusively on Somali women. A range of healthcare contexts are covered (13 out of 40 papers focus specifically on obstetric care situations).		mixture of contexts and not only on maternity settings.
**4.2 Being judged and stereotyped** Women reported feeling judged and discriminated against within some of their healthcare encounters. They reported feeling that health providers made negative assumptions about them and provided sub-optimal care based on racial, religious or other stereotypes and misconceptions about their culture in general as well as about FGM/C. Such experiences caused distress, anger and avoidance of the health provider or service.	n= 36 2-10,13,15,16,18,19,22,24-34,36,37,39,44,45,47-50,54	Moderate concerns regarding methodological quality<br><br>There are methodological concerns across studies due to lack of reflexivity, no stated paradigm and serious concerns resulting from no or inadequate ethics statements (n=4), inadequate participant voices (n=1) and a mismatch between methodology and methods (n=1).	Minor concerns regarding coherence<br><br>There was minor variability across studies in terms of the ways in which this phenomenon was experienced.	Minor concerns regarding adequacy<br><br>36 papers report on this theme. The majority of these papers report relatively rich or in-depth findings with only 2 studies providing thin data.	Minor concerns regarding relevance<br><br>Studies span several contexts and 13 countries, involving mainly Somali participants (n=15) but with several studies involving more than 3 participant groups (n=15) and 2 other participant groups (n=4).	**High confidence**	Even though there are some studies with serious methodological concerns (n=6), there are good quality studies across mixed contexts and participant groups, with very minor concerns on coherence, adequacy and relevance.
**4.3 Lacking choice, power and control** Women reported experiences when they felt that they lacked control within the healthcare encounter, especially in the maternity setting and around key interventions such as caesarean sections or episiotomies. They reported feeling excluded from healthcare decision making, not listened to, at the mercy of the system and unable to express their needs. Such experiences could	n=32 3-5,7,9,10,12,15-20,24,25,27-35,37,38,40,41,47-49,54,56	Moderate concerns regarding methodological quality<br><br>Overall most studies are high quality. Some studies present moderate concerns (n=15) due to lack of reflexivity, and no stated paradigm. Two studies have serious concerns due	No concerns regarding coherence	Minor concerns regarding adequacy<br><br>A large number of studies report this theme. Most of these provide rich data to this theme, with only 8 studies providing 'thin' accounts.	Minor concerns regarding relevance<br><br>The studies span 13 different OECD countries. The majority of studies involve Somali participants (n=20), and the majority refer to obstetric care	**High confidence**	Although there are moderate methodological concerns due to mainly studies not stating their methodology or theoretical paradigm, the conduct of majority studies was good and there

Summary of review finding	Studies coded to finding	Methodological limitations	Coherence	Adequacy	Relevance	CERQual assessment	Explanation of CERQual score
be highly traumatising. Attempts to assert themselves were sometimes construed as being 'difficult'.		to lack of ethics descriptions.			contexts (n=18) – as stated in the theme.		are only minor concerns regarding coherence, adequacy and relevance.
**4.4 Feeling unsafe and vulnerable** Women expected health providers to be knowledgeable, competent and confident in managing their FGM/C. Some women reported times when this did not seem to be the case and where they felt their care had been adversely affected as a result of poor provider skills. In such situations, women felt unsafe and highly vulnerable, describing painful and traumatic clinical experiences. Feelings of vulnerability were heightened for women who lacked social support.	n= 46 [2-7,9,10,12-29,31-41,43,45,47-49,54-57]	Moderate concerns regarding methodological quality<br><br>Half of the papers are high quality with only minor concerns (n= 23). Limitations in studies are mainly due to partial or lack of reflexivity which is deemed to be of particular relevance to the reporting of this theme. Five studies have more serious concerns due to lacking ethics statements, poor methodology and partial participant voices.	No concerns regarding coherence	No concerns regarding adequacy<br><br>This theme has a high number of studies contributing to it (n=46). The majority of these provide rich or in-depth data (n=42) in relation to this finding.	No concerns regarding relevance<br><br>Studies involve mixed participant groups, and across a range of health contexts, topics and countries.	**High Confidence**	There are a few studies with serious methodological concerns, and some concern regarding the lack of reflexivity. However the data is very rich and consistent across contexts, hence there are no concerns regarding adequacy, coherence or relevance.
**Analytical theme 5: positive care encounters**							
**5.1 Trusting and appreciating providers and the system** Women appreciated the good medical services available in the host country. Women also expressed great appreciation for providers who made them feel safe and respected. Such providers were described as knowledgeable and experienced, who treated women with respect, who understood their individual needs and who involved them in their care. Women described these	n= 34 [3-5,7-10,12,14,17-22,26-32,34-39,41,47,48,54-56]	Moderate concerns regarding methodological limitations<br><br>There are 3 studies with serious concerns for not having clear ethics, no paradigm and poor description of methods. There are moderate concerns in 13 papers due to no stated paradigm, lack	No concerns regarding coherence	No concerns regarding adequacy<br><br>A large number of papers report this theme (n=34). The majority of papers (n=29) provide rich or in-depth data in relation to this finding. Only 5 papers report thin data	No concerns regarding relevance<br><br>Diverse studies across different countries. Fourteen studies are exclusively with Somali women, but other groups (or mixed samples) are also represented. This theme occurs in	**High Confidence**	There are some concerns due to methodological limitations. However the data is rich and consistent across contexts, and there are no concerns regarding adequacy, coherence or relevance.

For peer review only

Summary of review finding	Studies coded to finding	Methodological limitations	Coherence	Adequacy	Relevance	CERQual assessment	Explanation of CERQual score
characteristics as facilitating trust and leading to open communication about FGM/C. This, in turn, enhanced the likelihood of women being involved in, and receiving, appropriate care.		of reflexivity or inadequate participant voices.			relation to a range of health care contexts.		
**5.2 Voicing healthcare needs and preferences** Women's recommendations for good quality safe FGM/C related care and services they could trust included: (i) interpersonal provider characteristics and behaviours (such as providers being willing and able to talk about FGM/C, providers being skilled and knowledgeable around FGM/C, providers offering culturally sensitive and respectful care) and (ii) service organisation issues (such as having specialist services for FGM/C, being given information and awareness about FGM/C-related services, and involving women and affected communities in FGM service development).	n=45 [1,3-10,12-17,19-22,24-26,28-33,35-39,41-45,47-50,53,54,57]	Moderate concerns regarding methodological limitations.<br><br>The major limitation across studies relates to lack of reflexivity. It is unclear to what extent this may have impacted upon this particular review finding.	Minor concerns regarding coherence<br><br>Studies report on several aspects of what women would like in regard to healthcare services related to FGM/C. Not all studies report on all the different elements of this theme.	Minor concerns regarding adequacy<br><br>45 studies report this theme. The majority have rich or in-depth data (n=31), but 14 have relatively thin data due to limited representation of participant voices or only small aspects of the theme reported on.	No concerns regarding relevance<br><br>Studies report these recommendations across all care settings for women (including studies which did not specify a care setting). This finding included studies from 13 countries and a good mix of participants, with highest being among Somali only (n=19) and a relatively higher number of studies from the UK (n=16).	**High Confidence**	Lack of reflexivity may an issue, however the data is highly consistent across contexts, hence there are no or only minor concerns regarding adequacy, coherence or relevance.

For peer review only

References for CERQual Assessment

1. Abdullahi A, Copping J, Kessel A, et al. Cervical screening: perceptions and barriers to uptake among Somali women in Camden. *Public Health* 2009;123(10):680-5. doi: 10.1016/j.puhe.2009.09.011

2. Ahlberg BM, Krantz I, Lindmark G, et al. 'It's only a tradition': making sense of eradication interventions and the persistence of female 'circumcision' within a Swedish context. *Crit Soc Policy* 2004;24(1):50-78. doi: 10.1107/0261018304039679

3. Berggren V, Bergstrom S, Edberg A-K. Being different and vulnerable: experiences of immigrant African women who have been circumcised and sought maternity care in Sweden. *J Transcult Nurs* 2006;17(1):50-7. doi: 10.1177/1043659605281981

4. Betts V. Transnational Gender, Sexuality and Identity Construction: Ambivalent Subjectivities of Somali Refugee Women in the GTA. MA Thesis, The University of Guelph, 2011.

5. Bulman KH, McCourt C. Somali refugee women's experiences of maternity care in west London: a case study. *Crit Public Health* 2002;12(4):365-80.

6. Carroll J, Epstein R, Fiscella K, et al. Caring for Somali women: implications for clinician-patient communication. *Patient Educ Couns* 2007;66(3):337-45. doi: 10.1016/j.pec.2007.01.008

7. Chalmers B, Omer-Hashi K. What Somali women say about giving birth in Canada. *J Reprod Infant Psychol* 2002;20(4):267-82. doi: 10.1080/0264683021000033183

8. Degni F, Suominen SB, El Ansari W, et al. Reproductive and maternity health care services in Finland: perceptions and experiences of Somali-born immigrant women. *Ethn Health* 2014;19(3):348-66. doi: 10.1080/13557858.2013.797567

9. d'Entremont M, Smythe L, McAra-Couper J. The sounds of silence: a hermeneutic interpretation of childbirth post excision. *Health Care Women Int* 2014;35(3):300-19. doi: 10.1080/07399332.2013.838245

10. Gali MA. Female Circumcision: A Transcultural Study of Attitudes, Identity and Reproductive Health of East African Immigrants [Degree of Doctor of Philosophy in Psychology]. The Wright Institute, University of Berkeley, 1997.

11. Ghebre RG, Sewali B, Osman S, et al. Cervical cancer: barriers to screening in the Somali community in Minnesota. *J Immigr Minor Health* 2015;17(3):722-28. doi: 10.1007/s10903-014-0080-1

12. Hill N, Hunt E, Hyrkas K. Somali immigrant women's health care experiences and beliefs regarding pregnancy and birth in the United States. *J Transcult Nurs* 2012;23(1):72-81. doi: 10.1177/1043659611423828 [published Online First: 2011/11/05]

13. Hussein E. Women's Experiences, Perceptions and Attitudes of Female Genital Mutilation: The Bristol PEER Study. London: FORWARD, 2010.

For peer review only

14. Hussen MA. Services for Women with Female Genital Mutilation in Christchurch: Perspectives of Women and their Health Providers. University of Canterbury, 2014.

15. Fawcett L. Somali Refugee Women and their U.S. Healthcare Providers: Knowledge, Perceptions and Experiences of Childbearing [Doctor of Philosophy]. Arizona State University, 2014.

16. Maternity Action. Women's Voices on Health: Addressing Barriers to Accessing Primary Care. London: Women’s Health and Equality Consortium, 2014.

17. Moxey JM, Jones LL. A qualitative study exploring how Somali women exposed to female genital mutilation experience and perceive antenatal and intrapartum care in England. *BMJ Open* 2016;6(1):e009846. doi: 10.1136/bmjopen-2015-009846

18. Murray L, Windsor C, Parker E, et al. The experiences of African women giving birth in Brisbane, Australia. *Health Care Women Int* 2010;31(5):458-72. doi: 10.1080/07399330903548928

19. Norman K, Hemmings J, Hussein E, et al. "FGM is Always With Us": Experiences, Perceptions and Beliefs of Women Affected by Female Genital Mutilation in London: Results from a PEER Study. London: Options Consultancy Services and FORWARD, 2009.

20. O’Brien O, Baldeh F, Hassan J, et al. My Voice: Participatory Action Research Project with Men, Women and Young People on Female Genital Mutilation (FGM) in Scotland: (Phase 2). Waverly Care, Edinburgh: Queen Margaret University, 2017.

21. O’Brien O, Baldeh F, Sivapatham S, et al. Participatory Action Research Project with Men, Women and Young People on Female Genital Mutilation (FGM) in Scotland: (Phase 1). Waverly Care, Edinburgh: Queen Margaret University, 2016.

22. Palfreyman A, Brown E, Nam S. Understanding Female Genital Mutilation in Birmingham: Findings from a PEER Study: Options Consultancy Services and Birmingham & Solihull Women’s Aid, 2011.

23. Salad J, Verdonk P, de Boer F, et al. "A Somali girl is Muslim and does not have premarital sex - is vaccination really necessary?" A qualitative study into the perceptions of Somali women in the Netherlands about the prevention of cervical cancer. *Int J Equity Health* 2015;14(1):1-13. doi: 10.1186/s12939-015-0198-3

24. Straus L, McEwen A, Hussein FM. Somali women's experience of childbirth in the UK: perspectives from Somali health workers. *Midwifery* 2009;25(2):181-6. doi: 10.1016/j.midw.2007.02.002

25. Thierfelder C, Tanner M, Bodiang CMK. Female genital mutilation in the context of migration: experience of African women with the Swiss health care system. *Eur J Public Health* 2005;15(1):86-90. doi: 10.1093/eurpub/cki120

26. Upvall MJ, Mohammed K, Dodge PD. Perspectives of Somali Bantu refugee women living with circumcision in the United States: a focus group approach. *Int J Nurs Stud* 2009;46(3):360-8. doi: 10.1016/j.ijnurstu.2008.04.009

For peer review only

27. Vangen S, Johansen REB, Sundby J, et al. Qualitative study of perinatal care experiences among Somali women and local health care professionals in Norway. *Eur J Obstet Gynecol Reprod Biol* 2004;112(1):29-35. doi: 10.1016/S0301-2115(03)00313-0

28. Vaughan C, White N, Keogh L, et al. Listening to North Yarra Communities about Female Genital Cutting. Melbourne, Australia: The University of Melbourne, 2014.

29. Vaughan C, White N, Keogh L, et al. Female Genital Mutilation/Cutting in Regional Victoria. Research to Practice. Melbourne, Australia: The University of Melbourne, 2014.

30. Vloeberghs E, van der Kwaak A, Knipscheer J, et al. Coping and chronic psychosocial consequences of female genital mutilation in the Netherlands. *Ethn Health* 2012;17(6):677-95. doi: 10.1080/13557858.2013.771148

31. Wiklund H, Aden AS, Högberg U, et al. Somalis giving birth in Sweden: a challenge to culture and gender specific values and behaviours. *Midwifery* 2000;16(2):105-15. doi: http://dx.doi.org/10.1054/midw.1999.0197

32. Ameresekere M, Borg R, Frederick J, et al. Somali immigrant women's perceptions of cesarean delivery and patient-provider communication surrounding female circumcision and childbirth in the USA. *Int J Gynecol Obstet* 2011;115(3):227-30. doi: 10.1016/j.ijgo.2011.07.019

33. Baldeh F. Obstetric Care in Scotland: The Experience of Women who have Undergone Female Genital Mutilation (FGM) [MSc Thesis]. Queen Margaret University, 2013.

34. Behrendt A. Listening to African Voices: Female Genital Mutilation/Cutting among Immigrants in Hamburg: Knowledge, Attitudes and Practice. Hamburg, Germany: Plan, 2011.

35. Beine K, Fullerton J, Palinkas L, et al. Conceptions of prenatal care among Somali women in San Diego. *J Nurs Midwifery* 1995;40(4):376-81. doi: Doi 10.1016/0091-2182(95)00024-E

36. Bravo Pastor del Mar M. [Women Subjected to Female Genital Mutilation: Knowledge for the Nursing Discipline] La Voz de las Mujeres Sometidas a Mutilación Genital Femenina: Saberes Para la Disciplina Enfermera [Tesis Doctoral]. University of Murcia, 2014.

37. Asefaw F. [Female Genital Mutilation: A Field Study with Special Consideration of the Background as well as the Health and Psychosexual Consquences for those Affected and their Partners in Eritrea and in Germany] Weibliche Genitalbeschneidung: Eine Feldstudie Unter besonderer Berücksichtigung der Hintergründe Sowie der Gesundheitlichen und Psychosexuellen Folgen für Betroffene und Partner in Eritrea und Deutschland. Humboldt University of Berlin, 2007.

38. Glazer E. Embodiment, Pain and Circumcision in Somali-Canadian Women [M.Sc.]. University of Toronto, 2012.

For peer review only

39. Jones A. Working Psychologically with Female Genital Mutilation: An Exploration of the Views and Experiences of Women who have Experienced FGM and of Clinical Psychologists. University of East London, 2010.

40. Khaja K. Female Circumcision: Life Histories of Somali Women [Doctor of Philosophy]. The University of Utah, 2004.

41. Lundberg PC, Gerezgiher A. Experiences from pregnancy and childbirth related to female genital mutilation among Eritrean immigrant women in Sweden. *Midwifery* 2008;24(2):214-25. doi: 10.1016/j.midw.2006.10.003

42. Ahmed M. Attitudes towards FGM among Somali women living in the UK. In: Momoh C, ed. Female Genital Mutilation. London: Radcliffe 2005:95-117.

43. McNeely S, Christie-de Jong F. Somali refugees' perspectives regarding FGM/C in the US. *Int J Migr Health Soc Care* 2016;12(3):157-69. doi: 10.1108/Ijmhsc-09-2015-0033

44. Norman K, Gegzabher SB, Otoo-Oyortey N. "Between Two Cultures": A Rapid PEER Study Exploring Migrant Communities' Views on Female Genital Mutilation in Essex and Norfolk, UK. FORWARD & National FGM Centre Report, , 2016.

45. Recchia N, McGarry J. "Don't judge me": narratives of living with FGM. *Int J Hum Rights Healthc* 2017;10(1):4-13. doi: 10.1108/Ijhrh-10-2016-0016

46. Safari F. A qualitative study of women's lived experience after deinfibulation in the UK. *Midwifery* 2013;29(2):154-8. doi: 10.1016/j.midw.2011.12.005

47. Shermarke MAA. Understanding the Canadian Community Context of Female Circumcision [M.S.W.]. McGill University, 1996.

48. Glover J, Liebling H, Barrett H, et al. The psychological and social impact of female genital mutilation: a holistic conceptual framework. *J Int Stud* 2017;10(2):219-38.

49. Guerin PB, Allotey P, Elmi FH, et al. Advocacy as a means to an end: assisting refugee women to take control of their reproductive health needs. *Women Health* 2006;43(4):7-25.

50. Khaja K, Lay K, Boys S. Female circumcision: toward an inclusive practice of care. *Health Care Women Int* 2010;31(8):686-99. doi: 10.1080/07399332.2010.490313

51. Abdi R. Carving culture: Creating identity through female genital cutting. *Durham Anthropology Journal* 2012;18(1):115-53.

52. Thierfelder C. Female Genital Mutilation and the Swiss Health Care System. University of Basel, 2003.

53. Johansen RE. Undoing female genital cutting: perceptions and experiences of infibulation, defibulation and virginity among Somali and Sudanese migrants in Norway. *Cult Health Sex* 2017;19(4):528-42. doi: 10.1080/13691058.2016.1239838

For peer review only

54. Maier C. [Echoes of Silence: Voices of Concern for Genital Mutilation in African Immigrant Women in Vienna: An Ethnological Study] Echo des Schweigens: Stimmen der Betroffenheit zur Genitalverstümmelung bei Afrikanischen Immigrantinnen in Wien: Ethnologische Studie. University of Vienna, 2003.

55. Ballesteros Meseguer C, Almansa Martinez P, Pastor Bravo MdM, et al. [The voice of women subjected to female genital mutilation in the region of Murcia, Spain] La Voz de las mujeres sometidas a mutilacion genital femenina en la region de Murcia. *Gac Sanit* 2014;28(4):287-91. doi: 10.1016/j.gaceta.2014.02.006

56. Essén B, Johnsdotter S, Hovelius B, et al. Qualitative study of pregnancy and childbirth experiences in Somalian women resident in Sweden. *Br J Obstet Gynaecol* 2000;107(12):1507-12.

57. Shaw E. Female circumcision: perceptions of clients and caregivers. *J Am Coll Health* 1985;33(5):193-7. doi: 10.1080/07448481.1985.9939604

For peer review only

PRISMA 2009 Checklist

Section/topic	#	Checklist item	Reported on page #
**TITLE**			
Title	1	Identify the report as a systematic review, meta-analysis, or both.	1
**ABSTRACT**			
Structured summary	2	Provide a structured summary including, as applicable: background; objectives; data sources; study eligibility criteria, participants, and interventions; study appraisal and synthesis methods; results; limitations; conclusions and implications of key findings; systematic review registration number.	2
**INTRODUCTION**			
Rationale	3	Describe the rationale for the review in the context of what is already known.	4-6
Objectives	4	Provide an explicit statement of questions being addressed with reference to participants, interventions, comparisons, outcomes, and study design (PICOS).	5
**METHODS**			
Protocol and registration	5	Indicate if a review protocol exists, if and where it can be accessed (e.g., Web address), and, if available, provide registration information including registration number.	8
Eligibility criteria	6	Specify study characteristics (e.g., PICOS, length of follow-up) and report characteristics (e.g., years considered, language, publication status) used as criteria for eligibility, giving rationale.	9
Information sources	7	Describe all information sources (e.g., databases with dates of coverage, contact with study authors to identify additional studies) in the search and date last searched.	9
Search	8	Present full electronic search strategy for at least one database, including any limits used, such that it could be repeated.	Supplementary file 1
Study selection	9	State the process for selecting studies (i.e., screening, eligibility, included in systematic review, and, if applicable, included in the meta-analysis).	9
Data collection process	10	Describe method of data extraction from reports (e.g., piloted forms, independently, in duplicate) and any processes for obtaining and confirming data from investigators.	11
Data items	11	List and define all variables for which data were sought (e.g., PICOS, funding sources) and any assumptions and simplifications made.	11-12
Risk of bias in individual studies	12	Describe methods used for assessing risk of bias of individual studies (including specification of whether this was done at the study or outcome level), and how this information is to be used in any data synthesis.	10
Summary measures	13	State the principal summary measures (e.g., risk ratio, difference in means).	11-12
Synthesis of results	14	Describe the methods of handling data and combining results of studies, if done, including measures of consistency (e.g., I^2) for each meta-analysis.	11-12

For peer review only

PRISMA 2009 Checklist

Section/topic	#	Checklist item	Reported on page #
Risk of bias across studies	15	Specify any assessment of risk of bias that may affect the cumulative evidence (e.g., publication bias, selective reporting within studies).	10, 13, 33
Additional analyses	16	Describe methods of additional analyses (e.g., sensitivity or subgroup analyses, meta-regression), if done, indicating which were pre-specified.	11-12
**RESULTS**			
Study selection	17	Give numbers of studies screened, assessed for eligibility, and included in the review, with reasons for exclusions at each stage, ideally with a flow diagram.	13
Study characteristics	18	For each study, present characteristics for which data were extracted (e.g., study size, PICOS, follow-up period) and provide the citations.	14-18
Risk of bias within studies	19	Present data on risk of bias of each study and, if available, any outcome level assessment (see item 12).	20&14-18
Results of individual studies	20	For all outcomes considered (benefits or harms), present, for each study: (a) simple summary data for each intervention group (b) effect estimates and confidence intervals, ideally with a forest plot.	n/a
Synthesis of results	21	Present results of each meta-analysis done, including confidence intervals and measures of consistency.	20-33
Risk of bias across studies	22	Present results of any assessment of risk of bias across studies (see Item 15).	20, 33
Additional analysis	23	Give results of additional analyses, if done (e.g., sensitivity or subgroup analyses, meta-regression [see Item 16]).	n/a
**DISCUSSION**			
Summary of evidence	24	Summarize the main findings including the strength of evidence for each main outcome; consider their relevance to key groups (e.g., healthcare providers, users, and policy makers).	33-34
Limitations	25	Discuss limitations at study and outcome level (e.g., risk of bias), and at review-level (e.g., incomplete retrieval of identified research, reporting bias).	37
Conclusions	26	Provide a general interpretation of the results in the context of other evidence, and implications for future research.	33-37
**FUNDING**			
Funding	27	Describe sources of funding for the systematic review and other support (e.g., supply of data); role of funders for the systematic review.	39

From: Moher D, Liberati A, Tetzlaff J, Altman DG, The PRISMA Group (2009). Preferred Reporting Items for Systematic Reviews and Meta-Analyses: The PRISMA Statement. PLoS Med 6(6): e1000097. doi:10.1371/journal.pmed1000097

For more information, visit: www.prisma-statement.org.

For peer review only - http://bmjopen.bmj.com/site/about/guidelines.xhtml
